# Nanopore long-read RNA-seq and absolute quantification delineate transcription dynamics in early embryo development of an insect pest

**DOI:** 10.1038/s41598-021-86753-7

**Published:** 2021-04-12

**Authors:** Anthony Bayega, Spyros Oikonomopoulos, Maria-Eleni Gregoriou, Konstantina T. Tsoumani, Antonis Giakountis, Yu Chang Wang, Kostas D. Mathiopoulos, Jiannis Ragoussis

**Affiliations:** 1 McGill Genome Centre, Department of Human Genetics, McGill University, Montréal, Québec Canada; 2 Laboratory of Molecular Biology and Genomics, Department of Biochemistry and Biotechnology, University of Thessaly, Larissa, Greece; 3 Department of Bioengineering, McGill University, Montréal, Québec Canada

**Keywords:** Computational biology and bioinformatics, Developmental biology, Embryogenesis

## Abstract

The olive fruit fly, *Bactrocera oleae*, is the most important pest for the olive fruit but lacks adequate transcriptomic characterization that could aid in molecular control approaches. We apply nanopore long-read RNA-seq with internal RNA standards allowing absolute transcript quantification to analyze transcription dynamics during early embryo development for the first time in this organism. Sequencing on the MinION platform generated over 31 million reads. Over 50% of the expressed genes had at least one read covering its entire length validating our full-length approach. We generated a de novo transcriptome assembly and identified 1768 new genes and a total of 79,810 isoforms; a fourfold increase in transcriptome diversity compared to the current NCBI predicted transcriptome. Absolute transcript quantification per embryo allowed an insight into the dramatic re-organization of maternal transcripts. We further identified *Zelda* as a possible regulator of early zygotic genome activation in *B. oleae* and provide further insights into the maternal-to-zygotic transition. These data show the utility of long-read RNA in improving characterization of non-model organisms that lack a fully annotated genome, provide potential targets for sterile insect technic approaches, and provide the first insight into the transcriptome landscape of the developing olive fruit fly embryo.

## Introduction

Short-read RNA sequencing (RNA-seq) is currently the most widely used high throughput approach to gene expression profiling. However, for non-model organisms which usually lack a well structurally and functionally annotated genome long-read RNA-seq has been shown to perform better^1^. Long-read RNA-seq provides better recovery of large transcripts, new gene locus identification, better support and higher accuracy in splice junctions, more accurate exon/intron structure, and gene model correction^1^. Given a comparable amount of sequencing depth, long reads usually detect more alternative splicing events than short-read RNA-seq^1^ providing more accurate transcriptome profiling and quantification of isoform expression. We and others have shown that the Oxford Nanopore Technologies (ONT) MinION sequencing platform provides full-length transcript resolution^2,3^ and enables identification of hitherto unknown genes and isoforms^4–7^ and provides gene expression quantification that is comparable to current standards^4–7^.

Relative quantification of gene expression is the most common method in RNA-seq. Relative normalization, however, is very sensitive to global changes in gene expression. In order to profile gene expression in rapidly changing biological systems such as developing embryos where precise and coordinated dramatic shifts in transcriptome kinetics occur in quick succession direct absolute quantification has been shown to perform superiorly to relative quantification^8^. Absolute quantification can be achieved by addition of a predetermined amount of known RNAs into the sample prior to library generation. One example of such RNAs is the ERCC mix; a solution of 92 different poly(A) RNAs at different concentrations developed by the External RNA Control Consortium^9^. ERCC spike-in act as an internal standard and have been successfully used to determine the absolute number of transcripts per embryo^8^. Coupled with time-course experimentation, absolute quantification enables direct measurement of transcript kinetics thus providing a quantitative understanding of the rate of change of transcript copy numbers with time providing both magnitude and direction of change in gene expression.

In this work, we use long-read RNA-seq and absolute quantification to elucidate the transcription dynamics in the developing embryos of the olive fruit fly collected at hourly intervals for the first 6 h after egg laying (AEL). Olive fruit flies (*Bactrocera oleae*) are insects of huge economic importance in the olive agribusiness industry costing an estimated 800 million US dollars annually^10^ due to the devastating physical damage they inflict on olive fruits. Whereas the sterile insect technique (SIT) has been effective in controlling the closely related Mediterranean fruit fly (*Ceratitis capitata*) this method was shown to be less effective in pilot experiments with the olive fruit fly. One of the potential avenues to improve *B. oleae* SIT is identification of genes involved in early development which could be targeted for sex-specific embryo lethality or reduce fitness of the offspring of male insects. Early embryo development also presents an opportunity to elucidate the complex mechanisms of embryo development such as the maternal-to-zygotic transition (MZT). MZT occurs in many metazoans. In *Drosophila*, this process starts ~ 1 h after fertilization (AF) and involves the clearance of up at least 35% of transcripts that are maternally deposited in the developing egg^11^ followed by activation of the zygotic genome (reviewed in Tadros and Lipshitz^11^, and Langley et al.^12^). These changes are highly dynamic and tightly regulated, thus capturing them could inform environmentally friendly measures aimed at controlling pests. Indeed, many early embryonic genes and their promoter/enhancer regions have been under intensive studies since they are used in pest control approaches involving transgenic embryonic lethality^13,14^.

We generated a long-read based transcriptome assembly of the olive fruit expanding the current known transcriptome by fourfold and identifying 1768 new genes previously missed in the automated NCBI annotation and correct previously mis-annotated genes. We elucidate the early transcript dynamics in the olive fruit fly embryo identifying a new dramatic reorganization of transcripts that is potentially mediated by post-transcriptional modifications of maternally deposited transcripts. We identified *Zelda* as a potential initiator of zygotic genome activation in *B. oleae*, as observed in *Drosophila melanogaster*. We further measured transcript kinetics, which can be linked to biological processes occurring during embryogenesis and provide sets of co-regulatory genes across the different time points that can facilitate our understanding on the sequence of events during early development. A range of data analysis tools currently available for long-read technologies were also explored. We also generated an independent data set from each of the samples using Illumina short-read RNA-seq and used the data to compare the two technologies and verify some of the isoform models.

## Results

### The current *B. oleae* genome assembly and genome annotation

The olive fruit fly genome is diploid, consisting of six pairs of chromosomes which include a pair of heterochromatic sex chromosomes with the male being the heterogametic sex^15^. We recently submitted to NCBI the *B. oleae* genome (GenBank accession GCA_001188975.2) which was annotated using the NCBI Eukaryotic Genome Annotation Pipeline yielding the *Bactrocera oleae* Annotation Release 100^16^. See Supplementary materials for details of the genome and NCBI gene models. We assigned a *D. melanogaster* homologue to 12,494 (95%) out of the 13,198 protein coding genes (Table S1, E-value ≤ 1e−3). Of these, 57% were identified in the UniProtKB/Swiss-Prot database, which comprises high quality manually annotated and nonredundant proteins, while the remaining 43% were identified in the UniProtKB/TrEMBL database which contains high quality computationally annotated and classified proteins.

### De novo genome-guided transcriptome assembly of the olive fruit fly identifies new genes and isoforms

**Figure 1:**
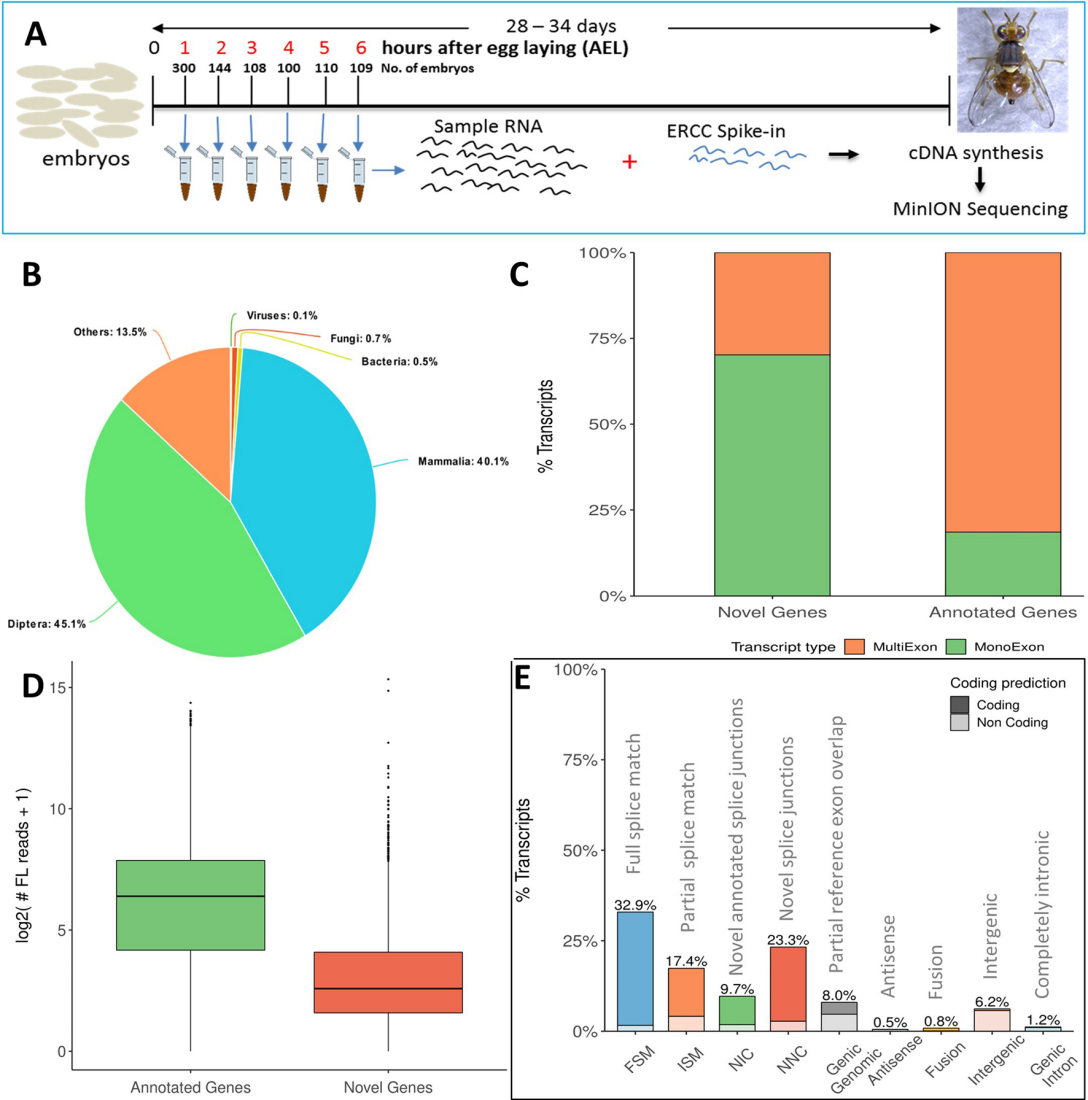
Exploration of the transcriptome assembly. (**A**) Schematic of cDNA library generation and sequencing process. *Bactrocera oleae* embryos were collected at hourly intervals, counted, pooled, and total RNA extracted using the Trizol method. At cDNA synthesis step, external RNA standards (ERCC) were added to each sample commensurate to the number of embryos that were used. The Smart-Seq2 protocol was used to generate full length cDNA, followed by PCR amplification of the cDNA. The Oxford Nanopore Technologies (ONT) SQK-LSK108 protocol for library preparation was then followed, albeit with some custom changes. The library was then sequenced on the ONT MinION, followed by basecalling using ONT Albacore basecaller. After egg laying (AEL) (**B**) Distribution of top Blastp hits of transcripts in the *B. oleae *de novo long-read transcriptome assembly to UniProt Swiss-Prot database. (**C**) Distribution of mono and multi-exon transcripts among previously known genes and novel genes (**D**) Gene expression levels of previous annotated genes and novel genes using long-read counts. (**E**) Distribution of de novo transcriptome assembly transcripts among different structural categories when compared to NCBI predicted gene models. (**C**–**E**) Generated using SQANTI^21^. *FSM* full splice match, *ISM* incomplete splice match, *NIC* novel in catalogue, *NNC* novel not in catalogue (see Table S1 for explanation).

We performed cDNA synthesis for pooled mixed sex *B. oleae* embryos collected at hourly intervals for the first six hours of development using our optimized and customized SMARTer protocol^17^ aimed at capturing poly(A) + RNA (Fig. 1A, Supplementary Figure S1, Supplementary protocol, Supplementary Table S2). Two cDNA libraries separately generated from the heads of adult male and female flies were also included to expand the transcriptome. A total of 31 million reads were generated (see Supplementary Tables S3, S4, and S5 for sequencing and alignment statistics). We evaluated 3 long-read transcriptome assembly tools; TAMA^18^, Cupcake ToFU^19^, and TAPIS^20^, and selected Cupcake ToFU due to its computational efficiency, high sensitivity and precision (Supplementary Materials). Our comparison of short-read and long-read transcriptome assemblies showed that ONT long-read assembly recovered a higher number of isoforms (43,676 versus 21,840, respectively) and had a higher sensitivity at exon level (Supplementary materials).

To derive the final transcriptome assembly, 3.9 million reads among all error corrected reads that were aligned at least 99% in length and with at least 95% identity were used. The de novo assembled transcriptome was analyzed using SQANTI^21^ and PRAPI^22^. The transcriptome assembly contained a total of 10,840 genes of which 9072 genes matched the NCBI annotated genes, while 1768 genes were new, hereafter referred to as novel genes (Supplementary Table S6). Among novel genes, we selected 454 high-confidence genes that had support from 5 or more long-read transcripts and transcripts per million (TPM) of 0.2 or more as determined from Illumina short-read RNA-seq (Supplementary Table S7). The total isoforms identified were 78,018 expanding the olive fruit fly transcriptome by four-fold over the current NCBI annotation. A Blast search of the predicted protein sequences among all transcripts in the long-read transcriptome assembly against UniProt Swiss-Prot database showed that the top hits were of the order Diptera (45.1%, Fig. 1B), followed by class Mammalia (40.1%). Further analysis revealed that 99% of the hits to Mammalia also had hits to Diptera. Hits to Viruses, Fungi, and Bacteria accounted for only 1.3%.

Surprisingly, although 1768 novel genes were identified, only 242 of them were predicted to contain open reading frames suggesting that most of the novel genes were noncoding genes. Further, 70% of novel genes were mono-exon compared to only 19% among NCBI annotated genes (Fig. 1C). Novel genes also showed lower expression compared to annotated genes (Fig. 1D). This suggested that genes that are non-coding, of low expression, and/or are mono exon might be more likely to be missed in computational gene prediction pipelines. Structural comparison showed that most of the transcripts in the long-read de novo transcriptome assembly were a full splice junction match (FSM, 32.9%) to the NCBI predicted transcripts, showing the utility of the widely used NCBI prediction models in capturing majority of genes. This was followed by transcripts with incomplete splice junction matches (ISM, 23.3%, Supplementary Table S6, Fig. 1E). We further used PRAPI and identified 63 genes that were miss-annotated as two or more separate genes but we could find single transcript reads covering these genes (Supplementary Table S8, Supplementary Figure S2). A more detailed exploration of the long-read derived transcriptome is shown in Supplementary material. Additional files 1–S5 provide the expression matrix and additional information on novel genes.

### Direct absolute normalization of RNA-seq data outperforms relative normalization

**Figure 2:**
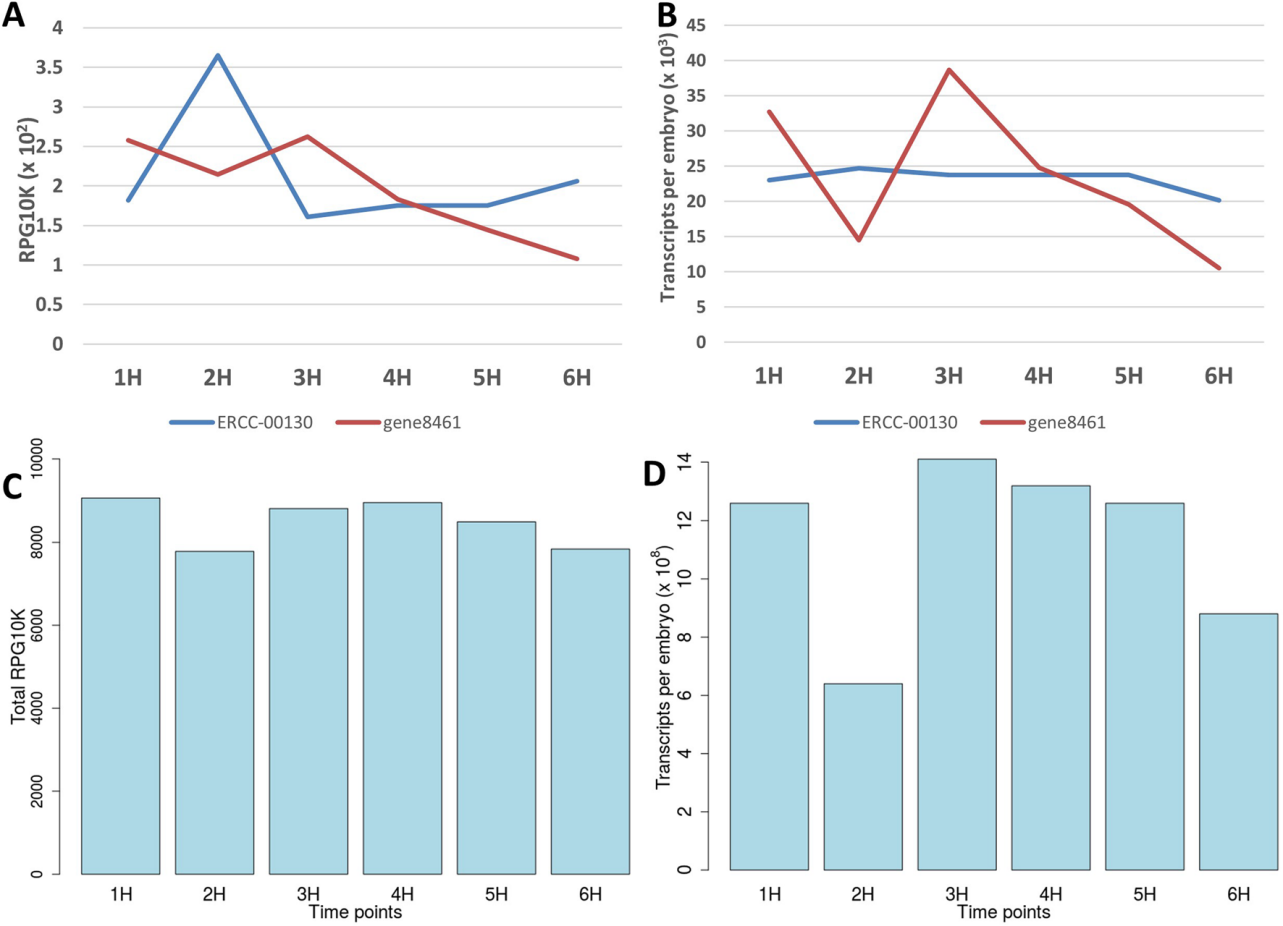
Comparison of relative and absolute normalization. (**A**) Relative gene expression quantification of ERCC internal spike-in control 00130 (blue) and gene8461 (red) as obtained with Mandalorion software^4^. For each gene Mandalorion reports its quantification as reads per gene per 10,000 mapped reads (RPG10K). (**B**) Same as (**A**) but showing number of transcripts per embryo following absolute normalization (see “Methods”). (**C**) Summed RPG10K for all genes at each timepoint. (**D**) Same as (**C**) but showing total number of transcripts per embryo across the six timepoints following absolute normalization.

We used the absolute normalization method as previously described by Owens et al.^8^ (also see Supplementary materials). In contrast to the relative expression (Fig. 2A), absolute normalization showed more constant abundance of internal ERCC standards across timepoints (Fig. 2B, Supplementary Figure S3). Unlike the summed relative gene expression levels (Fig. 2C), the summed absolute number of transcripts per embryo for all genes (Fig. 2D) mirrored that of cDNA generated per embryo (Supplementary Figure S4A), thus validating the absolute normalization approach. Further, as we had anticipated, the successive timepoints showed higher gene expression correlation than distant timepoints with Spearman correlation for successive samples consistently equal or above 0.96 (Supplementary Figure S5). This suggests that our sampling was close enough to capture transcriptional dynamics across the sampling time. We also calculated the lower limit of detection setting our sensitivity to RPG10K of 0.01 which corresponds to ~ 2 mapped reads. Averaged over the 6 samples, the detection limit was 1038 transcripts per embryo (Supplementary Table S9). Principal component analysis (PCA) and hierarchical clustering using the most variable genes not only recapitulated the developmental trajectory but also suggested close similarity in gene expression patterns between *B. oleae* and *D. melanogaster* early embryos (Supplementary Figures S6, S7).

### Total mRNA content of the embryo and biological replication validate absolute quantification

**Figure 3:**
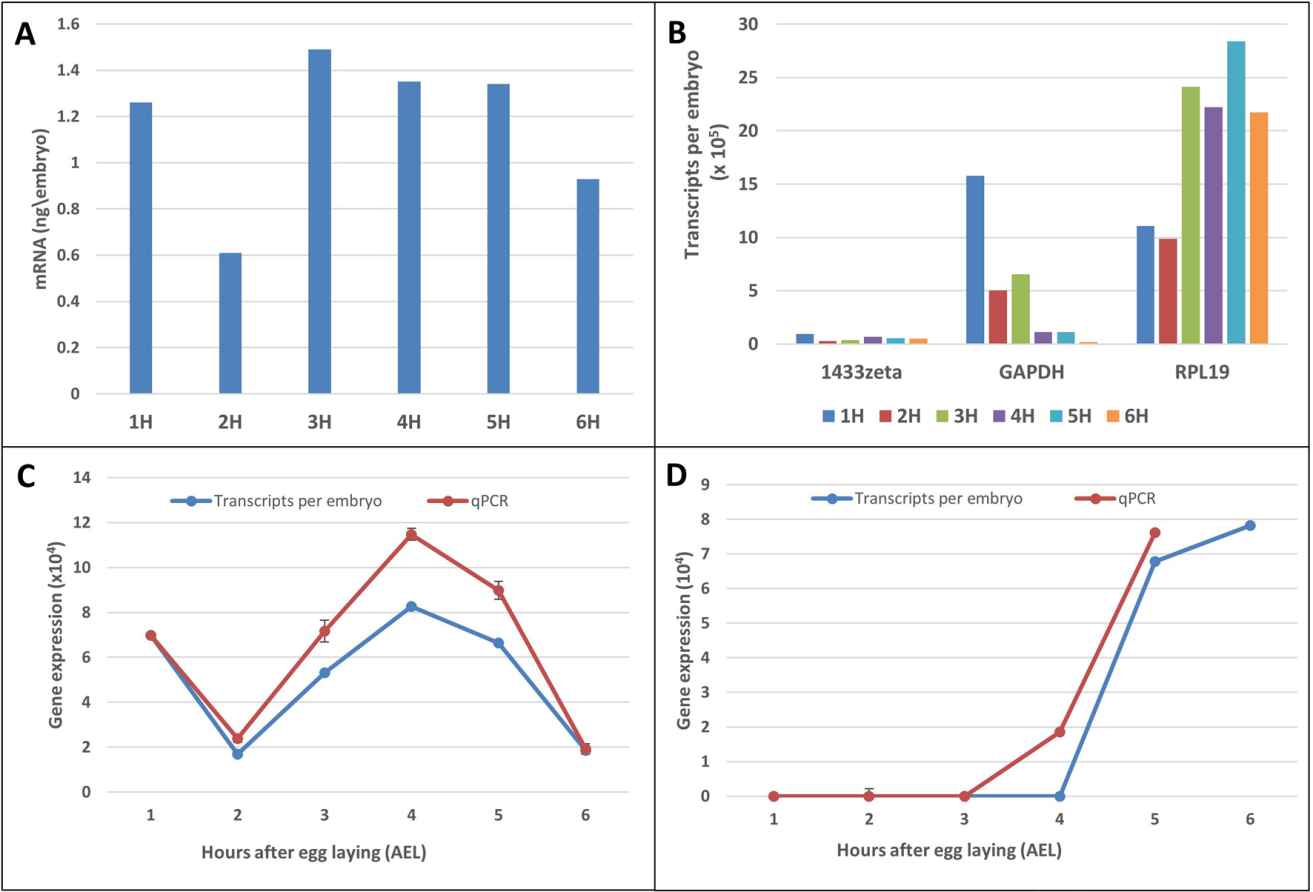
Validation of absolute normalization. (**A**) Total mRNA (ng) per embryo across timepoints derived from conversion of absolute number of transcripts per gene to nanograms. (**B**) Absolute gene expression of 3 genes proposed by Sagri et al.^30^ as candidate housekeeping genes (*14.3.3.zeta*, *GAPDH*, *RPL19*). (**C**) Absolute expression (blue) and qPCR expression (red) of serendipity alpha (*sry*). qPCR expression values were scaled to compare expression profiles. (**D**) Same as C but for head involution defective (*hid*). The reverse transcription step of the qPCR used a mixture of oligo(dT) and random hexamers. The 6-h timepoint for this qPCR assay is skipped due to technical limitations. Standard error of the mean of two biological replicates is depicted in bars.

We computed the total mRNA per embryo by summing all transcripts per gene per embryo and calculating the equivalent in nanograms (Fig. 3A). The total mRNA dropped from 1.26 ng/embryo at 1 h AEL to 0.61 ng/embryo at 2 h AEL and then rebounded to 1.49 ng/embryo at 3 h AEL before dropping to 0.93 ng/embryo at 6 h AEL when we ended our sampling. The pattern mirrors the total transcripts per embryo (Fig. 2D). The mRNA levels agree with the empirically determined total RNA yields we obtained per embryo (~ 33 ng/embryo at 1 h AEL and 53 ng/embryo at 6 h AEL, Supplementary Table S2) assuming 2–5% of total RNA is polyadenylated^23^. We also compared the volume of *B. oleae* embryos to *Xenopus tropicalis* (0.025 mm^3^ versus 0.268 mm^3^, respectively, giving a volume ratio of 10.7). *X. tropicalis* embryos contain 10–15 ng of mRNA/embryo at fertilization^8^ which closely matches the *B. oleae* 13.5 ng mRNA/embryo at 1 h AEL, after accounting for the volume ratio. Cell volume and RNA content are correlated such that larger cells have more RNA content^8,24^. However, our results differ from results of *D. melanogaster.* The volume of *D. melanogaster* eggs at oviposition is 0.012 mm^3^^25,26^ and contains 140–210 ng/embryo of total RNA across all stages^23,27,28^ of which ~ 2% is poly(A) (~ 3.8 ng)^23^.

Real-time quantitative PCR (qPCR) provided further validation. First, we determined that as previously reported *14-3-3zeta* and ribosomal protein L19 (*RPL19*) had low variability in expression and could be used as reference genes for qPCR^29,30^ (Fig. 3B, Supplementary Figure S8). qPCR expression of 2 genes; serendipity alpha (*sry-a*) and head involution defective (*hid*) in a different set of biological replicate samples using *RPL19* and *14-3-3zeta* as reference genes showed similar trends of gene expression with our absolute quantified expression (particularly with RPL 19, Fig. 3C,D compared to *14-3-3zeta*, Supplementary Figure S9). We provide another 10 genes from our embryo dataset that had the lowest standard deviation in their row z-score suggesting that these genes had the lowest variability in their abundance across our samples (Supplementary Table S10). These could be used as qPCR reference genes in early embryo development.

### Dramatic reorganization of maternal transcripts in *B. oleae* embryos

In many organisms, oocyte development is arrested during meiosis followed by deposition, into the oocyte, of maternally derived transcripts that represent majority of protein coding genes. Among the 13,198 *B. oleae* protein coding genes, 62% (8132) were detectable at 1 h AEL (detection limit of 1110 transcripts per embryo). By far, the most abundant transcript in the 1-h embryos was the mitochondrial encoded 16S ribosomal RNA which accounted for ~ 2.5% of the total embryo mRNA content. We observed an interesting phenomenon when examining the total mRNA content per embryo during development across timepoints. The total mRNA per embryo, as measured by molecular conversion of transcripts to mass, dropped 51% at 2 h AEL compared to levels at 1-h AEL and increased 143% at 3 h AEL compared to levels at 2 h AEL (Fig. 3A). This profile mirrored that of cDNA generated; although our cDNA synthesis protocol used equal amounts of total RNA per time point (300 ng), the amount of cDNA generated at 2 h AEL was 2.3 times less than that derived at 1-h AEL after adjusting for number of embryos used (Supplementary Figure S4A). Further, the total RNA profile showed comparable quality among our samples, ruling out RNA degradation artifacts (see Extended Fig. 2 in supplementary material). Indeed, the dramatic drop in abundance of poly(A) transcripts observed at 2 h AEL (Fig. 3A) could be replicated in a different set of biological samples using qPCR which used a mixture of both oligo(dT) primers and random hexamers in the reverse transcription step (Fig. 3C, Supplementary Figure S9). We show in Supplementary Figures S10B,C, S11, and Supplementary materials that the dramatic downregulation of polyadenylated maternal transcripts seen at 2 h AEL and the rebound seen at 3 h AEL is systemic rather than targeted and might act as a “normalization” process that mainly affects the most highly abundant genes, to bring their levels down to basal levels of other genes.

Following this ‘normalization’ process, we observed a gradual reduction in the abundance of polyadenylated transcripts starting at 4 h AEL up to 6 h AEL. This most likely reflects the beginning of the clearance of maternal transcripts signifying the initiation of the maternal-to-zygotic transition (MZT). MZT involves two main stages: the clearance of a large proportion of maternal transcripts and proteins originally deposited into the oocyte during oogenesis, and the initiation of zygotic transcription^11^. Compared to *D. melanogaster* embryos that contain ~ 3 × 10^9^ copies of maternal poly(A) transcripts^31^ during early development, *B. oleae* embryos contained 1.5 × 10^9^ poly(A) RNA transcripts at 1 h AEL. In Drosophila, MZT involves destabilization of up to 20% of maternally supplied transcripts by maternally encoded proteins by the end of 2 h after fertilization (AF) and another 15% of maternal transcripts are destabilized by zygotically encoded protein by 3 h AF^11^. In *B. oleae*, the number of transcripts per embryo dropped from 1.7 × 10^9^ at 3 h AEL to 1.1 × 10^9^ at 6 h AEL, a 36% drop, after adjusting for zygotic genes.

### Early zygotic genes and genes involved in early embryo development

In *D. melanogaster*, zygotic genome activation, which depends on absolute time or developmental stage, occurs in two waves starting with a minor wave about 1 h after fertilization followed by a major wave about 2 h after fertilization^11^. The promoters of genes that are expressed earliest during drosophilid embryo development are enriched with TAGteam sites^32^ which is principally composed of the motif CAGGTAG. These TAGteam sites play a role in timing of transcription of early zygotic genes in *Drosophila*^32^. The zinc finger transcription factor *Zelda* (*zld*) which is maternally supplied and maintained all through embryo development binds the TAGsites and is a general activator of hundreds of genes during and after MTZ including early sex-related genes such as *sisterless A* (*sisA*), *scute* (*sc*), *sex lethal* (*Sxl*), *deadpan* (*dpn*) and cellular blastoderm formation genes such as ‘slow as molasses’ (*slam*) and *serendipity* (*sry-α*)^33^.

**Figure 4:**
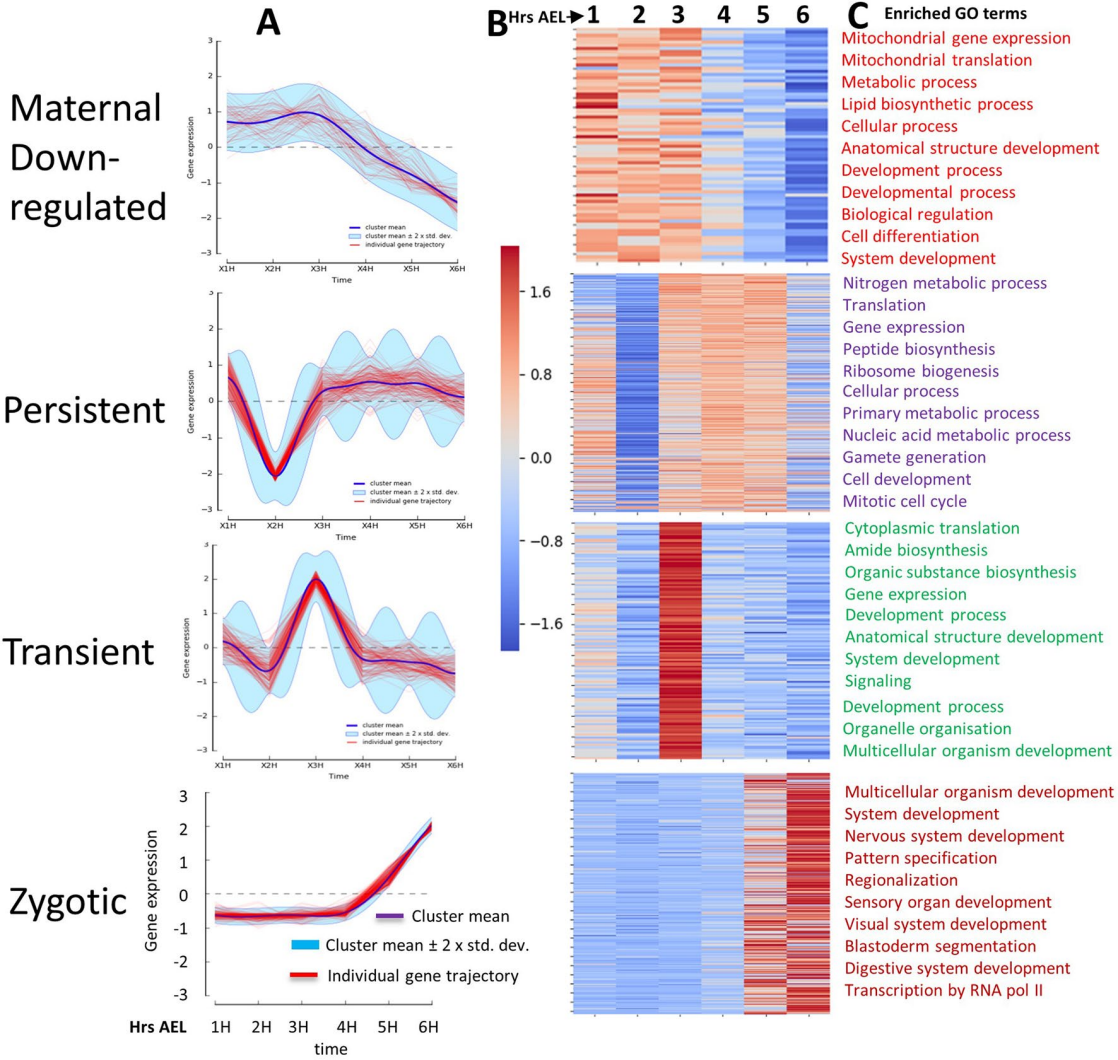
Temporal synexpression and gene ontology enrichment analysis of *Bactrocera oleae* genes at different timepoints after egg laying (AEL). Drichlet process Gaussian process clustering^48^ was used to cluster genes according to their temporal expression. Out of the 87 clusters obtained 4 groups of genes were identified; Maternal, Persistent, Transient, and Zygotic. (**A**) Representative profiles for the different groups. (**B**) Heatmap of gene expression for each gene group. (**C**) Top enriched Gene ontology (GO) terms among the corresponding gene groups. Homologes of *B. oleae* were identified through a Blastp search against UniProtKB/Swiss-Prot *D. melanogaster* database (Evalue 1e−3). Enriched GO terms were identified using gProfiler with 5% false discovery rate.

Using the GFOLD^34^ method we identified 159 differentially expressed genes. Because these genes were not detectable at 0–1 or 1–2 h AEL but detectable at any of the other time points suggesting that their transcription was from the zygotic genome, they should be enriched in zygotic genes (here referred to as zygotic-early genes, Supplementary Table S11). This list of genes includes some well-known *D. melanogaster* early zygotic genes such as *sisA*, *sna*, *kni*, *nullo*, *Kr*, *dpp*, *gt*, *odd*, and *nrt*. We also identified *zld* and found that, indeed, *zld* was expressed throughout our sampling time which led us to hypothesize that *zld* plays a similar role in both *B. oleae* and *Drosophila*. We used the DREME tool of MEME suite^35,36^ to identify differentially enriched motifs in promoter sequences (1000 bp upstream of transcription start sites, TSS) between zygotic-early genes and genes that are maternally supplied but downregulated and have no evidence of being transcribed from the zygote (Fig. 4A). The motif CAGGTAB was by far the most enriched (E-value 4.7e−12, Supplementary Figure S12 A,B). Of the 159 high confident early zygotic genes used, this motif was most enriched in 300 bp upstream of the TSS of 83 genes, as determined using CentriMo tool^37^ (Supplementary Figure S13). This agrees with the same finding in *Drosophila* where TAGteam sites were enriched in 500 bp upstream of TSS of early zygotic genes^32^. Gene ontology enrichment analysis^38,39^ among the 83 genes with TAGteam sites located 300 upstream of TSS showed that these genes were enriched in key developmental processes such as Malpighian tubule development, renal tubule development, hindgut morphogenesis, digestive tract morphogenesis, blastoderm segmentation. The remaining 76 zygotic-early genes that had no TAGteam sites enrichment in the 300 bp upstream of TSS had no statistically significant enriched processes. Gabrieli et al.^40^ reported lack of TAGsites in *Ceratitis capitata,* a closely related species to *B. oleae*. However, this was based on a single gene, *sry-α*, which is maternally supplied in *B. oleae* according to our analysis (see Additional file 9). Our data suggests that *Zelda* could play a similar function in activation of early zygotic genes in *B. oleae* as it does in drosophilids^33^.

It has been observed across different species that early zygotic genes are shorter (which would be transcribed quickest) and contain fewer introns (which would require less processing) compared to the rest of the genes^41–43^. Indeed, we observed that our set of early zygotic genes were shorter and contained fewer exons than other genes (Wilcoxon test p value 5.1e−7, Supplementary Figures S14, S15). The transcript lengths of the maternally supplied and early zygotic genes were comparable (Wilcoxon test p value 0.8, Supplementary Figure S15) indicating that genes with fewer or no introns were selected as early zygotic genes. Further, the few zygotic genes with introns showed higher rates of intron containing transcripts than maternal genes suggesting nascence of transcription and/or faster rates of transcription than processing (Supplementary Figure S16). This phenomenon has been previously observed in *Drosophila* embryos^44^ and has been previously used to identify zygotic genes in zebrafish^45^. These data strongly suggest that these genes were indeed emanating from the zygotic genome and not maternally supplied. Such genes could be potential targets for insect sterile techniques and other avenues for environment-friendly insect control strategies.

### Clustering of genes based on temporal expression dynamics

In developing embryos, regulation of spatio-temporal dynamics in gene expression is critical for proper organ development. Clustering of genes based on their temporal expression dynamics enables identification of genes with similar biological function^46,47^. Temporal gene expressions have been suggested to follow Gaussian distribution^8^. We thus clustered our data using DPGP^48^ which jointly models data clusters with a Dirichlet process and temporal dependencies with Gaussian processes. Indeed, we identified gene clusters with differing kinetics suggesting specific roles for these genes during defined developmental periods (Fig. 4A, Supplementary material). We further grouped the clusters into 4 groups: (1) maternal-downregulated: comprising genes that were highly expressed at 1 h AEL and were destabilized at 2 h AEL and generally decreased over time. (2) Persistent: genes whose expression was maintained through 3–5 h AEL. (3) Transient: genes whose expression peaked only at specific timepoints. And (4) zygotic: genes whose expression was only detectable starting from 4 h AEL implying that they emanated from zygotic genome. Genes in the different categories were combined, a heatmap generated (Fig. 4B, showing a representative cluster for each category) and gene ontology (GO) enrichment analysis performed (Fig. 4C). Maternal-downregulated genes, which were also among the most highly expressed at 1 h AEL were enriched in mitochondrial gene expression, metabolic processes, lipid biosynthesis processes, cellular processes, development among other processes (Fig. 4C). Persistent genes were enriched in nitrogen metabolic process, translation, gene expression, ribosome biogenesis among others which reflects the high metabolic activity of the rapidly growing embryo. Transient genes showed enrichment of developmental processes, anatomical structure development, system development, signaling, among other processes which implicated these genes in key developmental processes. Strikingly, Zygotic genes were enriched in specific and key tissue formation and developing processes including; multicellular organism development, system development, nervous system development, pattern specification, sensory organ development, hindgut development, digestive tract morphogenesis, and thus showing that we captured genes required for organ formation in this organism (Fig. 4C).

### Long-read RNA-seq improves annotation of genes in the sex determination pathway

**Figure 5:**
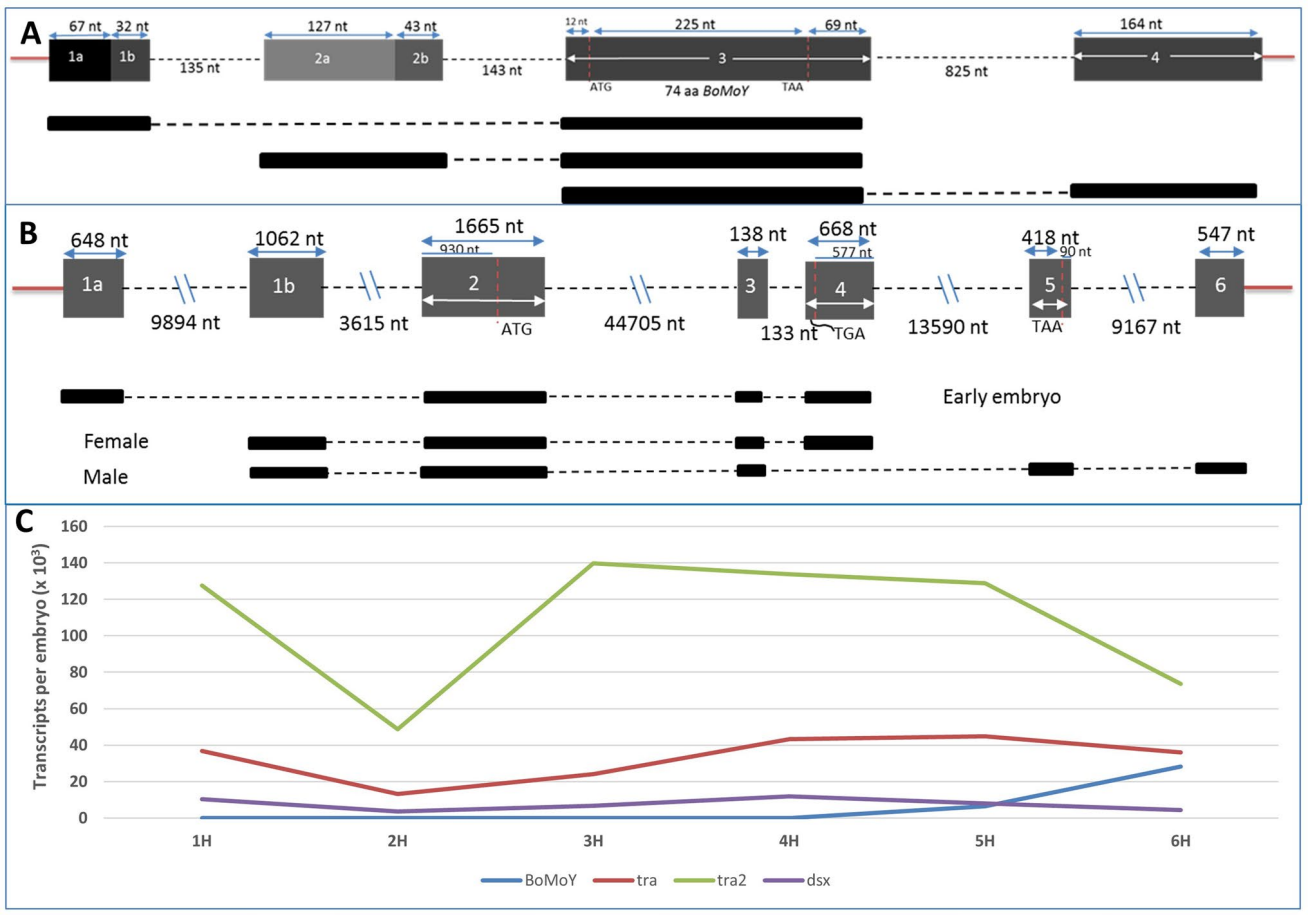
Schematic of the gene structure and expression profile of *B. oleae* sex-determining genes. (**A**) *Bactrocera oleae* maleness-on-Y (*BoMoY*) gene model and the 3 isoforms observed. (**B**) *Double sex* (*dsx*) gene model and the prominent isoforms observed in early embryo development as well as adult male and female. (**C**) Expression profile of *BoMoY*, *transformer* (*tra*), *transformer2* (*tra2*), and *doublesex* (*dsx*) shown as number of transcripts per embryo. The expression was calculated for total gene expression rather than sex-specific isoforms for *tra* and *dsx* which were below quantification range.

The sex determination molecular cascade in *B. oleae* includes 4 major proteins: maleness-on-the-Y (*BoMoY*), transformer (*tra*), transformer 2 (*tra2*), and doublesex (*dsx*). Although different models have been previously reported for these genes^49^, we provide new models based on our long-read RNA-seq which better elucidates the gene structures. *BoMoY* spans 1842 bp genomic region containing 4 exons while the transcript spans 739 bp. Exon 3, which is also the largest, encodes a predicted 74 amino acid protein (Fig. 5A). Just like the *Ceratitis capitata* maleness-on-the-Y (*CcMoY*)^50^, the first expression of *BoMoY* was detected at 5 h AEL (Fig. 5C). *B. oleae dsx* has 6 exons (Fig. 5B). Exon 4 is female-specific and a potential target for CRISPR/Cas9 editing. Supplementary Figures S17–S25 and Supplementary materials have details of the models and PCR validation of some isoforms.

## Discussion

Early embryo development is an ideal period for transcriptomic studies. In *Bactrocera spp* the mechanism of sex determination, which is mediated by alternative splicing of genes, is initiated during this period^51^. Further, evidence from Mediterranean fruit fly (*C. capitata*)^52,53^, olive fruit fly (*B. oleae*)^54^, and Queensland fruit fly (*B. tyroni*)^55^ shows that in Tephritidae flies, pole cells, the primordial germ cells, are established during this period, typically 3–4 h following oviposition. Pole cells are critical targets for successful horizontally transferable genetic manipulations because successful creation of transgenic flies capable of passing on the mutation requires injection of syncytial preblastoderm embryos before the establishment of pole cells, typically 20 min to 2 h following oviposition^56–58^. Genes active during this period and/or their promoters and enhancers could be potential targets for generation of sterile strains or development of molecular sexing strains, among other sterile insect technique applications for pest control. However, lack of well annotated genomes in important non-model organisms makes identification of such genes difficult.

We used long-read RNA-seq in the olive fruit fly and generated a de novo transcriptome assembly which identified 1768 new genes. The current *B. oleae* NCBI estimation of 13,936 genes is comparable to those of the closely related *Ceratitis capitata* (14,652^59^). The *C. capitata* genes were however, computationally predicted and thus could face similar limitations as the *B. oleae* predicted genes. The well-studied *D. melanogaster* contains ~ 17,000 genes. We thus argue that the current *B. oleae* number of genes is underestimated, and our transcriptome provides 1768 novel genes bringing the total number of known genes to 15,704 towards completing the annotation of the genome. Pavlidi et al.^60^ previously generated a transcriptome assembly of the olive fruit fly and assembled a total of 14,204 contigs. However, they used a short-read sequencing technology (454 pyrosequencing) and the average contig length was 421 bp compared to an average of 9000 bp in our transcriptome and 9597 in the NCBI predicted gene models.

Calibration of gene expression quantification using internal standards enabled absolute quantification in terms of the number of transcripts per gene per embryo, using ERCC standards. Compared to relative normalization, absolute normalization enabled better recapitulation of the mRNA content per embryo across timepoints thus showing the utility of ERCC molecular internal standards to capture the dramatic transcription changes in highly dynamic systems like developing embryos. Further, there was remarkable similarity between *B. oleae* and *X. tropicalis* total mRNA content after accounting for embryo volume differences providing further validation of our quantification. Owens et al., reported a similar finding in *Xenopus* when they compared *X. tropicalis* and *X. levis* embryos which have a volume ratio of 3.31^8^. Although *B. oleae* is more closely related to *D. melanogaster,* our estimation of mRNA content in *B. oleae* was different to the reported mRNA content in *D. melanogaster*. *B. oleae* embryos at 0.02 mm^3^ are twice the volume *D. melanogaster* embryos yet *B. oleae* embryos contain less than half the mRNA (1.2 versus 3.8 ng/embryo, respectively). It is likely that the very fast rate of embryo development in *Drosophila* (~ 3 times that of olive fruit fly) necessitates a much higher number of maternal transcripts which might explain the higher amount of mRNA.

We determined that among the 13,198 NCBI predicted *B. oleae* protein coding genes, 62% (8132) were detectable at 1 h AEL (detection limit of 1110 transcripts per embryo). Since the zygotic genome is generally not yet activated during this period, most of these genes can be assumed to be maternally deposited in the embryo. These results are similar to other organisms. For example, maternal transcripts represent about 75% of the total protein-coding genes in *Drosophila melanogaster*^42^ and *Danio relio* (zebrafish)^61^. The enrichment of maternal transcripts with genes involved in translation, cellular processes, metabolic processes, and transcription factors like DBREF, agrees with the embryo’s dependency on this rich medium for the first 2 h of development where rapid cell division and expansion occurs^11^. We show that in *B. oleae*, there is a reorganization of maternal transcripts characterized by a dramatic reduction in global poly(A) content of embryos. This phenomenon likely happens in other non-drosophilid insects. It could have been missed in other insects because many studies used low resolution sampling since they combine embryos at different hours, for example 0–2, 2–4 as was the case for the mosquito *Ae. aegypti*^62^. The presumed lack of zygotic transcription in the first 3 h of embryo development suggests that the ‘normalization’ event we observed is mediated by de-adenylating and polyadenylating mechanisms. In *Xenopus* (frog), high resolution sampling (every 30 min) combined with poly(A) and ribosomal depletion RNA-seq (rdRNA-seq) showed evidence of poly-adenylation of maternal transcripts^8^. Although this profile resembles the pattern we observed for *B. oleae*, lack of a ribosomal depleted dataset prevented us from differentiating polyadenylation, de-adenylation, and degradation events.

In most organisms studied, a portion of the maternal transcripts and proteins are degraded through a key developmental process known as maternal-to-zygotic transition (MZT)^63^. We used our time-course data to elucidate the mechanism of MZT, a process that has not been studied before in *B. oleae*. Our sampling captures the initial events of this period as it begins at oviposition (1 AEL) up to the beginning of blastoderm cellularization (6 AEL) when pole cells are already established at the posterior end of the embryo^54^. Starting at 4 h AEL up to the end of our sampling at 6 h AEL, we observed a gradual decrease in mRNA content per embryo. Since the *B. oleae* embryos develop about 3 times slower than *Drosophila* embryos and whereas MZT in *Drosophila* starts ~ 1 h AEL, this gradual decrease is consistent with the degradation of maternally supplied transcript and factors in the developing embryo. Accompanied with this, we started to notice the transcription of zygotic genes such as male sex determining gene, *BoMoY*, that is transcribed from Y chromosome. These zygotic genes were enriched in developmental processes. However, due to our short sampling time we could not resolve the whole MZT period. It seems likely from the expression profile that the *B. oleae* embryos continue this gradual degradation of maternal genes all through the first 8 h of development. The olive fly genome size is ~ 3 times larger than the *D. melanogaster* genome and incidentally the olive fruit fly embryo develops at ~ 3 time slower than *D. melanogaster*. Since the *Drosophila* MZT ends before the cellular blastoderm stage (~ 2.5 h AEL^11^) the olive fly MZT might not end until ~ 7 to 9 h AEL when the cellular blastoderm starts to form. Proper cellular blastoderm development of *D. melanogaster* has been suggested to depend highly on zygotic genes^64^. We identified genes that are potentially among the first zygotic genes to be transcribed in the olive fruit fly. We also identified *Zelda* as a potential regulator of zygotic genome activation in the olive fly embryo. Maternal-only transcripts lacked *Zelda* bind motif. It has previously been observed that maternal and zygotic transcripts have different regulatory mechanisms^65^. These zygotic genes are potential targets for sterile insect techniques.

A limitation of the study is the lack of extensive biological replication in form of RNA-seq, due to the limited availability of biological material. An independent sample set analyzed using the same methodology would have allowed the introduction of further statistical tests to support significant expression differences.

Another limitation of our approach is that it is uses a poly(A) tail (≥ 30 bases) for reverse transcription and cannot directly investigate RNA degradation. However, removal of the poly(A) tail should lead to degradation of the respective RNA. Indeed, in *Drosophila* RNA binding proteins that play a key role in MZT, such as *Smaug, BRAT*, and *PUM,* recruit the CCR4-NOT-deadenylase complex to remove the poly(A) tail and mediate the degradation of target genes^63,66–68^. The known exceptions of, for example, histone genes that lack poly(A) tail is noteworthy^69^. Further, our approach cannot differentiate between genes directly transcribed from zygotic genome and maternal transcripts which undergo post-transcriptional modification (for example polyadenylation) and thus making them appear in our quantification. Indeed, in *Drosophila*, the cytoplasmic poly(A) polymerase encoded by *wispy* promotes poly(A)-tail lengthening during both oocyte maturation and egg activation^70–72^. We, however, use the intron signal to infer zygotic genes (Supplementary Figure S16), as previously applied to identify zygotic genes in zebrafish^45^. Nevertheless, expression profiling methods that do not rely on oligo dT priming such as total RNA sequencing of ribosomal depleted samples, or genetic manipulations, as described, for example, by De Renzis et al.^42^ who used chromosomal deletion, would need to be performed to identify purely zygotic genes and purely maternal genes in *B. oleae*.

## Methods

### Olive fruit fly breeding

The olive fruit fly (*Bactrocera oleae*) ‘Demokritos’ strain, that is considered in this study, is maintained in our laboratory for over 15 years and was originally sourced from the Nuclear Research Centre in Athens, Greece. No wild flies have been added since then, hence the strain has maintained a genetic uniformity. Olive fruit flies were reared in appropriate holding cages at 25 ± 1 °C, 60 ± 10% relative humidity and 14 L: 10D cycles as previously described^73^.

### Embryo collection, RNA extraction and quality control

Olive fruit flies from the Demokritos line, were mated with males and then monitored to observe egg laying. Once the eggs were laid, the eggs were incubated at room temperature for 1, 2, 3, 4, 5, or 6 h, respectively followed by RNA extraction using the Trizol method. We also included RNA from adult heads to increase number of genes in the transcriptome assembly. The quantity of the extracted RNA was determined using a Qubit RNA HS Assay Kit (Thermo Fischer Scientific, Q32852). The quality of the isolated RNA was assessed using an Agilent TapeStation instrument and Agilent RNA ScreenTape kit as per manufacturer’s instruction.

### ERCC spike-in addition and cDNA synthesis

For each timepoint, 300 ng of total RNA was used for the cDNA synthesis protocol. ERCC Spike-in Mix 1 (Thermo Fischer Scientific, 4456740) were added to the cDNA synthesis master mix at a rate commensurate with the number of embryos used (see Supplementary protocol). Our customized and published cDNA synthesis protocol^17^ is based on the highly sensitive Smart-seq2 protocol^74^. cDNA sequencing was performed on the ONT MinION sequencer using SQK-LSK108 kit and R9.4 flow cells. A step-by-step detail protocol of the cDNA library synthesis and sequencing is provided as Supplementary protocol, Additional file 6. We also performed Illumina short-read RNA-seq to collaborate findings such as correction of errors in the long-read data, identification of splice junctions, and gene expression quantification (see Supplementary materials). However, all the present work relates to the Nanopore long-read RNA-seq unless otherwise stated.

### Genome guided de novo transcriptome assembly

We developed a de novo transcriptome assembly pipeline shown in Supplementary Figure S1. We used Cupcake ToFU^19^ for transcriptome assembly because of its adequate user options and reasonable running speed. ToFU was used to collapse the transcripts into a non-redundant set of transcripts comprising the genes and their associated isoforms. SQANTI^21^ was then used to analyze the transcripts, identify novel genes, and perform open reading frame prediction using the GeneMarkS algorithm.

### Read alignment

The NCBI *B. oleae* assembly (accession code GCA_001188975.2) was used for the alignment. However, since we included ERCC in our cDNA the ERCC sequences were included in the NCBI assembly prior to alignment. Alignment of reads to the reference genome and transcriptome was performed using 2 splice-aware and long-read enabled aligners; GMAP^75^ and Minimap2^76^.

### Relative quantification of gene expression

Relative quantification of gene expression was performed using a customized version of Mandalorion pipeline^77^. The *Bactrocera oleae* Annotation release 100 from NCBI, updated with novel genes identified in this study, and the *B. oleae* assembly (accession code GCA_001188975.2) were used as reference files. Mandalorion counts the number of reads overlapping exon features of a gene and normalizes for sequencing depth and calculates the relative abundance as Reads Per Gene per 10,000 aligned reads (RPG10K).

### Direct absolute normalization of gene expression

Absolute gene expression was computed using the method previously reported by Owens et al.^8^. The method relies on the use of known transcript copy numbers for each ERCC standard and their corresponding relative expressions to derive a conversion factor. The conversion factor is derived from a generalized linear model with a dispersed Poisson likelihood using R statistical software^78^ as follows;

$${\text{glm}}\left( {{\text{formula}} = {\text{r}}_{{{\text{qj}}}} \sim {\text{offset}}\left( {{\text{log}}\left( {{\text{Sq}}} \right)} \right),{\text{family}} = {\text{poisson}}\left( {{\text{link}} = {\text{log}}} \right)} \right)$$

where r_qj_ is the relative abundance (RPG10K) of standard q in sample j, Sq is the known abundance (number of molecules /transcripts) of standard q.

The intercept coefficient from the above function is the conversion factor used to convert RPG10K to absolute quantification using the following formula;

$$m_{ij} = \rho_{ij} {\text{e}^{-}}^{\beta j}$$

where m_ij_ is the absolute abundance (number of molecules /transcripts) for gene i in sample j, ρ_*ij*_ is the relative abundance (RPG10K) of gene i in sample j, β_j_ is the conversion factor.

### Temporal clustering of developmental stage-specific genes

We used Dirichlet process Gaussian process (DPGP^48^) to cluster genes based on their temporal co-expression. The expression matrix was used as input to cluster genes with similar expression profiles. The resulting clusters were divided into 4 categories: maternal-downregulated, persistent, transient, zygotic. Genes in clusters that corresponded to each category were combined and used in gene ontology enrichment analysis using gProfiler^79^.

## Supplementary Information

Supplementary Information 1.

Supplementary Information 2.

Supplementary Information 3.

Supplementary Information 4.

Supplementary Information 5.

Supplementary Information 6.

Supplementary Information 7.

Supplementary Information 8.

## Abbreviations

SIT: Sterile insect technique
ONT: Oxford nanopore technologies
TGS: Third generation sequencing
PacBio: Pacific bioscience
kb: Kilobase(s)
Mb: Megabase(s)
Gb: Gigabases
ng: Nanogram(s)
NCBI: National Center of Biotechnology Information
PCR: Polymerase chain reaction
vs: Versus
rpm: Rounds per million
ml: Milliliter
µg: Microgram
mM: Millimolar
EDTA: Ethylene di-amine tetra-acetic acid
nM: Nanomolar
pM: Picomolar
µl: Microlitre
HMW: High molecular weight
