# Supplementary material for: Nanopore long-read RNA-seq and absolute quantification delineate transcription dynamics in early embryo development of an insect pest
Source: Sci Rep. 2021 Apr 12;11:7878. doi: 10.1038/s41598-021-86753-7 (PMC8042104; doi:10.1038/s41598-021-86753-7)
Supplement: Supplementary file 6 — Supplementary Information 6. [file 41598_2021_86753_MOESM6_ESM.docx]

**Nanopore long-read RNA-seq and absolute quantification delineate transcription dynamics in early embryo development of an insect pest**

Anthony Bayega^1^*, Spyros Oikonomopoulos^1^, Maria-Eleni Gregoriou^2^, Konstantina T Tsoumani^2^, Antonis Giakountis^2^, Yu Chang Wang^1^, Kostas D Mathiopoulos^2#^, Jiannis Ragoussis^1,3#^

1. McGill Genome Centre, Department of Human Genetics, McGill University, Montréal, Québec, Canada

2. Laboratory of Molecular Biology and Genomics, Department of Biochemistry & Biotechnology, University of Thessaly, Larissa, Greece

3. Department of Bioengineering, McGill University, Montréal, Québec, Canada

*First author

#corresponding author

Additional file 8: Supplementary Figures S1-S21.


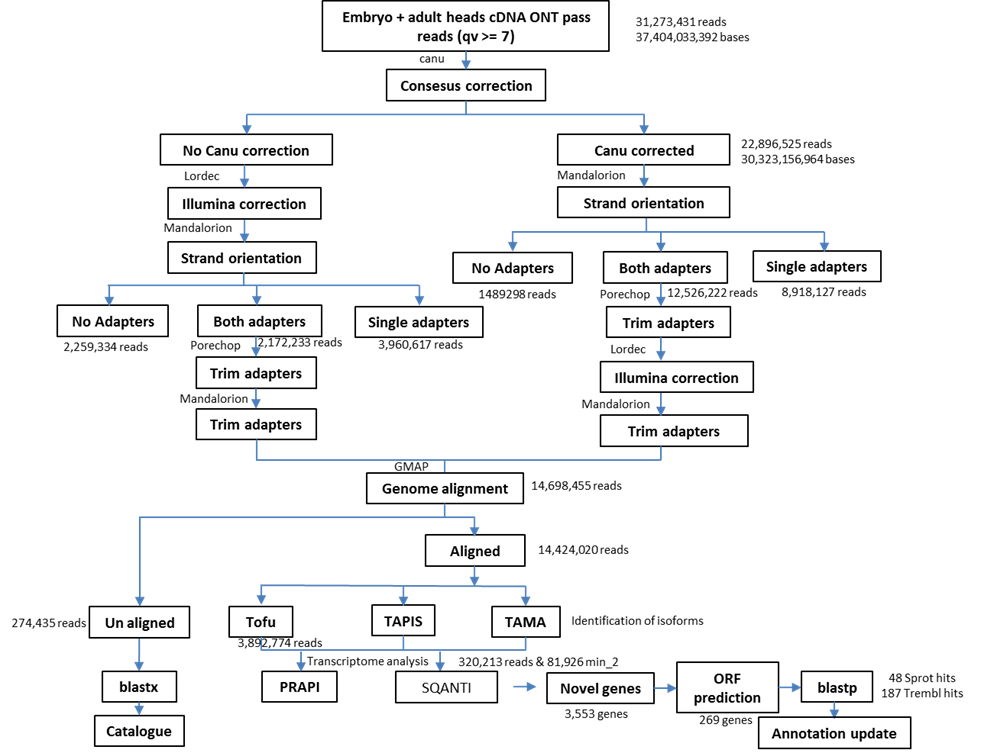


Figure S1: Long-read genome guided *de novo* transcriptome assembly workflow. All reads were provided to Canu to perform consensus error correction. A customized version of Mandalorion was used to return the correct original strand of each read based on detection of the 5’ and 3’ adapters. Only reads with both 5’ and 3’ adapters detected were used in transcriptome assembly. The adapters were trimmed using Porechop. Short-read Illumina reads were used to perform hybrid error correction with Lordec. Another customized version of Mandalorion was used to perform a final round of adapter trimming. Reads that had not been error corrected using Canu were taken through a similar pre-processing described above and combined with the error-corrected reads. The pre-processed reads were aligned to the genome using GMAP. ToFU was used to derive the final transcriptome assembly, followed by transcriptome analysis using SQANTI and PRAPI. TAMA, and TAPIS were also evaluated for transcriptome assembly.


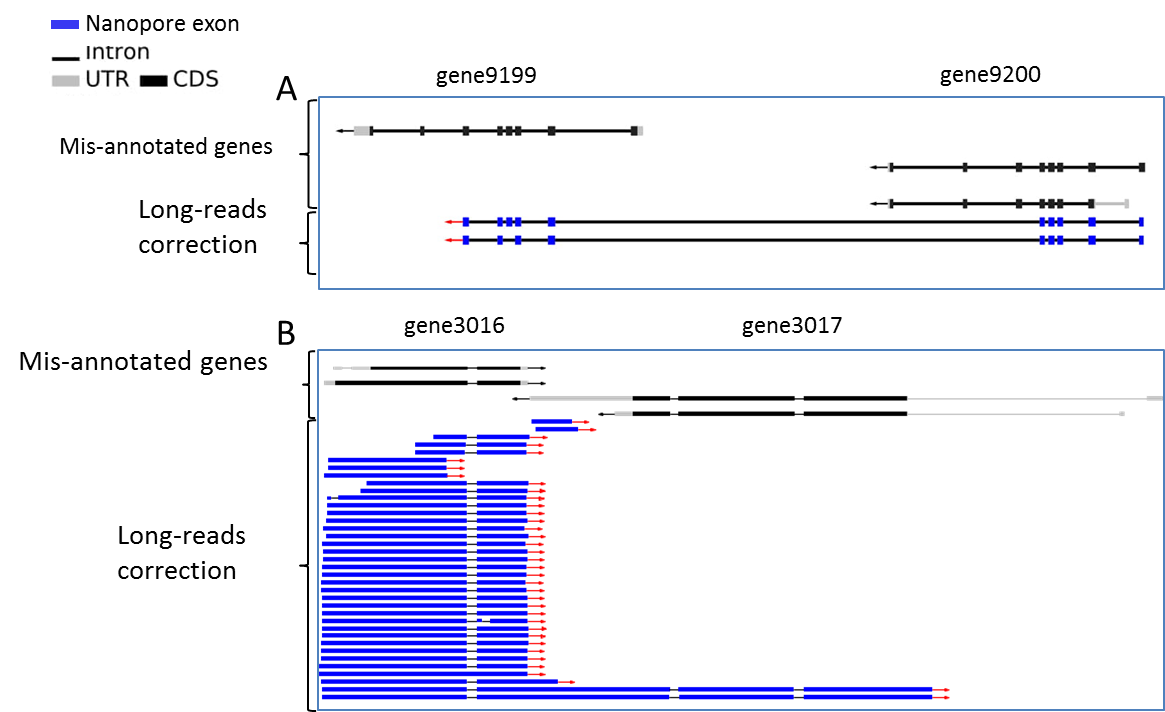


Figure S2: Long-read RNA-seq corrects mistakes in computationally predicted gene annotations. PRAPI was used to align transcripts to the NCBI predicted gene models and identify miss-annotated genes. Miss-annotated genes are described as single genes that were wrongly predicted as 2 or 3 separate genes. Two examples of such genes are shown here (A and B). The miss-annotated isoforms are shown on top while the long-read alignments are shown at the bottom of each panel. Full-length cDNA sequencing can provide evidence showing that miss-annotated genes are one single gene. See Table S8 for a full list of corrected genes.


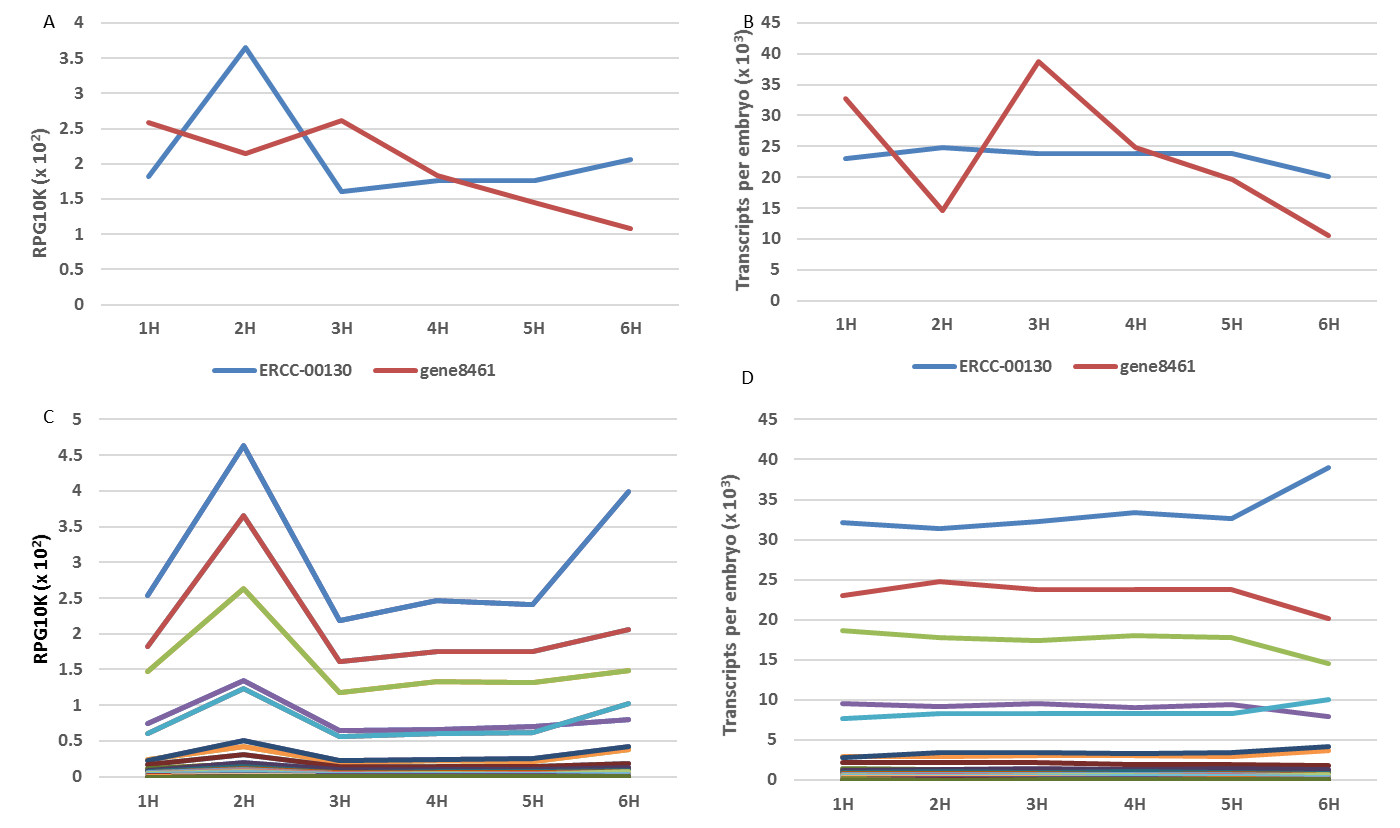


Figure S3: Comparison of relative normalization and absolute normalization of ERCC internal standards. A) Relative normalization of the most abundant ERCC (ERCC00130, blue) and a randomly picked gene (gene8461, red). Abundances of ERCC00130 varied with time (H=hours AEL) most likely due to changes in the amount of poly(A) RNA in the embryo. B) Same as A but showing absolute normalization of the most abundant ERCC (ERCC00130, blue) and a randomly picked gene (gene8461, red). Here, the abundance of the ERCC is stabilized across timepoints. C) Same as A but including all ERCC internal controls and excluding gene8461. D) Same as C but showing absolute normalization.


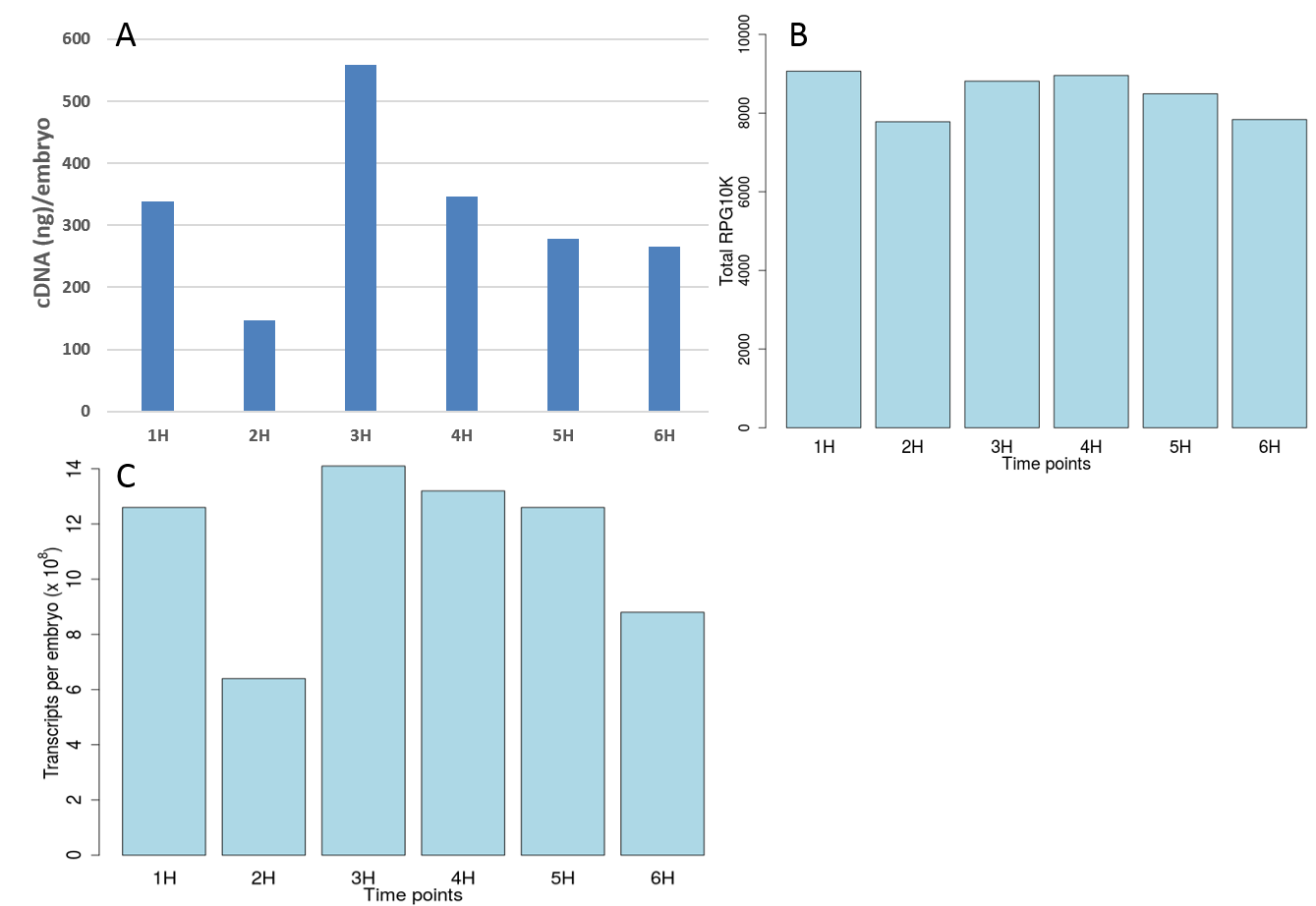


Figure S4: Comparison of relative normalization and absolute normalization of genes. A) Amount of synthesized cDNA per embryo at different timepoints (H; hours AEL). Equal amount of total RNA was used during cDNA synthesis. The amplified cDNA generated was purified and normalized to the number of embryos used at each timepoint. B) Summed expression values for all genes across timepoints. The relative method of quantifying gene expression was used. Here, read counts aligning to a gene are normalized by the total reads aligned to all other genes and further normalized to 10000 reads (RPG10K). This profile does not closely resemble the total cDNA profile in A. C) Same as B but using the absolute normalization and normalizing for the number of embryos. This profile shows close resemblance to the cDNA profile, demonstrating the advantage of absolute normalization.


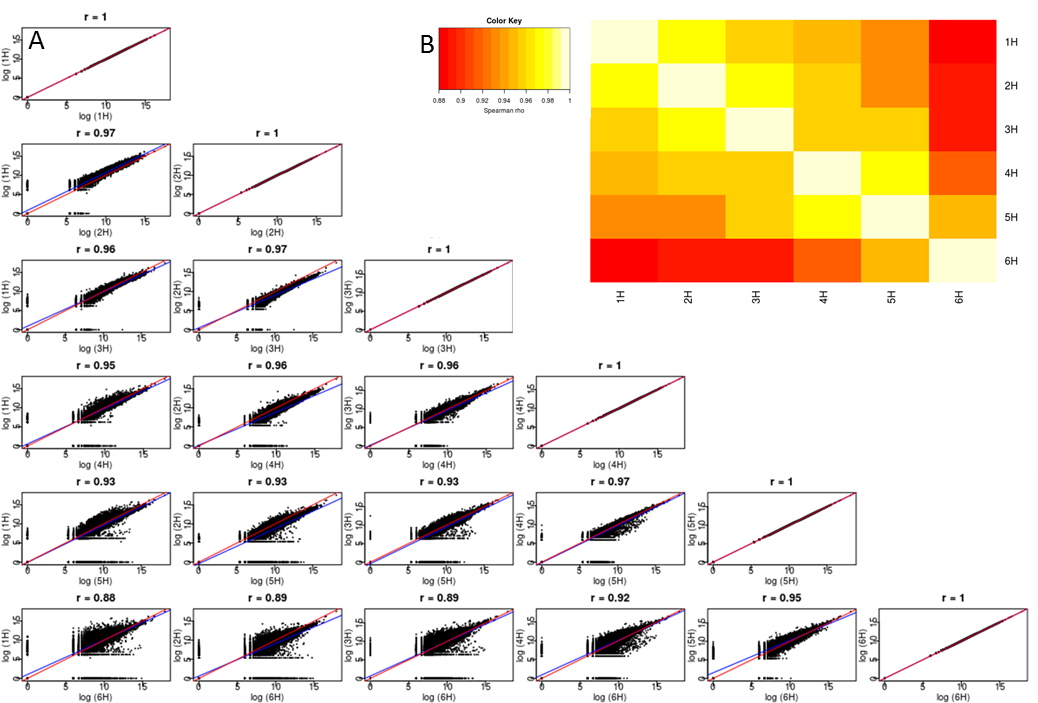


Figure S5: Correlation of gene expression across time-points. A) The Spearman rho (r) correlation of all expressed genes for each timepoint with itself and other timepoints is shown. Correlation was determined for the log absolute expression values. The plots are fitted with linear model (blue) and arbitrary line with intercept set at 0 and slope of 1 (red). B) Heatmap of the Spearman correlations from (A). Sample labels refer to the time (hours) after egg laying.


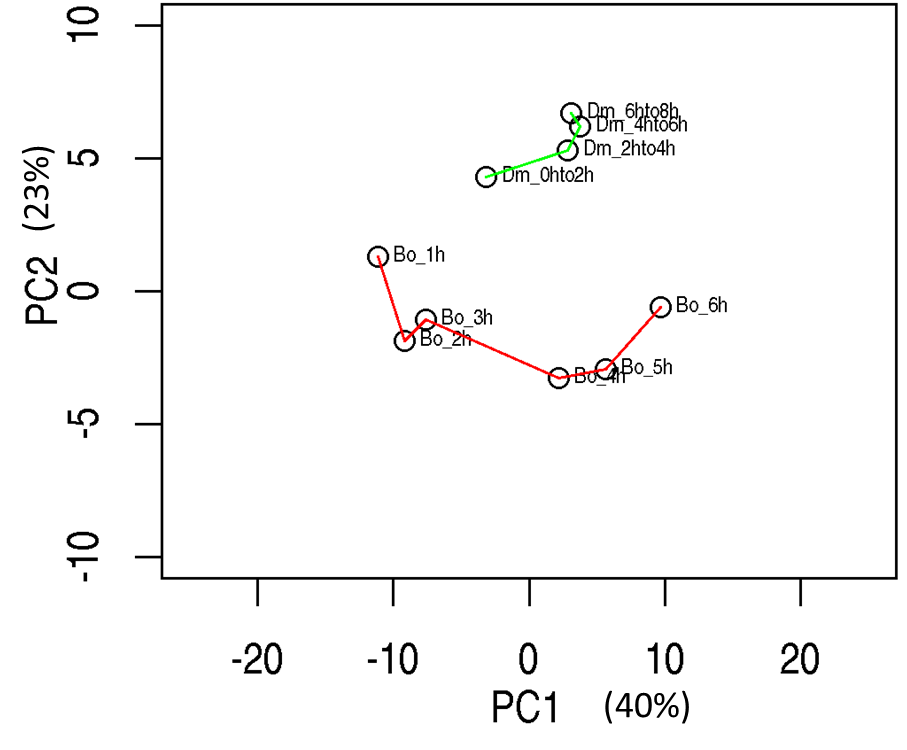


Figure S6: Principal component analysis (PCA) of early embryo development of *Bactrocera oleae* (red) and *D. melanogaster* (green). PCA was performed using the 100 most variable genes. For each organism the individual points are labelled with the corresponding scientific name initials and the hours after egg laying (Bo_1h for example refers to *B. oleae* 1 hour after egg laying). Data for *D. melanogaster* was downloaded from the FlyBase^1^.


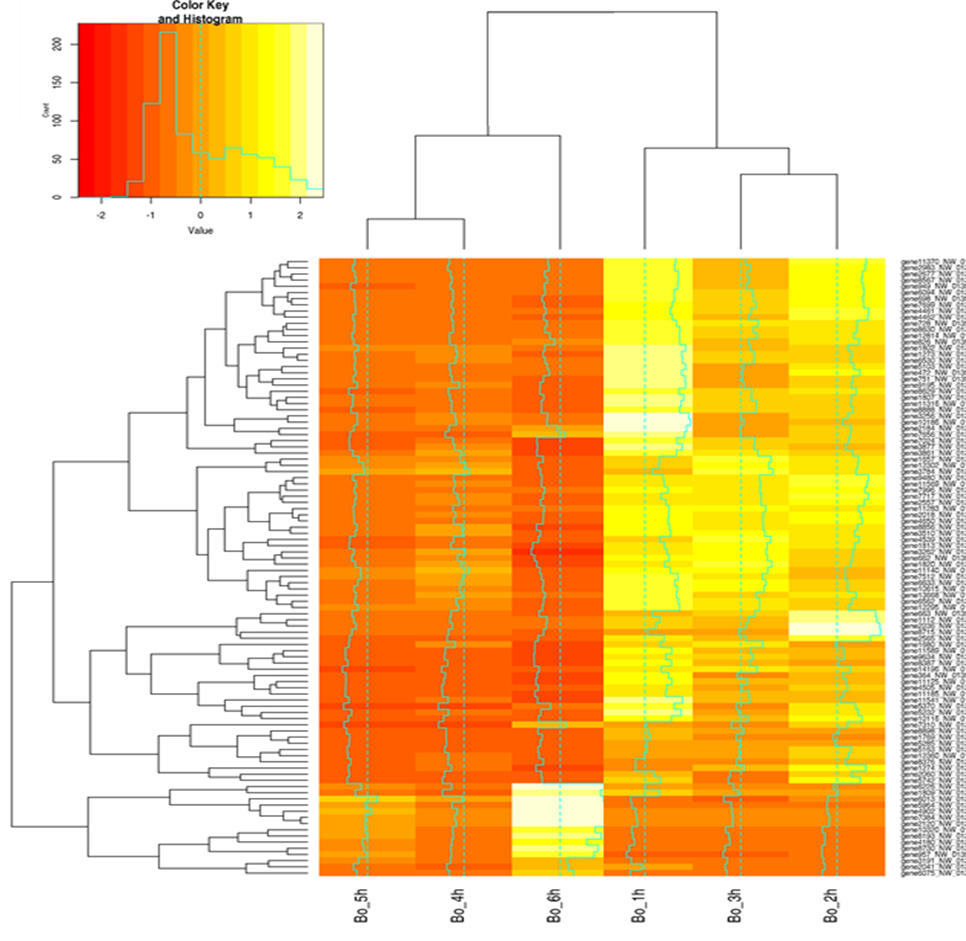


Figure S7: Hierarchical clustering of *Bactrocera oleae* embryo timepoints showing the similarity of transcript abundance between the 1-3 hours after egg laying (AEL) and 4-6 hours AEL timepoints, respectively. Clustering was done based on the 100 most variable genes from the 6 different experimental time points.


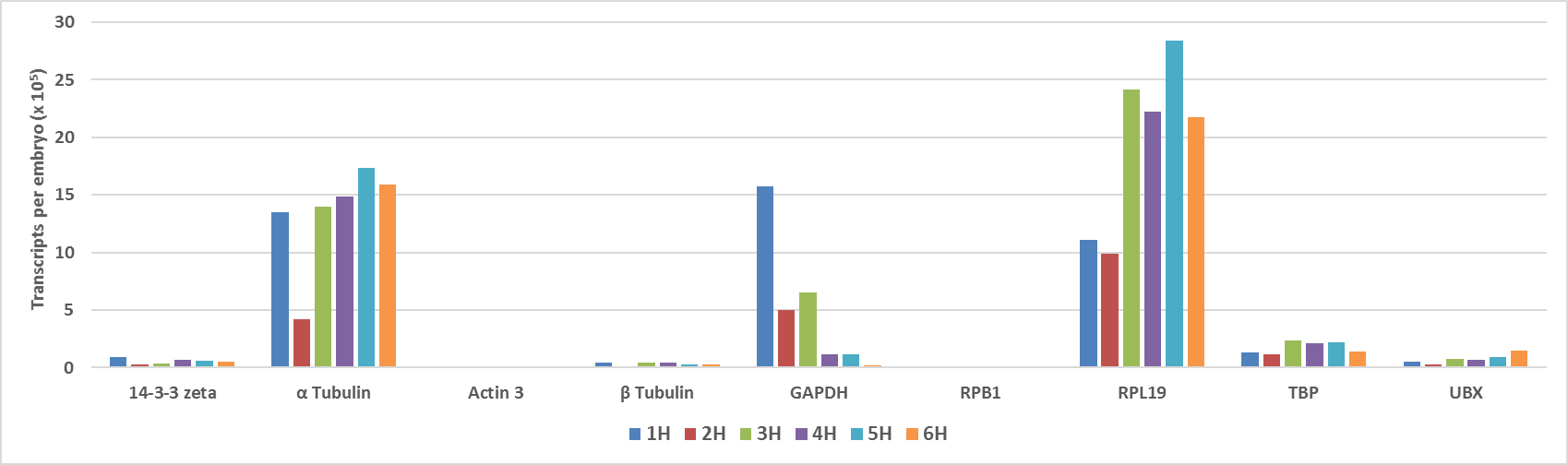


Figure S8: Comparison of absolute gene expression patterns of 9 genes routinely used as reference genes in qPCR normalization^2^. The number of transcripts per embryo is shown for *14-3-3 zeta*, *alpha Tubulin* (α-Tubulin), *Actin 3*, *beta Tubulin* (β-Tubulin), *Glyceraldehyde 3-phosphate dehydrogenase* (GAPDH), *DNA-directed RNA polymerase II* (RPB1), *ribosomal protein L19* (*RPL19*), *TATA box binding protein* (*TBP*), and *homeotic protein ultrabithorax* (UBX).


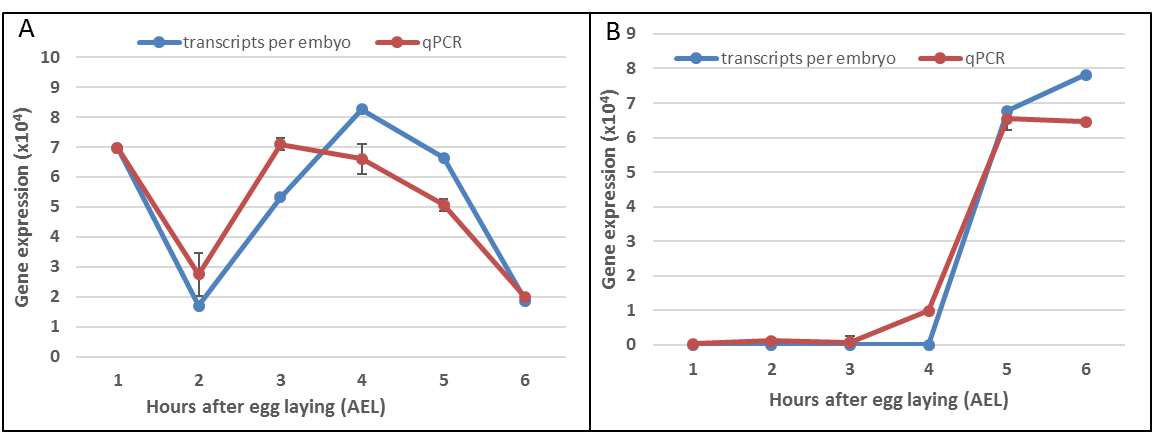


Figure S9: mRNA content per embryo and qPCR expression of *hid* and *sry-a*. A) Absolute expression (blue) and qPCR expression (red) of serendipity alpha (*sry-a*) normalized with 14-3-3 *zeta*. qPCR expression values were scaled to compare expression profiles. B) Same as A but for head involution defective *(hid)*. These results were generated using a different set of biological samples. The qPCR used oligo(dT) and random primers during the reverse transcription step. Standard error of the mean of two biological replicates is depicted in bars.


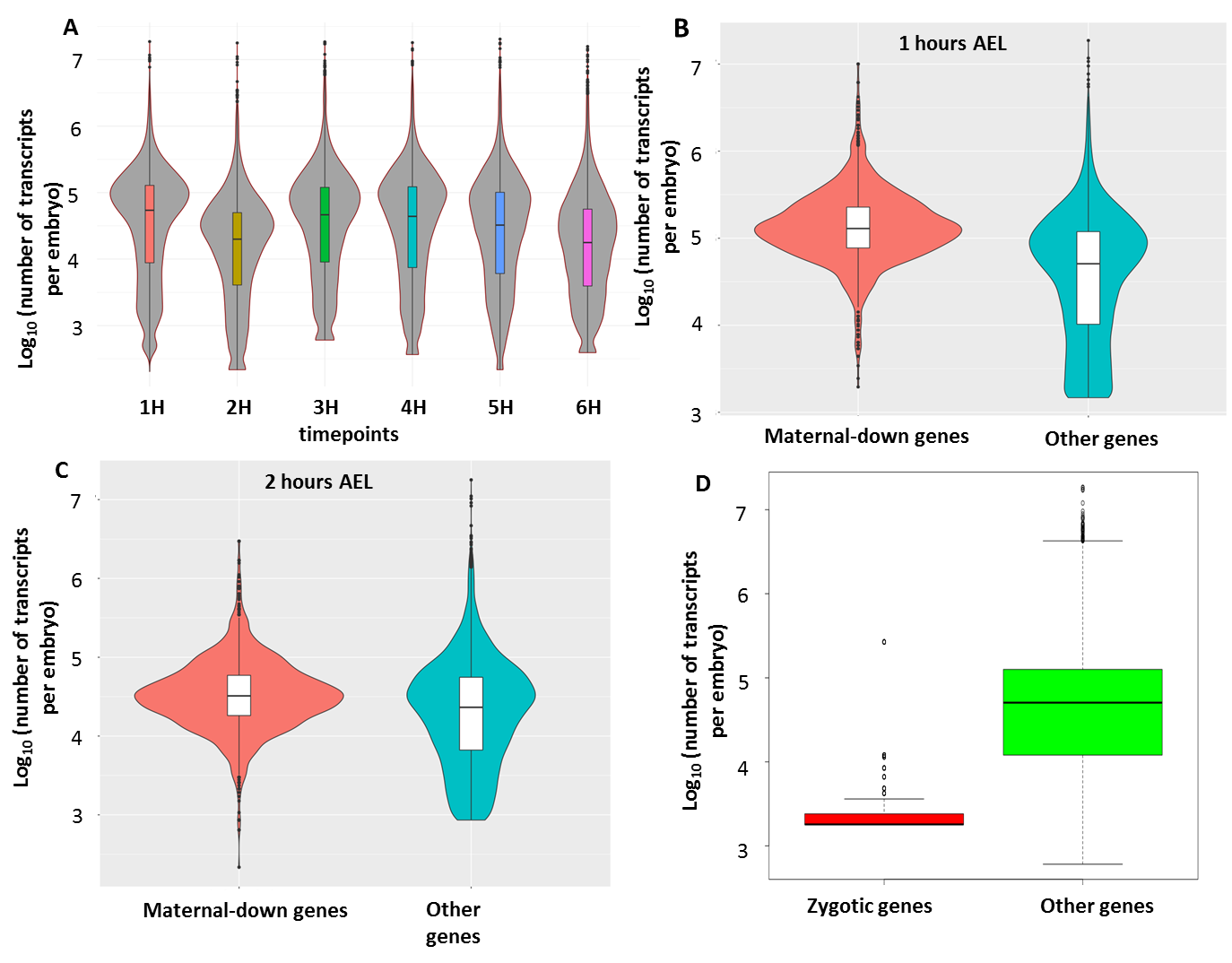


Figure S10: Profile of gene expression during embryo development reveals reduction in abundance of highly expressed maternal genes. A) Violin plot showing the variation in gene expression across timepoints. B) Violin plot comparing gene expression between maternal-down genes and the rest of the genes at 1-hour after egg laying (AEL). C) Same as B but for 2 hours AEL. D) Boxplot comparing gene expression pattern of zygotic genes to all the other genes at 3 hours AEL. Maternal-down genes are defined as genes that were most significantly reduced in abundance between 1-hour AEL and 2 hours AEL (log2 fold change >1). Zygotic genes are genes whose expression was not detected at 1 or 2 hours AEL but detected thereafter suggesting they were transcribed from the zygotic genome as opposed to being maternally derived. This figure shows that some maternally derived transcripts are at very high abundance and within the 2^nd^ hour of embryo development these genes are downregulated such their levels is similar to other genes. This re-organization does not involve any major zygotic genome activation.


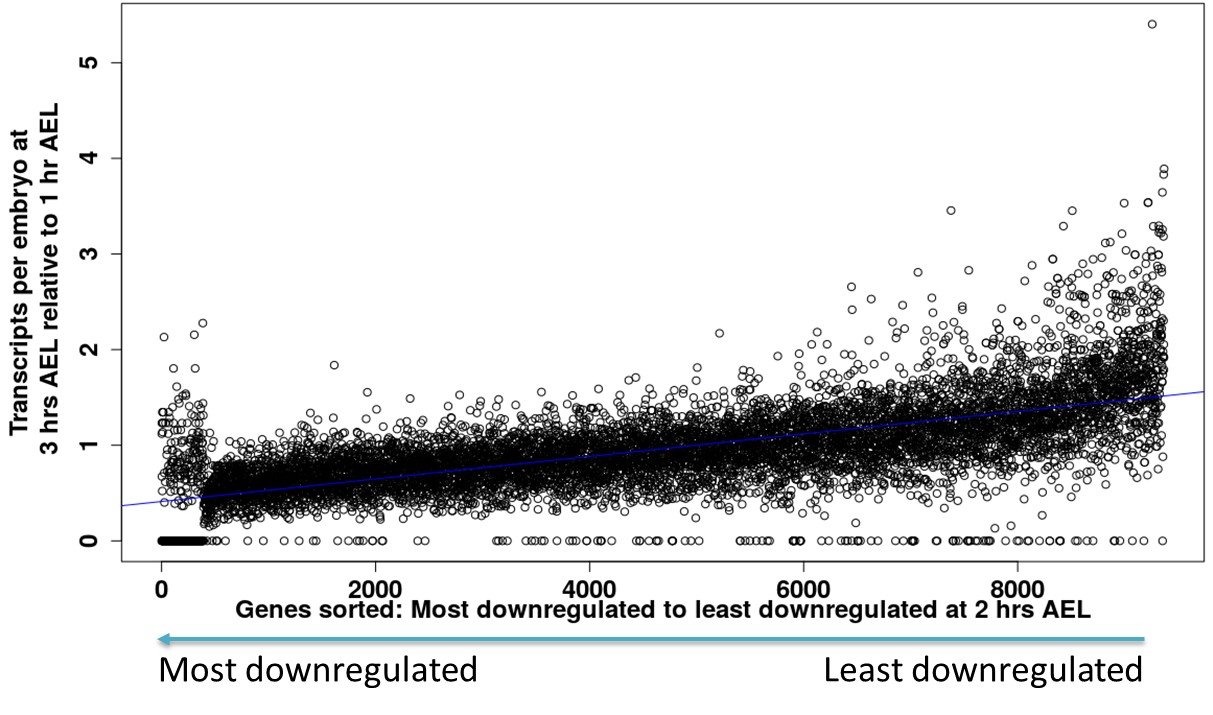


Figure S11: Reorganization of maternal transcripts prior to maternal-to-zygotic transition. We noted a drop in expression for majority of genes at 2 hours after egg laying (AEL). The percentage difference in expression of all quantifiable genes at 2 hours AEL compared to 1 hour AEL was computed as [100*(1_hr_AEL – 2_hr_AEL)/1-hr_AEL]. Genes were sorted from highest percentage (most down regulated) to lowest percentage (least down regulated). We then computed the relative expression of all these genes at 3 hours AEL compared to 1 hour AEL as [3_hr_AEL/1_hr_AEL]. The figure shows that maternally supplied genes that are most down regulated at 2 hours AEL are not later enriched at 3 hours AEL. However, the least downregulated genes are enriched at 3 hours AEL suggesting a reorganization of maternal transcripts.


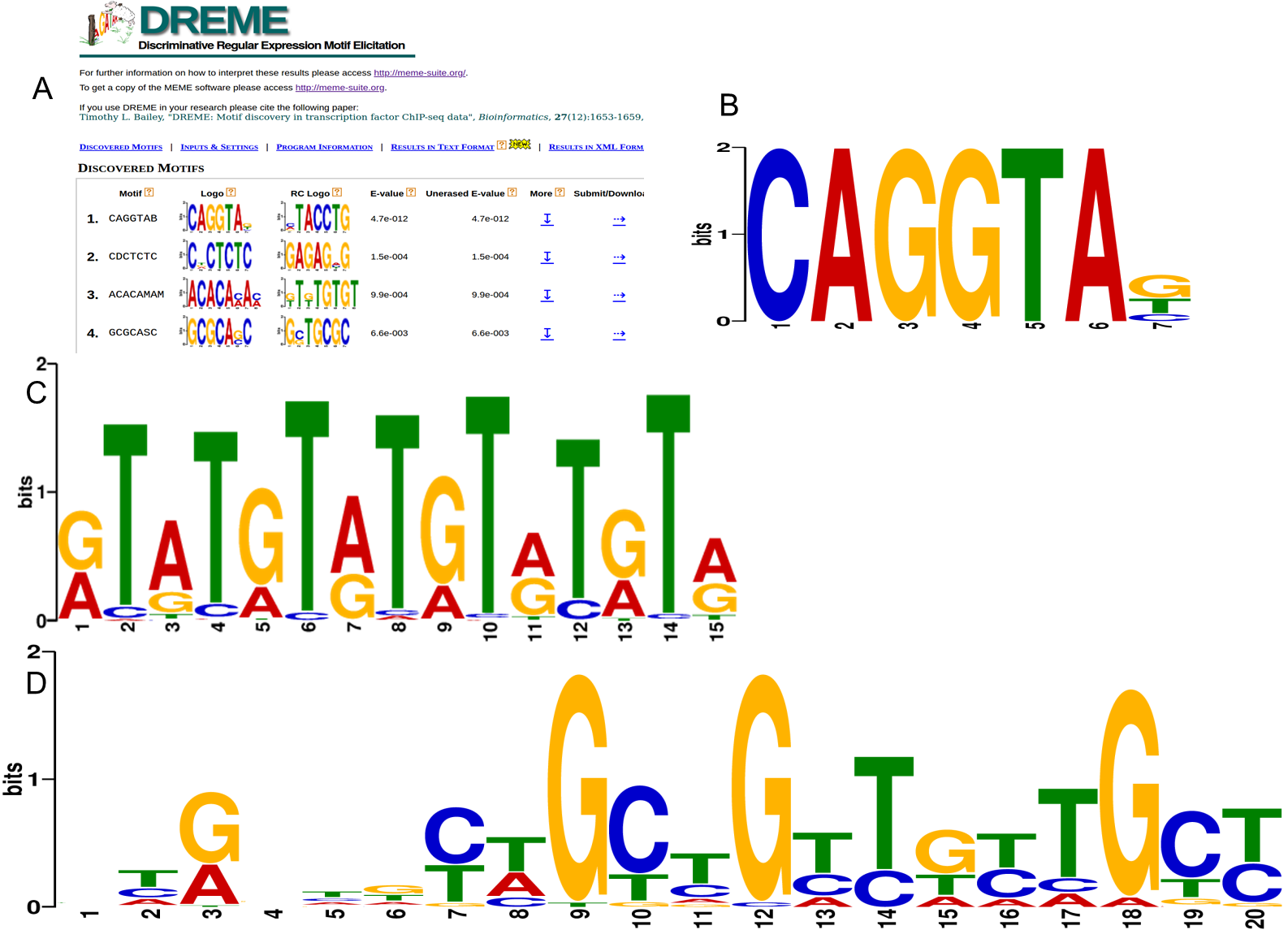


Figure S12: Motifs identified in promoter regions of zygotic and maternal genes. A) Screenshot showing results obtained with DREME tool of MEME suite^3,4^. Differentially enriched motifs in promoter sequences (1000 bp upstream of transcription start sites, TSS) between zygotic-early genes and genes that are maternally supplied but downregulated and have no evidence of being transcribed from the zygote were searched using DREME. This identified the CAGGTAB motif shown in (B) as the most enriched. C) RTATGTRTGTRTRTR motif found to be enriched in the promoter regions of 1132 zygotic genes (genes that were not detectable at 1 or 2 hours AEL but detectable at other time points). This enriched motif was identified using MEME^5^ (*E-value* 2.0e-138). D) Motif enriched in Maternal-down genes (these are maternally supplied genes that are downregulated at 2 hours after egg laying and have no evidence of being transcribed from the zygote).


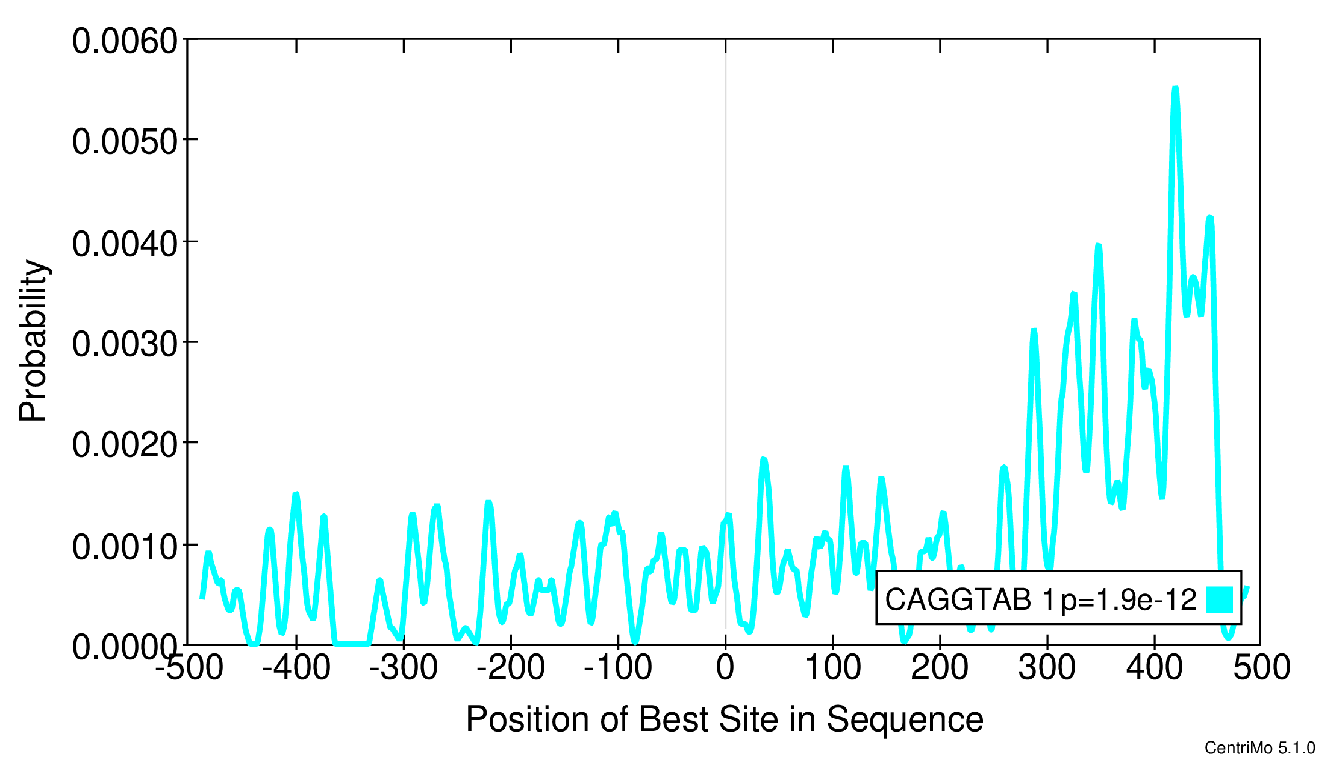


Figure S13: Significant CAGGTAB motif enrichment 500 bp upstream of transcription start (TSS). A region 1000 bp upstream of the TSS of 159 early zygotic genes identified using GFOLD differential expression was examined for motif enrichment using CentriMo^6^. The output shows significant CAGGTAB motif enrichment in 500 bp upstream of the TSS. The X-axis shows the 1000 bp region whereby 0 marks the middle of the sequence and position 500 is at the TSS.


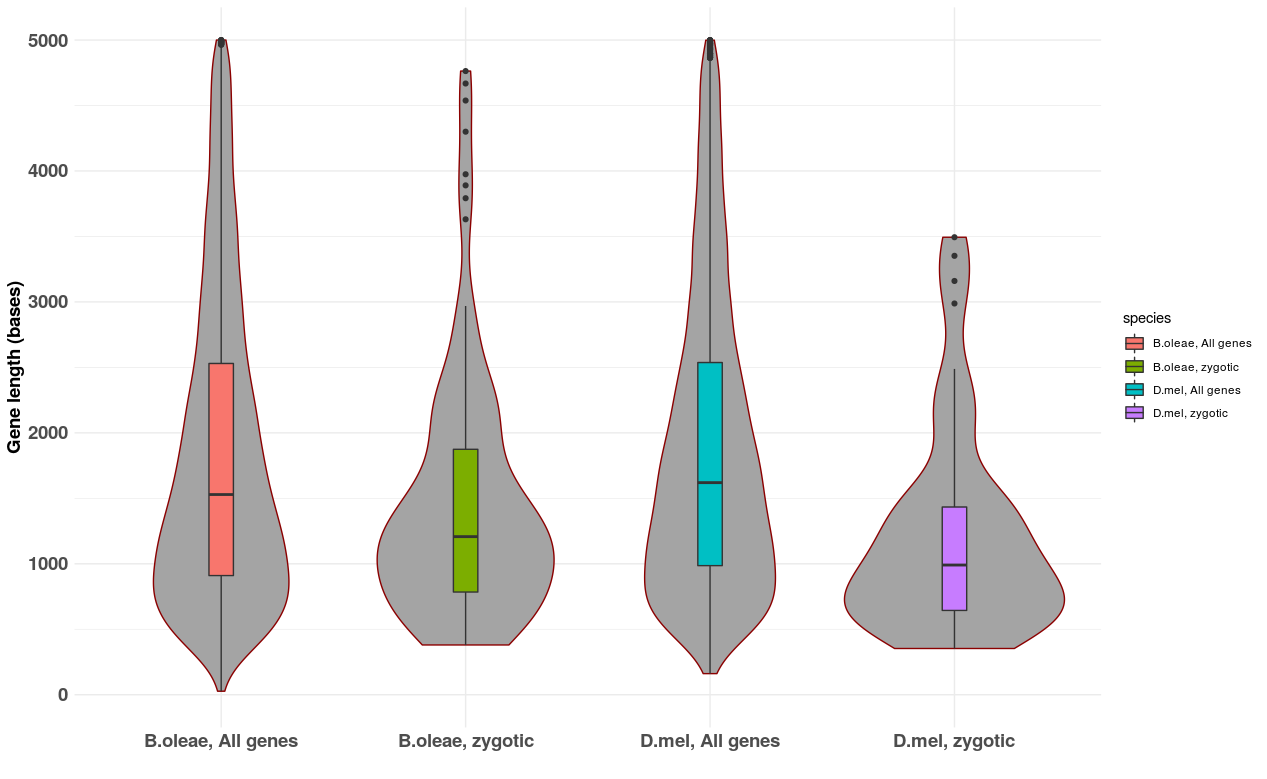


Figure S14: Early zygotic genes are shorter in length compared to other genes in both *Bactrocera oleae* and *Drosophila melanogaster*. Gene transfer files (GTF) for gene model annotations from both *Bactrocera oleae* (B.oleae) and *Drosophila melanogaster* (D.mel) were used to determine the lengths for all protein coding genes and early zygotic protein coding genes. *B. oleae* early zygotic genes were identified in this study while *D. melanogaster* early zygotic genes were identified by De Renzis *et al*.^7^. *B. oleae* GTF file was taken from the NCBI *Bactrocera oleae* annotation release 100 including the novel genes identified in this study while *D. melanogaster* GTF file was from flybase^1^ version r6.32. Zygotic gene lengths for both *B. oleae* and *D. melanogaster* differ significantly from nonzygotic genes (Wilcox p-values 5.1e -7 and 9.1e-7, respectively)


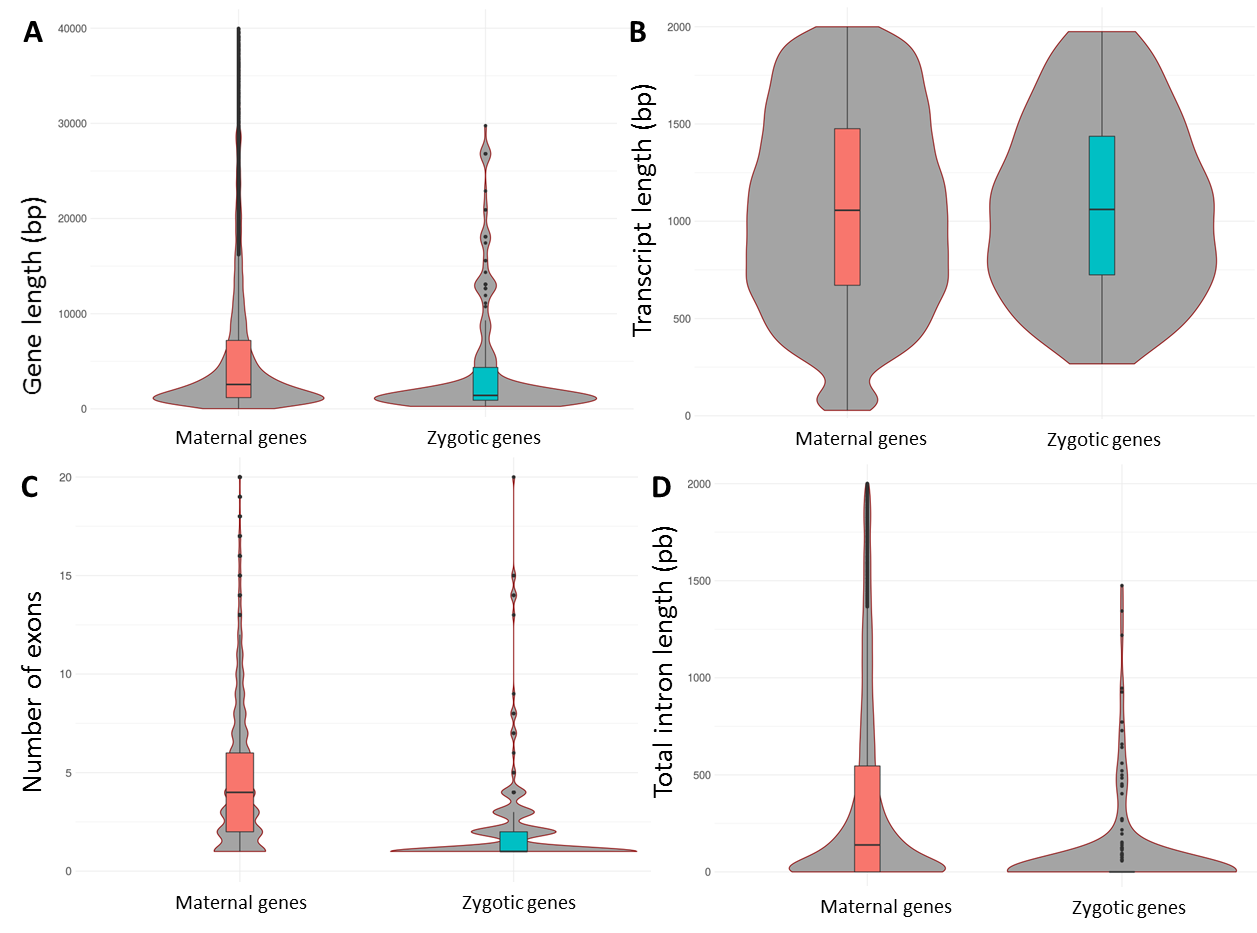


Figure S15: Comparison of zygotic and maternal genes lengths in *Bactrocera oleae*. Comparison of zygotic and maternal gene body length (A), transcript length (B), number of exons (C), and total intron length (D). Both zygotic and maternal genes were identified using GFOLD differential expression method. The *B. oleae* maternal gene lengths and zygotic gene lengths (A) were significantly different (Wilcox p-value 5.2e-8) whereas their transcript lengths (B) were not significantly different (Wilcox p-value 0.8).


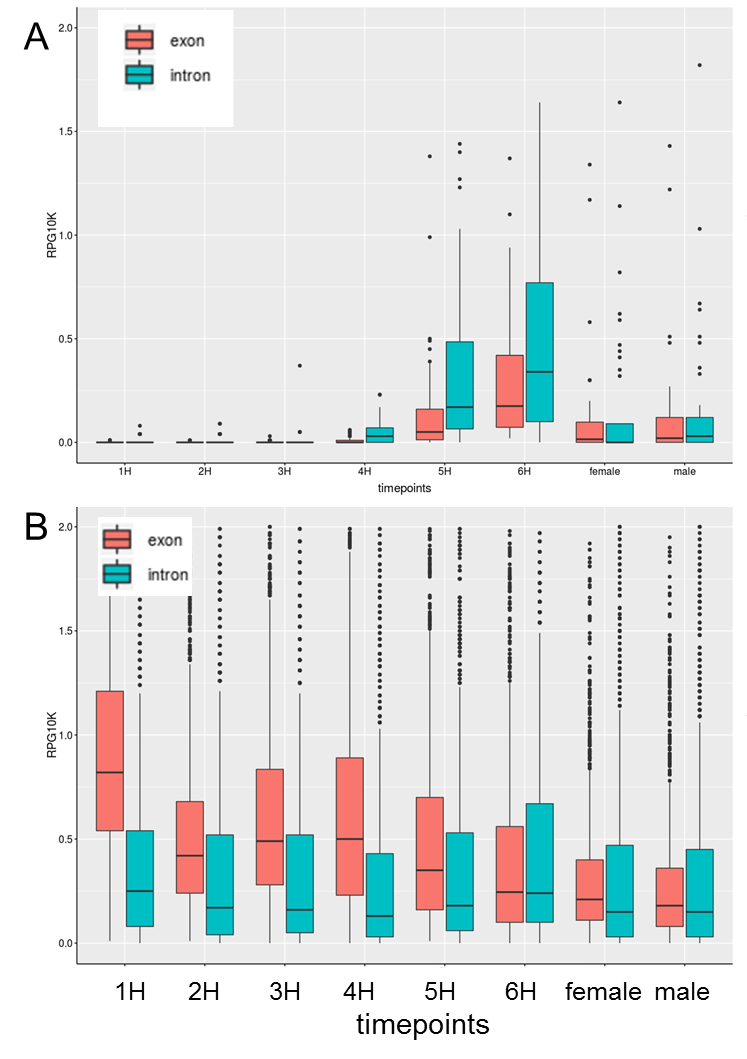


Figure S16: Comparison of intron and exon coverage among zygotic (A) and maternal-down genes (B). Zygotic and maternal genes were initially determined using GFOLD differential expression. The relative abundance of transcripts having reads that exclusively align to introns or exons was estimated using Mandalorion. Reads per gene per 10000 mapped reads (RPG10K).


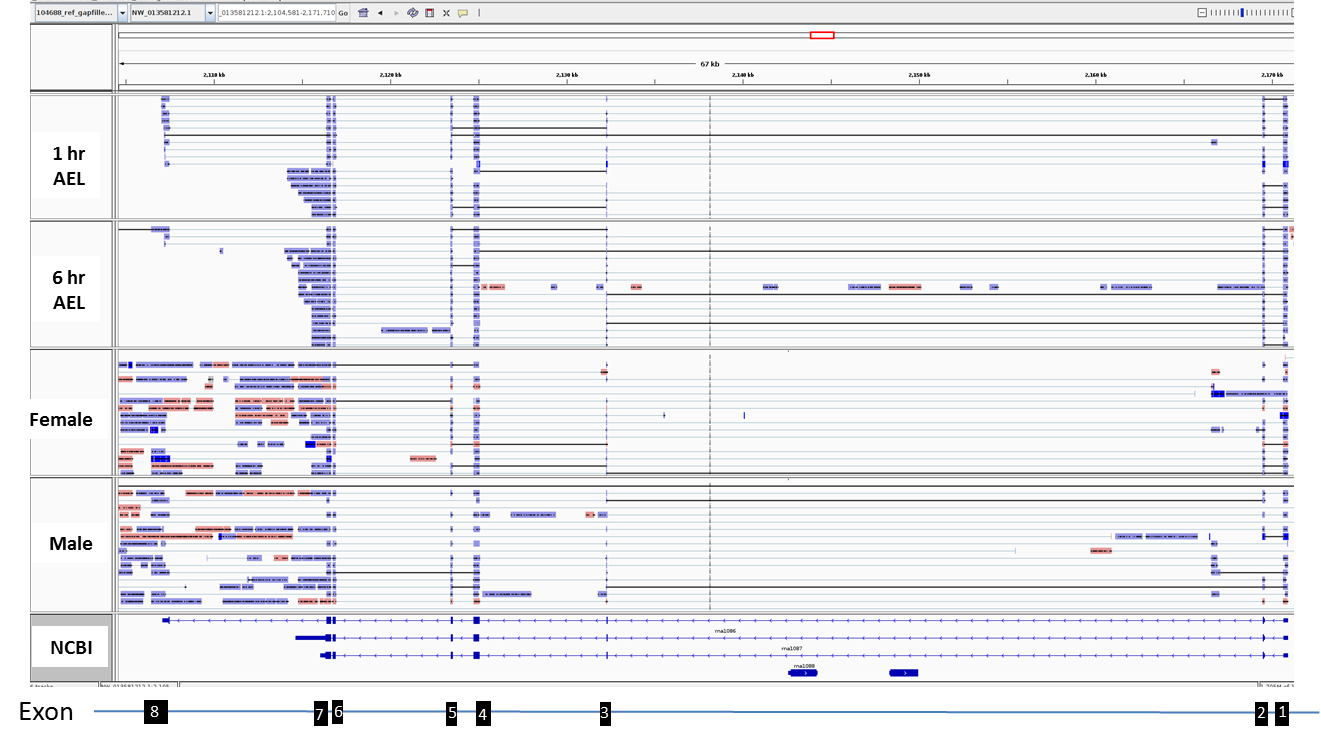


Figure S17: IGV screenshot showing long read alignments to *Bactrocera oleae* *sex lethal* (*Sxl*). Panels show alignment of raw long reads generated from embryos at 1 hour after egg laying (AEL), 6 hours AEL, adult female heads, and adult male heads, respectively. The NCBI predicted gene model is shown in the bottom-most panel*.* IGV=Integrated genome viewer


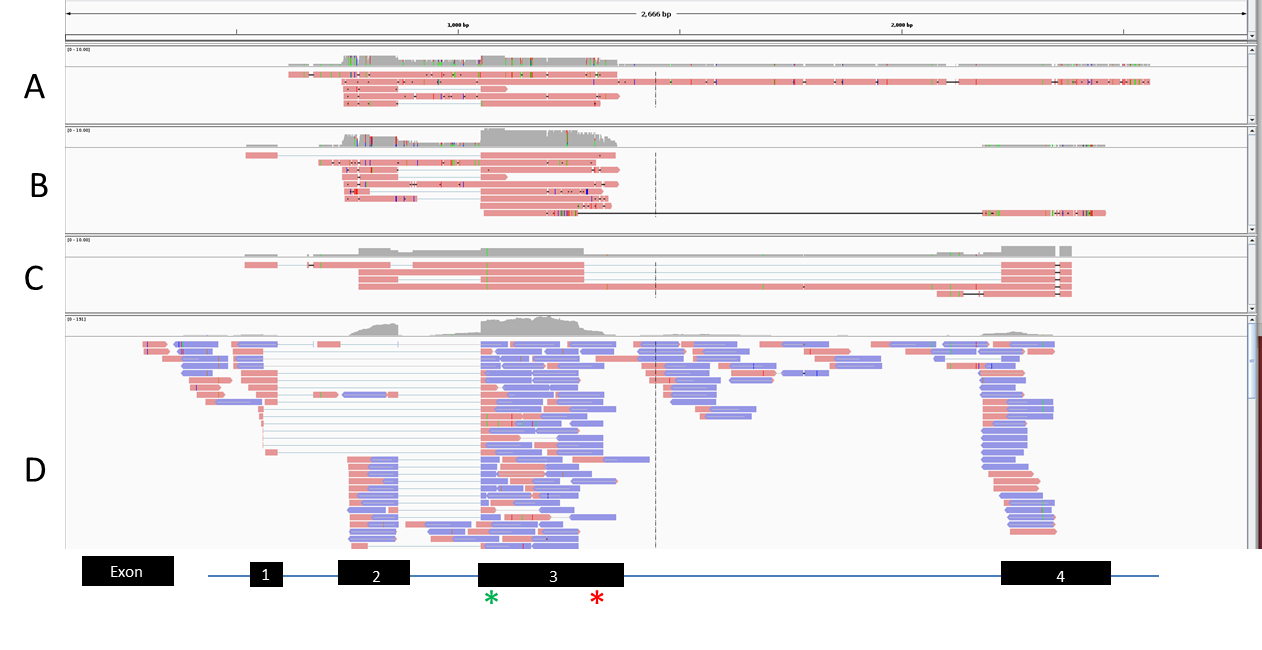


Figure S18: IGV screenshot showing read alignments to *Bactrocera oleae* maleness on Y (*BoMoY*) gene. A) First *BoMoY* transcripts observed at 5 hours after egg laying (AEL) using long-read RNA-seq, B) *BoMoY* transcripts observed at 6 hours AEL using long-read RNA-seq, C) *BoMoY* Trinity assembled isoforms obtained from Meccariello *et al*.^8^, D) Paired-end reads obtained from Illumina short-read RNA-seq of embryo samples at 6 hours AEL. Illumina reads are shown as combined forward-reverse. The gene model is shown as black boxes below panel D. Asterisks indicate location of start codon (green) and stop codon (red). IGV=Integrated genome viewer


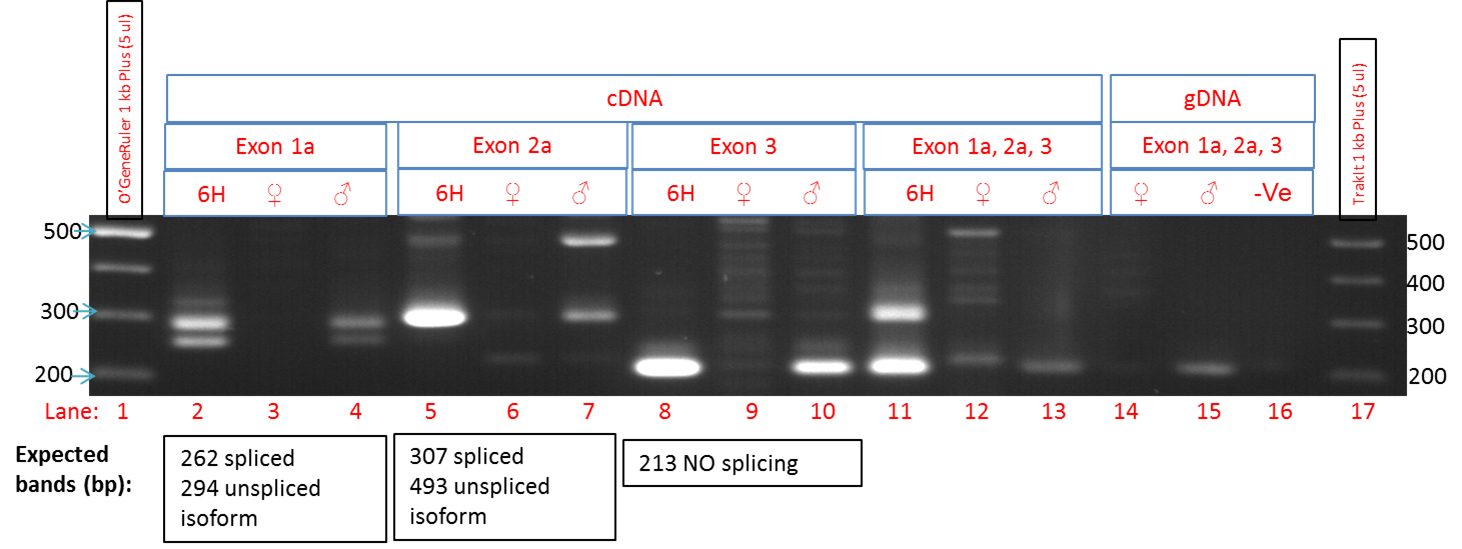


Figure S19: PCR amplification of regions of *Bactrocera oleae* maleness on the Y chromosome (*BoMoY*) transcripts. Lane 2 to 13: PCR products amplified using forward primers targeting the shown exons. The BoMoY_R_exon3 reverse primer was used for all reactions. Lanes 11 – 16 used a multiplex of forward primers. For reactions in lanes 1-13, cDNA generated from embryos at 6 hours after egg laying (6H AEL), adult female heads (♀), and adult male heads (♂) was used. For reactions in lanes 14 and 15, genomic DNA extracted from adult whole female and male insects, respectively, was used. Lane 16 shows the negative control. Lanes 1 and 17 show the ladders O’GeneRuler 1 kb plus (Thermo Fisher Scientific) and TrackIt 1 kb Plus (Thermo Fisher Scientific), respectively, and their band sizes in base pairs. See Table S13 for a list of the primers used. No PCR product is expected from female samples since *BoMoY* is located on the Y chromosome.


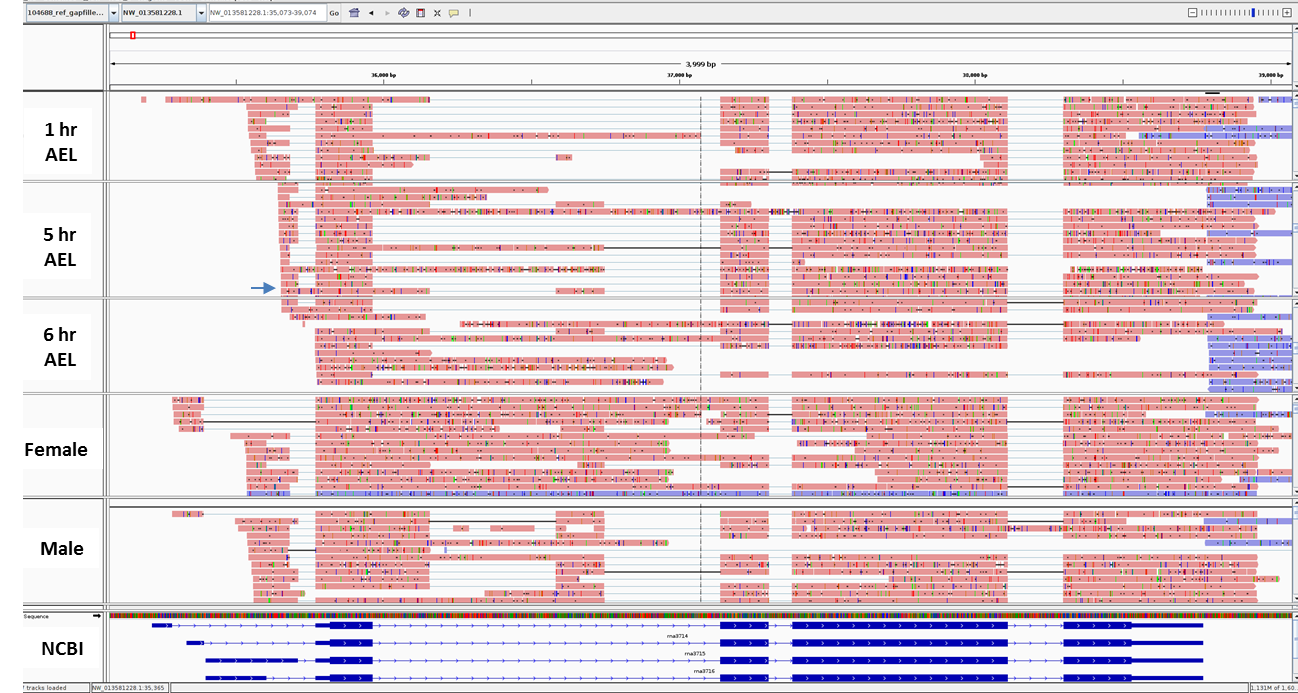


Figure S20: IGV screenshot showing long read alignments to *Bactrocera oleae* *transformer* (*tra*). Panels show alignment of raw long reads generated from embryos at 1 hour after egg laying (AEL), 5 hours AEL, 6 hours AEL, adult female heads, and adult male heads, respectively. The NCBI predicted gene model is shown in the bottom-most panel*.* The arrow indicates the male-specific isoform first detected at 5 hours AEL. IGV=Integrated genome viewer


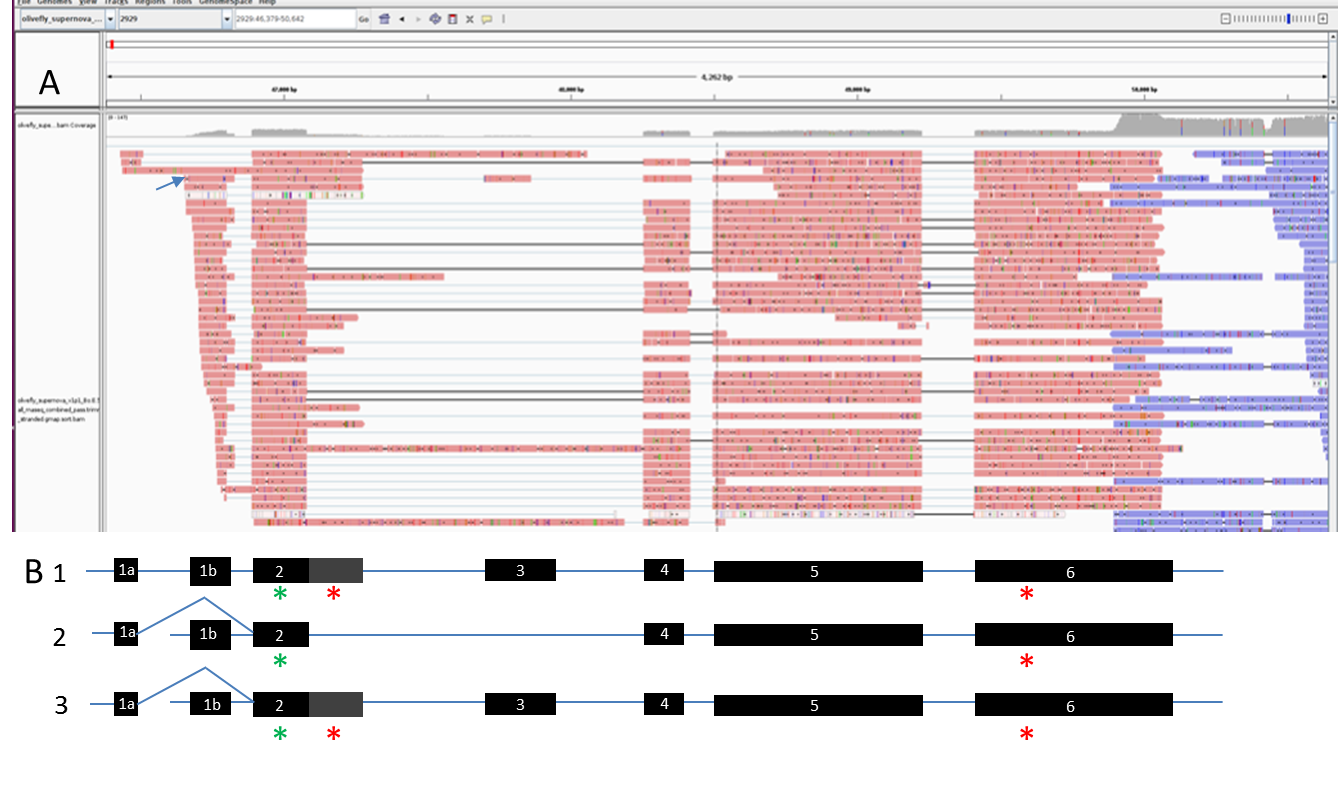


Figure S21: IGV screenshot showing long read alignments to *Bactrocera oleae* *Transformer* gene (*tra*). A) IGV screenshot showing reads aligned to *tra* observed in our bulk long-read RNA-seq of mixed-sex embryos at 5 hours after egg laying. Arrow indicates the signature transcript showing male-specific splicing pattern characterized by extension of exon 2 and retention of exon 3. B) Schematic of the full *tra* gene model (B1), female-specific *tra* isoform (B2) and male-specific *tra* isoform (B3). Asterisks indicate location of start codon (green) and stop codon (red). IGV=Integrated genome viewer


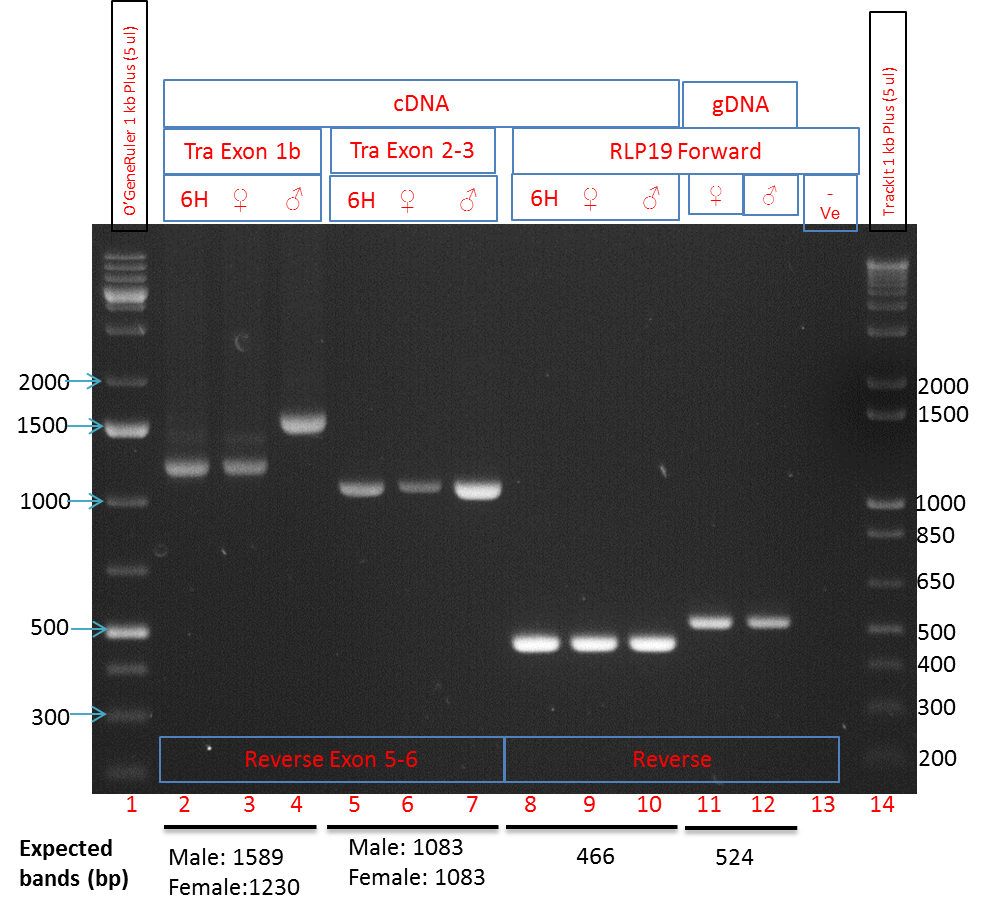


Figure S22: PCR amplification of regions of *Bactrocera oleae* Transformer (*Tra*) transcripts. Lane 2 to 7 show PCR products amplified using forward primers targeting exon 1b and the splice junction of exons 2 and 3. The Botra_R_exon5-6 reverse primer was used for these reactions. Ribosomal protein L19 (RPL19) was added as a positive control in lanes 8-12. For reactions in lanes 2-10, cDNA generated from embryos at 6 hours after egg laying (6H AEL), adult female heads (♀), and adult male heads (♂) was used. For reactions in lanes 11 and 12, genomic DNA extracted from adult whole female and male insects was used. Lane 13 shows the negative control. Lanes 1 and 14 show the ladders O’GeneRuler 1 kb plus and TrackIt 1 kb plus, respectively, and their band sizes (Thermo Fisher Scientific). See Table S13 for a list of the primers used.


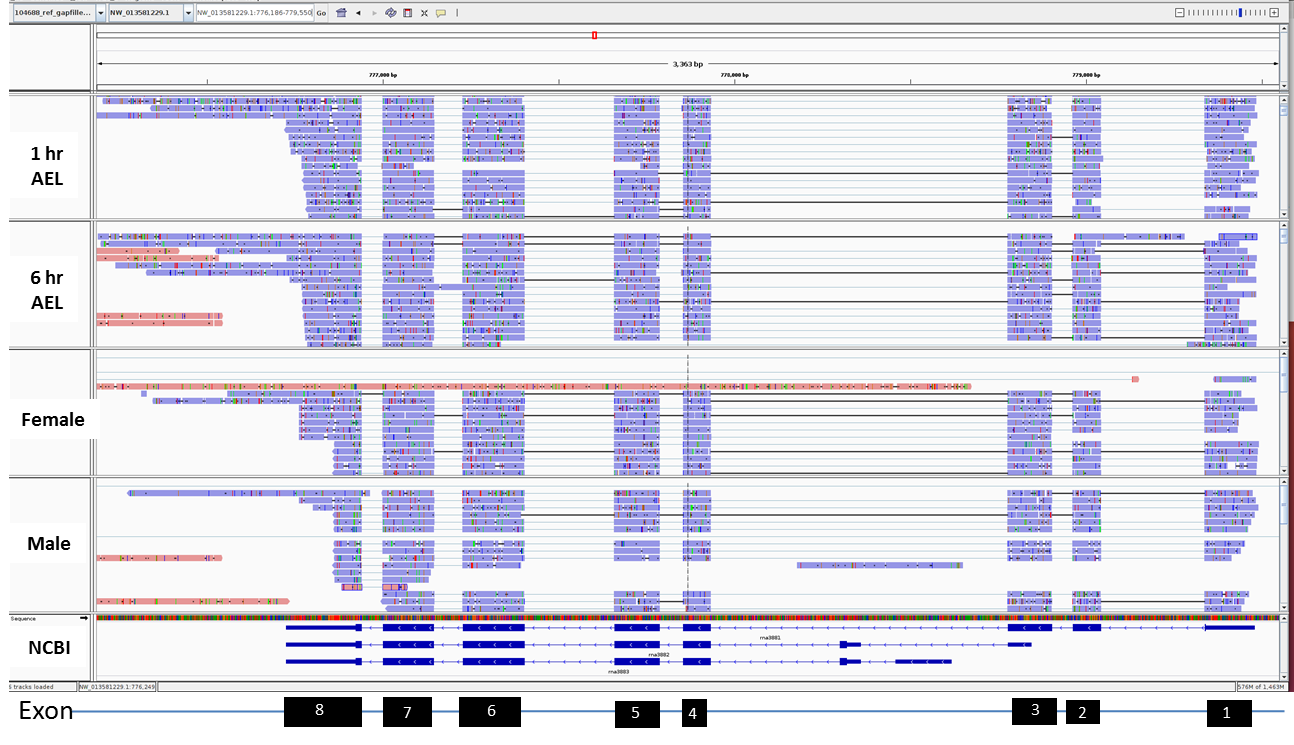


Figure S23: IGV screenshot showing long read alignments to *Bactrocera oleae* *transformer 2* (*tra2*). Panels show alignment of long reads generated from embryos at 1 hour after egg laying (AEL), 6 hours AEL, adult female heads, and adult male heads, respectively. The NCBI predicted gene model is shown in the bottom-most panel*.* IGV=Integrated genome viewer


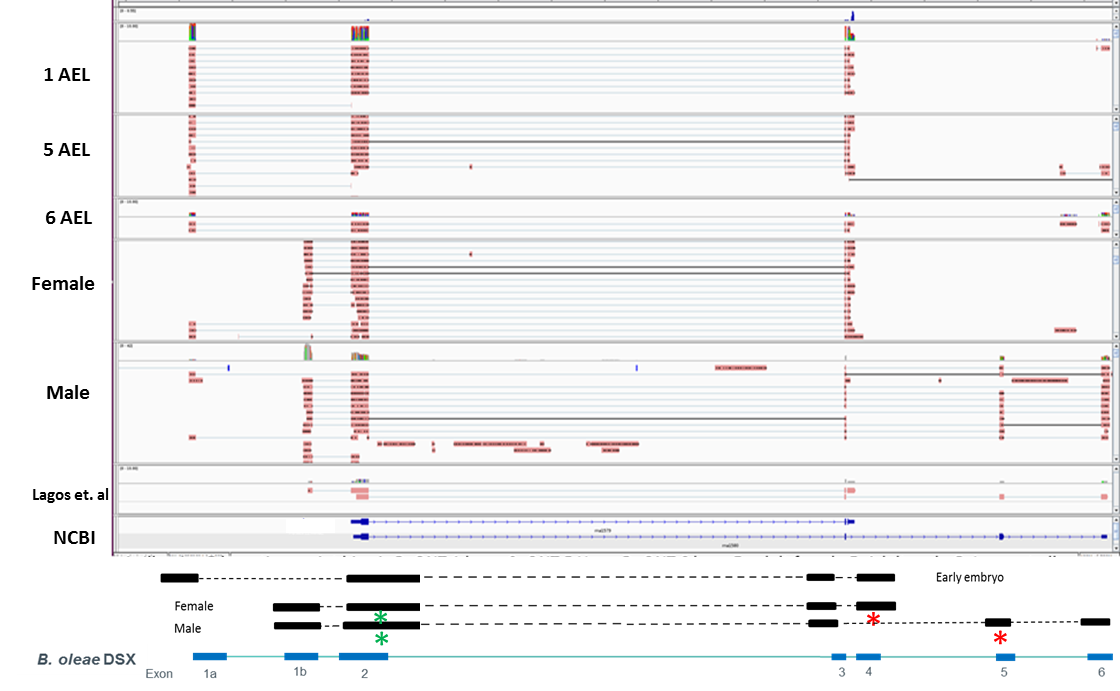


Figure S24: IGV screenshot showing long read alignments to *Bactrocera oleae double sex* (*dsx*). Reads are shown for embryos at 1, 5, and 6 hours after egg laying (AEL), and female and male adult heads. Male and female specific *dsx* cDNA as generated by Lagos *et al*.^9^ are shown. NCBI predicted gene model for *dsx* is shown in the bottom-most panel*.* The dominant isoform from the embryo stage, female heads, and male heads are shown below the screenshot. Asterisks indicate location of start codon (green) and stop codon (red). IGV=Integrated genome viewer

**
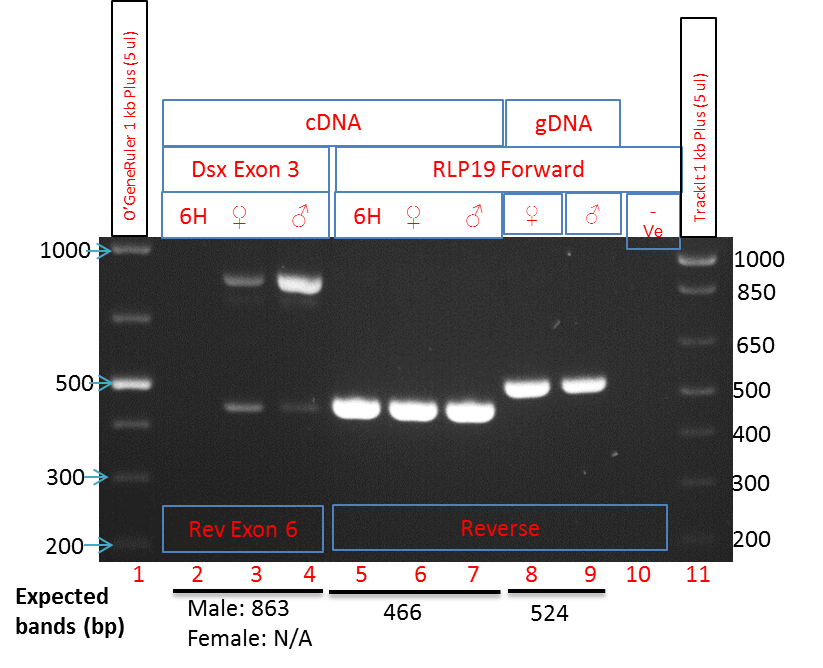
**

Figure S25: PCR amplification of regions of *Bactrocera oleae* Double sex (*Dsx*) transcripts. Lane 2 to 4 show PCR products amplified using forward primers targeting the region from exons 3 to 6. Ribosomal protein L19 (RPL19) was added as a positive control in lanes 5 to 9. For reactions in lanes 2-7, cDNA generated from embryos at 6 hours after egg laying (6H AEL), adult female heads (♀), and adult male heads (♂) was used. For reactions in lanes 8 and 9, genomic DNA extracted from adult whole female and male insects was used. Lane 10 shows the negative control. Lanes 1 and 11 show the ladders O’GeneRuler 1 kb plus and TrackIt 1 kb plus (Thermo Fisher Scientific), respectively, and their band sizes. See Table S13 for a list of the primers used.

**
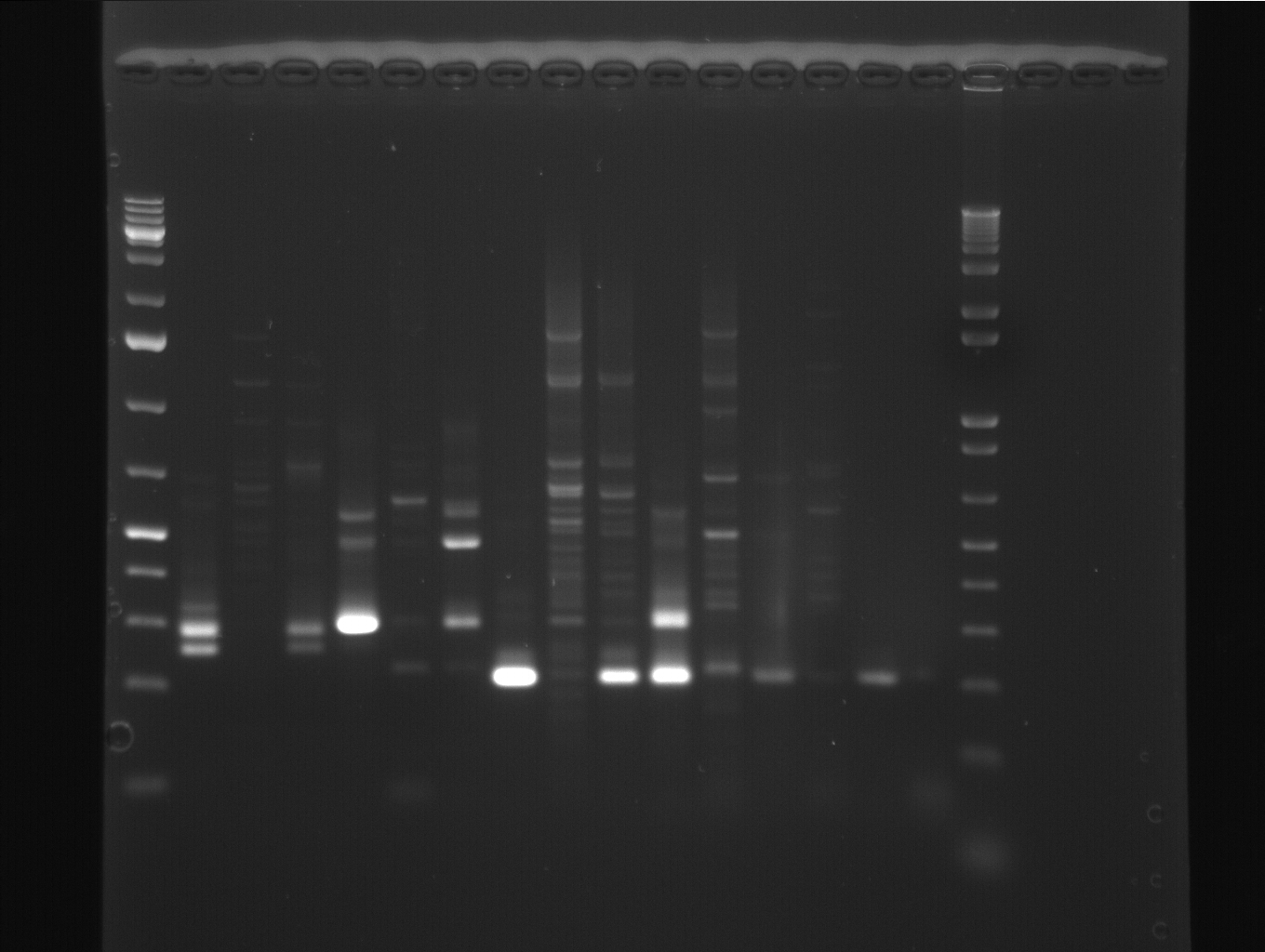
**

**Figure S26: Full uncropped version of Figure S19.**

**
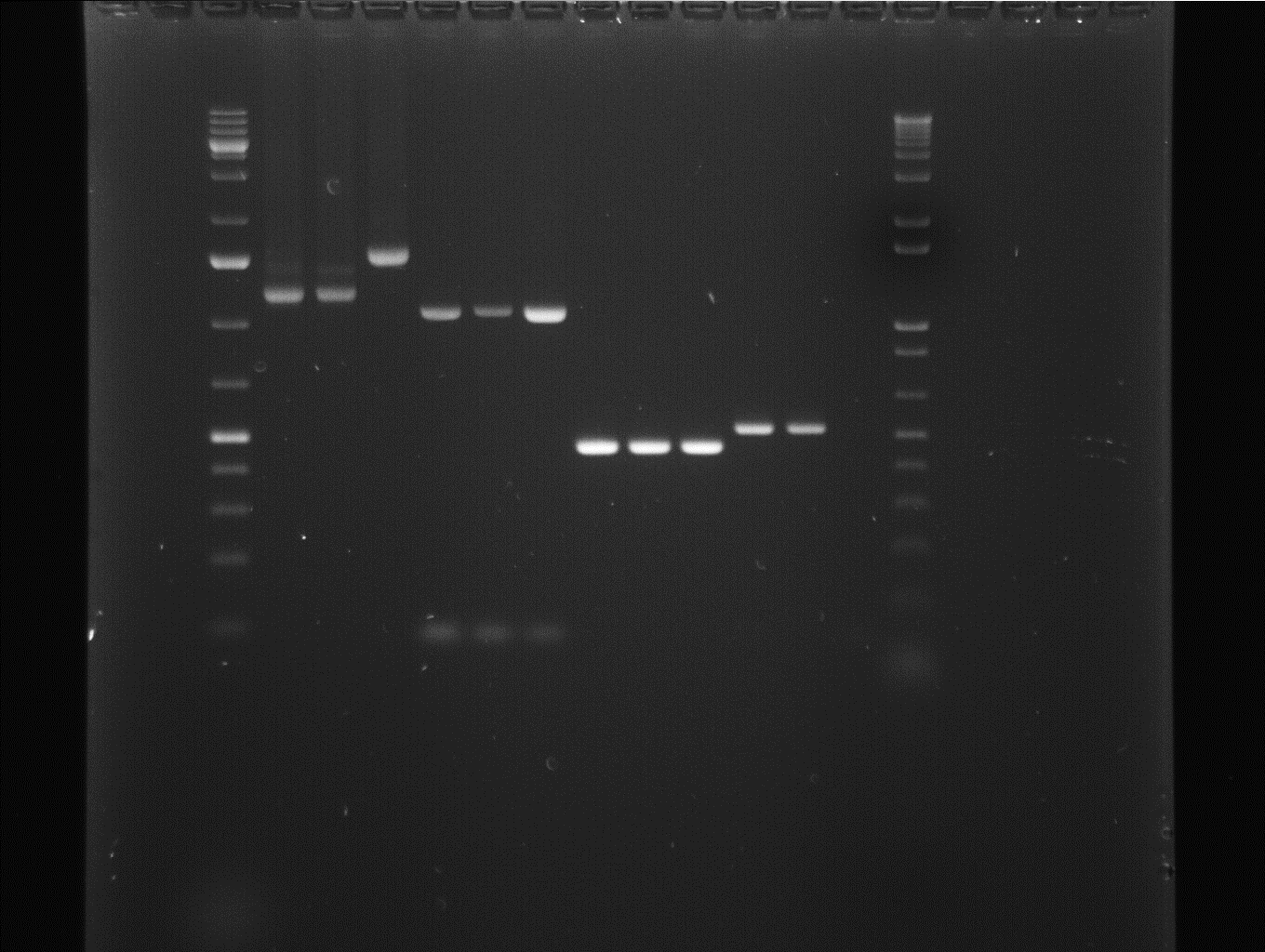
**

**Figure S27: Full uncropped version of Figure S22.**

**
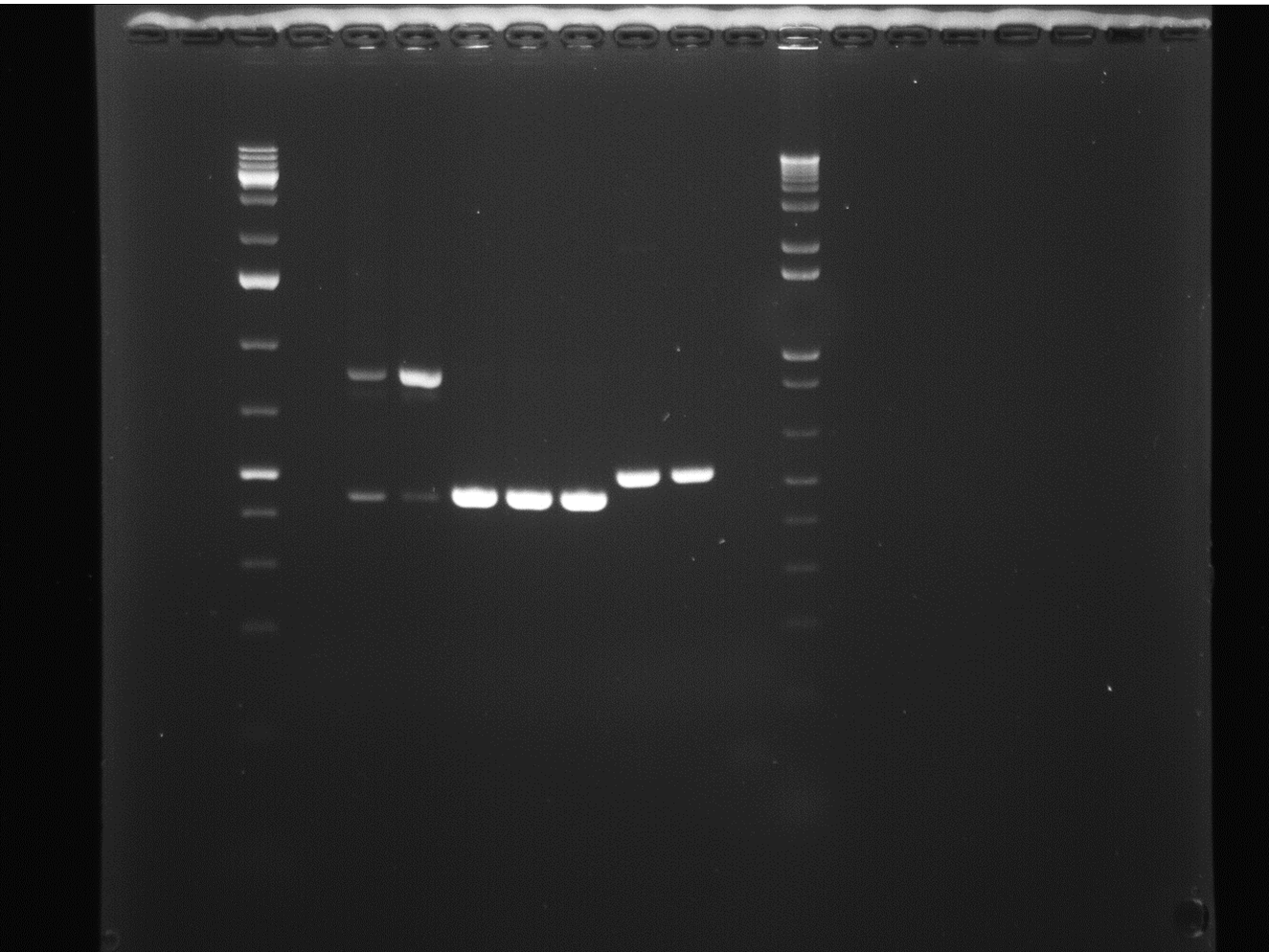
**

**Figure S28: Full uncropped version of Figure S25.**

**References**

1 FlyBase--the Drosophila database. The FlyBase Consortium. *Nucleic acids research* **22**, 3456-3458 (1994).

2 Sagri, E. *et al.* Housekeeping in Tephritid insects: the best gene choice for expression analyses in the medfly and the olive fly. *Scientific reports* **7**, 45634, doi:10.1038/srep45634 (2017).

3 Bailey, T. L. *et al.* MEME SUITE: tools for motif discovery and searching. *Nucleic acids research* **37**, W202-208, doi:10.1093/nar/gkp335 (2009).

4 Bailey, T. L. DREME: motif discovery in transcription factor ChIP-seq data. *Bioinformatics (Oxford, England)* **27**, 1653-1659, doi:10.1093/bioinformatics/btr261 (2011).

5 Elkan, T. L. B. a. C. in *Proceedings of the Second International Conference on Intelligent Systems for Molecular Biology.* 28-36 (AAAI Press).

6 Bailey, T. L. & Machanick, P. Inferring direct DNA binding from ChIP-seq. *Nucleic acids research* **40**, e128, doi:10.1093/nar/gks433 (2012).

7 De Renzis, S., Elemento, O., Tavazoie, S. & Wieschaus, E. F. Unmasking activation of the zygotic genome using chromosomal deletions in the Drosophila embryo. *PLoS biology* **5**, e117, doi:10.1371/journal.pbio.0050117 (2007).

8 Meccariello, A. *et al.* Maleness-on-the-Y (MoY) orchestrates male sex determination in major agricultural fruit fly pests. *Science (New York, N.Y.)*, doi:10.1126/science.aax1318 (2019).

9 Lagos, D., Ruiz, M. F., Sanchez, L. & Komitopoulou, K. Isolation and characterization of the Bactrocera oleae genes orthologous to the sex determining Sex-lethal and doublesex genes of Drosophila melanogaster. *Gene* **348**, 111-121, doi:10.1016/j.gene.2004.12.053 (2005).
