# Supplementary material for: Nanopore long-read RNA-seq and absolute quantification delineate transcription dynamics in early embryo development of an insect pest
Source: Sci Rep. 2021 Apr 12;11:7878. doi: 10.1038/s41598-021-86753-7 (PMC8042104; doi:10.1038/s41598-021-86753-7)
Supplement: Supplementary file 8 — Supplementary Information 8. [file 41598_2021_86753_MOESM8_ESM.pdf]

Nanopore long-read RNA-seq and absolute quantification delineate transcription dynamics in early embryo development of an insect pest

Anthony Bayega, Spyros Oikonomopoulos, Maria-Eleni Gregoriou, Konstantina T Tsoumani, Antonis Giakountis, Yu Chang Wang, Kostas D Mathiopoulos, Jiannis Ragoussis

1 Materials and methods

1 Sample processing and sequencing

1.1 Olive fruit fly breeding

The olive fly (*Bactrocera. oleae*) 'Demokritos' strain, that is considered in this study, has been maintained in our laboratory for over 15 years. It was originally sourced from the Nuclear Research Centre in Athens, Greece. No wild flies have been added since then, hence the strain has maintained a genetic uniformity. Olive flies were reared in appropriate holding cages at 25 ± 1 °C, 60 ± 10 % relative humidity and 14 L: 10D cycles according to the conditions previously[1].

1.2 Embryo collection, RNA extraction and quality control

In order to explore the transcriptome landscape of the developing embryo *B. oleae* individuals from an inbred isofemale line, were mated with males and then monitored to observe egg laying. Once the eggs were laid the eggs were incubated at room temperature for 1, 2, 3, 4, 5, and 6 hours, respectively followed by RNA extraction using Trizol method (Extended Figure 1). Briefly, the eggs were counted (see Table S2 for number of eggs used per timepoint) and then homogenized in TRI Reagent® (Sigma-Aldrich) and allowed to stand for 5 minutes at room temperature for complete dissociation of nucleoprotein complexes. The samples were then centrifuged at 12,000x g for 15 minutes at 4 °C. The aqueous phase was transferred to a new tube and 0.1 mL of 1-bromo-3-chloropropane (BCP) or 0.2 mL of chloroform per mL of TRI Reagent® added. The sample tubes were covered and shaken vigorously for 15 seconds, and then allowed to stand for 2 – 15 minutes at room temperature.

The resulting mixture was centrifuged at 12,000x g for 15 minutes at 4 °C to separate the mixture into 3 phases: a red organic phase (containing protein), an interphase (containing DNA), and a colorless upper aqueous phase (containing RNA). The aqueous phase was transferred to a fresh tube and 0.5 mL of 2-propanol per mL of TRI Reagent used in sample preparation (above) and mixed. Following a 5 – 10 minutes at room temperature incubation, samples were centrifuged at 12,000x g for 10 minutes at 4 °C to collect the RNA precipitate. The pellet was washed by adding

a minimum of 1 ml of 75% ethanol per 1 ml of TRI Reagent® used in sample preparation followed by 5 – 10 minutes air-drying. The pellet was finally resuspended in TE buffer for storage.

The quantity of the extracted RNA was determined using a Qubit RNA HS Assay Kit (Thermo Fischer Scientific, Q32852). The quality of the isolated RNA was assessed using an Agilent TapeStation instrument and Agilent RNA ScreenTape kit as per manufacturer's instruction (see Supplementary protocol). The profile of all the total RNA samples showed a single peak at ~2 kb which contrasts with mammalian total RNA profiles where 2 peaks at ~2 kb and 6 kb representing 18S and 28S ribosomal RNA, respectively (Extended Figure 2). This, however, is expected for most insects whose 28S rRNA contains a weak hydrogen bonds that easily denatures to release 2 similar sized fragments that run together with the 18S rRNA[2,3]. We, therefore, did not consider the RIN of the RNA samples.

1.3 ERCC Spike-in addition and cDNA synthesis

For each of the bulk samples, 300 ng of total RNA was used for the cDNA synthesis protocol. ERCC Spike-in Mix 1 (Thermo Fischer Scientific, 4456740) were added to the cDNA synthesis master mix (See Supplementary protocol). Our customized and published full-length cDNA synthesis protocol[4] is based on the highly sensitive Smart-seq2 protocol[5], which uses template switching and preamplification. It utilizes a combination of custom reagents and kits and is similar to the methodology tailored to long read sequencing we recently published[6] (See Supplementary protocol for the full step-by-step protocol).

1.3.1 Isoform validating PCR

In order to validate some of the isoforms for sex determining genes identified using RNA-seq, PCR amplification of regions of different transcripts was performed using primers listed in Table S13. The LongAmp® Taq PCR Kit (New England Biolabs, USA) was used. Briefly, 50 ng of cDNA generated in this study as already explained above was used as template in a 12.5 µl reaction containing 4 µM of each of the forward and reverse primers and 1X of the LongAmp PCR mastermix. The PCR cycles included initial denaturation at 94 °C for 2 minutes followed by 35

cycles of denaturation at 94 °C for 1 minute, annealing at 58 °C for 1 minute and extension at 72 °C for 6 minutes. A final extension at 72 °C for 10 minutes was performed and products kept at 12 °C. From PCR products 5 µl were then resolved on a 1.5 % agarose gel run in 1X TBE buffer for one hour at 150 Volts. Gel staining was by SYBR® Safe DNA Gel Stain (Invitrogen).

1.3.2 Quantitative real-time PCR (qRT-PCR or qPCR)

In order to determine the expression profiles of the serendipity alpha (*sry-a*) and head involuted defective (*hid*) genes in early embryo developmental stages a Real-Time PCR (RT-PCR) approach was used. First, embryos were collected in different timepoints as it is described above followed by RNA extraction using Extrazol reagent based on manufacturer's instructions. DNAse treatment was then performed to remove the residual DNA from the samples using Turbo DNase (Thermo Fisher Scientific) according to the manufacturer's instructions. The total amount of the DNA-free RNA was converted to cDNA using the PrimeScript RT Reagent Kit (Takara Bio) according to the manufacturer's instructions. The resulting cDNA was used in the subsequent qRT-PCRs reactions.

The total volume of the qRT-PCRs reaction was 15 µl consisted of 5 µl from a dilution 1:10 of the cDNA template, 2X Kapa SYRB fast qPCR Master Mix (KAPA Biosystem) and 300 nM of each primer. The qRT-PCR conditions were: DNA denaturation step at 95 °C for 3 min, followed by 40 cycles of denaturation at 95 °C for 10 s, annealing/extension, and plate read at 56 °C for 20 s, extension at 72 °C for 1 s and finally, a step of melting curve analysis at a gradual increase of temperature over the range 55 °C → 95 °C. The reactions were carried out on Bio-Rad Real-Time thermal cycler CFX96 (Bio-Rad, Hercules, CA, USA) and data were analyzed using the CFX Manager™ software. All qRT-PCRs were performed in duplicate biological replicates (1 biological replicate consisted of 10 eggs). Expression values for the *sry-a* and *hid* genes were calculated relatively to the housekeeping genes, ribosomal protein L19 (*RPL19*, Figure 3 C and D) and *14-3-3zeta* (Figure S7). All primers used in qRT-PCR (for *sry-a*, *hid*, *RPL19*, and *14-3-3zeta*) were previously reported in Sagri et al.[7,8] (see Table S13 for sequences).

1.4 ONT Library synthesis, sequencing, and quality assessment of reads

Sequencing libraries (1D) were prepared using the oxford Nanopore Technologies (ONT) SQK-LSK108 protocol. Sequencing was performed using on the ONT MinION Mk1b sequencer and run by MinKNOW version (1.10.16). Flow cells used were R9.4 version and the 1D library protocol. Due to known PCR biases that result from PCR amplification, we opted to use 12 cycles after performing cycle optimization and noticing that 12 cycles had negligible effect on cDNA profile (Extended Figure 3). For offline base-calling, Albacore version 2.0.2 was used. Quality analysis of the sequencing and base-called reads was performed using MinIONQC[9] and Pauvre (https://github.com/conchoecia/pauvre) (See Extended Figure 4 - Extended Figure 7 for QC results).

1.5 Illumina library preparation and sequencing

The Illumina Trueseq stranded mRNA sample preparation protocol was used to generate libraries for all the embryo and adult samples. Briefly, one microgram of total RNA was used to extract poly-adenylated transcripts using oligo dT Dynabeads (Invitrogen, USA). Purified RNA was fragmented. First and second strand cDNA synthesis was performed using SuperScript II. Following adenylation of 3’ ends, adapters were ligated and the fragments enriched by PCR using the following cycles: 98 °C for 30 seconds, 15 cycles of (98 °C for 10 seconds, 60 °C for 30 seconds, 72 °C for 30 seconds), and a final extension at 72 °C for 5 minutes. PCR products were purified using AMPure XP beads (Beckman Coulter). The quality and concentration of cDNA libraries was checked using BioAnalyser DNA-1000 chips (Agilent, USA), and qPCR, respectively. The samples were sequenced on Illumina HiSeq2500 following a 100 bp paired-end sequencing protocol.

2 Data processing

For all the tools used, the examples of parameters used and versions of tools are included in the Supplementary protocol.

2.1 The current *B. oleae* genome assembly and genome annotation

The olive fruit fly genome is diploid, consisting of six pairs of chromosomes which include a pair of heterochromatic sex chromosomes with the male being the heterogametic sex[10]. We recently submitted to NCBI the *B. oleae* genome assembled from short-reads followed by long-read scaffolding[11]. This assembly (GenBank accession GCA_001188975.2) has a total size of 471,780,370 bases and is contained in 36,198 scaffolds, with a contig N50 of 135,231 bases. This genome was annotated using the NCBI Eukaryotic Genome Annotation Pipeline yielding the *Bactrocera oleae* Annotation Release 100. The *Bactrocera oleae* Annotation Release 100 contains a total of 13,936 genes and pseudogenes of which 13198, 392, and 346, are predicted to be protein-coding, non-coding, and pseudogenes, respectively. Further, 2,759 genes are predicted to have variants (isoforms). In total, the *B. oleae* genome was predicted to contain 19,694 transcripts of which 18,702 are mRNA (with CDSs), 411 tRNA, 393 lncRNAs, and 188 miscellaneous RNAs. Whereas the mean length of the genes and transcripts is 9,597 bp and 2,259 bp, respectively, the longest gene is 497,921 bp while the longest transcript is 59,475 bp. All alignments and genes reported in this article refer to these resources: genome GCA_001188975.2 and annotation NCBI *Bactrocera oleae* Annotation Release 100, unless otherwise stated. Because only 14,555 mRNA (with CDSs) out of the 18,702 in the NCBI annotation (77.8 %) were assigned a gene product corresponding to their respective *D. melanogaster* homologue, we obtained the NCBI predicted proteins and re-determined the UniProt homologues to *D. melanogaster*. We assigned a *D. melanogaster* homologue to 12,494 (95 %) out of the 13,198 protein coding genes (Table S1, E-value $\leq$ 1e-3). Of these, 57 % were identified in the UniProtKB/Swiss-Prot database, which comprises high quality manually annotated and nonredundant proteins, while the remaining 43 % were identified in the UniProtKB/TrEMBL database which contains high quality computationally annotated and classified proteins.

2.2 Illumina data analysis

Following sequencing, quality control metrics were generated using FastQC (https://www.bioinformatics.babraham.ac.uk/projects/fastqc/). Reads were trimmed using Cutadapt[12] followed by Trimmomatic[13] processing. Using the NCBI published genome

(GCA_001188975.2) and the associated NCBI annotation, alignments to the genome where performed using HISAT2[14] while quantification of gene expression was performed using RSEM[15]. The Transcripts Per Million (TPM) quantification of RSEM was used. ERCCs were used as described in the ONT data analysis to transform relative TPM quantification into absolute number of transcripts per embryo. The statistics of the sequenced reads are included in Supplementary Table S3 while the gene expressions are provided in Additional file 1.

2.3 ONT Data processing

2.3.1 Sequencing Quality Control

The general data processing workflow is shown in Extended Figure 4. Between 3 – 5.5 million reads were generated per sample with the 1-hour timepoint (Bo.E.1H) having the least (3.6 million) and the 5-hour timepoint having the most (5.39 million, Table S3). Albacore, the ONT basecaller, classifies reads as "Pass" or "Fail" depending on a quality score (Q-score, equivalent to Phred score) greater or less than 7, respectively. In all timepoints 80 – 90 % of reads were classified as "Pass" and were used for further analysis (Extended Figure 5). The total number of bases ranged from 4.8 Gb – 5.8 Gb, of which > 90% belonged to reads classified as "Pass" (Extended Figure 6). Reads classified as "Fail", that is, having a phred score of <7 were not used in our analysis. The mean Q-score among pass reads decreased graduated over the 48-hour sequencing period while among fail reads mean Q-score was either variable or constant except for 3- and 6-hour timepoints (Extended Figure 7 B). The mean read length was constant over the 48-hour sequencing period regardless of the read Q-score (Extended Figure 7 B).

2.4 Genome guided *de novo* transcriptome assembly

2.4.1 Comparison of long-read transcriptome assembly tools

The *de novo* transcriptome assembly workflow is shown in Figure S1. We generated 31 million reads. Due to a median error rate of 16 % in the raw ONT reads we performed two orthogonal rounds of error correction; consensus self-alignment using Canu[16] and short-read correction using Lordec[17], which together generated 22 million reads (71 %). We then focused only on full-length

reads identified as those that possessed both 5' adapters and both poly(A) and 3' adapter. These were then pre-processed to orient the strands and trim adapters. First, we sought to compare 3 genome-guided *de novo* transcriptome assembly tools; TAMA[18], Cupcake ToFU[19], and TAPIS[20], on computation efficiency (memory and runtime), specificity, and precision. We used two tools currently available to analyze long-read *de novo* transcriptomes; PRAPI[21] and SQANTI[22]. The transcriptome assemblies generated from each tool were evaluated using SQANTI, and their respective annotation files were compared to the NCBI annotation file using Cuffcompare[23]. We used 3.07 million reads (3.29 Gb) taken from the *B. oleae* embryo sample collected at 5 hours post oviposition. We also used the NCBI genome assembly (GenBank accession GCA_001188975.2) and NCBI *Bactrocera oleae* Annotation Release 100 as references.

Regarding computational resources, we compared ToFU and TAMA directly since they involve similar operations. Runs were performed using our Cluster computing system; CentOS6, Linux x86-64 with one processor. Cupcake ToFU was more computationally efficient requiring 199.87 minutes wall time (199.81 CPU time) and 12.9 Gb at peak memory compared to TAMA's 6854.9 minutes wall time (6853.5 CPU time) and 226.3 Gb at peak memory to assemble the 3.07 million long-reads. TAPIS required 1046.3 minutes wall time (1097.5 CPU time) and 8.3 Gb at peak memory although TAPIS was run with an option to plot figures which extended run time.

Comparison of annotation files generated by each tool to the NCBI annotation revealed that TAMA had the highest sensitivity (87.5 %, capturing the most among reference transcripts) followed by ToFU at 82.9 %. TAMA also had the lowest precision suggestive of novel features although these can also be artifacts. We filtered ToFU transcripts to retain only transcripts with at least 2 supporting reads, an option that is not available for the other tools tested which might explain the higher precision. The high computational burden of TAMA and low precision of TAPIS discouraged us from continuing with them for generating the *B. oleae* whole transcriptome. Besides, ToFU allows to filter isoforms based on number of supporting reads and has good computational efficiency. We therefore used ToFU for our final assembly.

2.4.2 Comparison of genome guided *de novo* transcriptome assemblies form Illumina short-read versus ONT long-read RNA-seq

We sought to compare ToFU *de novo* assembly to an assembly generated from short reads. To control for differences in number of reads and bases generated between short-read and long-read technologies we plotted rare fraction curves to determine the number of reads and bases required to detect the same number of genes between ONT and Illumina. From the rare fraction curves, we estimated that for identification of the same number of genes about 40 times more reads and 8 times more bases were required from Illumina short-read cDNA-Seq compared to ONT long-read cDNA-Seq (Extended Figure 9). Here, a gene is considered identified if 90% of its length is covered by at least one read. We thus used 3.07 million ONT reads (3.29 Gb) and 95.4 million Illumina reads (18.6 Gb).

The Tuxedo genome guided protocol[23] which uses Cufflinks was used to derive the short-read assembly. The Cufflinks and ToFU (long-read) assemblies were compared to the NCBI assembly (Table E1). Both assemblies showed comparable sensitivity at base level (83.3 % versus 82.9 %, respectively). However, the long-read assembly showed slightly higher sensitivity at all other levels evaluated. The long-read assembly also showed higher precision at base level (74.9 % versus 65 %), although the short-read assembly had higher precision at other levels evaluated except at locus level. We also analysed the transcriptomes generated using SQANTI (Table E2). Long-reads generated a richer transcriptome containing 43,676 transcripts compared to 21,840 generated from short-reads. However, short-read transcripts overlapped 8239 annotated genes compared to 6776 genes overlapped by transcript generated from long-reads. Short-read transcriptome contained 8712 novel genes, a number which seemed exaggerated in our experience. Long-reads transcriptome contained 2060 novel genes which was reasonable to us. Long-reads transcriptome however, contained a higher number (14436) and percentage (33 %) of transcripts that fully matched an annotated transcript compared to Cufflinks (5962, 27 %, respectively). Interestingly, 80 % of genes in the short-read transcriptome had one isoform compared to only 25 % in the long-read assembly. This shows the power of long-read transcriptome assembly in identifying the full range of splicing patterns and the weakness of short-read RNA-seq in assembling transcriptomes as has been noted by others in the field. Noteworthy, although we used 3.07 million ONT reads,

only 1.98 million reads were used to generate the assembly while the rest were filtered out due to low identity, coverage or being unmapped.

2.4.3 *Bactrocera oleae* genome guided long-read transcriptome assembly

Although the olive fly has been annotated by NCBI (*Bactrocera oleae* Annotation Release 100) the annotation was based mainly on computational predictions and support from short-read data. We, therefore, sought to provide an assembly based on long reads which would confirm the NCBI predictions, improve on the annotations, and add missing genes, and particularly missing isoforms. We included reads generated from adult female and male heads and sequenced following the same protocol described above. All reads were provided to Canu[16] to perform consensus error correction. Of the 25.7 million total reads generated across 6 timepoints 18.5 million reads (72 %) were error-corrected which improved alignment identity from ~87 % to ~96 % (Extended Figure 14). Because we added different adapters at each end of each original cDNA molecule, and because the current MinION performs sequencing randomly starting from either 3' end of each molecule we used a customized version of Mandalorion to return the correct original strand of each molecule based on detection of the 5' and 3' adapters. Only full-length reads were used in transcriptome assembly (9.8 million reads). Full-length reads where identified as those that possessed the both 5' adapters and both poly(A) and 3' adapter. Using this filtering, about 50 % of the reads were classified as full-length (Extended Figure 8). The adapters were trimmed using Porechop. Since we had generated short-read Illumina reads for the all timepoints, we used Lordec to perform hybrid error correction which further improved the alignment identity from ~96 % to ~98 %. Another customized version of Mandalorion was used to perform a final round of adapter trimming. Reads that had not been error corrected using Canu were taken through a similar pre-processing described above and combined with the error-corrected reads. The pre-processed reads were aligned to the genome using GMAP.

We used Cupcake ToFU for transcriptome assembly. ToFU provided adequate user options and reasonable running speed. ToFU was used to collapse the transcripts into a non-redundant set of transcripts comprising the genes and their associated isoforms. SQANTI[22] was then used to

analyze the transcripts, identify novel genes, and perform open reading frame prediction using the GeneMarkS algorithm. Because ToFU was set to consider reads that were aligned at least 99 % in length and with at least 95 % identity, out of the total 14.7 million reads, 3.9 million were used to derive the transcriptome. See Extended Figure 11 for a summary of the SQANTI output.

The ToFU collapsed transcripts contained a total of 10,840 genes and 79,810 isoforms. Of the genes, 9072 matched the NCBI annotated genes while 1768 genes were novel. Over 50 % of novel genes were mono-exon compared to annotated genes where over 80 % of the genes were multi-exon (Extended Figure 11). Novel genes also showed lower expression compared to annotated genes (Extended Figure 11). Structurally, SQANTI categorises the transcripts into 9 groups depending on their splice junction and genomic coordinate including; full splice match (FSM), incomplete splice match (ISM), novel in catalogue (NIC), novel not in catalogue (NNC), genic genomic, antisense, fusion, intergenic, genic intron. Most of ToFU collapsed transcripts were a perfect splice match to the annotated transcripts (32.9 %), followed by transcripts containing alternatively spliced junctions (17.4 %, Extended Figure 11). The length distribution of transcripts across the different structural categories was largely similar (Extended Figure 11). There was however, difference in expression level across the structural categories; ISM, NIC, NNC, and fusion transcripts had the lowest expression as measured with short-read RNA-Seq (Extended Figure 11). Regarding splice junctions across structural categories, the antisense transcripts had the highest number of non-canonical splice junctions (Extended Figure 11).

About 274435 of the reads (1.9%) that passed pre-processing either did not align to the NCBI genome or aligned with less than 51% of their length. These reads were aligned to our recently improved *B. oleae* genome assembly that was generated using 10X Genomics Linked-reads technology followed by mate-pair and long-read scaffolding and gap-closing. This assembly (GenBank accession GCA_001188975.4[11]) is more contiguous than GCA_001188975.2 but is still undergoing annotation at NCBI. Of those unaligned reads, 32% aligned with ≥50% coverage. Cupcake ToFU and SQANTI were also run on these aligned reads to further identify genes missing from the NCBI assembly. We identified another 228 genes missing in the NCBI genome but found within the GCA_001188975.4 assembly.

All NCBI predicted proteins and the novel identified ORFs were blasted against Uniprot *Drosophila melanogaster* Swiss-Prot and TrEMBL databases to identify *D. melanogaster* homologues. We updated the NCBI *B. oleae* annotation to include novel genes and thus created a new gene transfer file (GTF) termed Annotation v2 (Additional file 2 and 3). We also added the novel genes that resulted from the GCA_001188975.4 *B. oleae* genome assembly to the NCBI assembly (GCA_001188975.2) and created a new assembly termed NCBI v2. The Annotation v2 and assembly NCBI v2 were used in gene expression quantification and expression profiling. The full transcriptome generated was also analyzed using PRAPI[21]. The novel genes that are found in the GCA_001188975.4 assembly are provided separately as Additional file 4.

2.5 Identification of Cricket paralysis virus as a major threat to our fly colony

Out of the 274435 reads (1.9%) that did not align to the NCBI *B. oleae* genome assembly, 187073 reads (68%) also failed to align to our improved GCA_001188975.4 assembly. We, therefore, performed blastx against the Uniprot Swiss-prot database. We obtained a significant hit from 37 % of these un-aligned reads (E-value <= 1e-4). Interestingly, 86% of the hits were to Viruses and among Viruses, the genus Cripavirus of the order Picornvirales accounted for 87% of the hits. The type species for this genus is cricket paralysis virus (CrPV). Further digging into this category revealed that the blastx hits to the Cripavirus were only to 3 proteins; Structural polyprotein (65%, Uniprot ID P13418 from CrPV), Replicase polyprotein, ORF1 (24%, Uniprot ID Q9IJX4 from CrPV), and Replicase polyprotein, ORF1 (10%, Uniprot ID O36966 from Drosophila C virus).

Identifying a high number of reads corresponding the structural polyprotein of CrPV which is the precursor of all viral capsid proteins suggests that this virus was replicating within the flies we used. Cricket paralysis virus has been shown to infect and replicate in adult olive flies[24]. Feeding insects on solution containing CrPV resulted in 80% of the flies dying within 12 days. Further, Replicase polyprotein, ORF1 (CrPV-1A) is a known suppressor of RNA-mediated gene silencing, an antiviral defense mechanism of insect cells. CrPV-1A obstructs the initial target searching of

Ago2-RISC by binding to Argonaute protein which is the core of the RNA-induced silencing complex (RISC) in insects and thus suppress its target cleavage reaction[25].

The reminder of the reads (3285) that did not align to both our genomes and failed to return a Blast hit might represent genes that are unique to the olive fruit fly which are located in regions of the genome that are difficult to assemble.

2.6 Read alignment

The NCBI *B. oleae* assembly (accession code GCA_001188975.2) was used for the alignment. However, since we included ERCC in our cDNA the ERCC sequences were included in the NCBI assembly prior to alignment. Reads were preprocessed to make them stranded and remove adapters and poly(A) tail. All the reads were also supplied to Canu to perform consensus error correction (see Supplementary protocol for run parameters). Alignment of reads to the reference genome and transcriptome was performed using 2 splice-aware and long-read enabled aligners; GMAP[26] and Minimap2[27].

2.6.1 Comparison of GMAP and Minimap2 splice-ware long-read aligners

Alignment of reads generated from third generation long-read sequencing technologies provides unique challenges due to the length of the reads and the relatively high raw read error rates. Alignment of discontinuous reads for example cDNA further complicates the alignment. Currently, GMAP and Minimap2 are the most widely used long-read splice-aware aligners although others like GraphMap[28] support transcript alignment albeit through an annotation file which is used to reconstruct the transcriptome. Recently, Krizanovic et al.[29] evaluated long-read RNA-seq aligners and found GMAP to be the best aligner for long-read cDNA-Seq. However, Minimap2 lacked splice-aware alignment at the time and thus was not evaluated. We have used RNAseqEval[29] developed by Krizanovic et al., to directly compare GMAP and Minimap2.

Using our Cluster computing system; CentOS6, Linux x86-64 with 32 Gb of memory and 24 cores we aligned 1 million reads subsampled from the 5-hour timepoint to the *B. oleae* NCBI genome (471,863,126 bases, including ERCC sequences) using GMAP and Minimap2 with their default settings except for restriction of secondary alignments and setting the number of threads. Both tools involve an indexing step of the genome although this is optional for Minimap2. Regarding genome indexing, Minimap2 showed exceptional speed taking only 0.52 minutes (wall time) compared to GMAP's 7.06 minutes. Minimap2 indexing also showed better memory usage taking up only 345 Mb of memory and returning a single 1.3 gigabytes index whereas GMAP used 1.3 gigabytes of memory returning an index of ~ 2.5 Gigabytes distributed in 15 files. Regarding read alignment Minimap2 showed exceptional speed (for example aligning the 1 million reads in only 4.2 minutes (wall time) with 24 threads) compared to GMAP's 63.5 minutes (Extended Figure 10). Both tools however, showed comparable scaling with the number of processors (threads) used (Extended Figure 10). Running with just 1 thread during alignment, we noticed that GMAP used 1.4 Gigabytes at peak memory compared to Minimap2's 3.3 gigabytes. Minimap2 however, includes a '-I' option to adjust the number of reads loaded into memory and this could probably reduce memory usage. Indeed, Chu et al.[30] who evaluated several genomic DNA mapping tools observed that Minimap (a less advanced version of Minimap2) was the most computationally efficient, specific and sensitive method on ONT datasets tested.

Minimap2 was therefore our preferred method for evaluating alignment statistics due to its speed and higher accuracy. For genome guided *de novo* transcriptome assembly we used GMAP because the assembly tools required GMAP and because GMAP includes options to control alignments; for example --max-intronlength-ends which helps prevent spurious alignments. To determine the alignment statistics, all reads regardless of their adapter content were aligned to the reference using Minimap2. Both custom scripts and AlignQC[31] were used to compute alignment statistics. The read alignment rates reached 98%. Alignment of reads to the *B. oleae* NCBI predicted gene models showed 91% alignment rate. Addition of novel genes identified following our *de novo* assembly increased alignment rates to gene models from 91% to 97%. However, after filtering for good alignments the alignment rates across timepoints were ~90%.

We found AlignQC to provide extensive exploration of alignment statistics. Majority of reads aligned were single alignments whereas gapped and chimeric alignment were 0.11 % and 0.76 %, respectively (Extended Figure 12). The median percentage of bases aligned (84 %) was slightly lower than reads aligned. Exons accounted for most of the base alignments (68.5 %) followed by introns, genome, and intergenic at 17 %, 12.4 %, and 6.5 %, respectively. The genome and intergenic fraction probably indicating novel genes and/or DNA contamination in our RNA samples.

As noted elsewhere, long-read sequencing technologies exhibit relatively high error rates compared to short-read sequencing technologies. We observe a median of 16.8 % error rates in aligned segments of reads with deletions contributing the most (7.5%), followed by insertions (4.7 %), and mismatches (4.5 %). Canu correction reduced the error rates from ~16% to ~8% (Table S5).

2.7 Relative quantification of gene expression

Relative quantification of gene expression was performed using customized Mandalorion pipeline[32,33] with the NCBI annotations updated with novel genes (Annotation v2) and assembly NCBI v2 as references. Mandalorion counts the number of reads overlapping exon features of a gene and normalizes for sequencing depth and calculates the relative abundance as Reads Per Gene Per 10,000 aligned reads (RPG10K). There was high correlation between sequenced ERCC internal standards and the expected molecules (Extended Figure 13 A). This was however, expected as we and others have shown that ONT cDNA-Seq shows highly accurate quantification of gene expression[6,31]. We noted that although we added ERCC standards at a constant ratio per embryo across sample the relative normalization showed varying levels at different timepoints (Figure 2A, Figure S3), perhaps reflecting the varying amount of poly(A) RNA in the embryos across timepoints. This was expected and could be correct by absolute normalization.

2.8 Direct Absolute normalization of gene expression

The method and justification for absolute normalization have been previously reported by Owens et al.[34]. The method relies on the use of known transcript copy numbers for each ERCC internal spike-in RNA standards[35,36] and their corresponding relative expressions to derive a conversion factor. The conversion factor is derived from a generalized linear model with a dispersed Poisson likelihood using R statistical software as follows;

$$\text{glm(formula} = r_{qj} \sim \text{offset(log(Sq)), family = poisson(link = log))}$$

where:

r_{qj} is the relative abundance (RPG10K) of standard q in sample j

Sq is the known abundance (number of molecules /transcripts) of standard q

The intercept coefficient from the above function is the conversion factor used to convert RPG10K to absolute quantification using the following formula;

$$m_{ij} = \rho_{ij} e^{-\beta j}$$

Where:

m_{ij} is the absolute abundance (number of molecules /transcripts) for gene i in sample j

ρ_{ij} is the relative abundance (RPG10K) of gene i in sample j

β_j is the conversion factor

The absolute gene abundances were normalized to the number of embryos used per timepoint to obtain the absolute transcripts per embryo (TPE). In contrast to the relative expression (Figure 2A), absolute normalization showed more constant abundance of internal ERCC standards across timepoints (Figure 2B, Figure S3B). When the absolute number of transcripts per embryo for all genes were summed and plotted across timepoints, the absolute normalized profile (Figure 2D) mirrored that of cDNA generated per embryo (Figure S4A), thus validating the absolute normalization approach. This was expected since an equal amount of ERCC standards were added per embryo to each sample. Absolute gene expression was also highly correlated between Illumina

short-read cDNA-Seq and ONT long-read cDNA-Seq both for ERCC and genes; Spearman r=0.94 and r=0.9, respectively (Extended Figure 13). As noted previously[34], ERCC-00116 performed poorly, deviating from its expected abundance, and was excluded from analysis. Further, as we had anticipated, the successive timepoints showed higher gene expression correlation than distant timepoints with Spearman correlation for successive samples consistently equal or above 0.96 (Figure S5) showing that our sampling was close enough to capture transcriptional dynamics across the sampling time.

2.9 Detection limits and mRNA content of the embryo

Detection limits were calculated as guided by the ERCC manufacturer (http://tools.thermofisher.com/content/sfs/manuals/cms_086340.pdf). For ONT long-read RNA-Seq we define the detection limit setting our sensitivity to RPG10K of 0.01 which corresponds to ~2 mapped reads. For Illumina short-read RNA-Seq the detection limit is defined by the number of transcripts per embryo required to produce 10 reads. Averaged over the 6 samples, the detection limit of our ONT data was 1,038 transcripts per embryo (Supplementary Table S9).

2.10 PCA captures transition through developmental time

To explore the expression profiles, we performed principal component analysis (PCA) and hierarchical clustering using the most variable genes. Projection of the expression onto the first two principle components showed temporal correlation of expression with successive time points recapitulating the developmental trajectory (Figure S6). The first principle component separated the first 3 timepoints from the last 3 timepoints. Hierarchical clustering showed similar results (Figure S7). We also downloaded *D. melanogaster* early embryo RNA-seq data (0-8 hours after fertilization, AF) from Flybase database[37] and projected them together to our *B. oleae* data. The first principle component showed that *D. melanogaster* embryos at 0-2 hours AF clustered with *B. oleae* 1-3 hours AEL timepoints. Similarly, the first principle component co-clustered *D. melanogaster* 2-8 hours AF and *B. oleae* 4-6 hours AEL (Figure S6). This suggested close similarity in gene expression patterns between these two dipteran insects whose evolutionary distance is more than 100 million years[38].

2.11 Dramatic re-organization of maternal transcripts

In many organisms, oocyte development is arrested during meiosis followed by deposition, into the oocyte, of maternally derived transcripts that represent a wide range of the proteome. Among the 13198 *B. oleae* protein coding genes, 62 % (8132) were detectable at 1 hour AEL (detection limit of 1110 transcripts per embryo). By far, the most abundant transcript in the 1-hour embryos was the mitochondrial encoded 16S ribosomal RNA which accounted for ~2.5 % of the total embryo mRNA content followed by mitochondrial encoded essential amino acids valine and leucine. The other 9 most abundant genes we could find *D. melanogaster* homologs for included the eukaryotic translation elongation factor 1 alpha 1 (*eEF1α1*), lipid storage droplet 2, ornithine decarboxylase antizyme (required to regulate polyamine synthesis), ribosomal protein L27A, cyclophilin 1 (required for intracellular signaling, transcription), histone H5, trailer hitch (required for oocyte dorsoventral patterning), thioredoxin peroxidase 1 (required to prevent oxidative damage), and ATP synthase subunit C. Since the zygotic genome is largely inactive during early development these highly abundant transcripts can be assumed to be maternally supplied. These results indicated that maternally deposited resources are highly geared towards macromolecular synthesis.

When examining the total mRNA content per embryo during development across timepoints we noted that the total mRNA per embryo dropped 51 % at 2 hours AEL compared to levels at 1-hour AEL and increased 143 % at 3 hours AEL compared to levels at 2 hours AEL. This profile mirrored that of cDNA generated; although our cDNA synthesis protocol used equal amounts of total RNA (300 ng) per time point, the amount of cDNA generated at 2 hours AEL was 2.3 times less than that derived at 1-hour AEL after adjusting for number of embryos used (Figure S4A). Further, the total RNA profile showed comparable quality among our samples, ruling out RNA degradation artifacts (see Extended figure 2 in supplementary material). Indeed, the dramatic drop in abundance of poly(A) transcripts observed at 2 hours AEL could be replicated in a different set of biological samples using qPCR which used a mixture of both poly(A) and random hexamers in the reverse transcription step (Figure S9).

The mechanism of dramatic downregulation of polyadenylated maternal transcripts seen at 2 hours AEL and the rebound seen at 3 hours AEL appeared to be systemic rather than targeted. This is because the number of expressed genes was similar between 1, 2 and 3 hours AEL (11000, 10847,

and 10232, respectively). We hypothesized that destabilized genes are enriched with highly expressed genes. Using our time-course data, we performed differential expression analysis between successive timepoints using GFOLD[39] which is designed for samples without biological replicates. GFOLD log2 fold change has been shown to correlate well with qPCR-determined fold change[40]. Genes were coded as upregulated or downregulated using a GFOLD log2 fold change cutoff of ±1. We identified 1,496 genes that were downregulated at 2 hours AEL compared to 1-hour AEL, here referred to as maternal-down genes. Indeed, at 1-hour AEL the expression level of maternal-down genes was significantly higher than that of the other genes (p-value < 2.2e-16, Figure S10B). Interestingly, at 2 hours AEL the abundance of maternal-down genes was comparable to other genes although still significantly different (p-value=4e-14, Figure S10C). It is likely that in some non-drosophilid flies, the initial phase of MZT includes a "normalization" process where the maternal genes are destabilized (as reflected by the abundance in poly(A) driven RNA-seq) in a process that mainly affects the most highly abundant genes, to bring their levels down to basal levels of other genes, again as far as poly(A) driven qPCR can determine. To further support this hypothesis, we sorted genes from the most downregulated to the least downregulated (as measured at 2 hours AEL compared to 1 hour AEL) and also determined their abundance at 3 hours AEL relative to their abundance at 1 hour AEL. Indeed, the least down-regulated genes were among the most up-regulated at 3 hours AEL (Figure S11).

2.12 Identification of DNA motifs important in early *B. oleae* embryo development

We obtained a DNA sequence 1000 bp upstream of annotated transcription start site for each of the gene groups (zygotic genes or maternal downregulated genes). These sequences were supplied to either CentriMo[41], TomTom[42], DREME[43], which are all part of MEME suite[4445] found at http://meme-suite.org. Gene ontology analysis was performed using the online version of Gene Ontology[46] found at http://www.geneontology.org. We found the motif CAGGTAB to be enriched among zygotic genes. To find other motifs among early zygotic genes that might be important in *B. oleae* embryo development, we used the absolute quantification data and picked all genes that were not detectable at 1 or 2 hours AEL but detectable at other time points which yielded 1132 genes (Supplementary Table S12). These genes should be enriched with zygotic genes. We used MEME to identify enriched motifs in promoter regions of these genes and identified the motif

RTATGTRTGTRTRTR as the top hit (E-value 2.0e-138, CAGGTAB was 3rd highest). Comparing this motif to a *Drosophila* database of known transcription factors and their binding motifs using the TomTom motif comparison tool[42] identified *topoisomerase II* (*Top2*) and *chorion factor 2* (*Cf2*) as the top hits (E-value/p-value 1.98e-2/1.4e-5 and 1.18e0/8.29e-4, respectively). *Top2* is essential for removing supercoils created during replication and transcription while *Cf2* is a zinc-finger transcription factor involved in follicle cell fate determination and regulation of myogenic gene expression. This motif was not enriched in a set of 1132 randomly picked maternal-down genes which were enriched in another motif (Figure S12) whose *Drosophila* top hit was adult enhancer factor 1 (Aef1).

2.13 Temporal clustering of developmental stage-specific genes

The expression matrix (transcripts per million, TPM) filtered for genes that were not expressed at any of the stages was used as input to DPGP[47] to cluster genes with similar expression profiles. Clusters of Genes in clusters that peak at either of the 4 metamorphotic stages; egg, larvae, pupae, adult were combined and used in gene ontology enrichment analysis using gProfiler[48].

2.14 Structure of genes involved in *B. oleae* early sex determination

Dipteran insects share a large part of their sex determination mechanism. In *D. melanogaster* where the sex determination mechanism has been extensively studied (reviewed in [49]), the *sex lethal* gene (*Sxl*) acts as the master regulator, mediating sex-specific alternative splicing of both itself and the *transformer* gene (*tra*) depending on the ratio of sex chromosomes to autosomes. *tra* together with *transformer2* (*tra2*) mediates sex-specific alternative splicing of *double sex* (*dsx*), the last member of the cascade and the mediator of differential sex development. *B. oleae* homologues of *Sxl*, *tra*, and *dsx* have all been identified although unlike *tra* and *dsx, Sxl* plays no role in *B. oleae* sex determination[50,51]. The temporal expression profile of these genes during early embryo development was previously investigated in the closely related *Ceratitis capitata* albeit using semi-quantitative PCR[52]. As previously described in *C. capitata*, we also observed that *Sxl*, *tra, tra2* and *dsx* are maternally inherited, bearing the default female-specific splicing pattern (Supplementary Figure S17 - Supplementary Figure S22).

The structure of *BoMoY* and *dsx* are described in the main manuscript. The *tra* gene model we observed differs from the model proposed by Gabrieli et al.[52] and largely agrees with the model proposed by Lagos et al.[50], although we change the naming of exons to show that *B. oleae tra* contains 6 exons with exon 3 being the male-specific exon (Figure S19 and Figure S20). The first male-specific splice pattern of *tra* was observed at 5 hours AEL coinciding with *BoMoY* expression (Figure S19). Gabrieli et al., also observed that in *C. capitata*, *tra* transcript splice variants start appearing at 5 hours AEL although the female-specific variant is not cleared in the males until 8 hours AEL[52]. As previously reported, we did not see alternative splicing of *tra2*. The mature and maternally supplied isoform of *tra2* was observed all through our 6 timepoints (Figure S21). *tra* and *tra2* showed abundant expression in the first 6 hours AEL (Figure 5).

Supplementary Figures

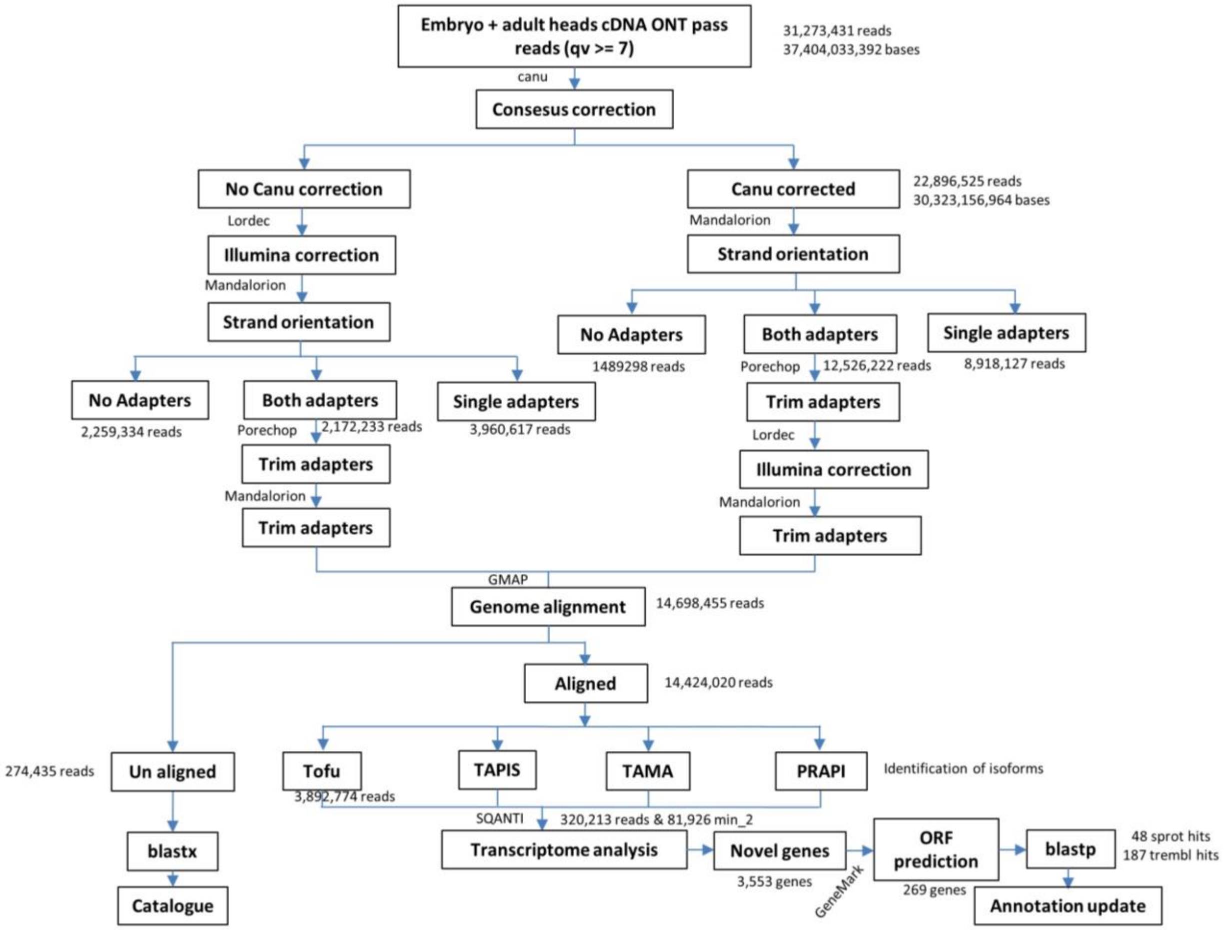

Figure S1. Long-read genome guided *de novo* transcriptome assembly workflow. All reads were provided to Canu to perform consensus error correction. A customized version of Mandalorion was used to return the correct original strand of each read based on detection of the 5' and 3' adapters. Only reads with both 5' and 3' adapters detected were used in transcriptome assembly. The adapters were trimmed using Porechop. Short-read Illumina reads were used to perform hybrid error correction with Lordec. Another customized version of Mandalorion was used to perform a final round of adapter trimming. Reads that had not been error corrected using Canu were taken through a similar pre-processing described above and combined with the error-corrected reads. The pre-processed reads were aligned to the genome using GMAP. ToFU was used to derive the final transcriptome assembly, followed by transcriptome analysis using SQANTI and PRAPI. TAMA, and TAPIS were also evaluated for transcriptome assembly.

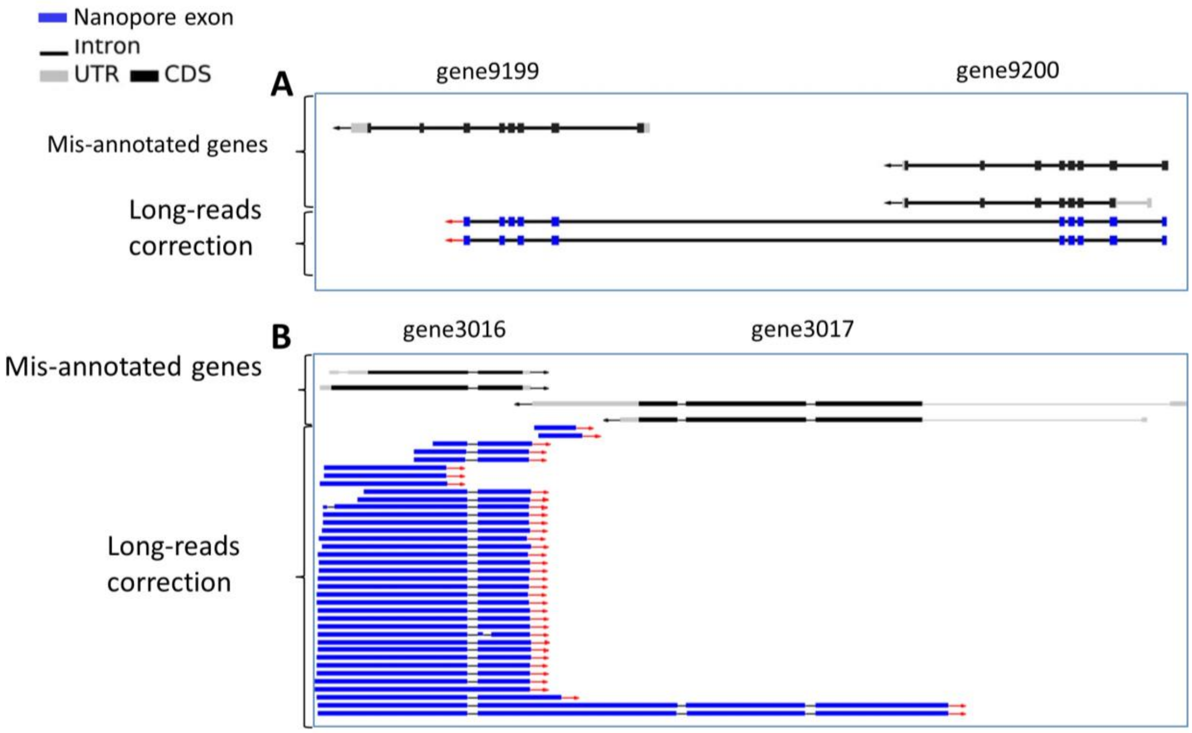

Figure S2. Long-read RNA-seq corrects mistakes in computationally predicted gene annotations. PRAPI was used to align transcripts to the NCBI predicted gene models and identify miss-annotated genes. Miss-annotated genes are described as single genes that were wrongly predicted as 2 or 3 separate genes. Two examples of such genes are shown here (**A** and **B**). The miss-annotated isoforms are shown on top while the long-read alignments are shown at the bottom of each panel. Full-length cDNA sequencing can provide evidence showing that miss-annotated genes are one single gene. See Table S8 for a full list of corrected genes.

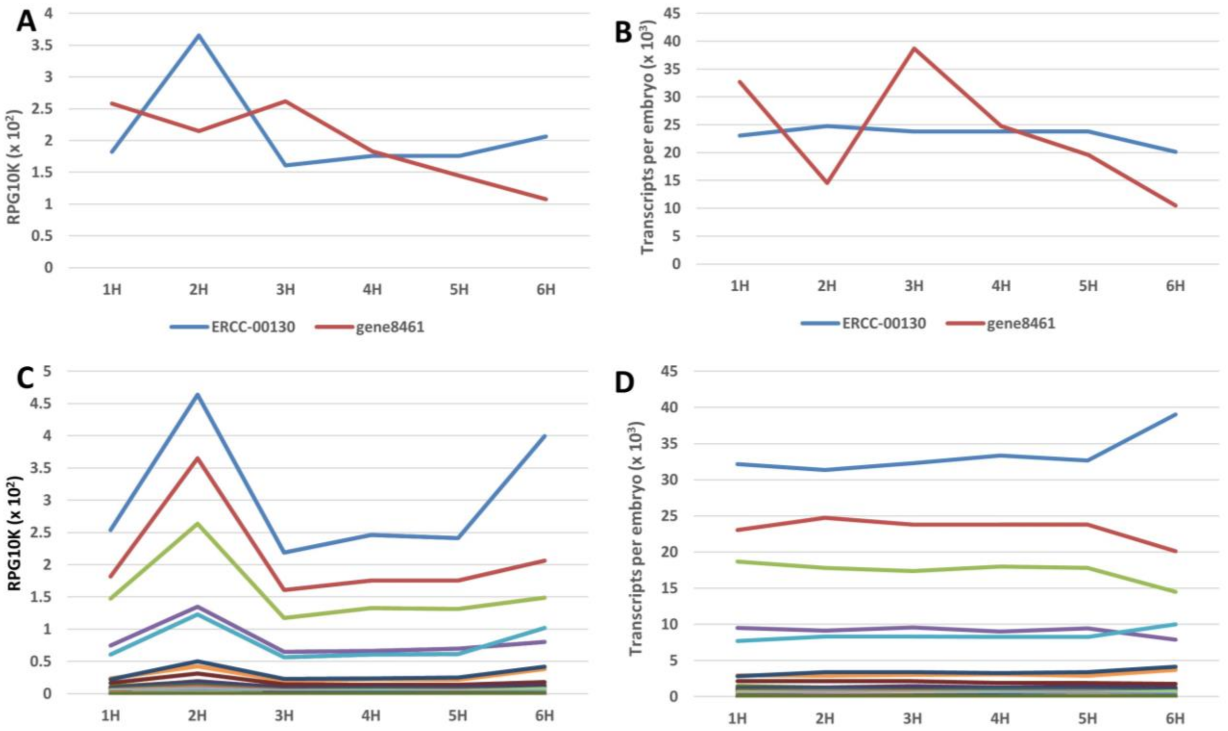

Figure S3. Comparison of relative normalization and absolute normalization of ERCC internal standards. A) Relative normalization of the most abundant ERCC (ERCC00130, blue) and a randomly picked gene (gene8461, red). Abundances of ERCC00130 varied with time (H=hours AEL) most likely due to changes in the amount of poly(A) RNA in the embryo. **B)** Same as A but showing absolute normalization of the most abundant ERCC (ERCC00130, blue) and a randomly picked gene (gene8461, red). Here, the abundance of the ERCC is stabilized across timepoints. **C)** Same as A but including all ERCC internal controls and excluding gene8461. **D)** Same as C but showing absolute normalization.

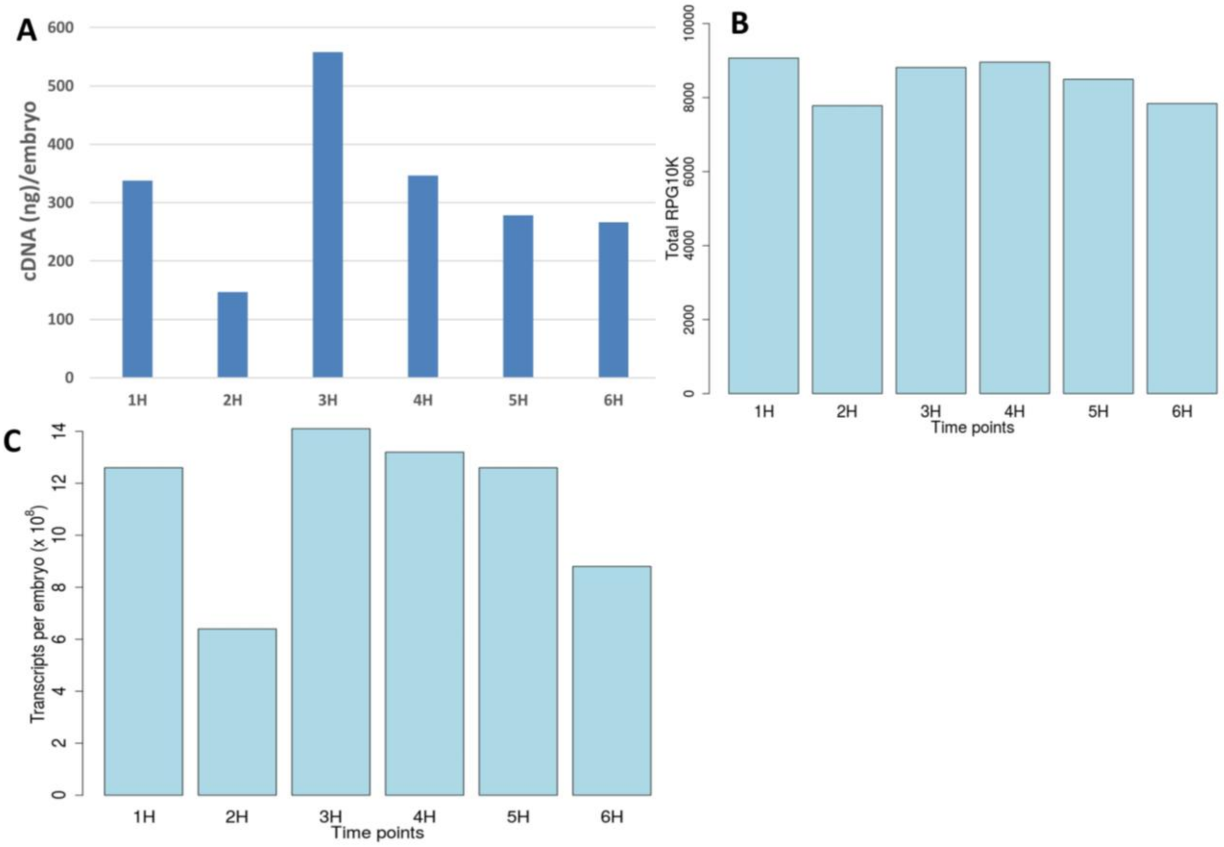

Figure S4. Comparison of relative normalization and absolute normalization of genes. **A)** Amount of synthesized cDNA per embryo at different timepoints (H; hours AEL). Equal amount of total RNA was used during cDNA synthesis. The amplified cDNA generated was purified and normalized to the number of embryos used at each timepoint. **B)** Summed expression values for all genes across timepoints. The relative method of quantifying gene expression was used. Here, read counts aligning to a gene are normalized by the total reads aligned to all other genes and further normalized to 10000 reads (RPG10K). This profile does not closely resemble the total cDNA profile in A. **C)** Same as B but using the absolute normalization and normalizing for the number of embryos. This profile shows close resemblance to the cDNA profile, demonstrating the advantage of absolute normalization.

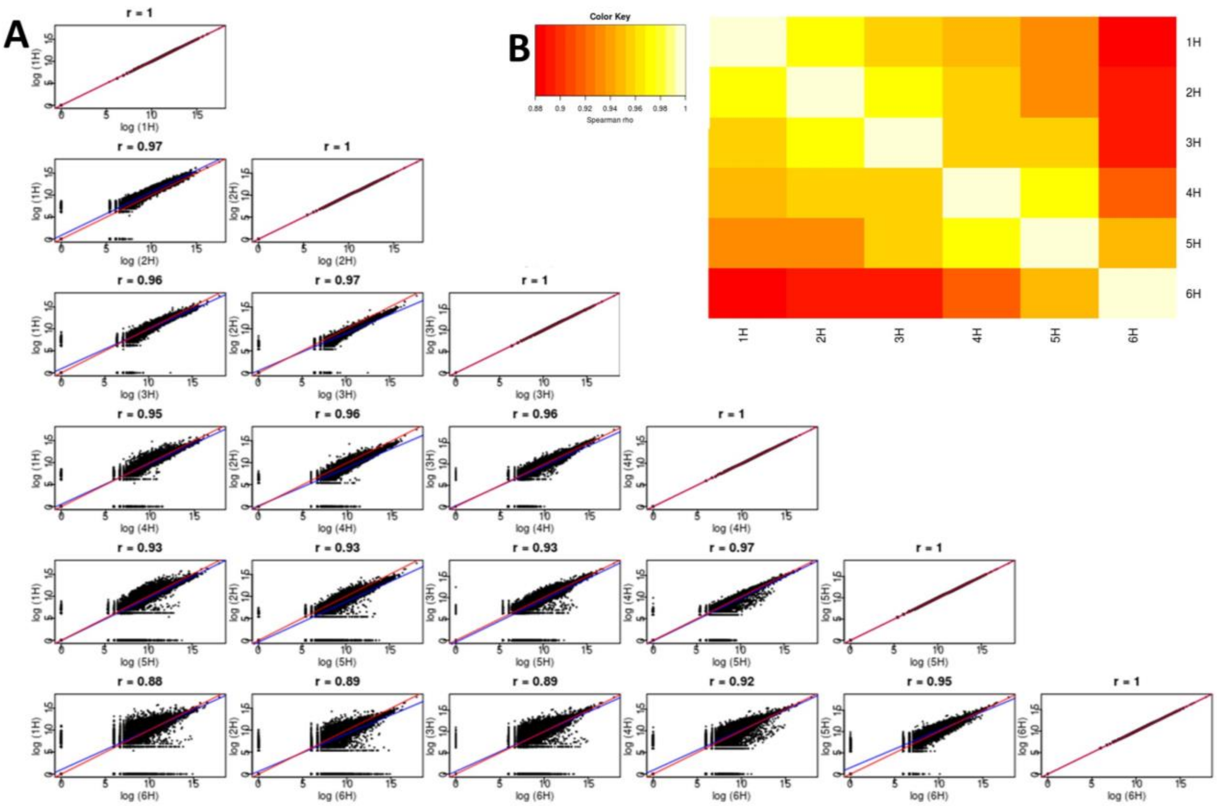

Figure S5. Correlation of gene expression across time-points. **A)** The Spearman rho (r) correlation of all expressed genes for each timepoint with itself and other timepoints is shown. Correlation was determined for the log absolute expression values. The plots are fitted with linear model (blue) and arbitrary line with intercept set at 0 and slope of 1 (red). **B)** Heatmap of the Spearman correlations from (A). Sample labels refer to the time (hours) after egg laying.

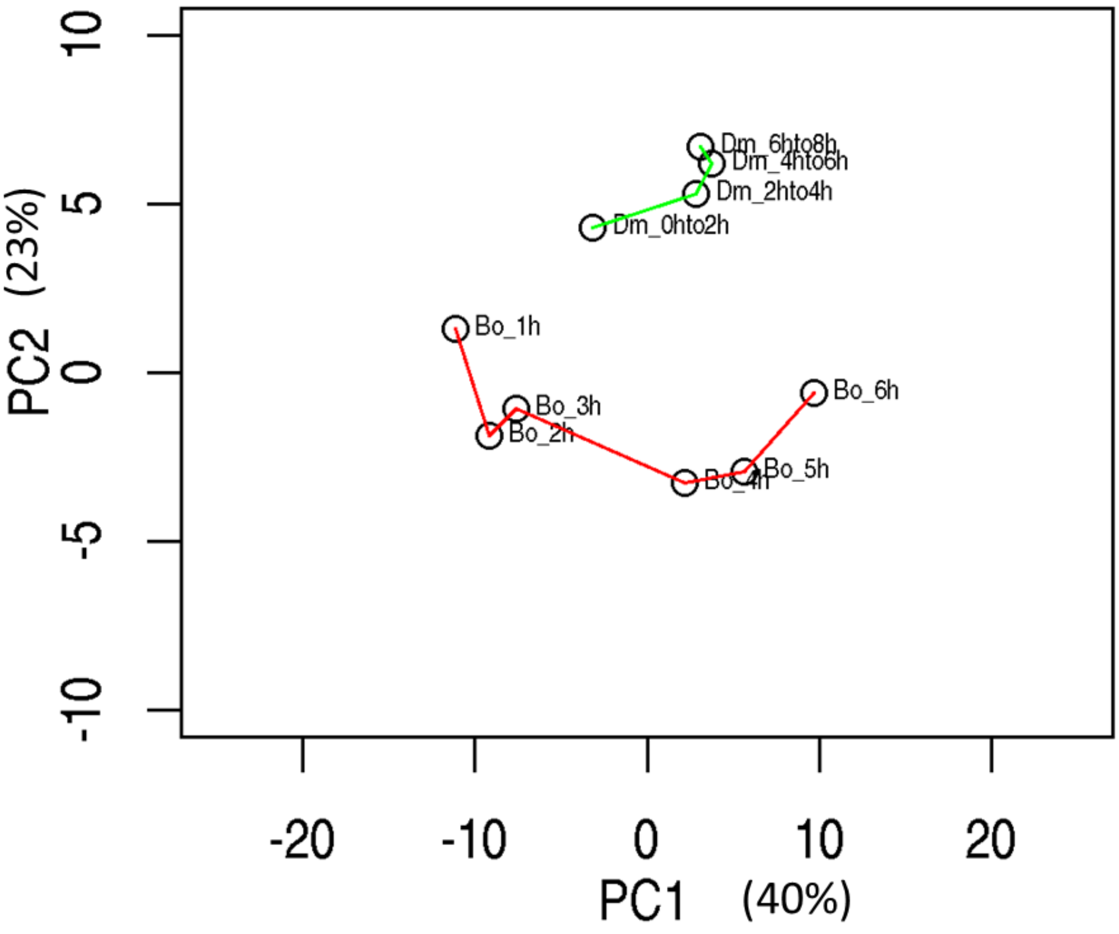

Figure S6: Principal component analysis (PCA) of early embryo development of *Bactrocera oleae* (red) and *D. melanogaster* (green). PCA was performed using the 100 most variable genes. For each organism the individual points are labelled with the corresponding scientific name initials and the hours after egg laying (Bo_1h for example refers to *B. oleae* 1 hour after egg laying). Data for *D. melanogaster* was downloaded from the FlyBase [37].

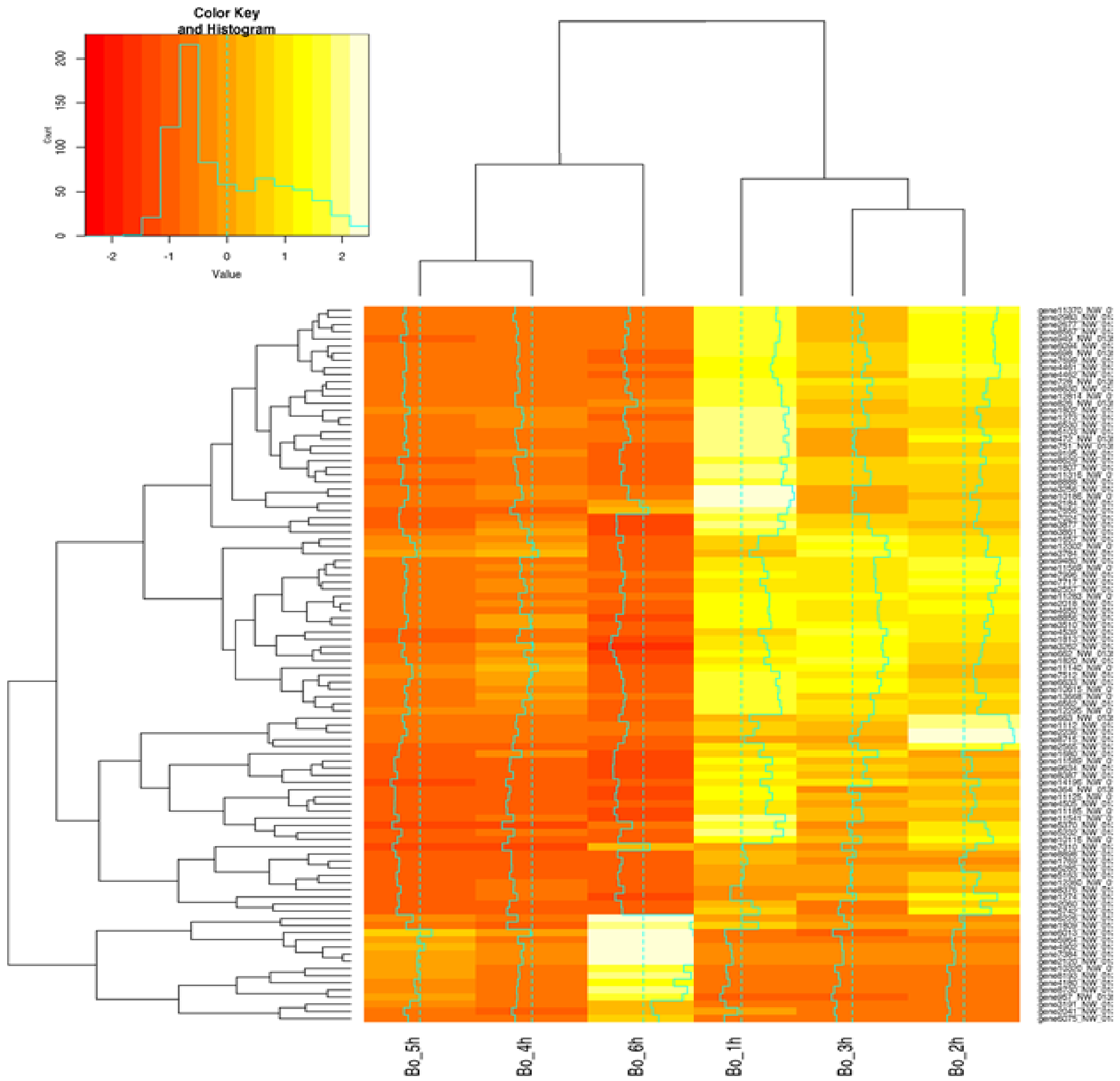

Figure S7: Hierarchical clustering of *Bactrocera oleae* embryo timepoints showing the similarity of transcript abundance between the 1-3 hours after egg laying (AEL) and 4-6 hours AEL timepoints, respectively. Clustering was done based on the 100 most variable genes from the 6 different experimental time points.

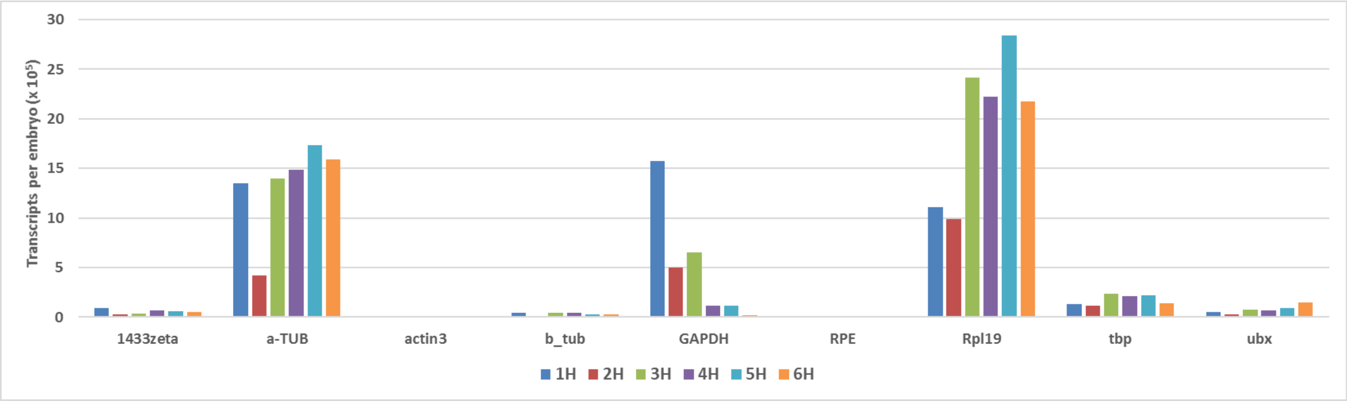

Figure S8: Comparison of absolute gene expression patterns of 9 genes routinely used as reference genes in qPCR normalization [8]. The number of transcripts per embryo is shown for *14-3-3 zeta*, *alpha Tubulin* (α-Tubulin), *Actin 3*, *beta Tubulin* (β-Tubulin), *Glyceraldehyde 3-phosphate dehydrogenase* (GAPDH), *DNA-directed RNA polymerase II* (RPB1), *ribosomal protein L19* (*RPL19*), *TATA box binding protein* (*TBP*), and *homeotic protein ultrabithorax* (UBX)**.**

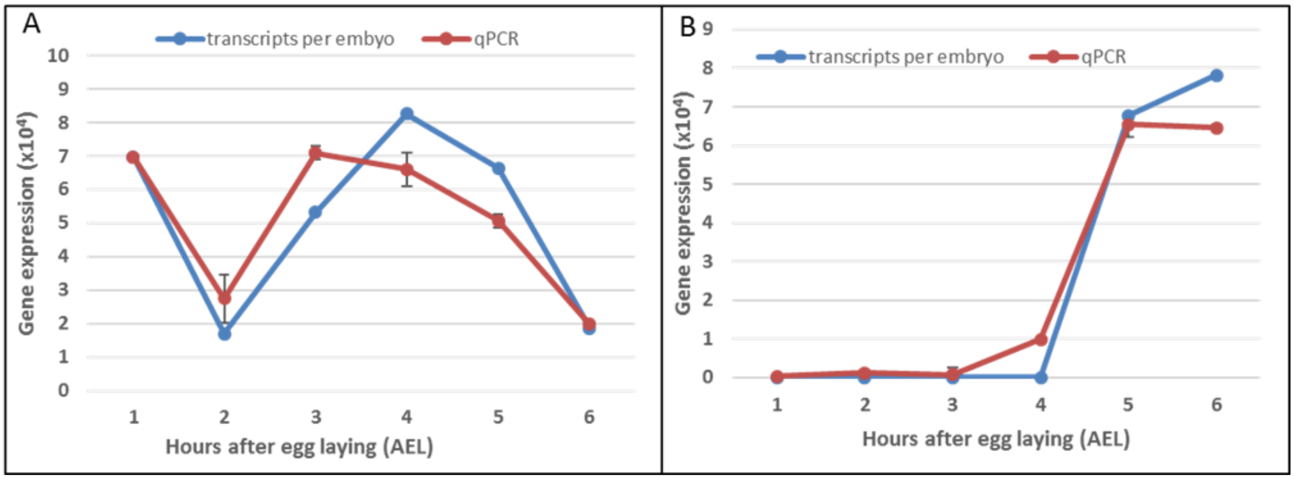

Figure S9: Real-time quantitative PCR (qPCR) expression of *hid* and *sry-a*. A) Absolute expression (blue) and qPCR expression (red) of serendipity alpha (*sry*) normalized with 14-3-3 *zeta*. qPCR expression values were scaled to compare expression profiles. **B)** Same as A but for head involution defective *(hid)*. These results were generated using a different set of biological samples. The qPCR used oligo(dT) and random primers during the reverse transcription step. Standard error of the mean of two biological replicates is depicted in bars.

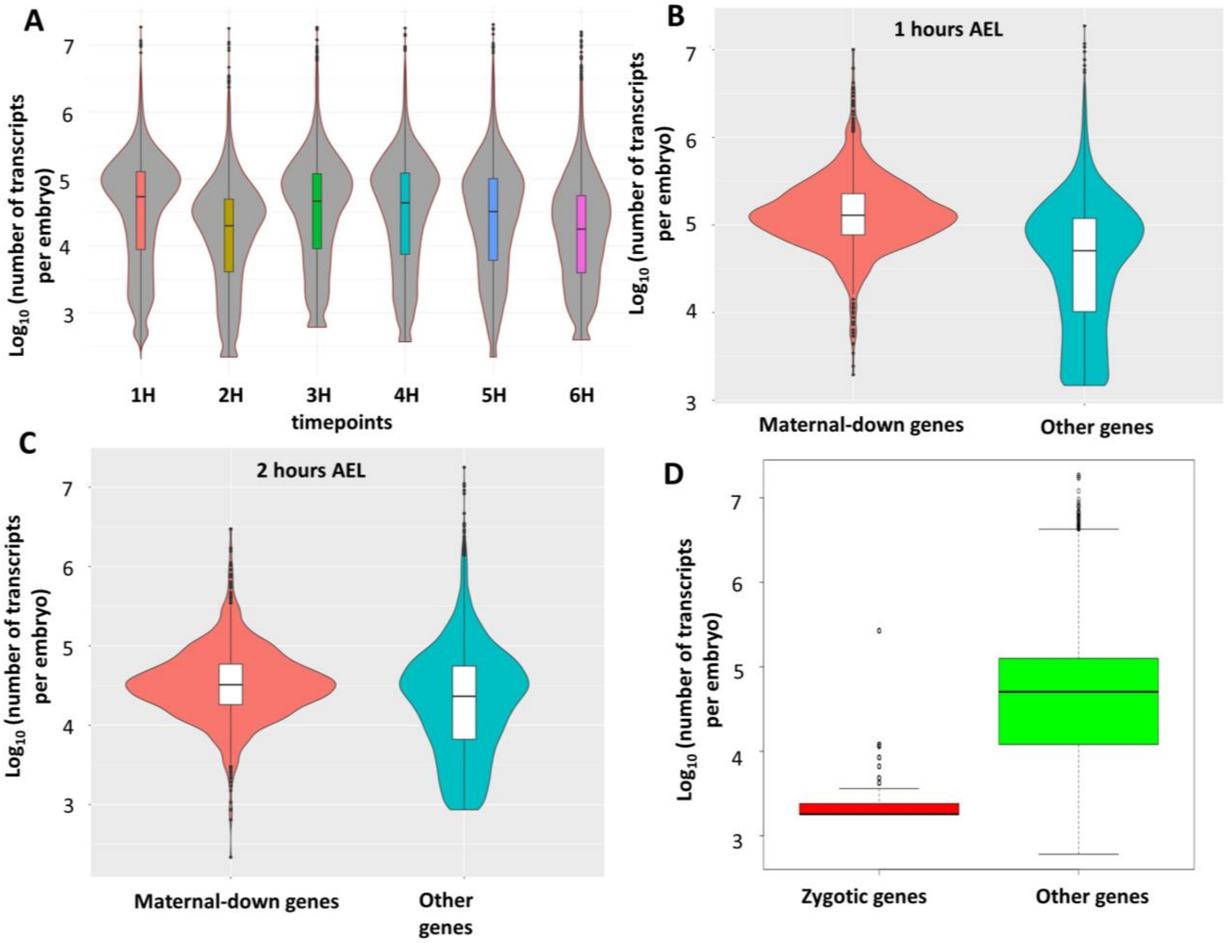

Figure S10: Profile of gene expression during embryo development reveals reduction in abundance of highly expressed maternal genes. **A)** Violin plot showing the variation in gene expression across timepoints. **B)** Violin plot comparing gene expression between maternal-down genes and the rest of the genes at 1-hour after egg laying (AEL). **C)** Same as B but for 2 hours AEL. **D)** Boxplot comparing gene expression pattern of zygotic genes to all the other genes at 3 hours AEL. Maternal-down genes are defined as genes that were most significantly reduced in abundance between 1-hour AEL and 2 hours AEL (log2 fold change >1). Zygotic genes are genes whose expression was not detected at 1 or 2 hours AEL but detected thereafter suggesting they were transcribed from the zygotic genome as opposed to being maternally derived. This figure shows that some maternally derived transcripts are at very high abundance and within the 2nd hour of embryo development these genes are downregulated such their levels is similar to other genes. This re-organization does not involve any major zygotic genome activation.

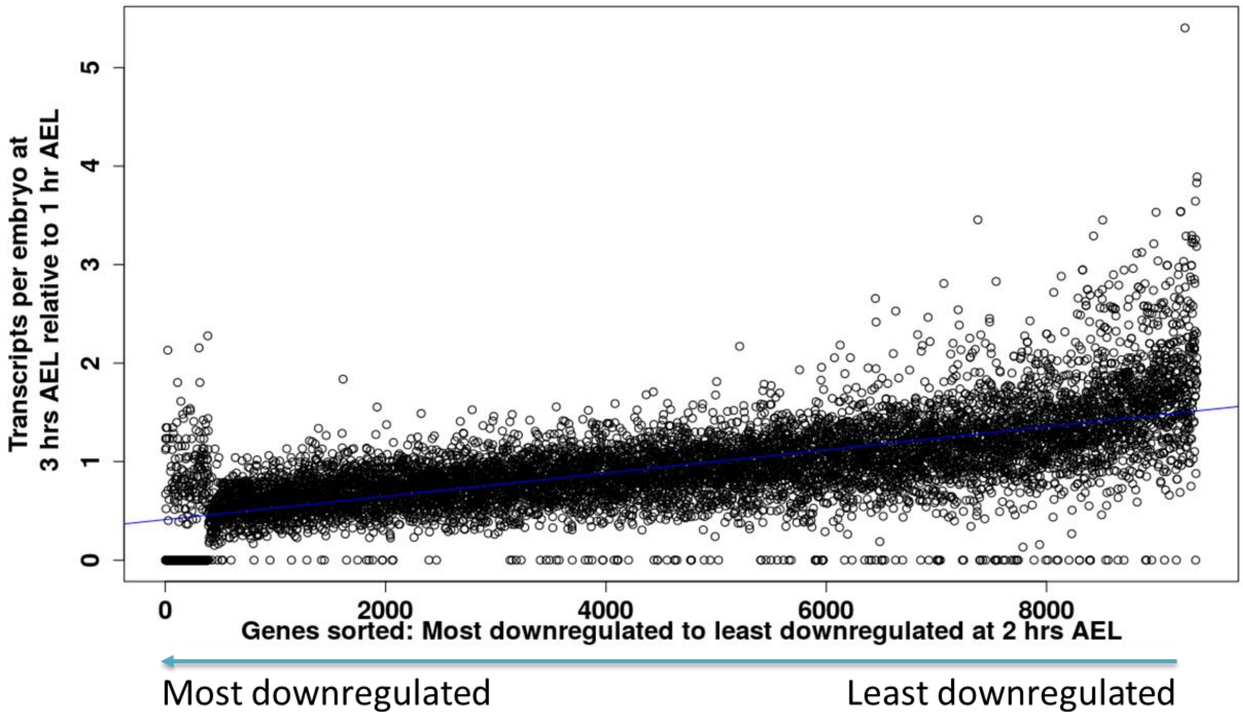

Figure S11: Reorganization of maternal transcripts prior to maternal-to-zygotic transition. We noted a drop in expression for majority of genes at 2 hours after egg laying (AEL). The percentage difference in expression of all quantifiable genes at 2 hours AEL compared to 1 hour AEL was computed as [100*(1_hr_AEL – 2_hr_AEL)/1-hr_AEL]. Genes were sorted from highest percentage (most down regulated) to lowest percentage (least down regulated). We then computed the relative expression of all these genes at 3 hours AEL compared to 1 hour AEL as [3_hr_AEL/1_hr_AEL]. The figure shows that maternally supplied genes that are most down regulated at 2 hours AEL are not later enriched at 3 hours AEL. However, the least downregulated genes are enriched later suggesting a reorganization of maternal transcripts

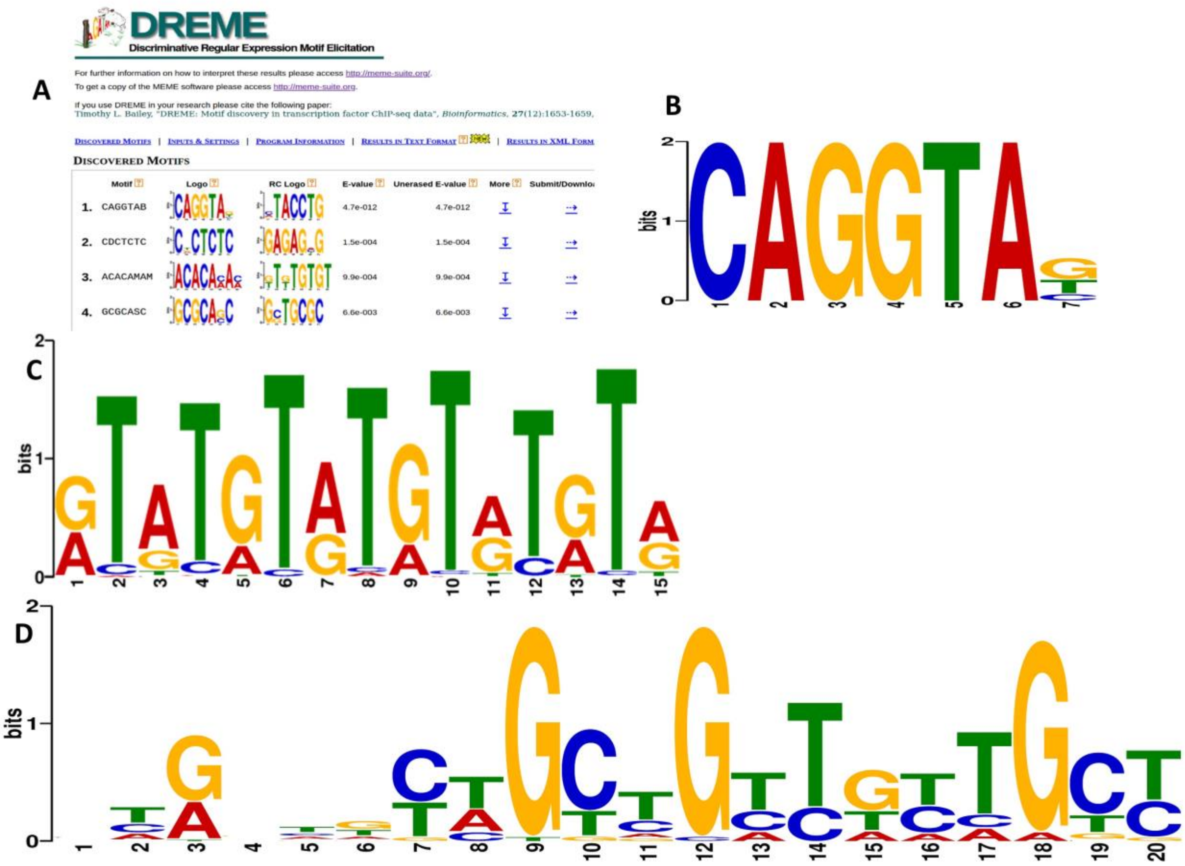

Figure S12: Motifs identified in promoter regions of zygotic and maternal genes. A) Screenshot showing results obtained with DREME tool of MEME suite [43,44]. Differentially enriched motifs in promoter sequences (1000 bp upstream of transcription start sites, TSS) between zygotic early genes and genes that are maternally supplied but downregulated and have no evidence of being transcribed from the zygote were searched using DREME. This identified the CAGGTAB motif shown in **(B)** as the most enriched. **C)** RTATGTRTGTRTRTR motif found to be enriched in the promoter regions of 1132 zygotic genes (genes that were not detectable at 1 or 2 hours AEL but detectable at other time points). This enriched motif was identified using MEME [45] (*E-value* 2.0e-138). **D)** Motif enriched in Maternal-down genes (these are maternally supplied genes that are downregulated at 2 hours after egg laying and have no evidence of being transcribed from the zygote).

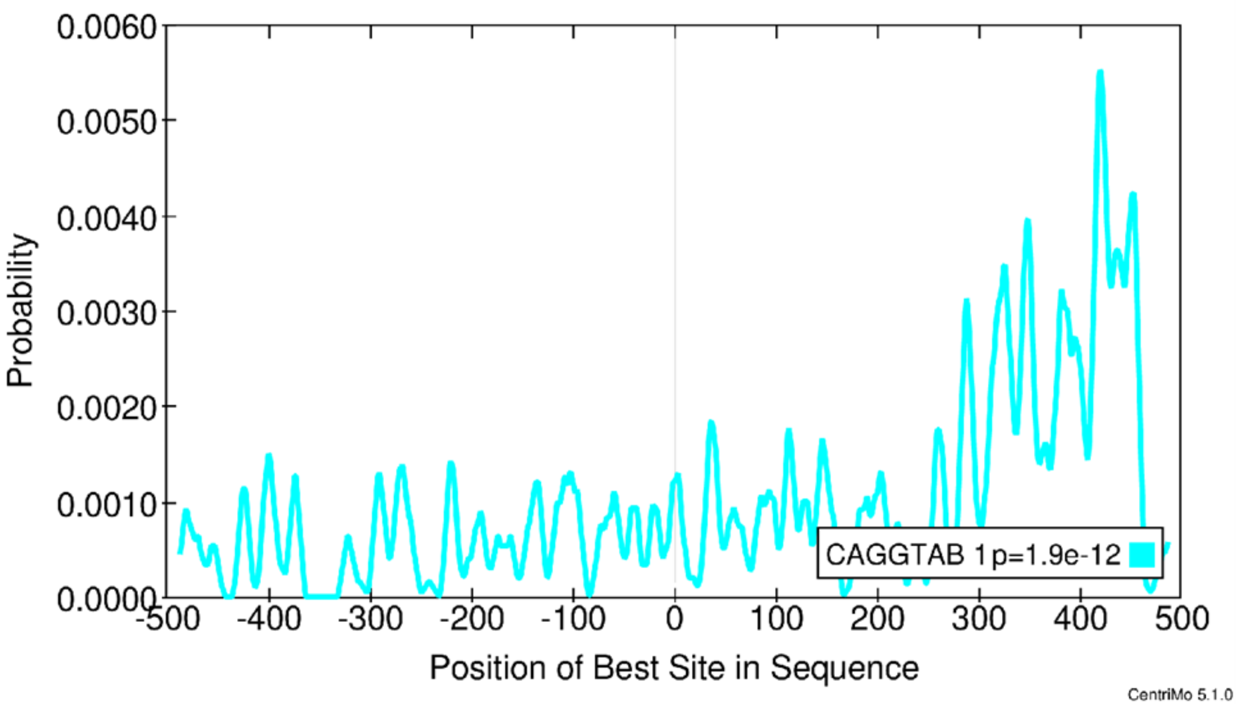

Figure S13: Significant CAGGTAB motif enrichment 500 bp upstream of transcription start in promoter regions. CentriMo[41] output showing significant enrichment of CAGGTAB motif in the 300 bp upstream of the promoter region of the transcription start site of 159 early zygotic genes identified using GFOLD differential expression. Promoter region was defined as 1000 bp upstream of the transcription start site.

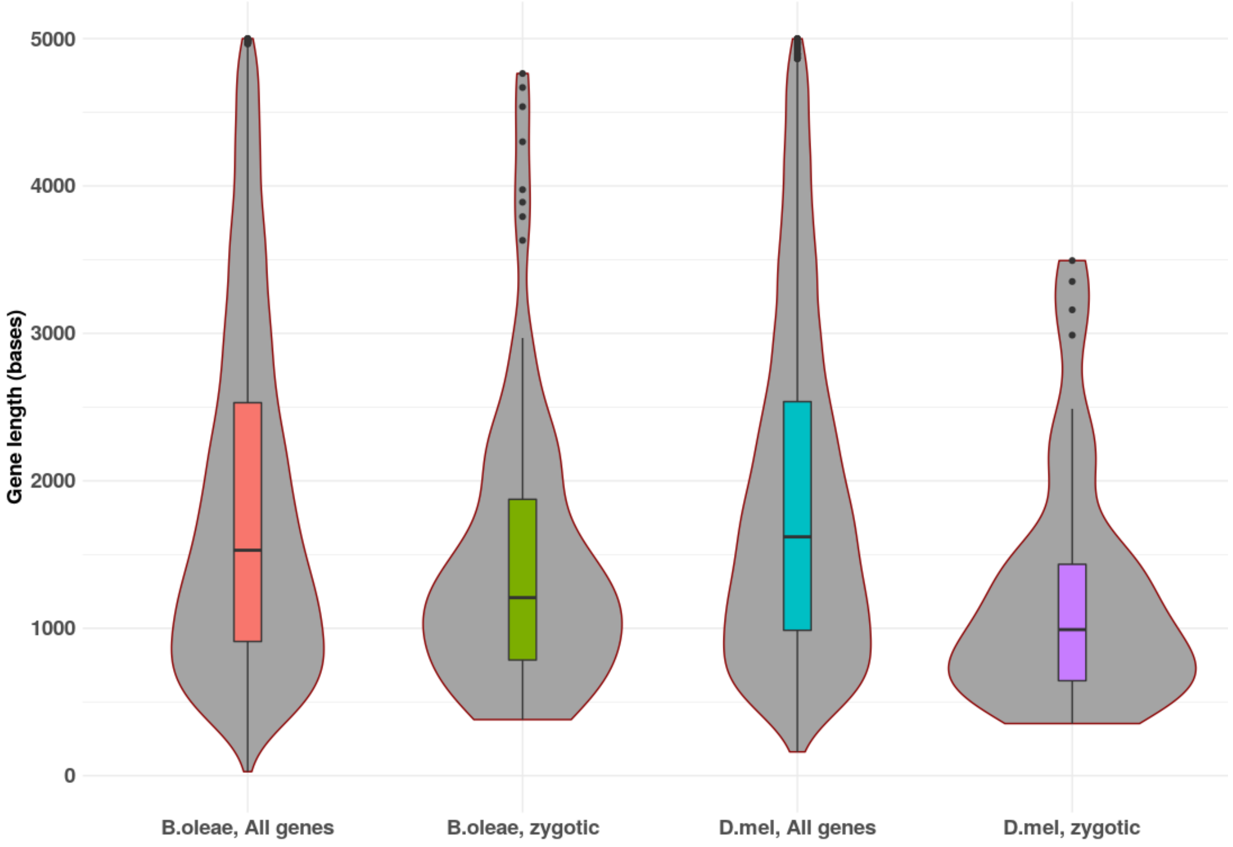

Figure S14: Early zygotic genes are shorter in length compared to other genes in both *Bactrocera oleae* and *Drosophila melanogaster*. Gene transfer files (GTF) for gene model annotations from both *Bactrocera oleae* (B.oleae) and *Drosophila melanogaster* (D.mel) were used to determine the lengths for all protein coding genes and early zygotic protein coding genes. *B. oleae* early zygotic genes were identified in this study while *D. melanogaster* early zygotic genes were identified by De Renzis et al[53]. *B. oleae* GTF file was taken from the NCBI *Bactrocera oleae* annotation release 100 including the novel genes identified in this study while *D. melanogaster* GTF file was from flybase[37] version r6.32. Zygotic gene lengths for both *B. oleae* and *D. melanogaster* differ significantly from nonzygotic genes (Wilcox p-values 5.1e -7 and 9.1e-7, respectively)

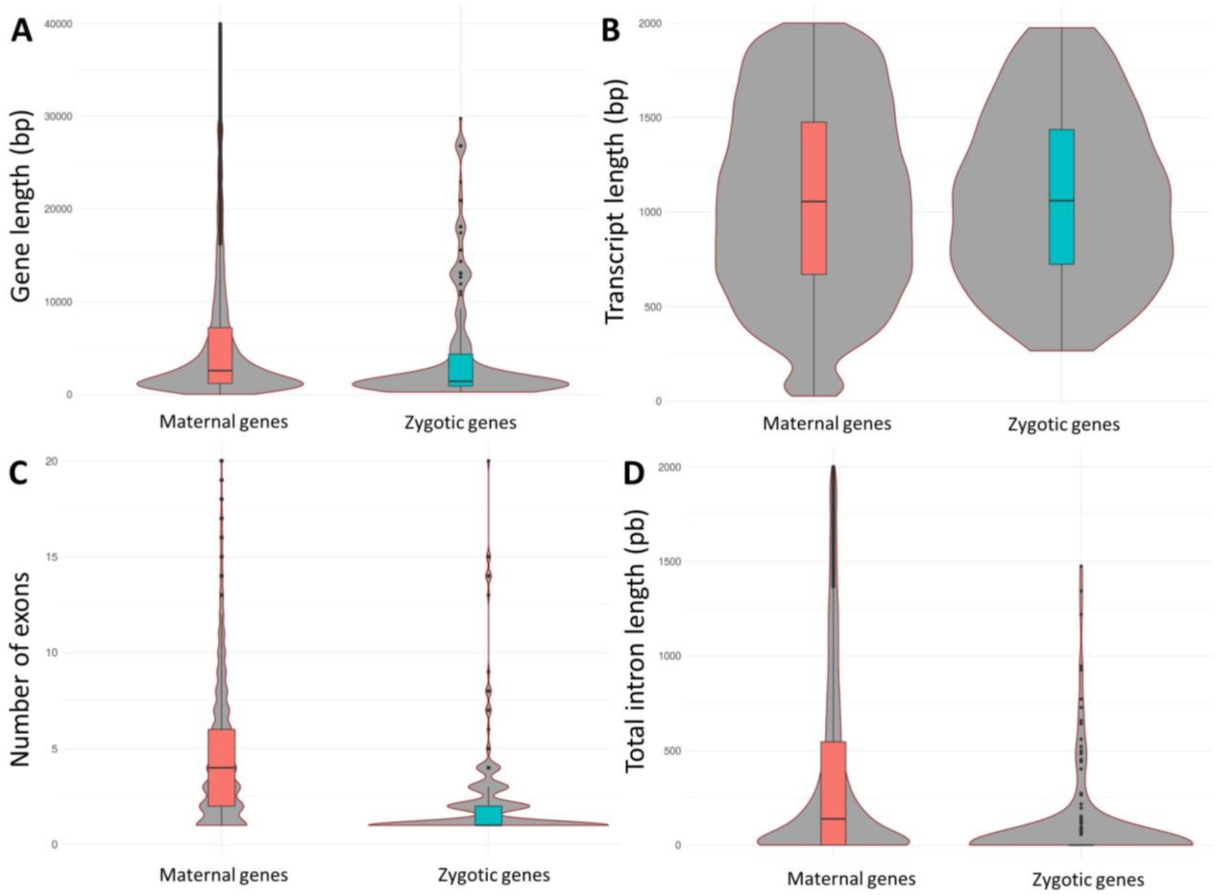

Figure S15: Comparison of zygotic and maternal genes lengths in *Bactrocera oleae*. Comparison of zygotic and maternal gene body length (**A**), transcript length (**B**), number of exons (**C**), and total intron length (**D**). Both zygotic and maternal genes were identified using GFOLD differential expression method. The *B. oleae* maternal gene lengths and zygotic gene lengths (A) were significantly different (Wilcox p-value 5.2e-8) whereas their transcript lengths (B) were not significantly different (Wilcox p-value 0.8).

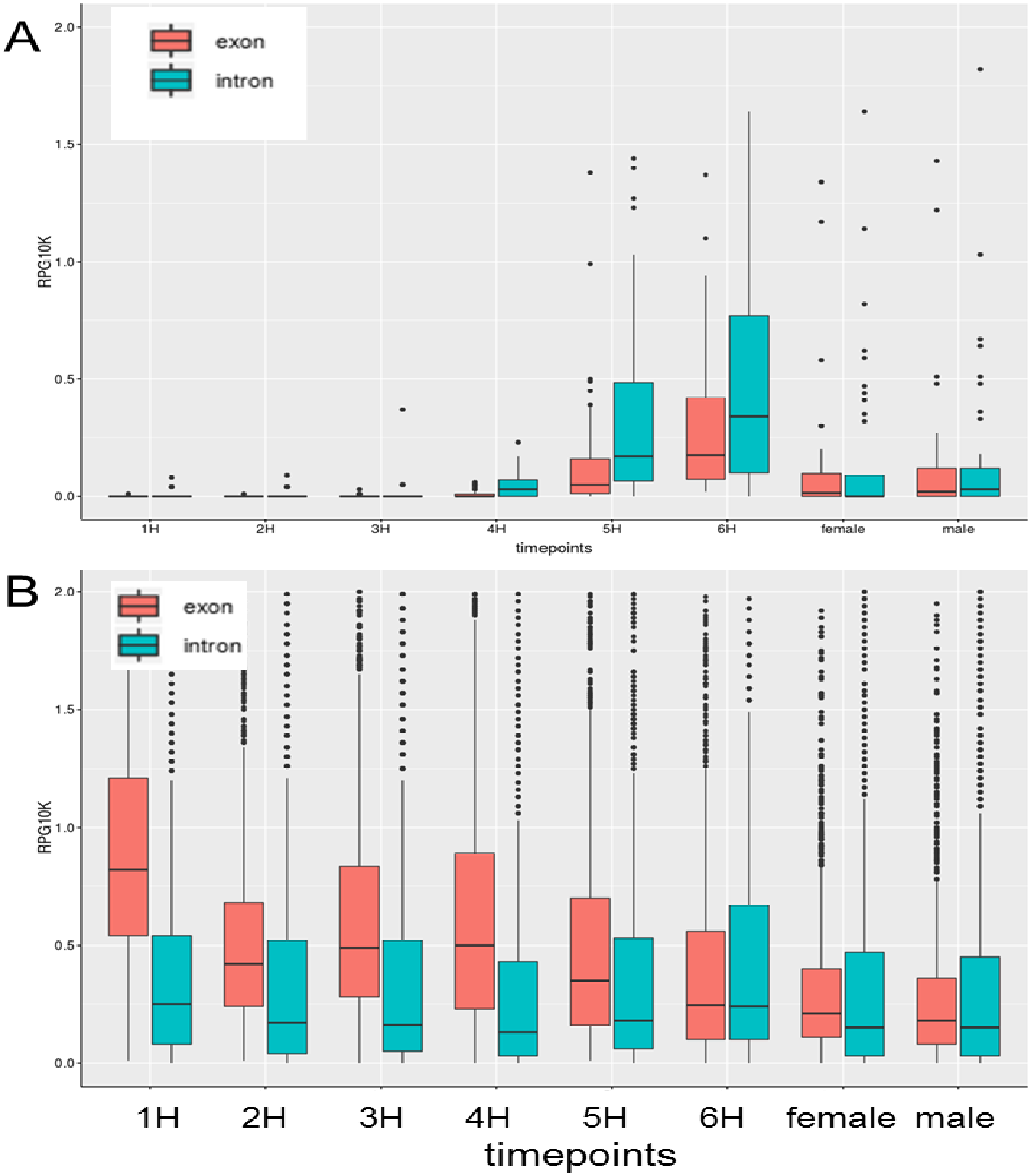

Figure S16: Comparison of intron and exon coverage among zygotic (A) and maternal-down genes (B). Zygotic and maternal genes were initially determined using GFOLD differential expression. The relative abundance of transcripts having reads that exclusively align to introns or exons was estimated using Mandalorion. Reads per gene per 10000 mapped reads (RPG10K).

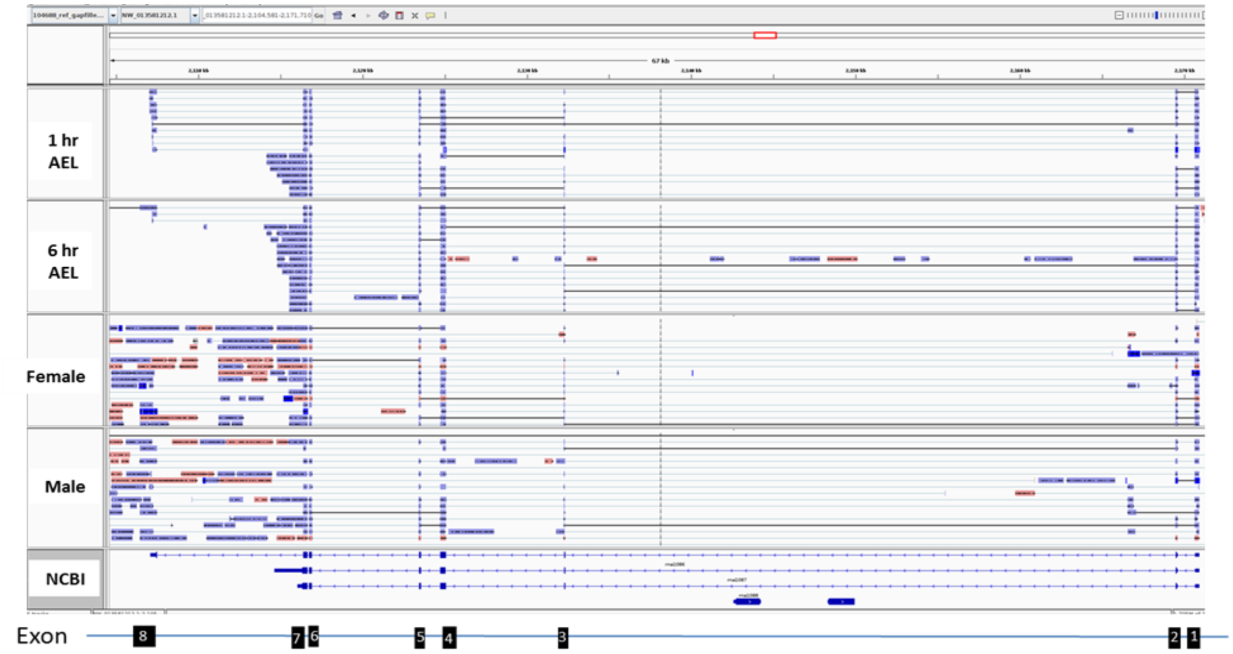

Figure S17: IGV screenshot showing long read alignments to *Bactrocera oleae sex lethal* (*Sxl*). Panels show alignment of raw long reads generated from embryos at 1 hour after egg laying (AEL), 6 hours AEL, adult female heads, and adult male heads, respectively. The NCBI predicted gene model is shown in the bottom-most panel. IGV=Integrated genome viewer

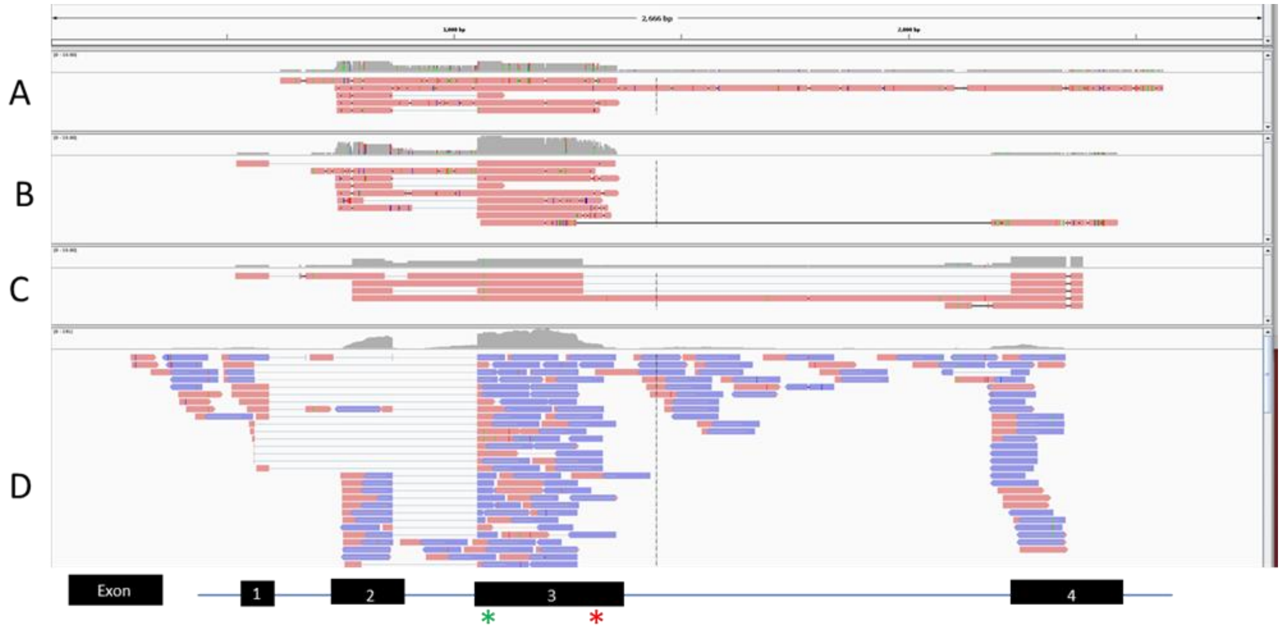

Figure S18: IGV screenshot showing read alignments to *Bactrocera oleae* maleness on Y (*BoMoY*) gene. A) First *BoMoY* transcripts observed at 5 hours after egg laying (AEL) using long-read RNA-seq, **B)** *BoMoY* transcripts observed at 6 hours AEL using long-read RNA-seq, **C)**

BoMoY Trinity assembled isoforms obtained from Meccariello at al., [54], **D)** Paired-end reads obtained from Illumina short-read RNA-seq of embryo samples at 6 hours AEL. Illumina reads are shown as combined forward-reverse. The gene model is shown as black boxes below panel D. Asterisks indicate location of start codon (green) and stop codon (red). IGV=Integrated genome viewer

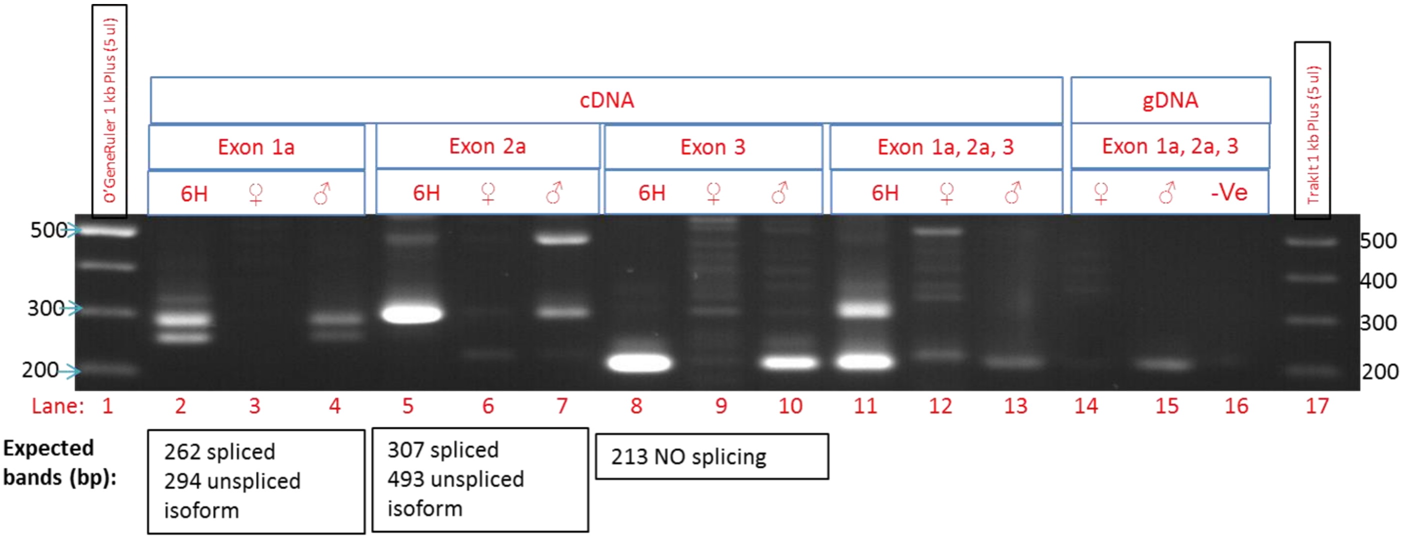

Figure S19: PCR amplification of regions of *Bactrocera oleae* maleness on the Y chromosome (*BoMoY*) transcripts. Lane 2 to 13: PCR products amplified using forward primers targeting the shown exons. The BoMoY_R_exon3 reverse primer was used for all reactions. Lanes 11 – 16 used a multiplex of forward primers. For reactions in lanes 1-13, cDNA generated from embryos at 6 hours after egg laying (6H AEL), adult female heads (♀), and adult male heads (♂) was used. For reactions in lanes 14 and 15, genomic DNA extracted from adult whole female and male insects, respectively, was used. Lane 16 shows the negative control. Lanes 1 and 17 show the ladders O'GeneRuler 1 kb plus (Thermo Fisher Scientific) and TrackIt 1 kb Plus (Thermo Fisher Scientific), respectively, and their band sizes in base pairs. See Table S13 for a list of the primers used. No PCR product is expected from female samples since *BoMoY* is located on the Y chromosome. See Figure S24 for the full uncropped version.

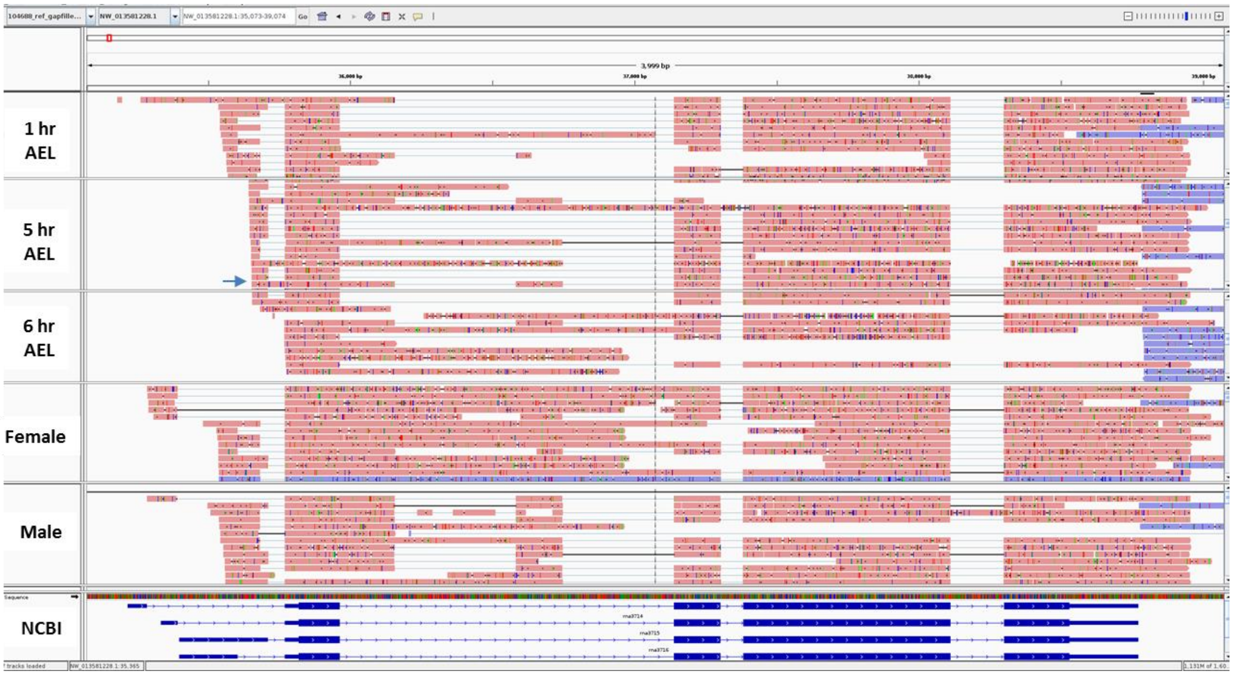

Figure S20: IGV screenshot showing long read alignments to *Bactrocera oleae transformer* (*tra*). Panels show alignment of raw long reads generated from embryos at 1 hour after egg laying (AEL), 5 hours AEL, 6 hours AEL, adult female heads, and adult male heads, respectively. The NCBI predicted gene model is shown in the bottom-most panel. The arrow indicates the male-specific isoform first detected at 5 hours AEL. IGV=Integrated genome viewer

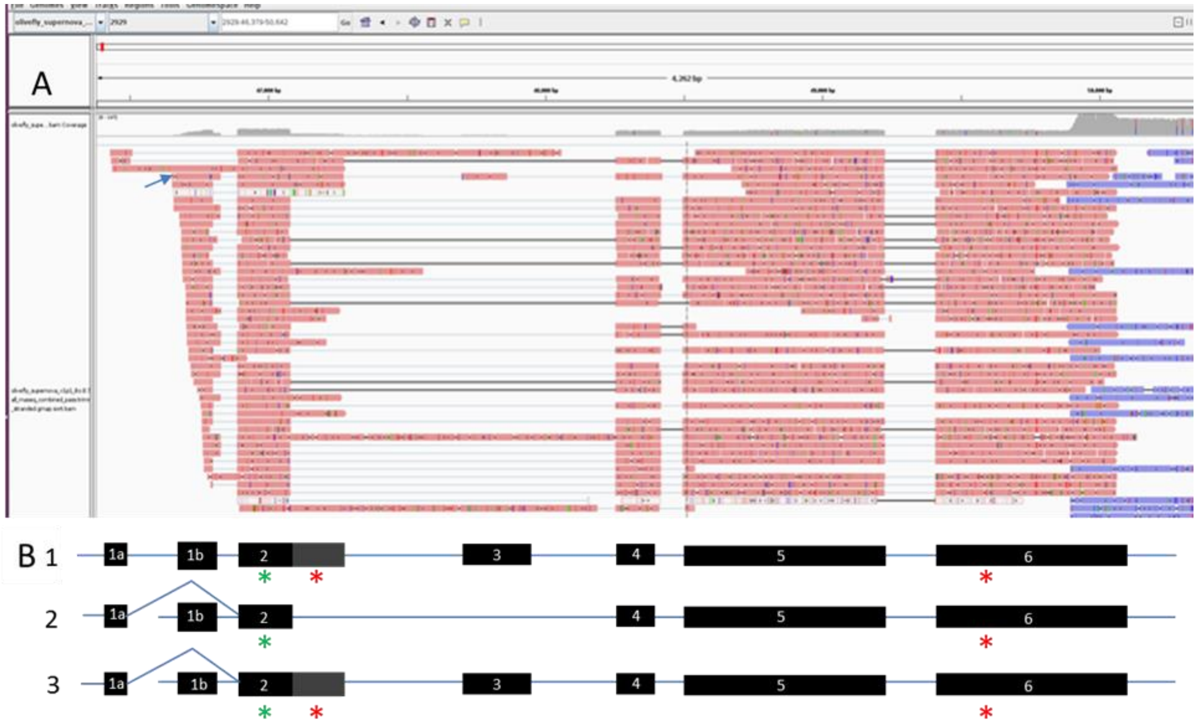

Figure S21: IGV screenshot showing long read alignments to *Bactrocera oleae Transformer* gene (*tra*). A) IGV screenshot showing reads aligned to *tra* observed in our bulk long-read RNA-seq of mixed-sex embryos at 5 hours after egg laying. Arrow indicates the signature transcript showing male-specific splicing pattern characterized by extension of exon 2 and retention of exon 3. **B)** Schematic of the full *tra* gene model (B1), female-specific *tra* isoform (B2) and male-specific *tra* isoform (B3). Asterisks indicate location of start codon (green) and stop codon (red). IGV=Integrated genome viewer

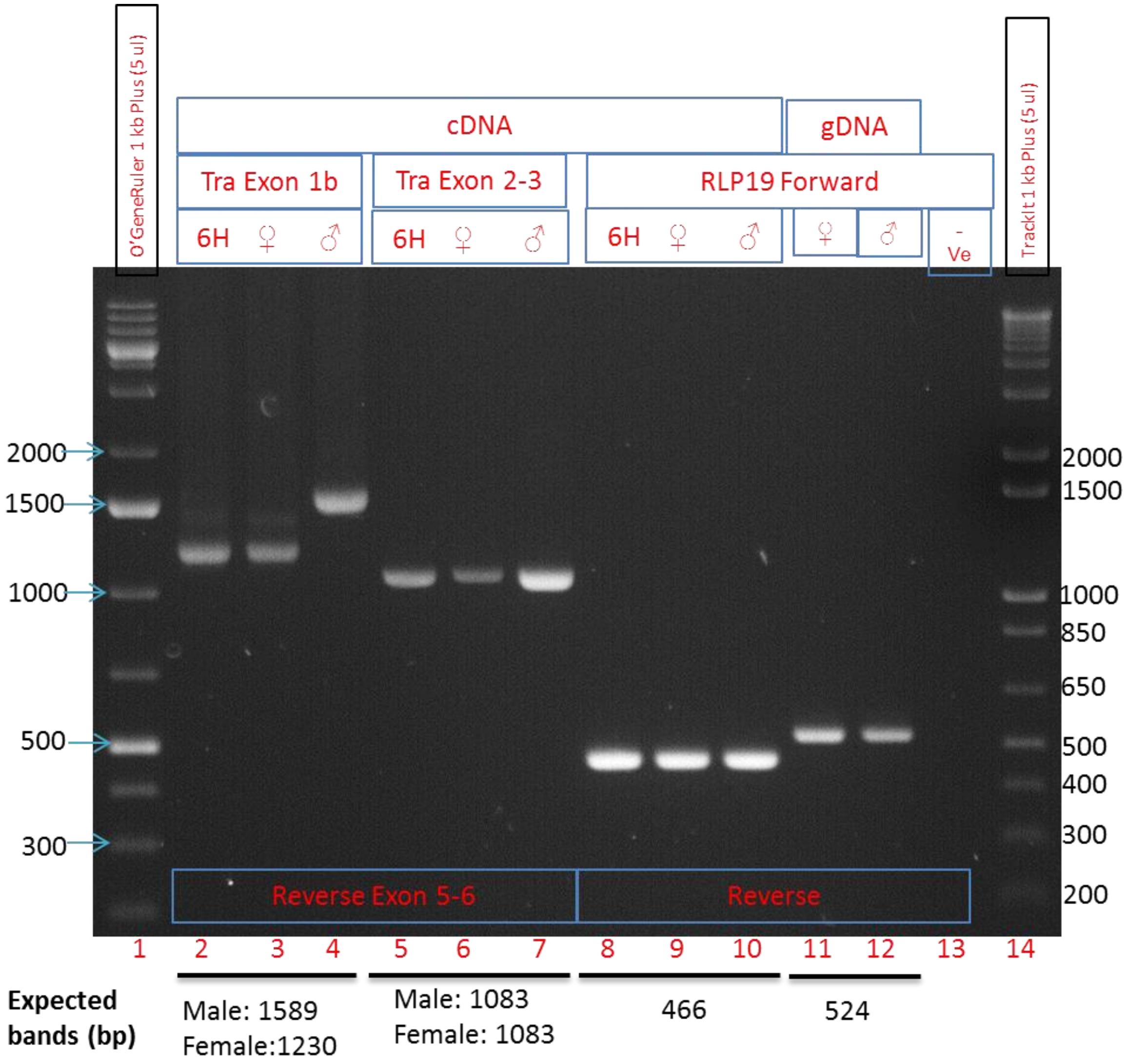

Figure S22: PCR amplification of regions of *Bactrocera oleae* Transformer (*Tra*) transcripts. Lane 2 to 7 show PCR products amplified using forward primers targeting exon 1b and the splice junction of exons 2 and 3. The Botra_R_exon5-6 reverse primer was used for these reactions. Ribosomal protein L19 (RPL19) was added as a positive control in lanes 8-12. For reactions in lanes 2-10, cDNA generated from embryos at 6 hours after egg laying (6H AEL), adult female heads (♀), and adult male heads (♂) was used. For reactions in lanes 11 and 12, genomic DNA extracted from adult whole female and male insects was used. Lane 13 shows the negative control. Lanes 1 and 14 show the ladders O'GeneRuler 1 kb plus and TrackIt 1 kb plus, respectively, and their band sizes (Thermo Fisher Scientific). See Table S13 for a list of the primers used.

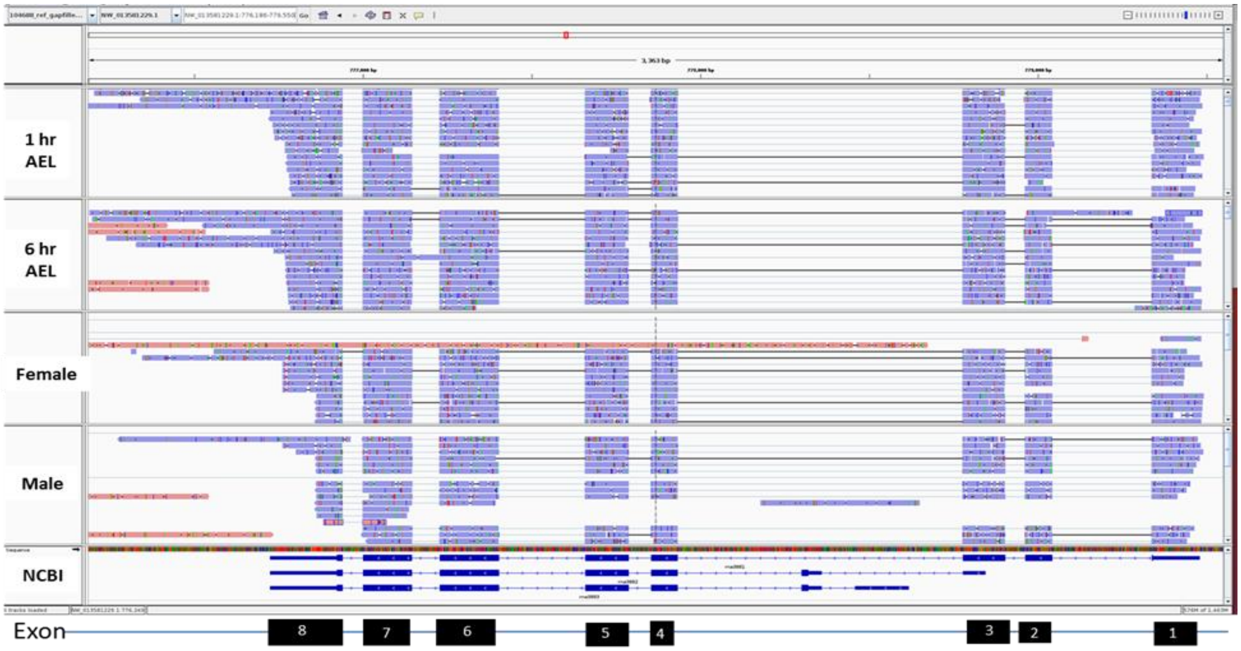

Figure S23: IGV screenshot showing long read alignments to *Bactrocera oleae transformer 2* (*tra2*). Panels show alignment of long reads generated from embryos at 1 hour after egg laying (AEL), 6 hours AEL, adult female heads, and adult male heads, respectively. The NCBI predicted gene model is shown in the bottom-most panel. IGV=Integrated genome viewer.

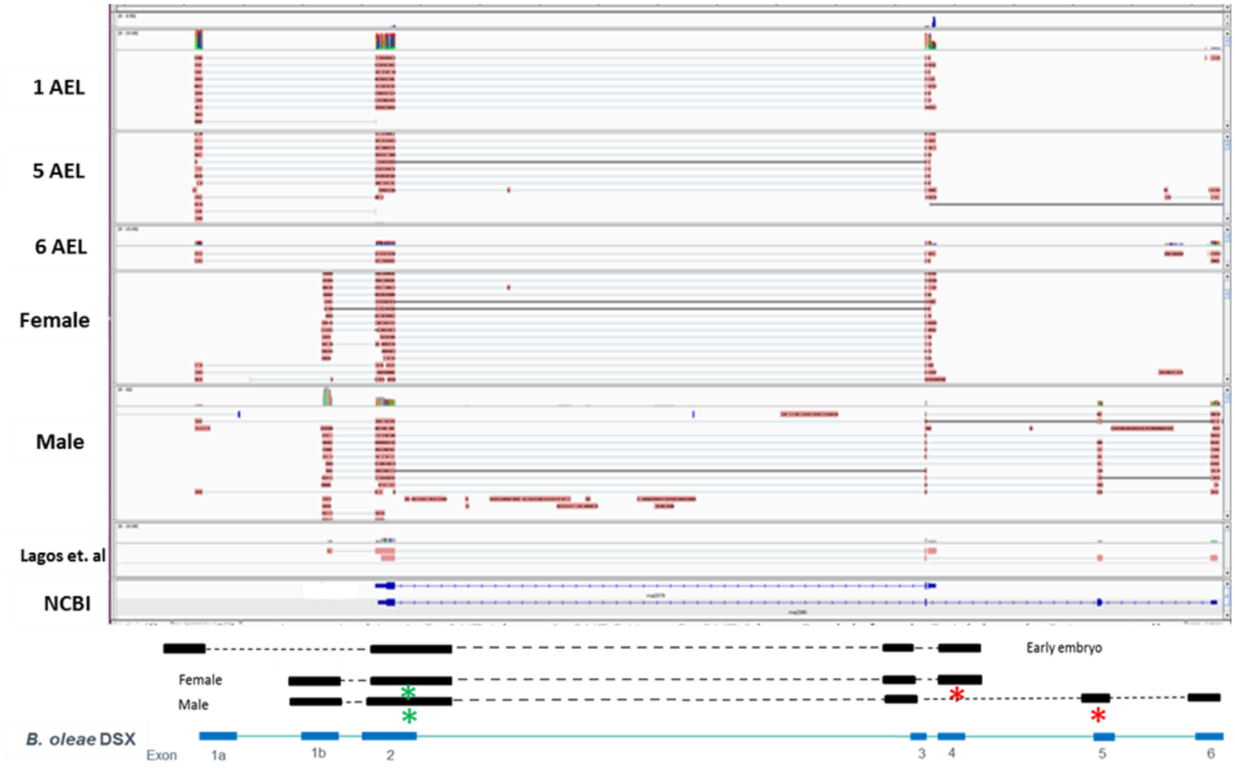

Figure S24: IGV screenshot showing long read alignments to *Bactrocera oleae double sex* (*dsx*). Reads are shown for embryos at 1, 5, and 6 hours after egg laying (AEL), and female and male adult heads. Male and female specific *dsx* cDNA as generated by Lagos et al. [51] are shown. NCBI predicted gene model for *dsx* is shown in the bottom-most panel. The dominant isoform from the embryo stage, female heads, and male heads are shown below the screenshot. Asterisks indicate location of start codon (green) and stop codon (red). IGV=Integrated genome viewer

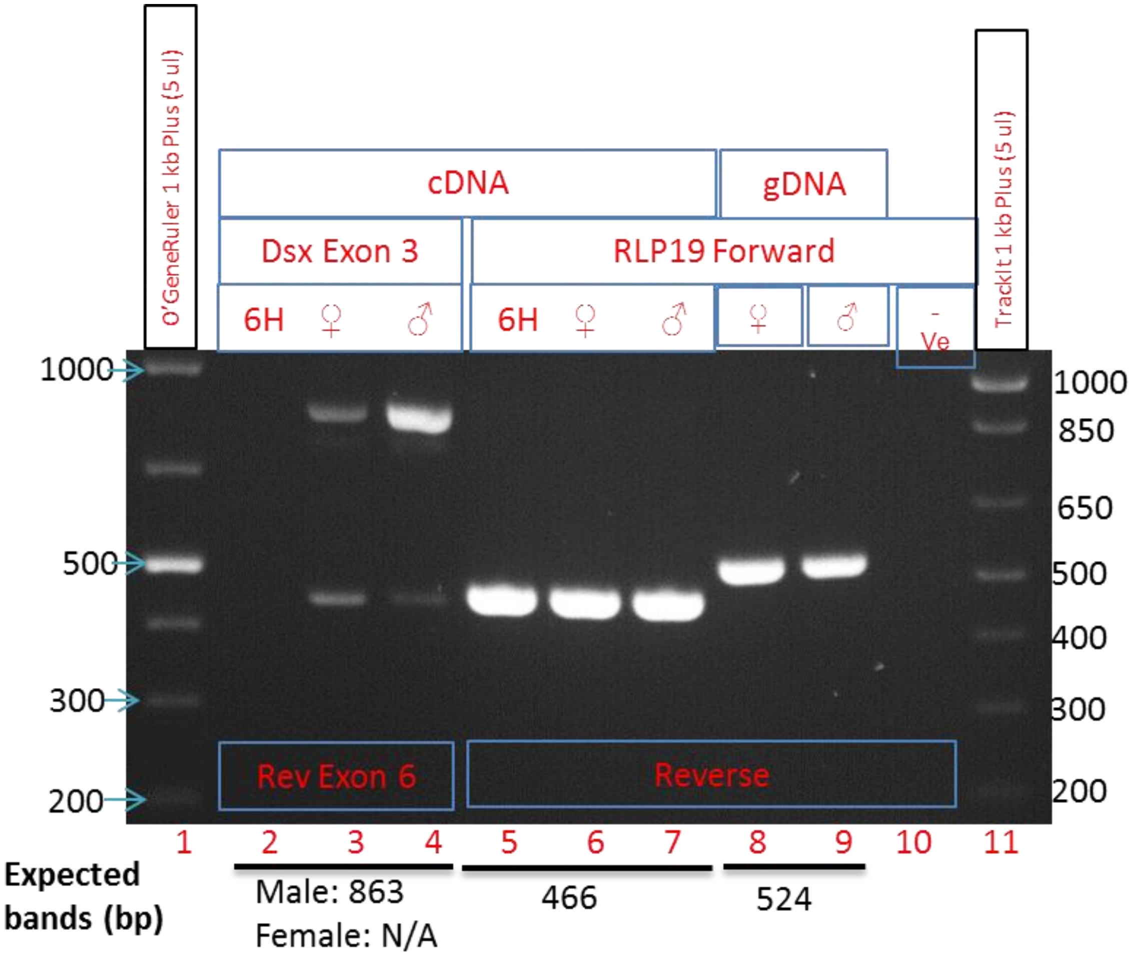

Figure S25: PCR amplification of regions of *Bactrocera oleae* Double sex (*Dsx*) transcripts. Lane 2 to 4 show PCR products amplified using forward primers targeting the region from exons 3 to 6. Ribosomal protein L19 (RPL19) was added as a positive control in lanes 5 to 9. For reactions in lanes 2-7, cDNA generated from embryos at 6 hours after egg laying (6H AEL), adult female heads (♀), and adult male heads (♂) was used. For reactions in lanes 8 and 9, genomic DNA extracted from adult whole female and male insects was used. Lane 10 shows the negative control. Lanes 1 and 11 show the ladders O'GeneRuler 1 kb plus and TrackIt 1 kb plus (Thermo Fisher Scientific), respectively, and their band sizes. See Table S13 for a list of the primers used.

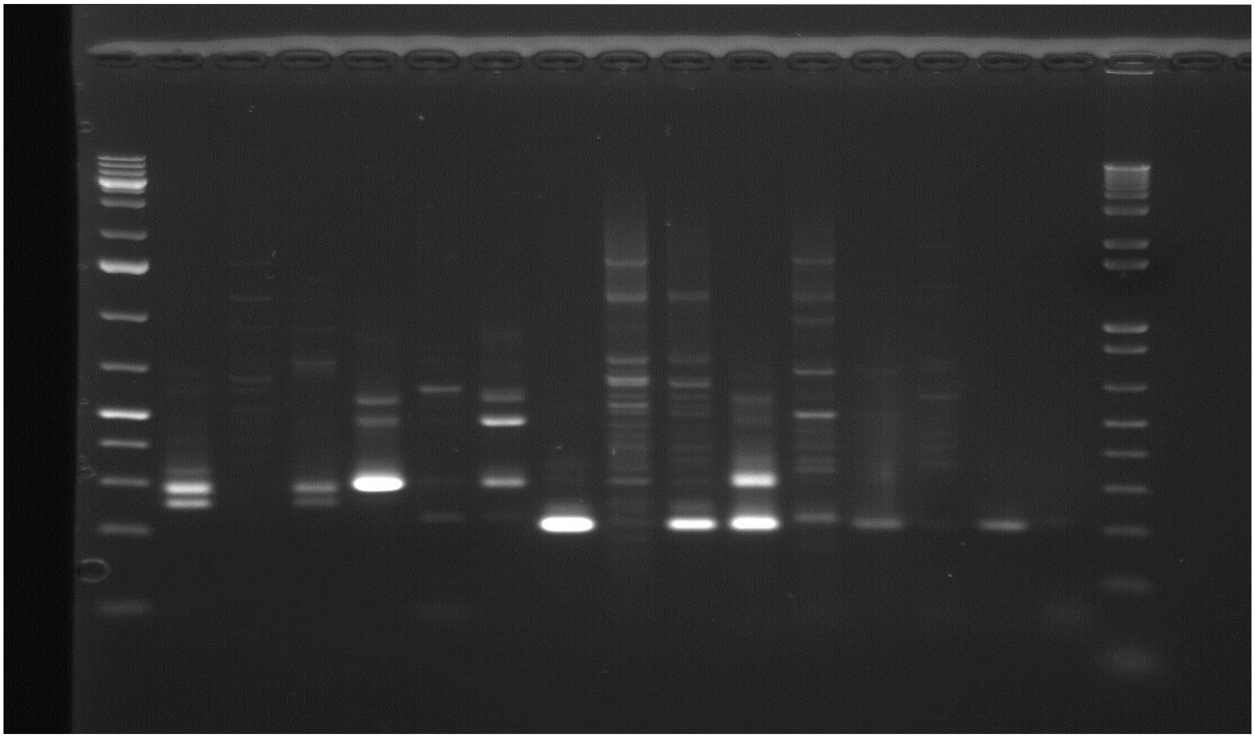

Figure S26: Full uncropped version of Figure S19.

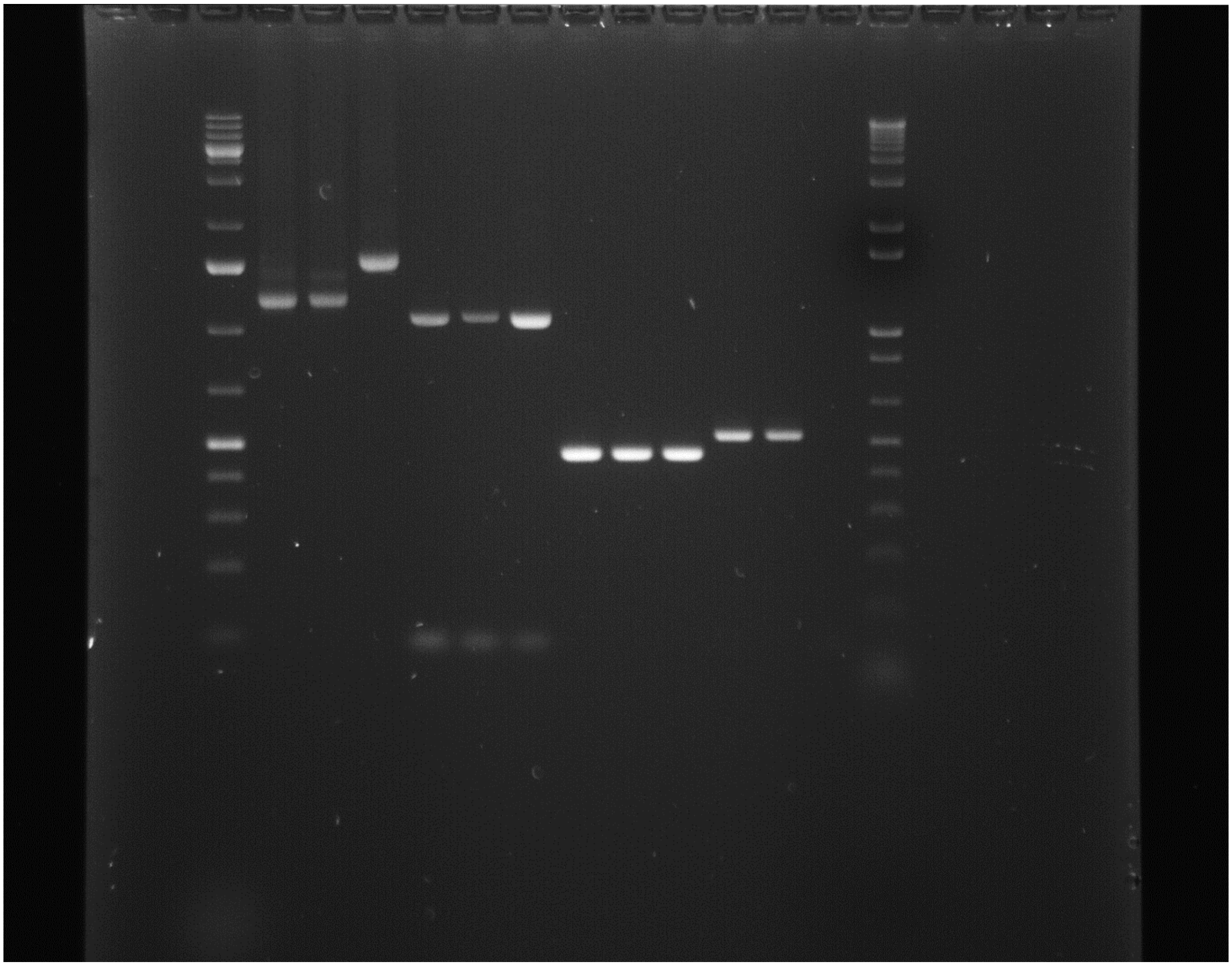

Figure S27: Full uncropped version of Figure S22.

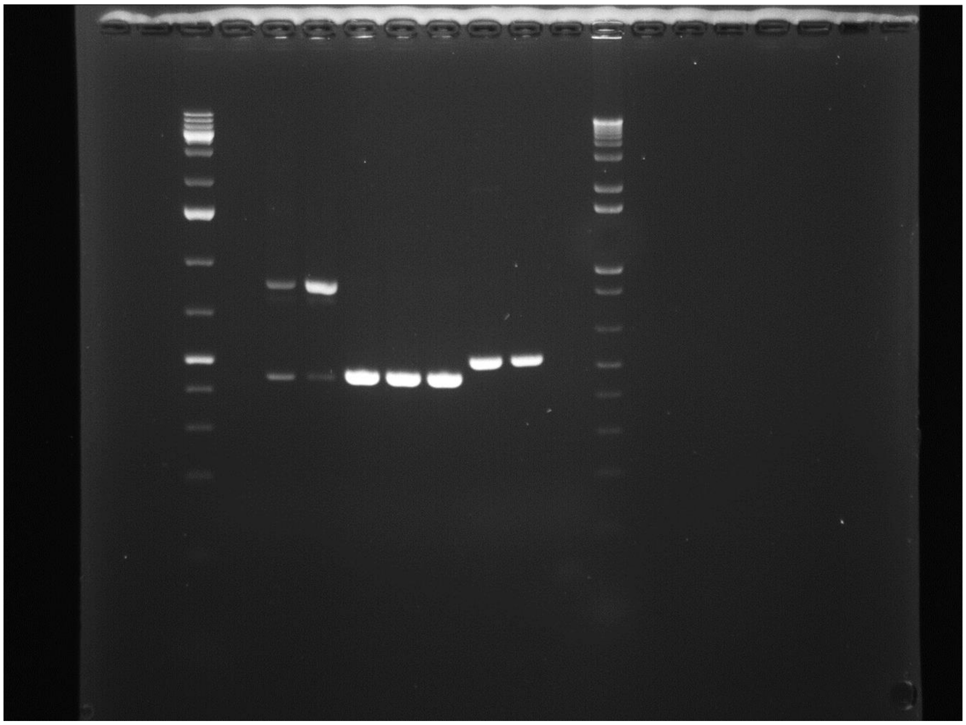

Figure S28: Full uncropped version of Figure S25.

Extended Tables

Table E1: Comparison of short-read and long-read genome guided *de novo* transcriptome assemblies to the NCBI predicted gene models. Cufflinks[23] and Cupcake ToFU were used to generate the short-read and long-read transcriptome assemblies, respectively. Cuffcompare from Cufflinks was used to compare the gene models to the NCBI assembly which is taken as the reference.

	Sensitivity (%)		Precision (%)	
	Short-read	Long-read	Short-read	Long-read
Base level	83.3	82.9	65	74.9
Exon level	61.4	65.4	54.9	46.1
Intron level	74.9	75.3	85	69.9
Intron chain level	41.1	49.3	44.1	25.9
Transcript level	42	50.5	26.4	19.9
Locus level	62	74.4	29.4	60.2

Sensitivity is the proportion of reference nucleotides that have been correctly assembled in the transcriptome assemblies while Precision is the proportion of nucleotides in the transcriptome assembly that are actually found in the reference. Thus, Sensitivity is a reflection of the number of features in the reference are absent in assembly (the higher the number of features the lower the sensitivity). Precision is the proportion of features present in the assembly but missed in the reference.

Table E2: Comparison of the *de novo* genome guided transcriptome assembly generated using Illumina short-read RNA-seq and Nanopore long-read RNA-seq. The number of reads required to detect the same number of genes between Illumina and ONT with 95 % coverage was determined using sampling (Extended Figure 9). *De novo* genome guided transcriptome assemblies were generated using Trinity and ToFU, respectively. The final transcriptome assemblies were analyzed using SQANTI. See Table 1 for explanation of structural categories.

**Metric**	**Illumina short-read assembly**		**ONT long-read assembly**
**Number of raw reads used**	Read 1	Read 2	
	95443253	95,443,253	1,987,637
**Total number of bases**	9,254,970,738	9,378,238,881	2,171,874,827
**Total genes**	16951		8836
**Annotated genes**	8239		6776
**Novel genes**	8712		2060
**Total isoforms**	21840		43676
**FSM**	5962		14436
**ISM**	1522		7058
**NIC**	1575		3205
**NNC**	2632		10950
**Intergenic**	7374		3019
**Genic intron**	1500		660
**Genic genomic**	654		3675
**Antisense**	379		410
**Fusion**	242		263

FSM; full splice match, ISM; incomplete splice match, NIC; novel in catalogue, NNC; novel not in catalogue.

Extended Figures

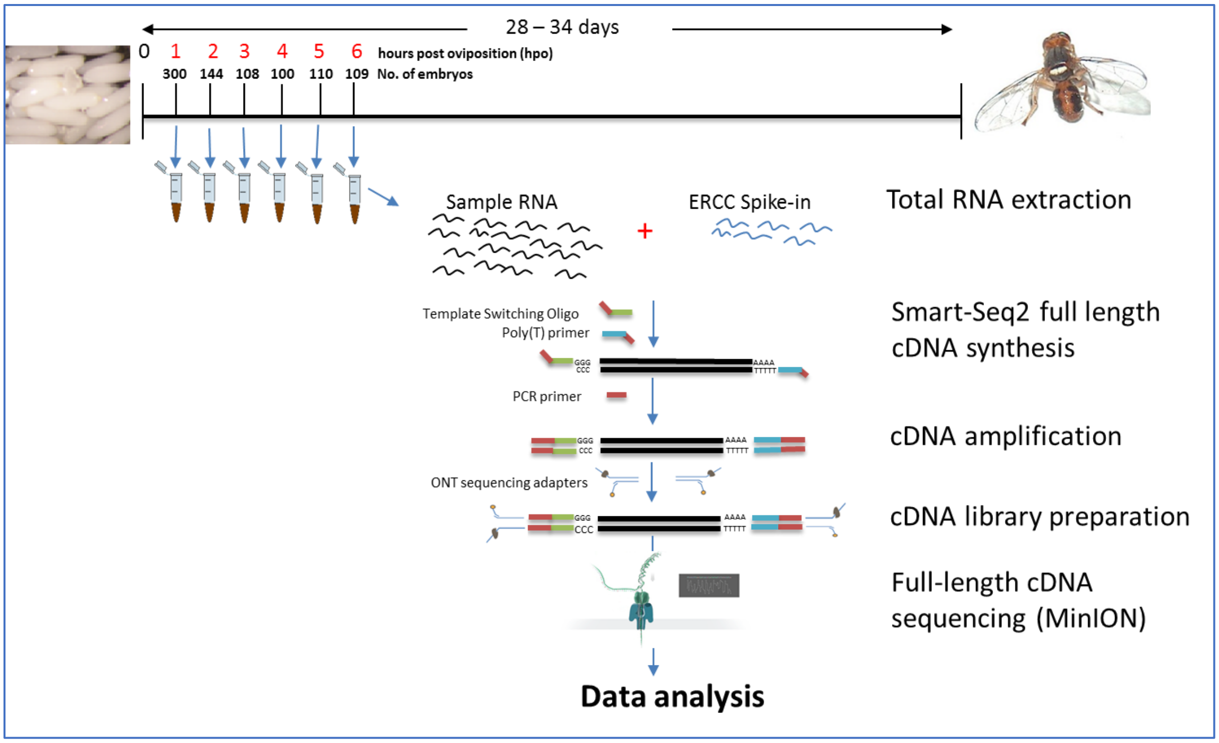

Extended Figure 1. Schematic of cDNA library generation and sequencing. Embryos were collected at hourly point post oviposition (hpo), counted and total RNA extracted using the Trizol method. At cDNA synthesis step, external RNA standards (ERCC) were added to each sample commensurate to the number of embryos that were used. The Smart-Seq2 protocol was used to generate full length cDNA, followed by PCR amplification of the cDNA. The Oxford Nanopore Technologies (ONT) SQK-LSK108 protocol for library preparation was then followed, albeit with some custom changes. The library was then sequenced on the ONT MinION, followed by basecalling using ONT Albacore basecaller.

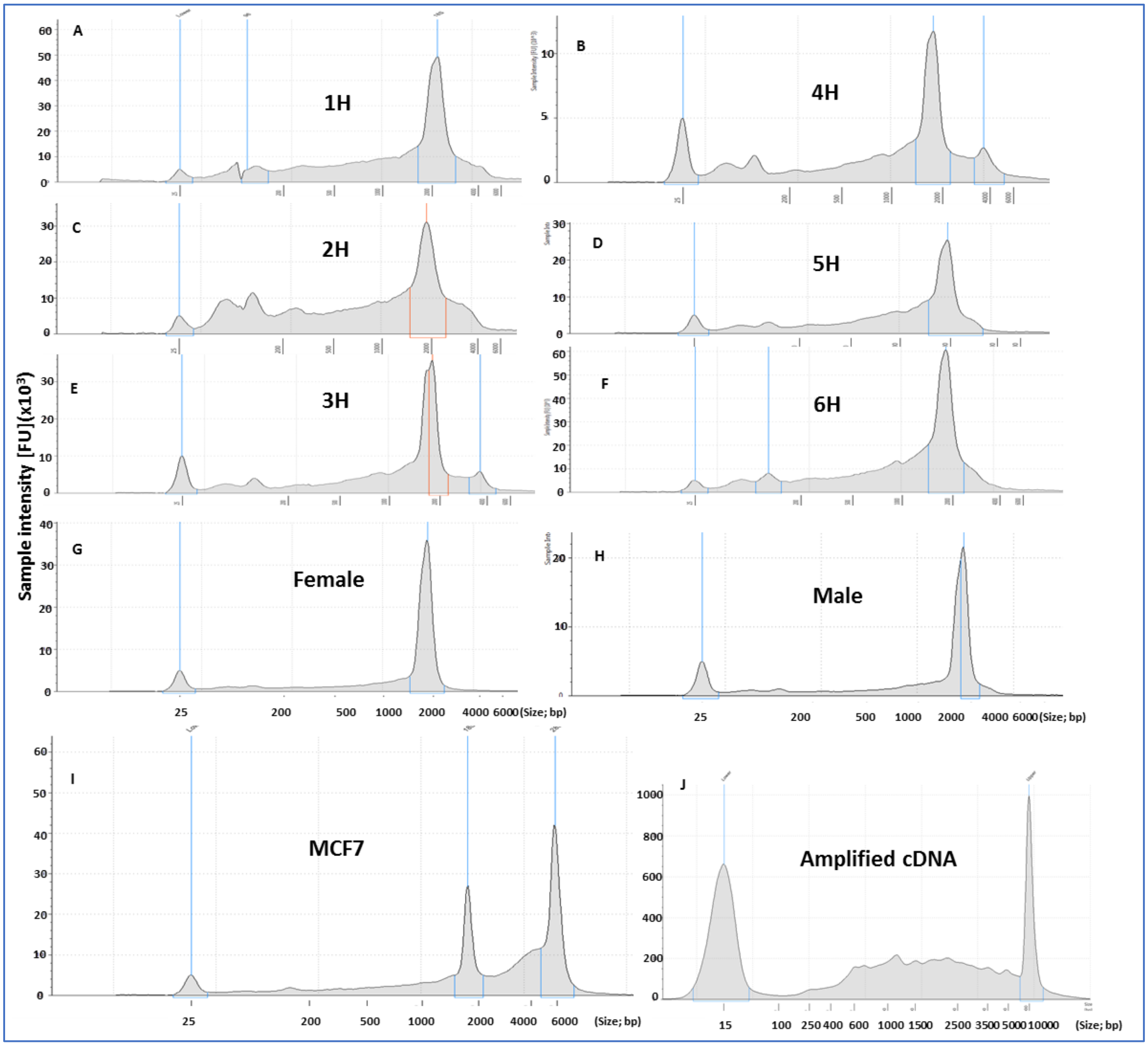

Extended Figure 2. Electropherogram showing the profile of total RNA of the samples used in this experiment. A-F) Show the profile of embryo samples at the indicated timepoints post oviposition. G-H) Show profile of mature insects. I) MCF-7 total RNA is added to show the difference in profile between mammalian and *B. oleae*. J) Example profile of amplified cDNA generated from the samples.

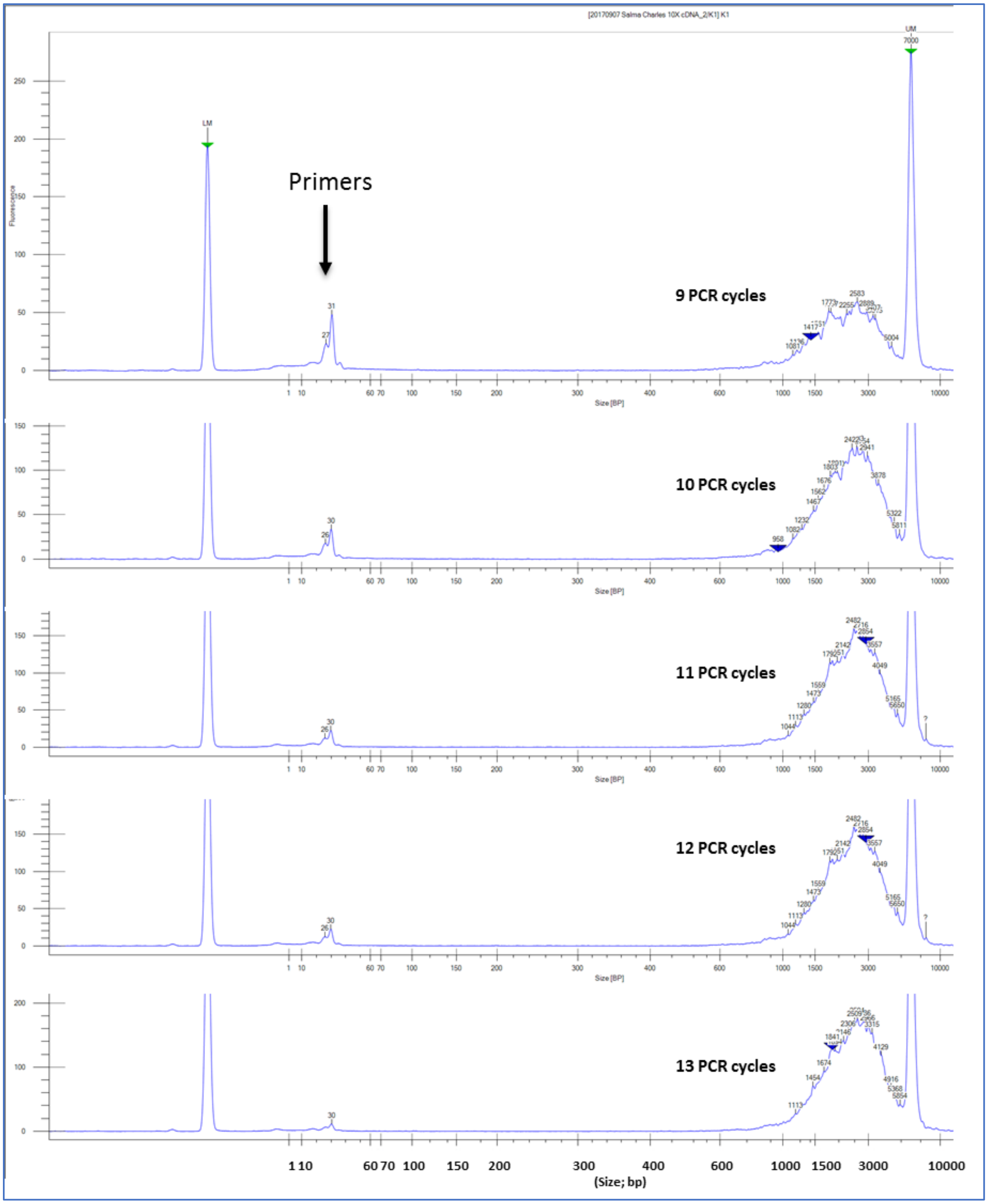

Extended Figure 3. Optimization of the number of cycles needed to amplify the cDNA in order to obtain enough material without skewing the distribution. We selected 12 cycles which gave enough material (~2 µg) without dramatically skewing cDNA profile.

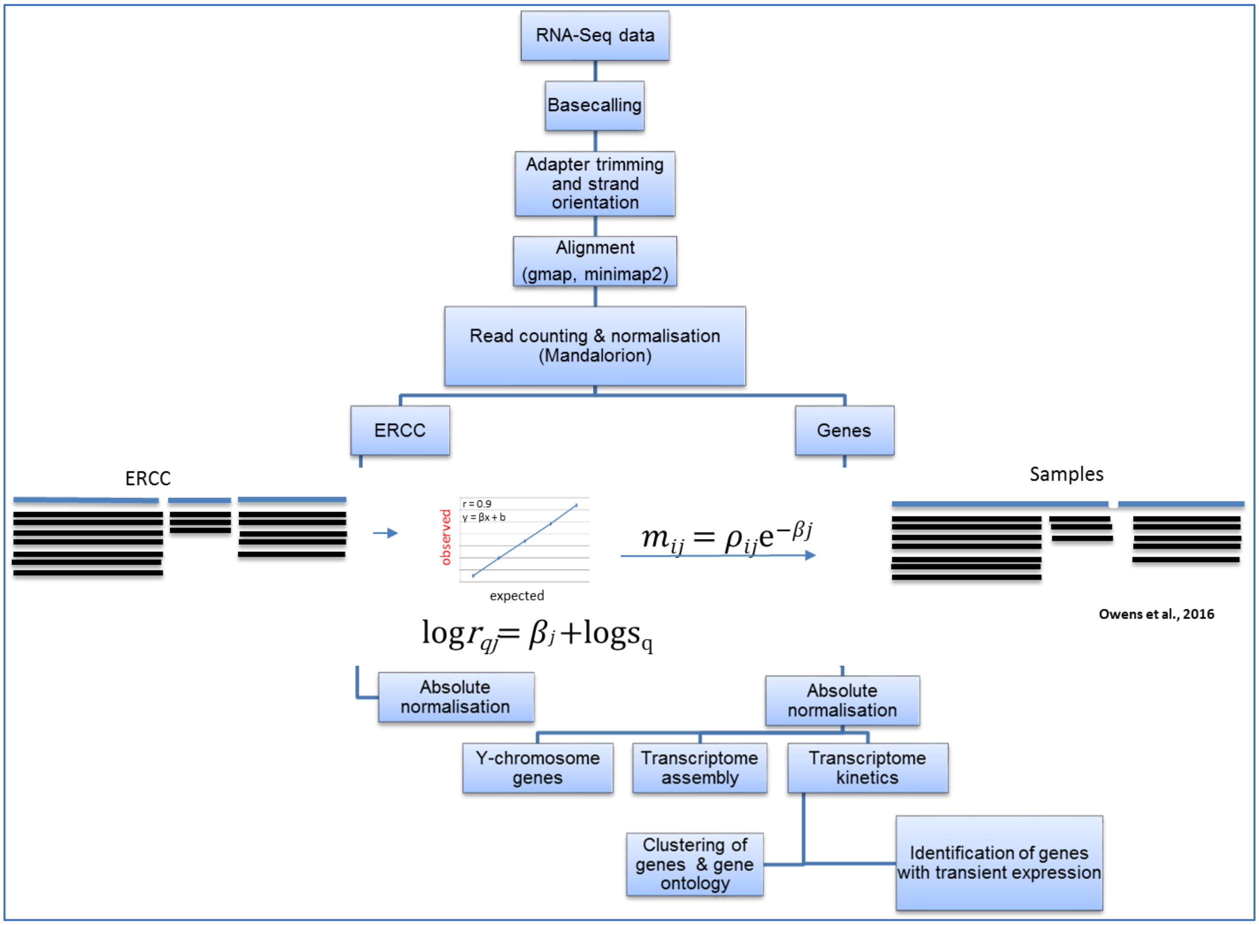

Extended Figure 4. Data analysis workflow. Following sequencing, reads were basecalled using Albacore (ONT, version 2.0.2). A customized version of Mandalorion was used to perform adapter trimming and strand orientation. Trimmed and stranded reads were aligned to the genome using GMAP followed by relative quantification of expression using Mandalorion. Relative expression were converted to absolute quantification using ERCC standards, followed by downstream data analyses

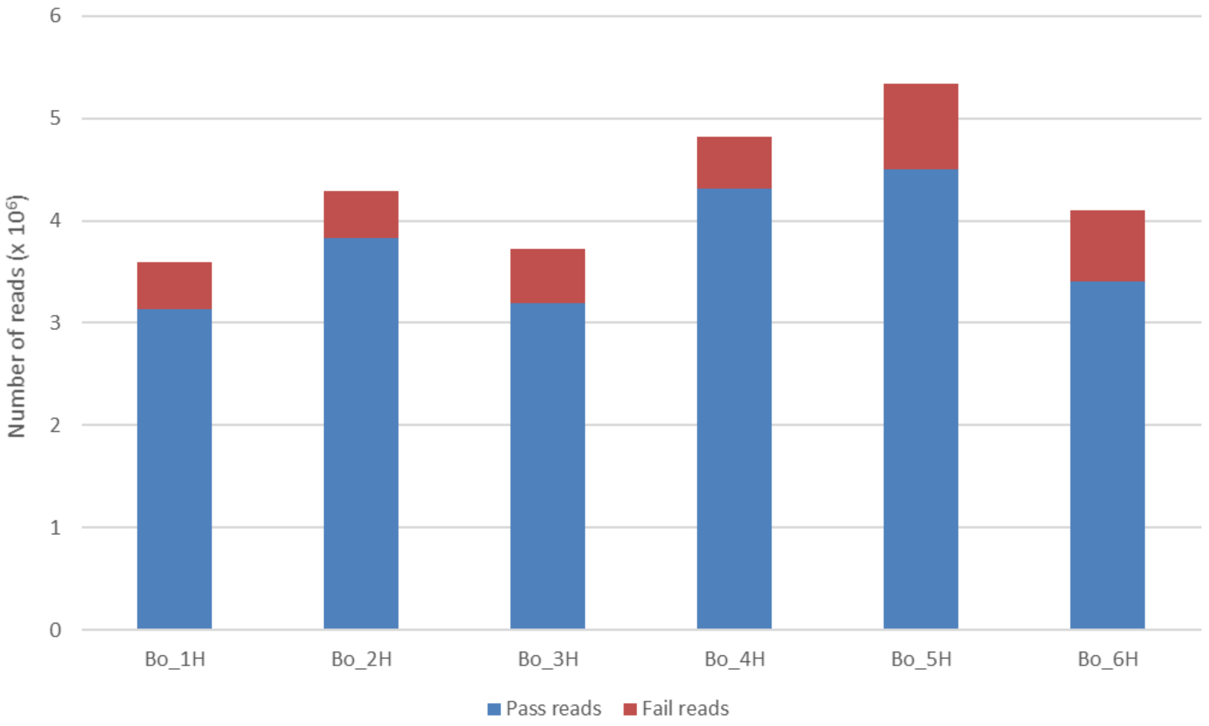

Extended Figure 5. Summary read stats from ONT sequencing of *B. oleae* embryo samples

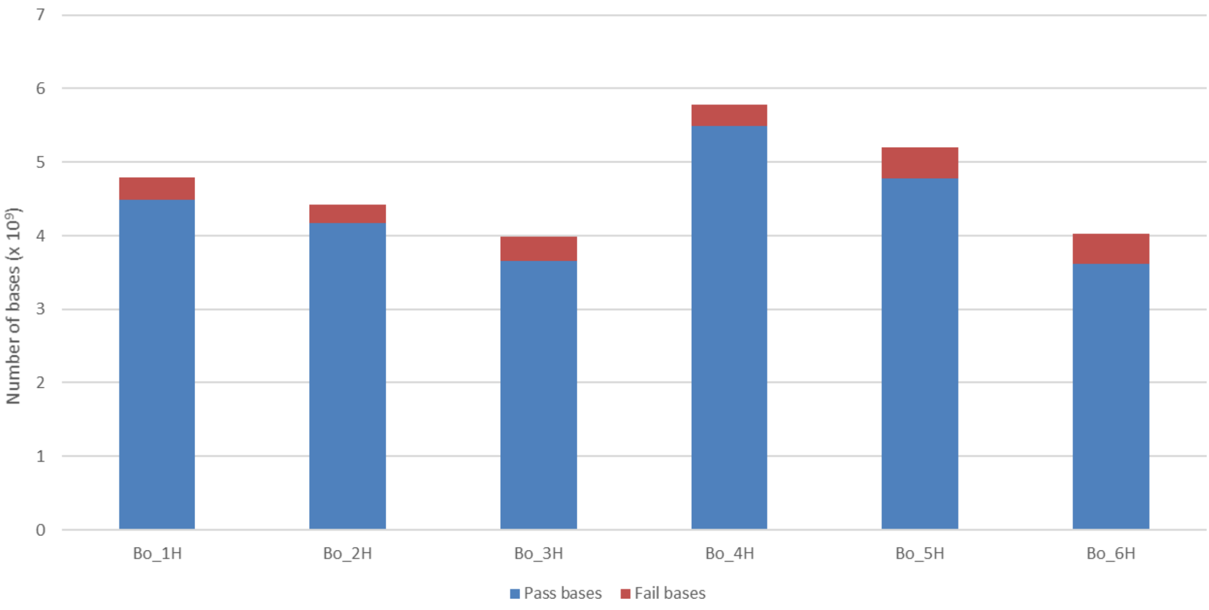

Extended Figure 6. Summary bases stats from ONT sequencing of *B. oleae* embryo samples

Extended Figure 7: Quality control profiles of Oxford Nanopore sequencing runs

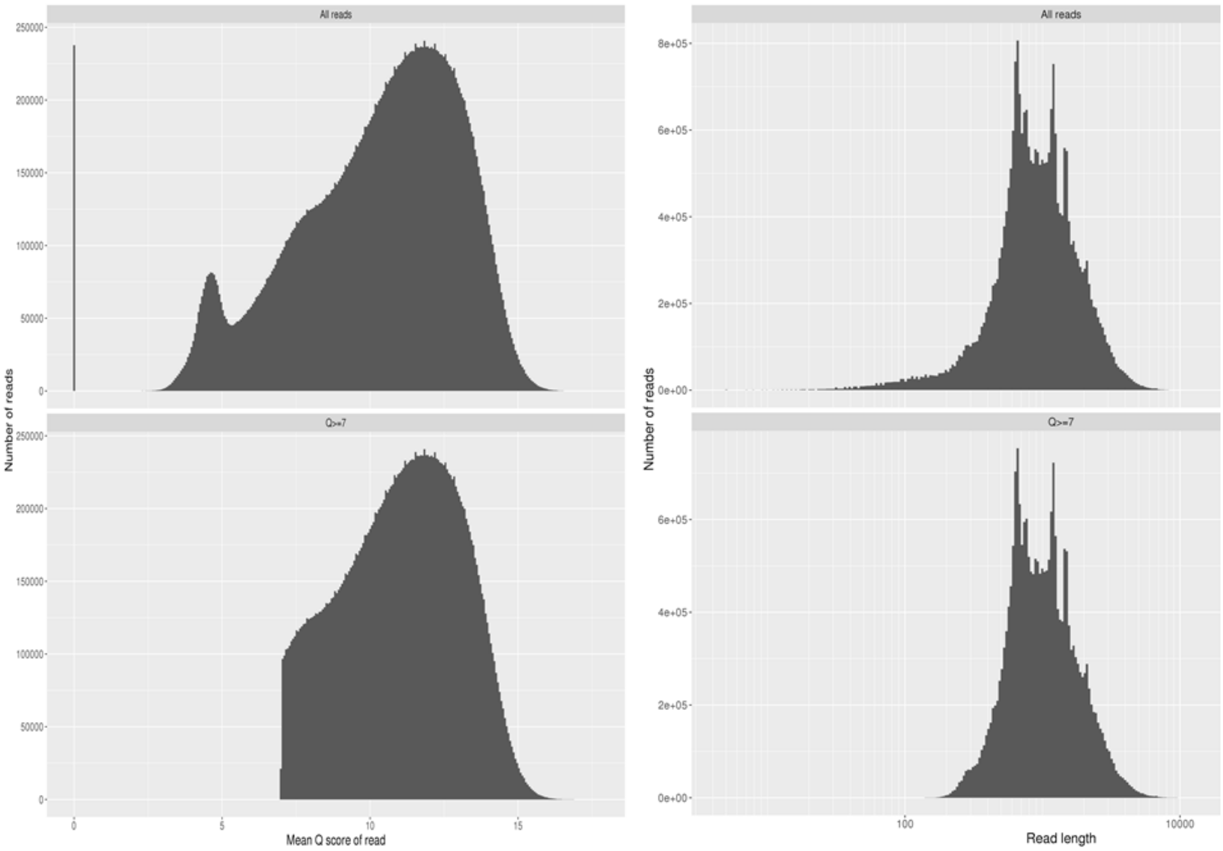

Extended Figure 7 A

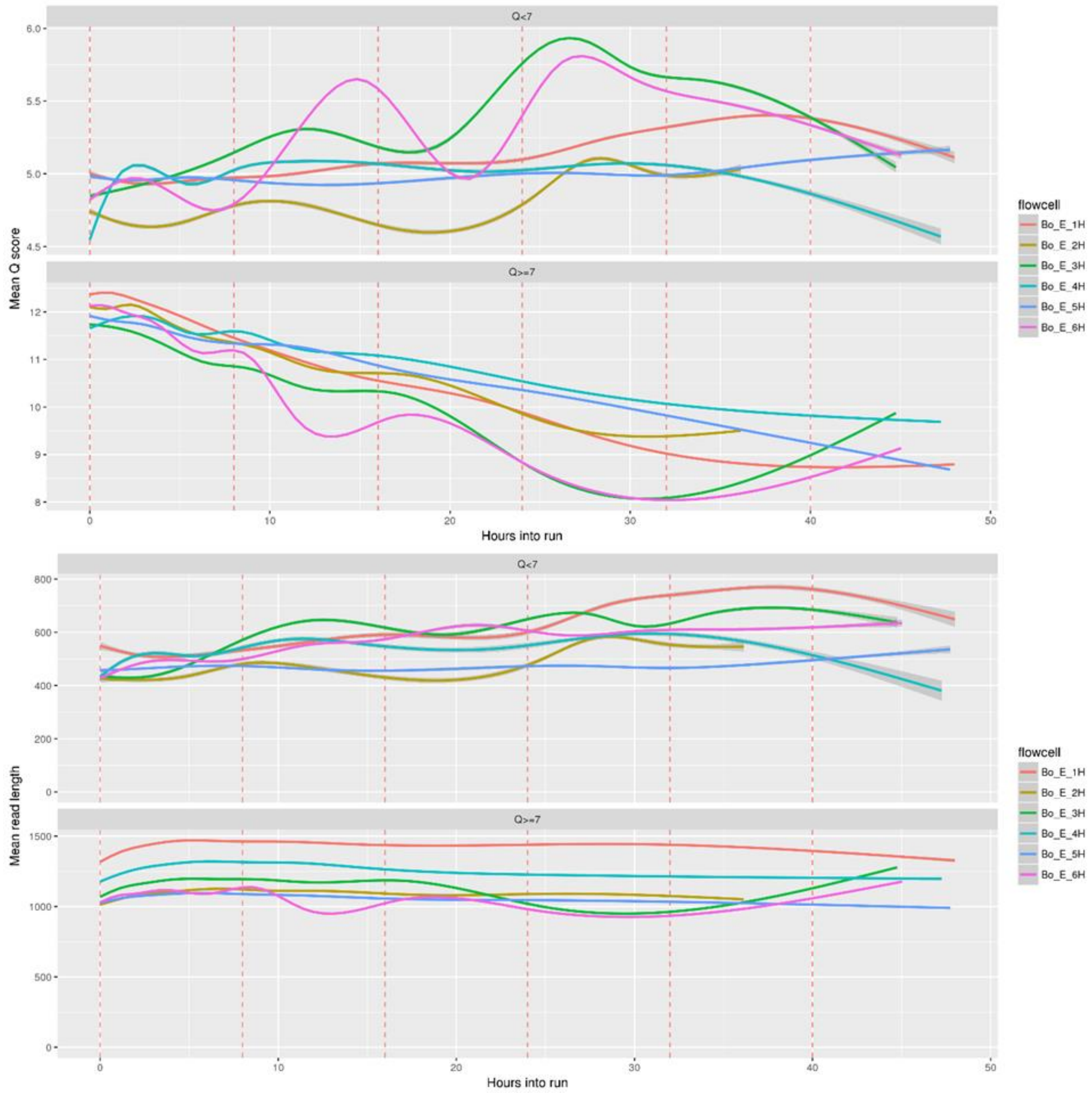

Extended Figure 7 B

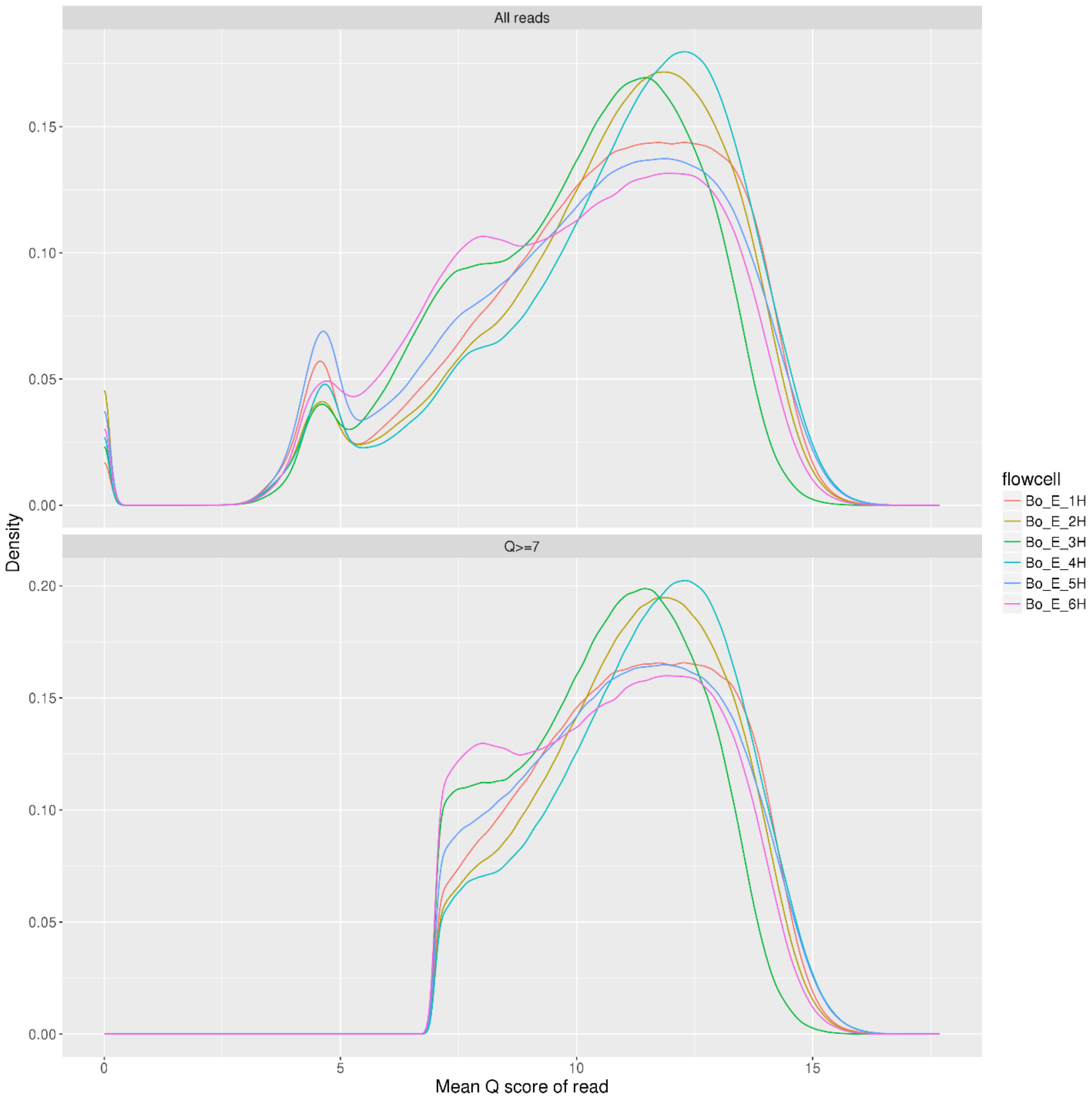

Extended Figure 7 C

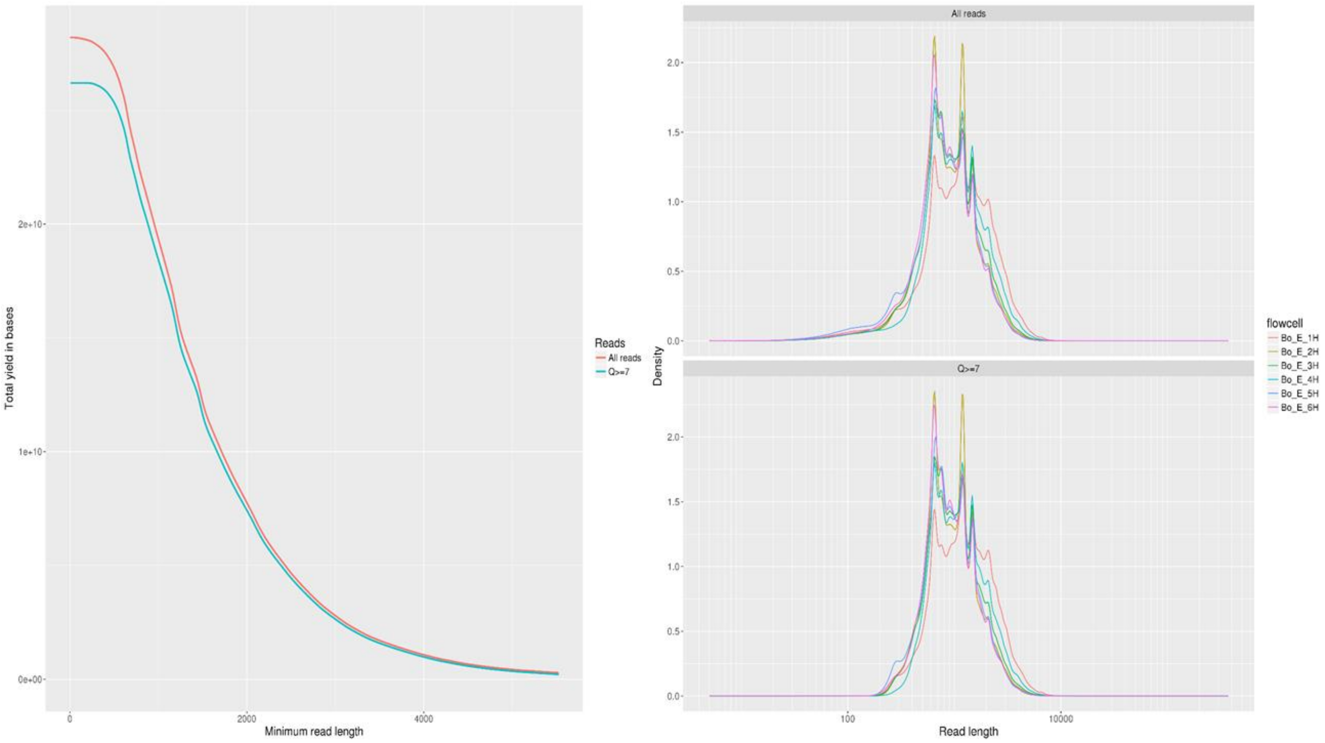

Extended Figure 7 D

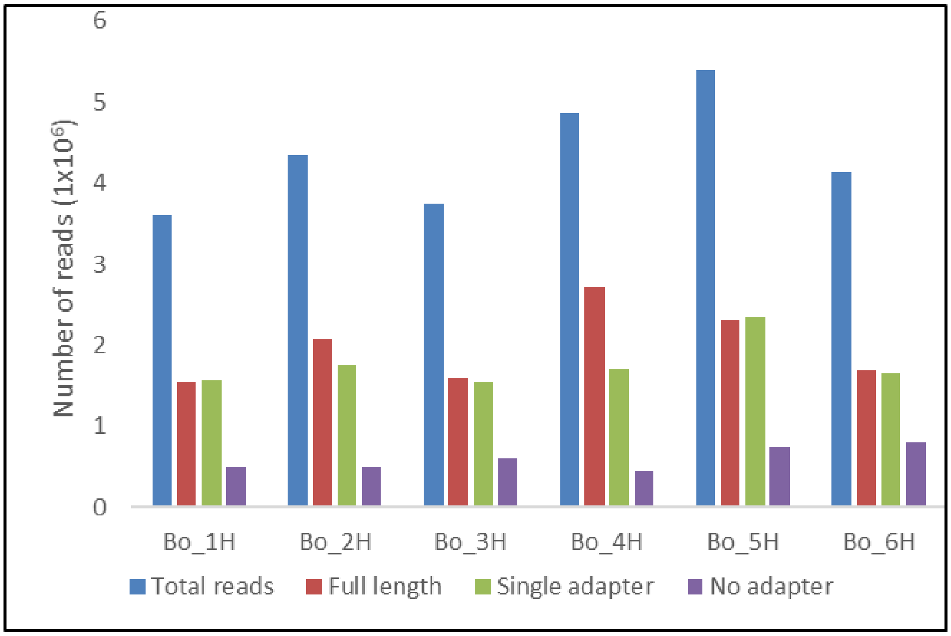

Extended Figure 8. Number of reads identified as full-length according to detection of 5' and 3' adapters and those with a single adapter or no adapter at all.

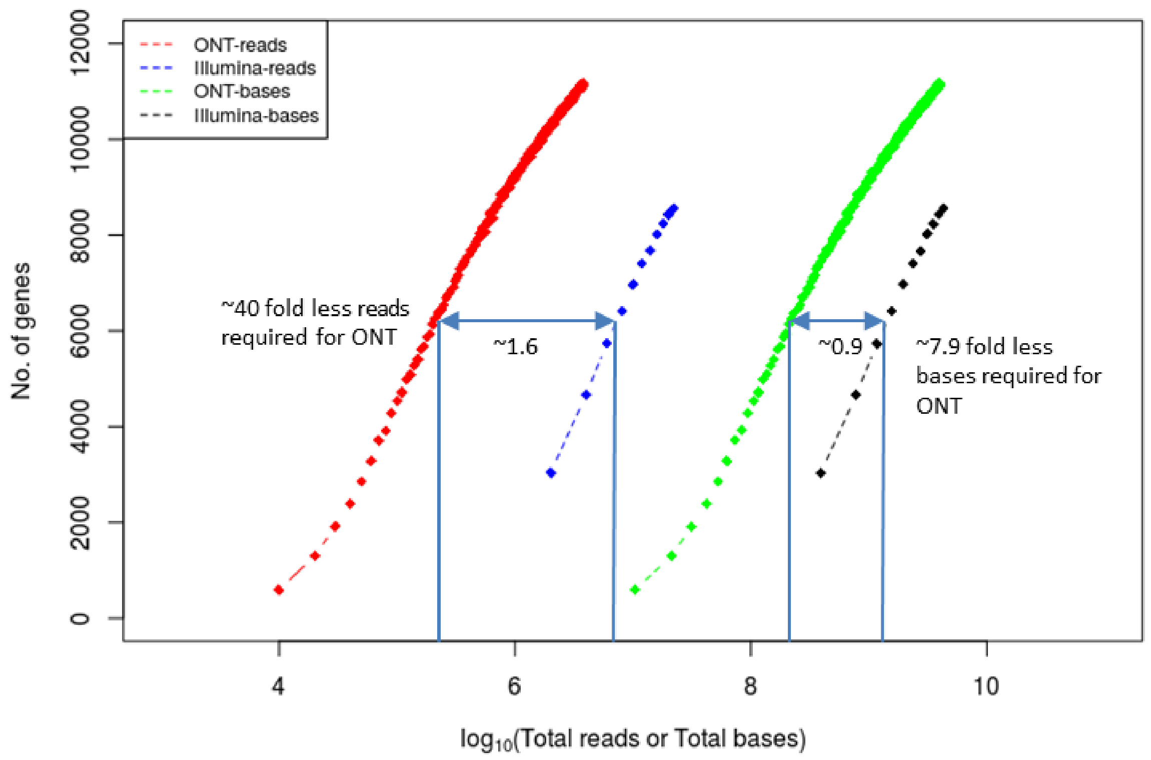

Extended Figure 9. Rare fraction curve comparing number of reads and bases required to observe the same number of genes between long-reads (Oxford Nanopore) and short-reads (Illumina).

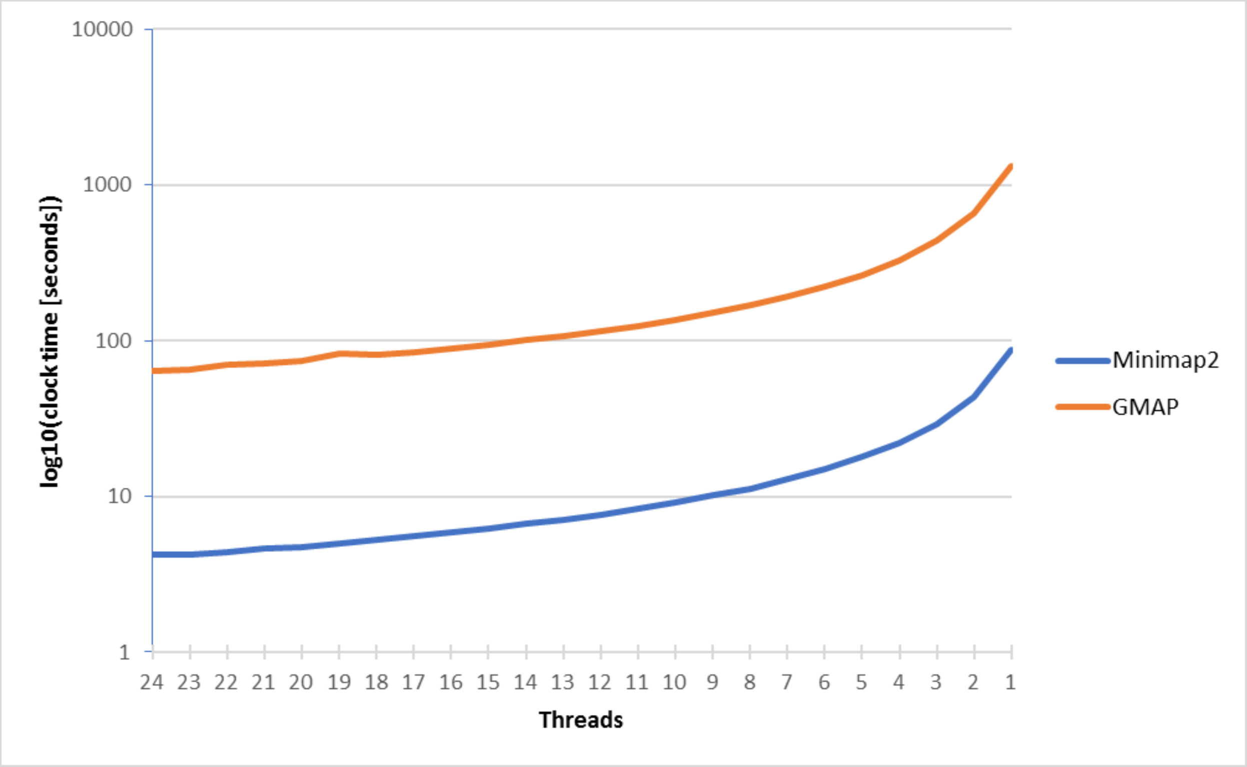

Extended Figure 10. Comparison of computational efficiency of GMAP and Minimap2. One million Nanopore long cDNA reads were aligned to the reference genome using GMAP or Minimap2 setting different processors (threads) and the amount of clock-time require to complete the time recorded. Both tools showed similar scaling with number of threads although Minimap2 showed exceptional speed in completing the jobs.

Extended Figure 11. In-depth characterization of B. oleae de novo transcriptome assembly with reference to the Bactrocera oleae Annotation release 100 from NCBI. Statistics derived using SQANTI.

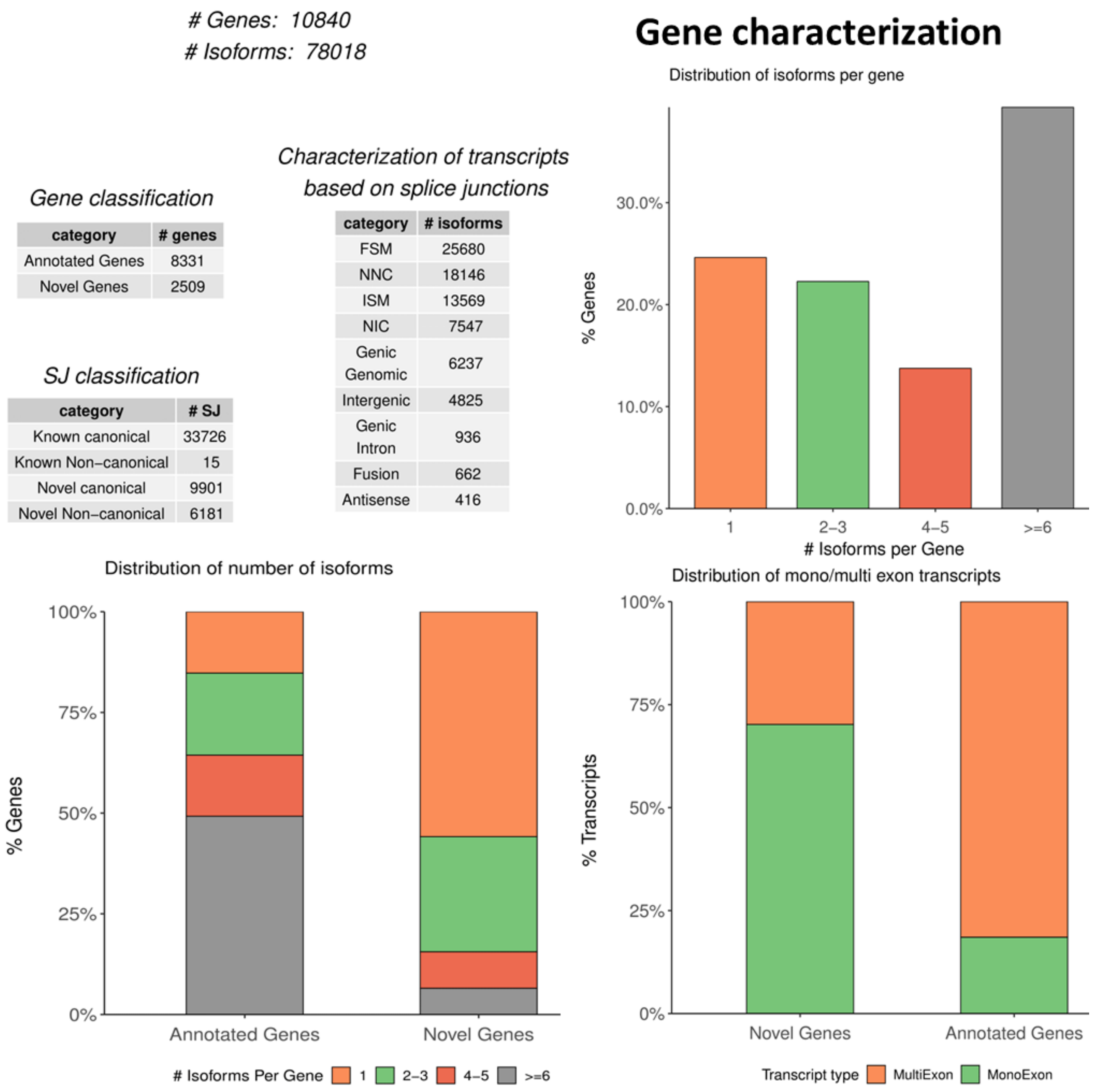

Gene classification

category	# genes
Annotated Genes	8331
Novel Genes	2509

SJ classification

category	# SJ
Known canonical	33726
Known Non–canonical	15
Novel canonical	9901
Novel Non–canonical	6181

Characterization of transcripts based on splice junctions

category	# isoforms
FSM	25680
NNC	18146
ISM	13569
NIC	7547
Genic Genomic	6237
Intergenic	4825
Genic Intron	936
Fusion	662
Antisense	416

Extended Figure 11 A

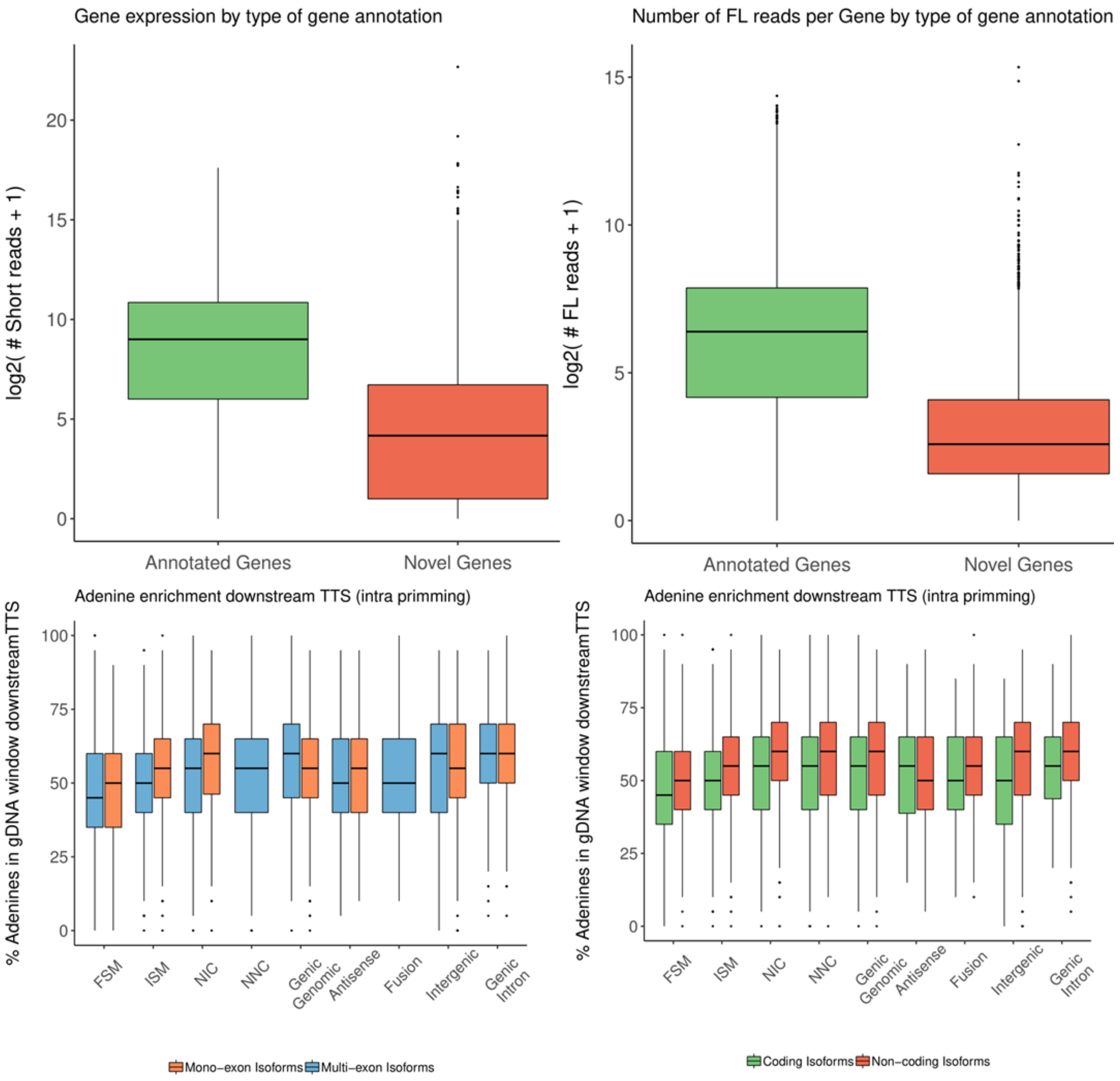

Extended Figure 11 B

Structural isoform characterization based on splice junctions

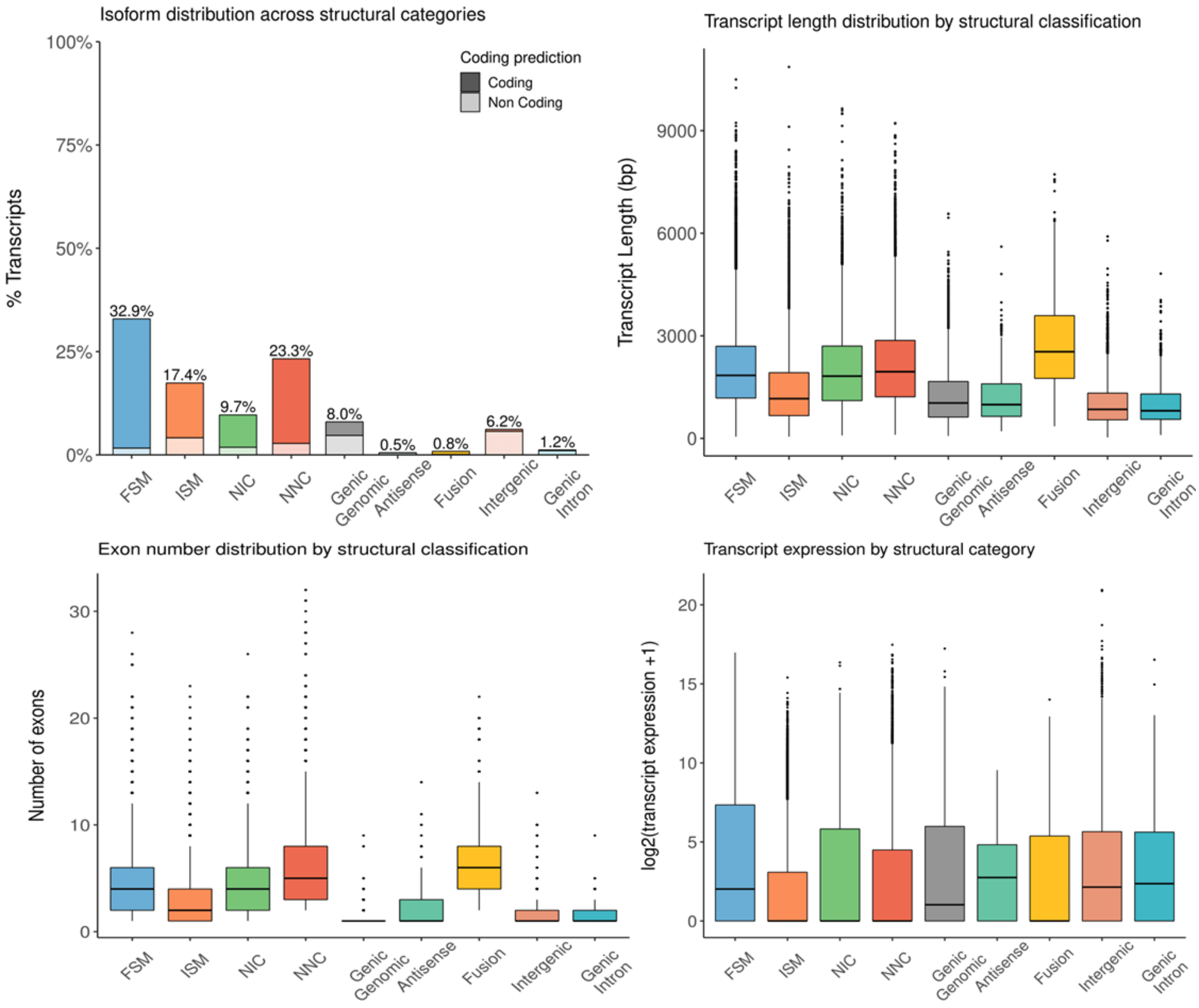

Extended Figure 11 C

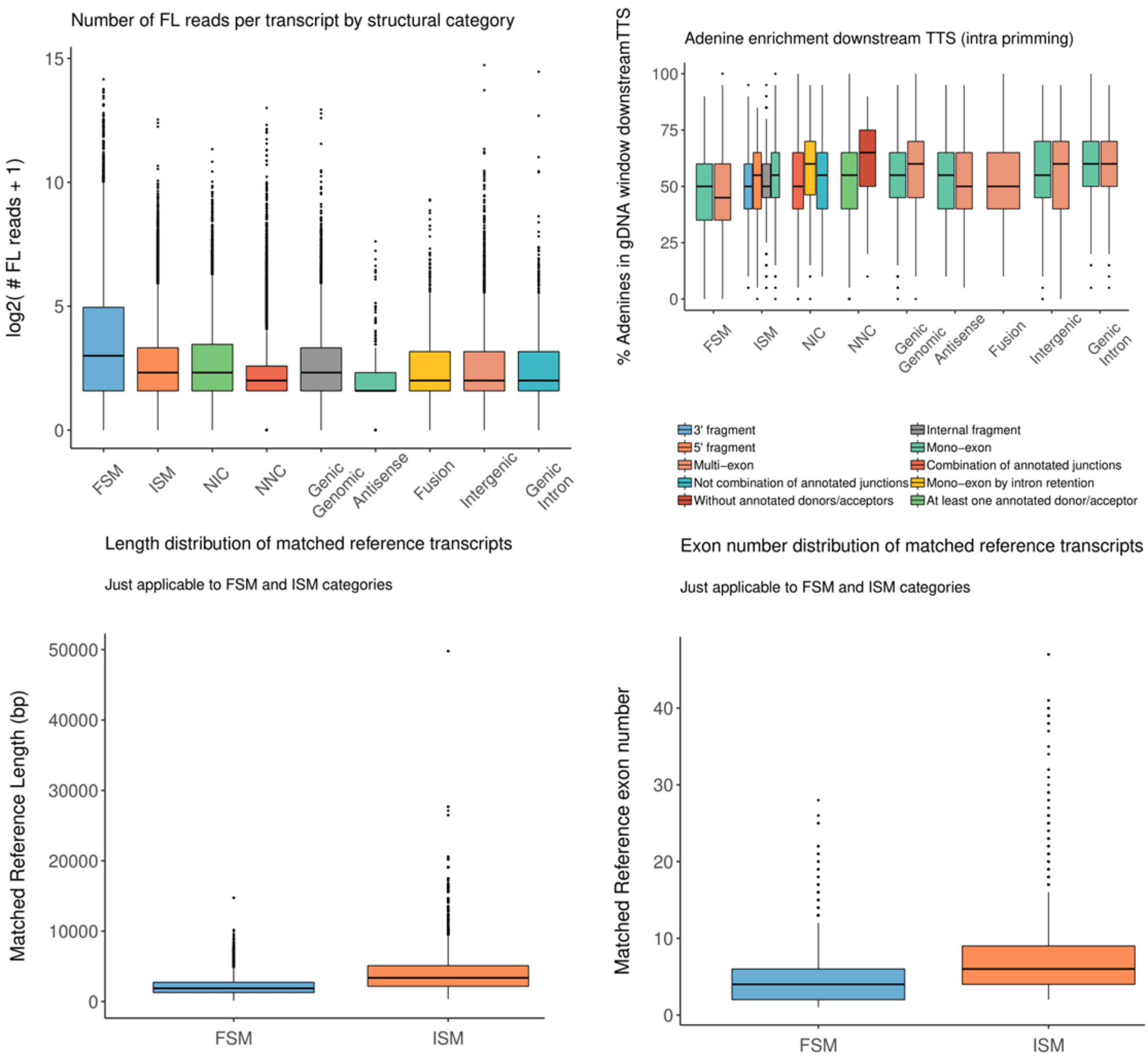

Extended Figure 11 D

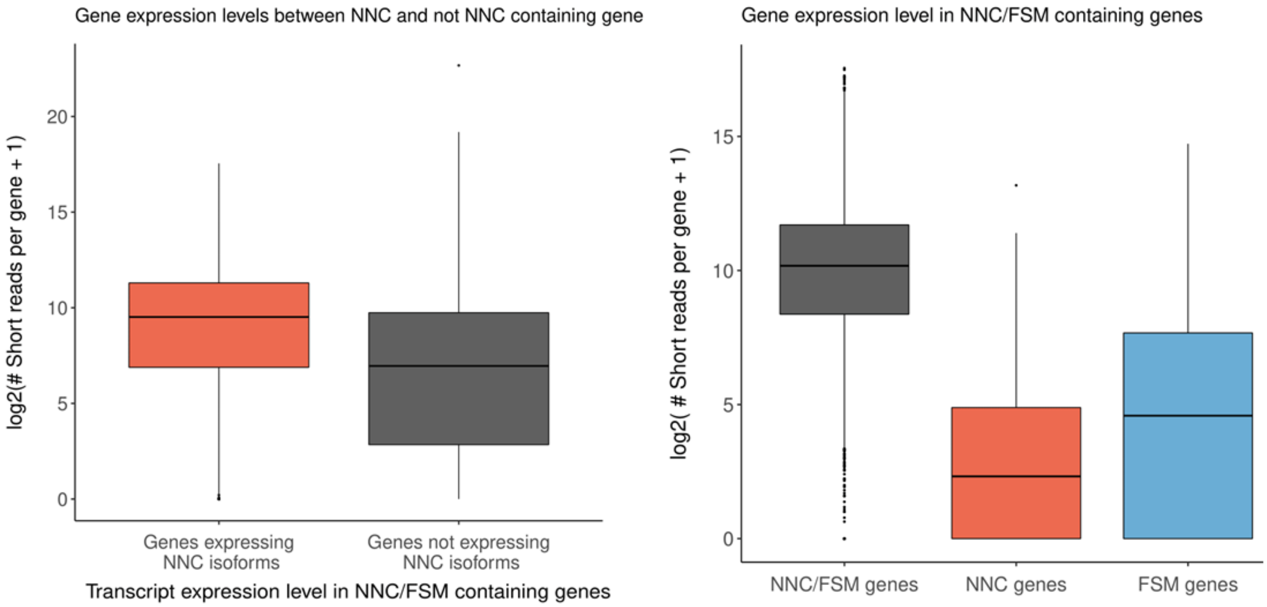

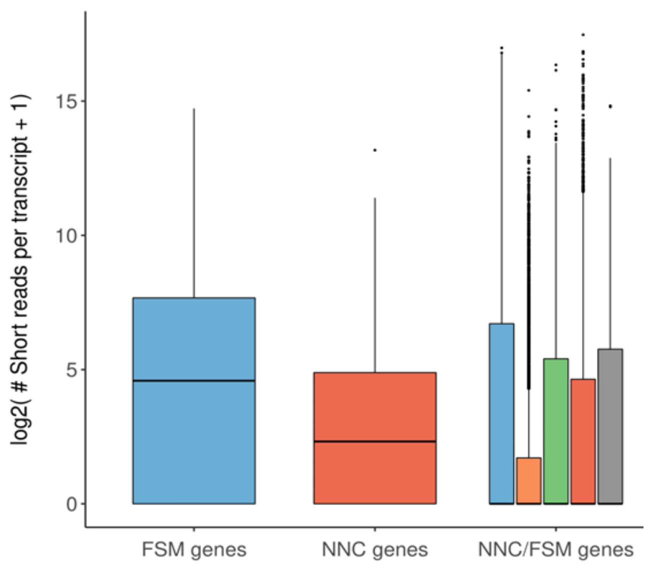

Splice junction characterization

Extended Figure 11 E

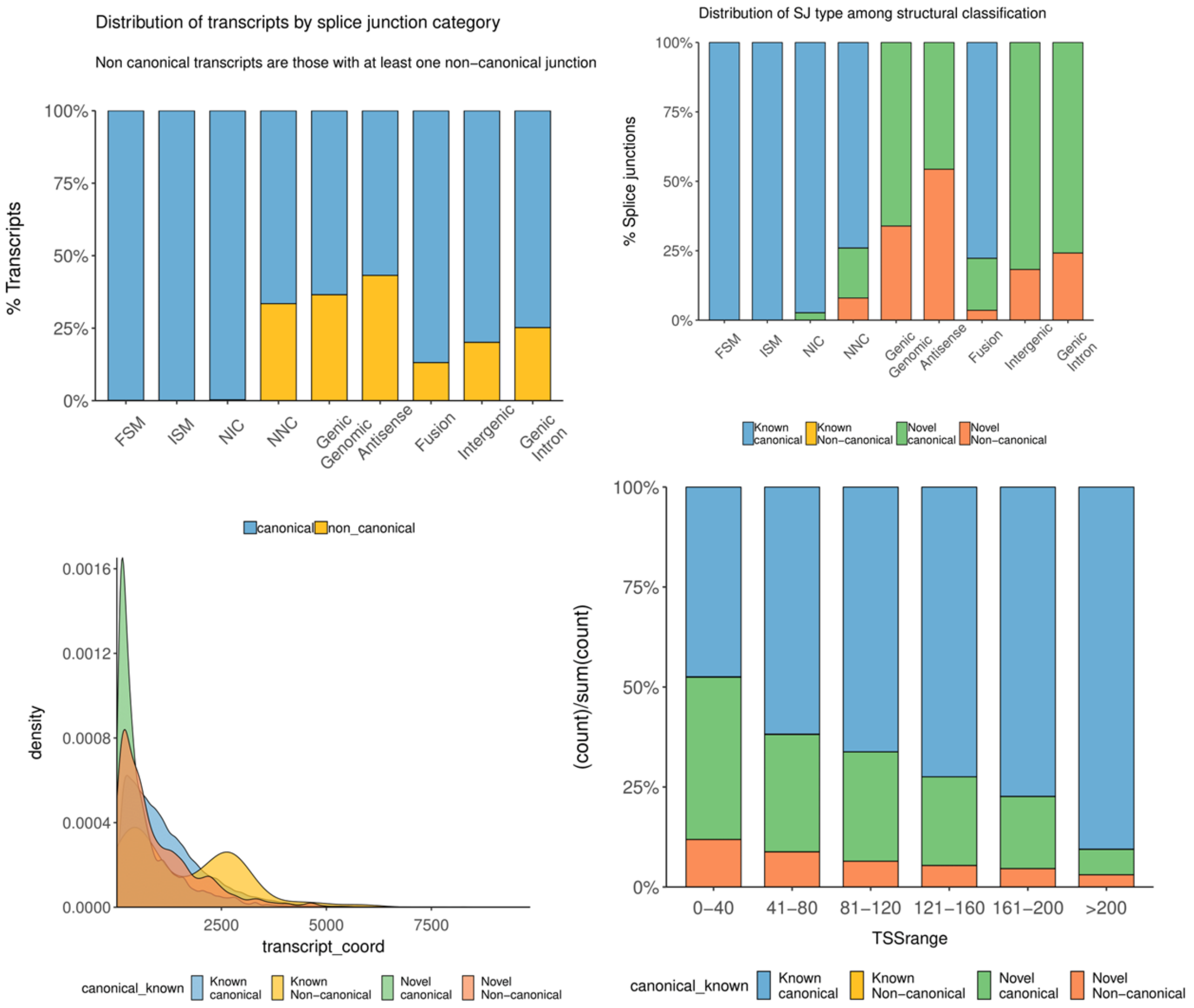

Extended Figure 11 F

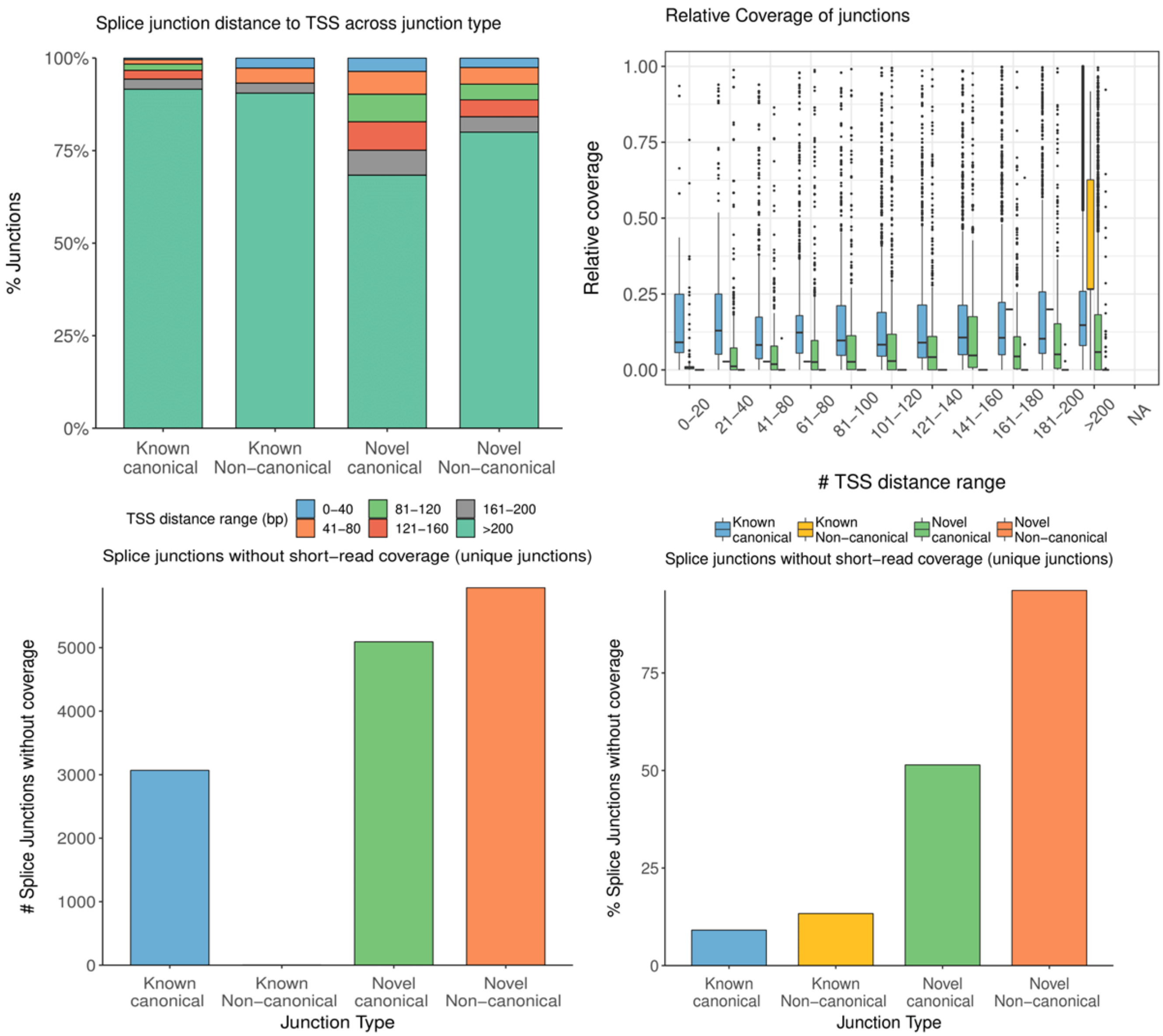

Extended Figure 11 G

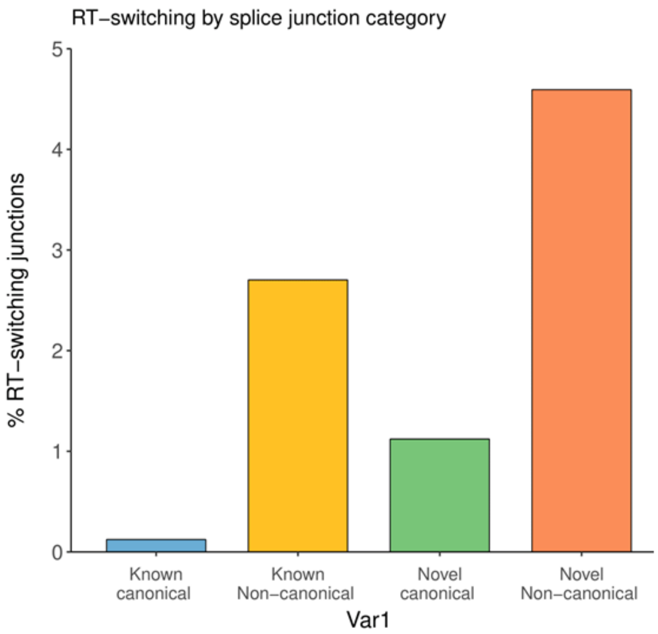

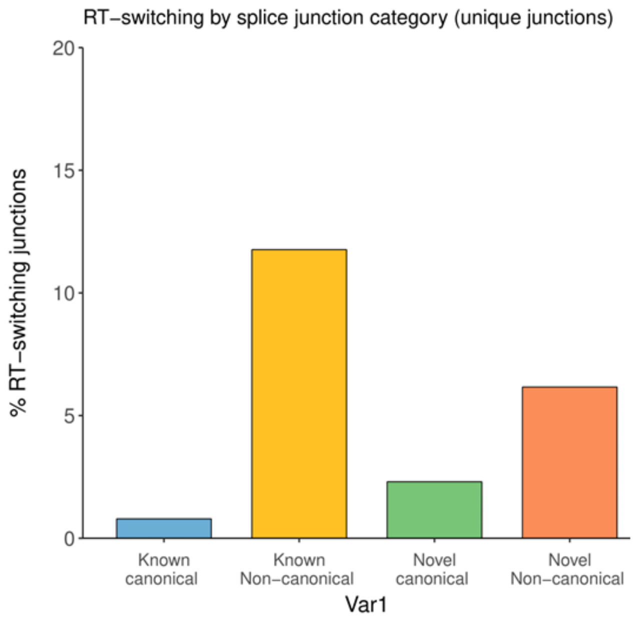

Full-lengthness characterization of isoforms

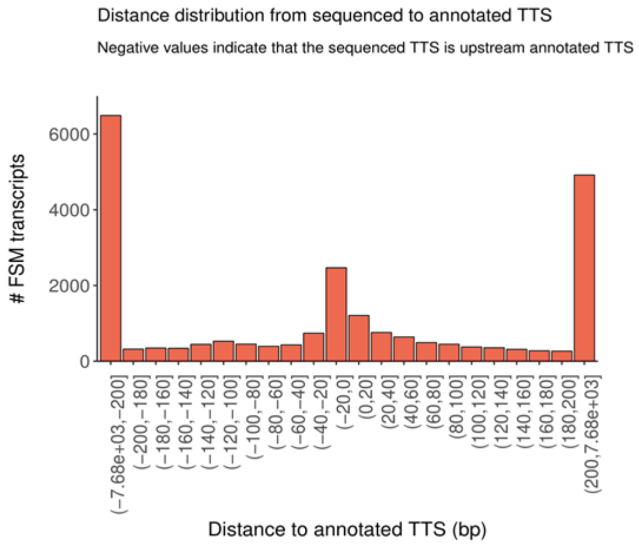

Extended Figure 11 H

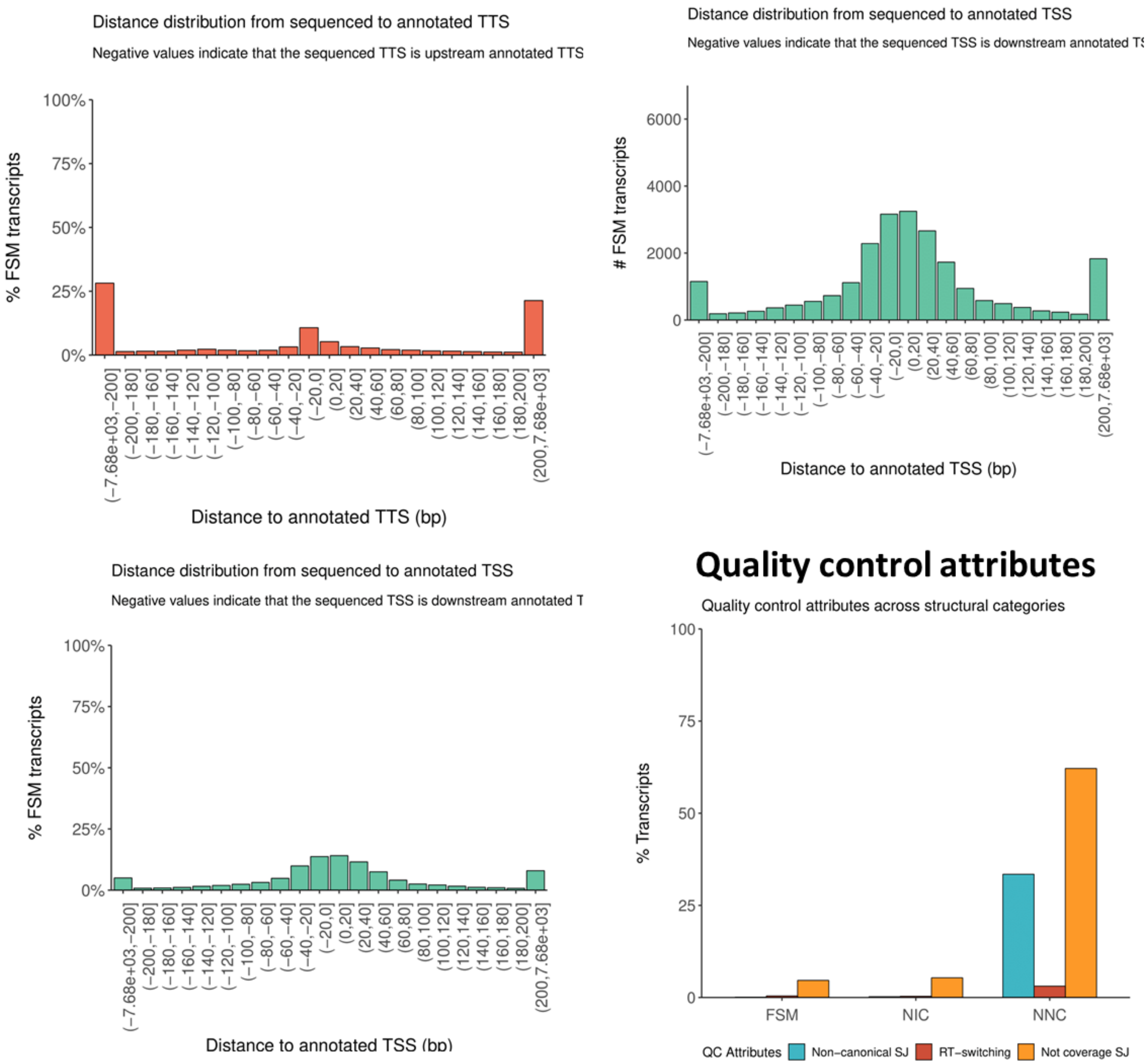

Extended Figure 11 I

Extended Figure 12. Alignment statistics of one of the samples. Results were generally similar across samples. Statistics generated using AlignQC

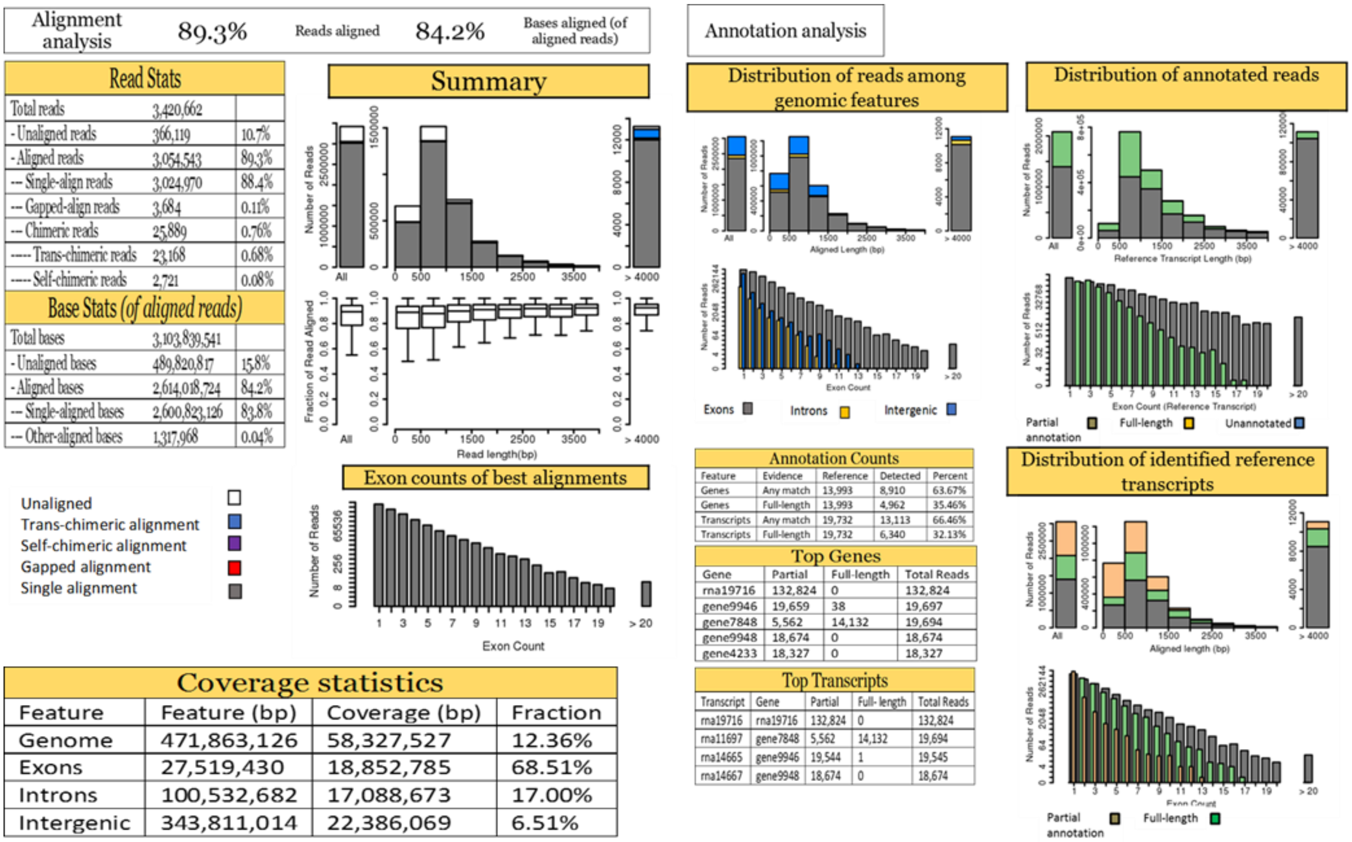

Extended Figure 12 A. Alignment QC (Using the 6-hour timepoint as example)

Coverage analysis	12.36%	Reference sequences covered		
Rarefraction analysis	8,910	Genes detected	4,962	Full-length genes

Gene detection rarefraction

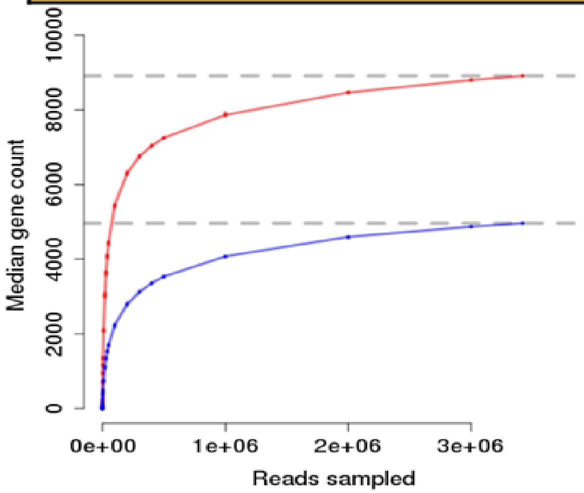

Transcript detection rarefraction

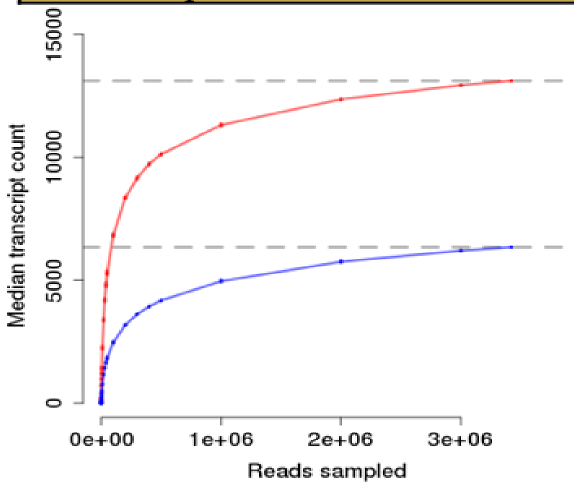

Any match ■ Full length ■
Vertical line height indicates 5%-95% CI of sampling

Rarefraction stats

Feature	Criteria	Count
Gene	full-length	4,962
Gene	any match	8,910
Transcript	full-length	6,340
Transcript	any match	13,113

Bias in alignment to reference transcripts

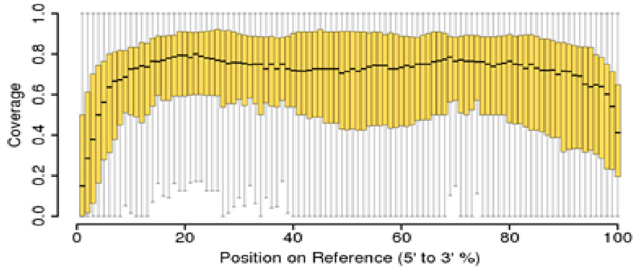

Annotated features coverage

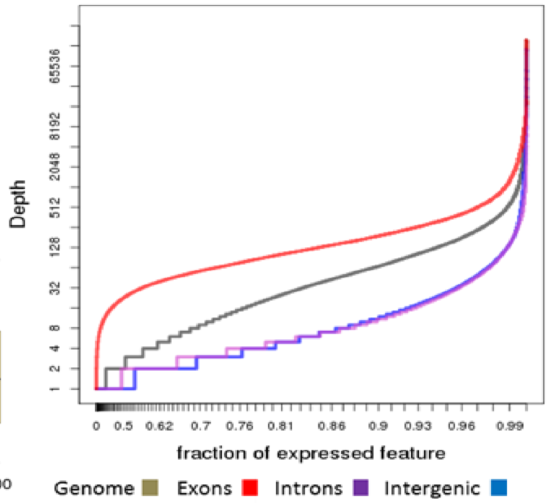

Extended Figure 12 B

Error pattern analysis 16.835% Error rate
based on aligned segments

Error rates, given a target sequence	Alignment-based error rates

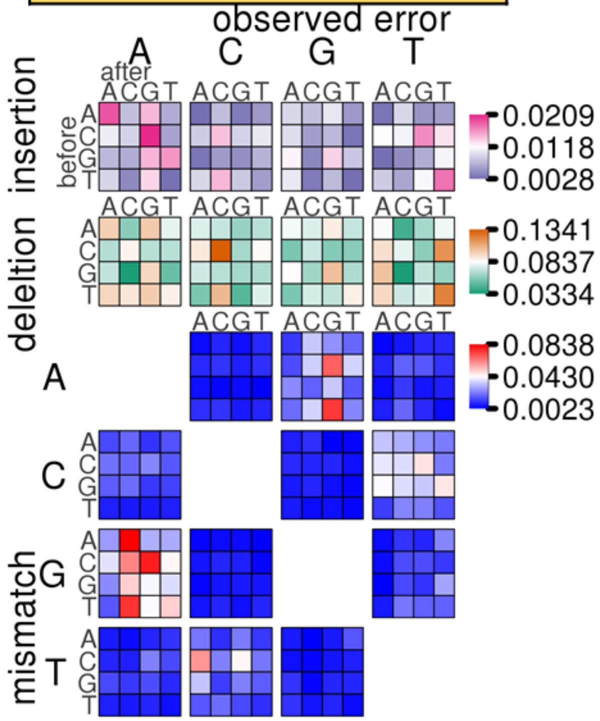

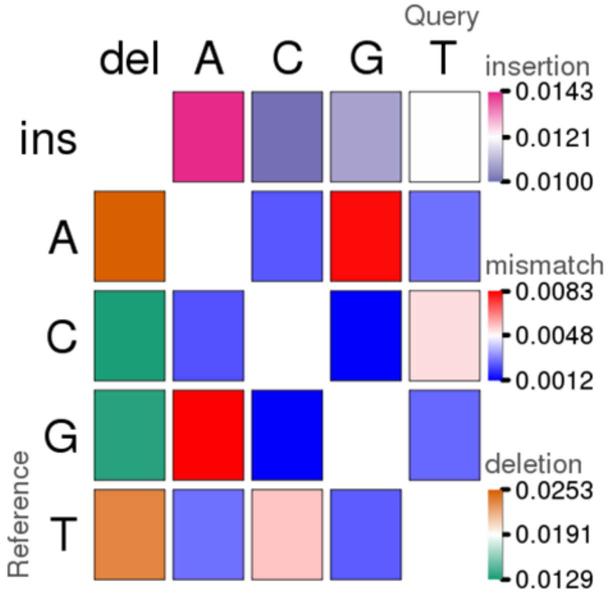

Alignment stats		
Best alignments sampled	1101	
**Base stats**		
Bases analyzed	1,060,762	
- Correctly aligned bases	882,179	83.2%
- Total error bases	178,583	16.835%
--- Mismatched bases	48,409	4.564%
--- Deletion bases	80,111	7.552%
----- Complete deletion bases	61,366	5.785%
----- Homopolymer deletion bases	18,745	1.767%
--- Insertion bases	50,063	4.720%
----- Complete insertion bases	32,343	3.049%
----- Homopolymer insertion bases	17,720	1.670%

Distance of observed junctions from reference junctions

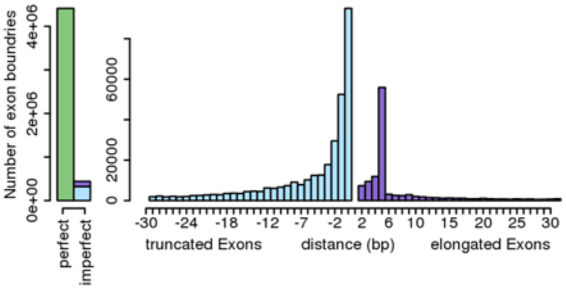

Extended Figure 12 C

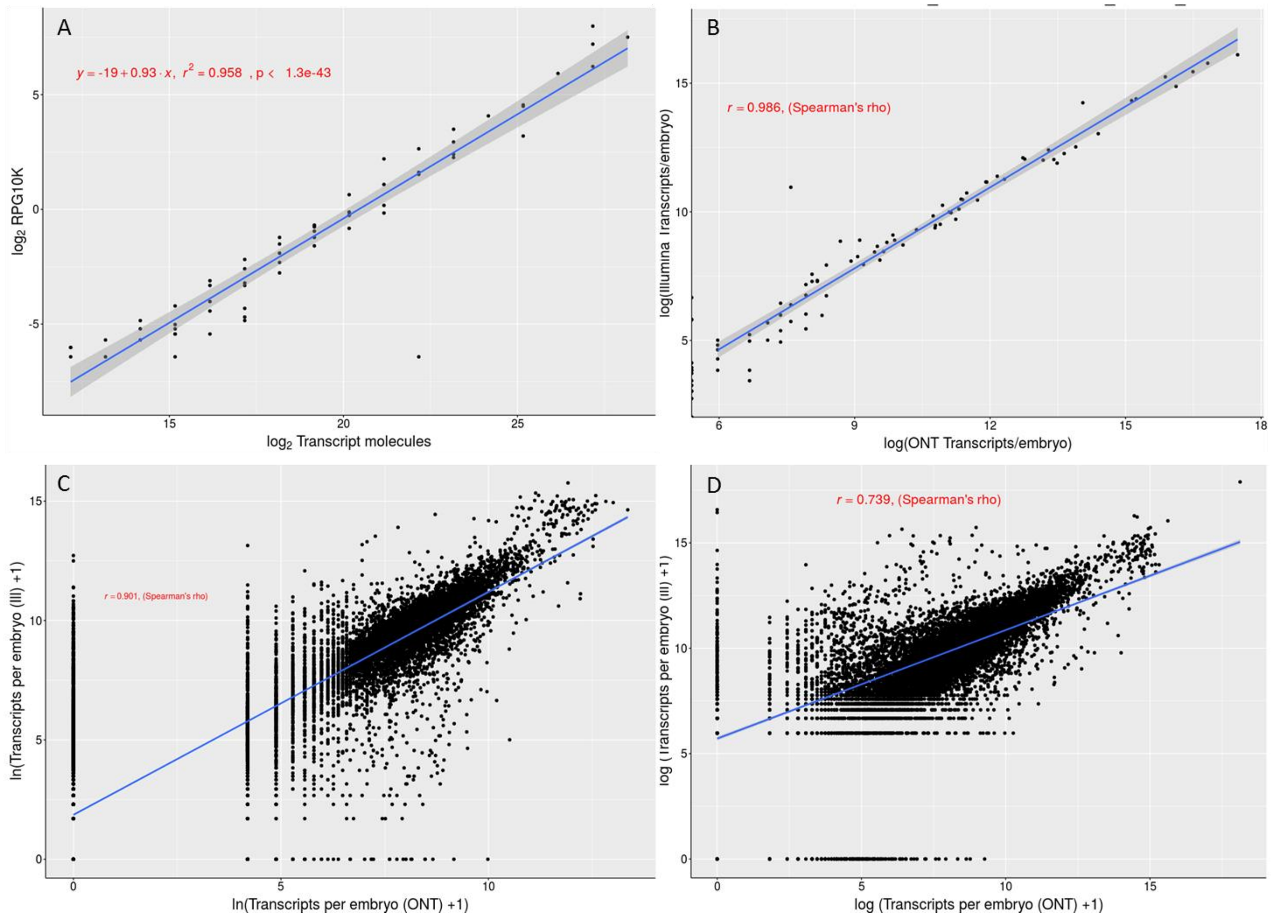

Extended Figure 13. A) Correlation of Oxford Nanopore Technologies (ONT) relative quantification of reads obtained from ERCC Spike-in RNA internal standards to the expected quantities. Relative quantification was achieved using Mandalorina and reported as reads per gene per 10000 mapped reads (RPG10K). **B**) Correlation of Illumina short-read and ONT long-read absolute quantification of ERCC Spike-in RNA internal standards. **C**) Correlation of Illumina short-read and ONT long-read quantification of annotate genes for the reads obtained from embryos at 5 hours after egg laying (AEL). **D**) same as 'C' but for the reads obtained from embryos at 6 hours AEL

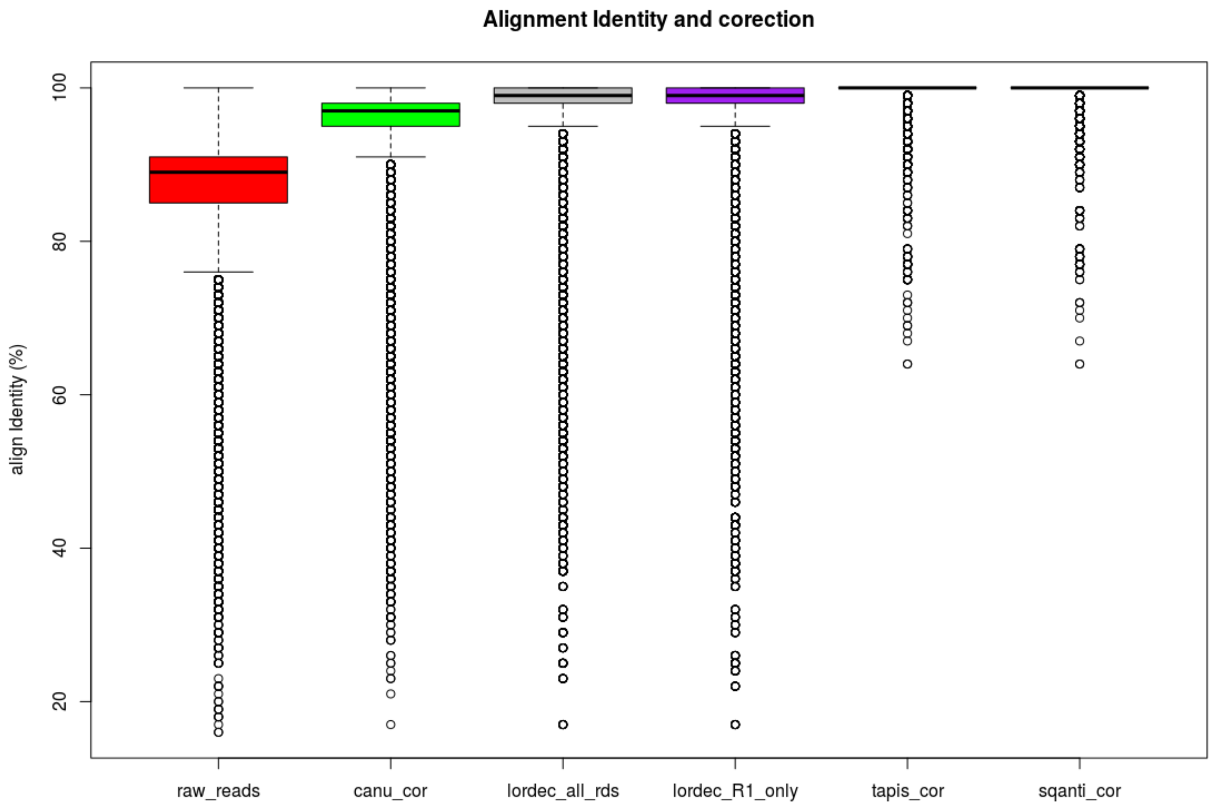

Extended Figure 14. Comparison of alignment identity following error correction. Raw_reads refers to the raw Nanopore cDNA reads. canu_cor refers to raw reads after one round of Canu correction. lordec_all_rds refers to Canu-corrected reads after one round of Lordec correction using read1 and read2 of Illumina short reads. Lordec_R1_only refers to Canu-corrected reads after one round of Lordec correction using only read1 of Illumina short reads. tapis_cor and sqanti_cor refer to reads after correction with TAPIS and SQANTI. TAPIS and SQANTI performed 3 rounds of genome guided error correction.

Extended Figure 16. Temporal clustering of gene expression using DGGP

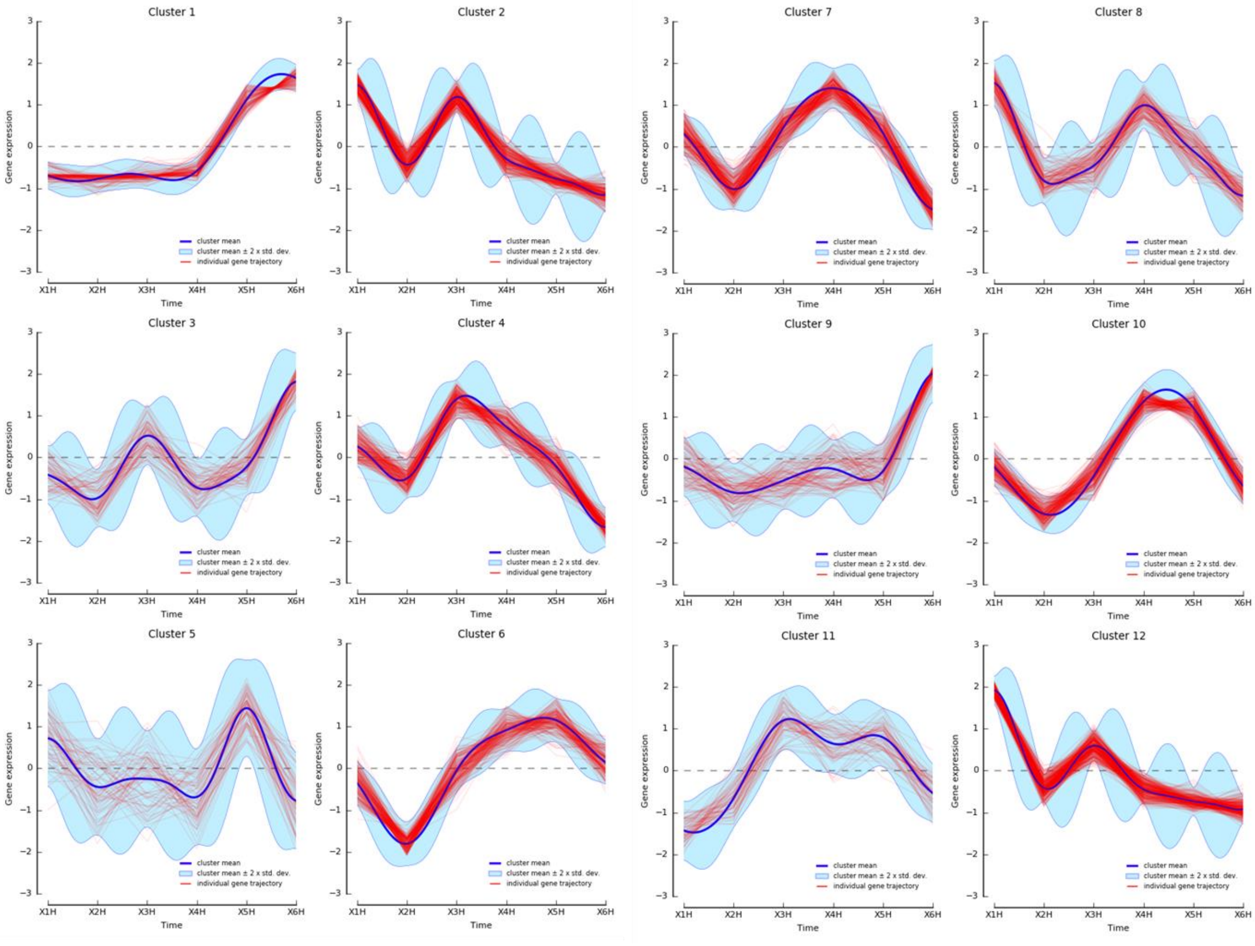

Extended Figure 15 A. Clustered trajectories of expressed genes across the early embryonic development of *B. oleae.*

(picture continuous in the next page)

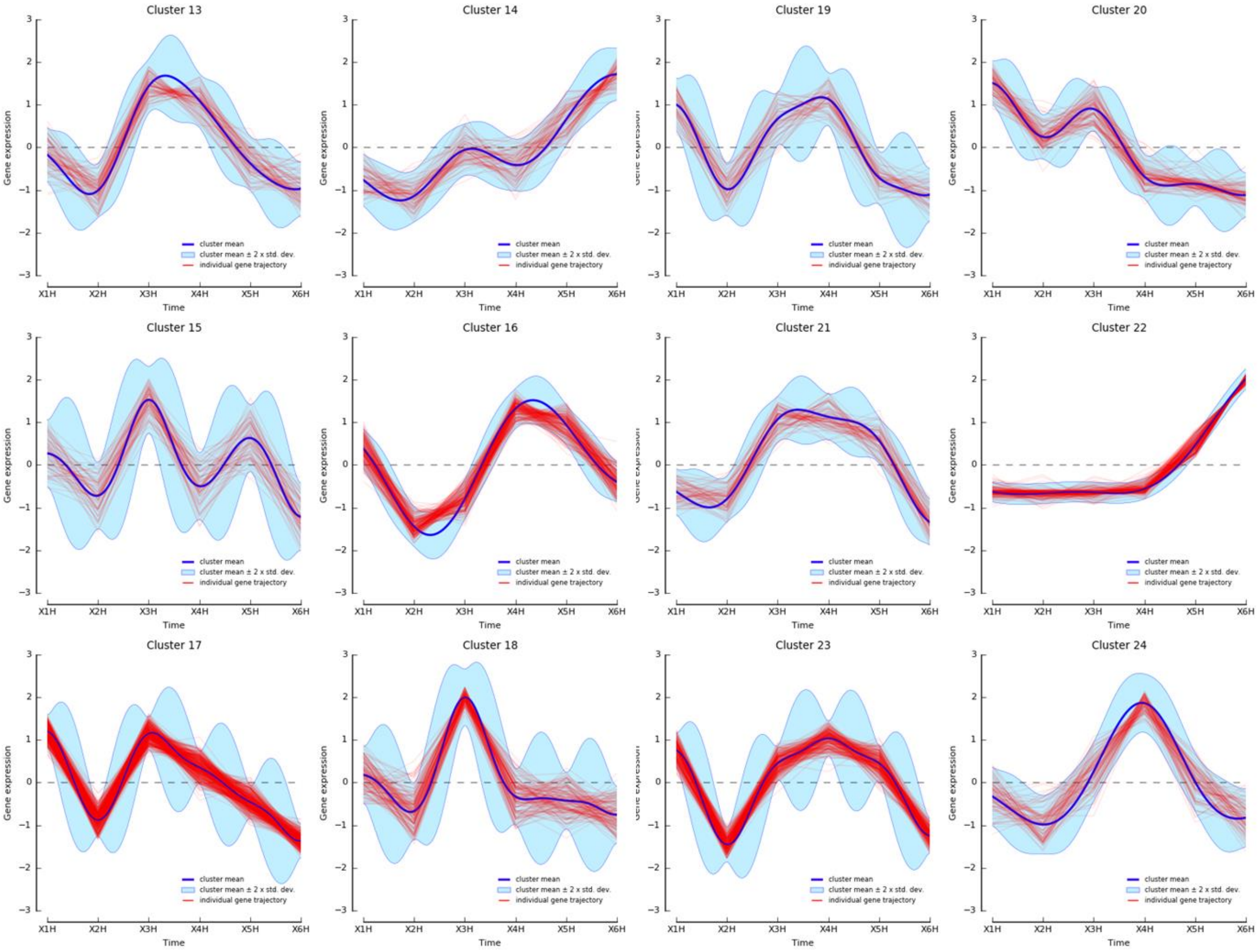

Extended Figure 16 B. Clustered trajectories of expressed genes across the early embryonic development of *B. oleae*.

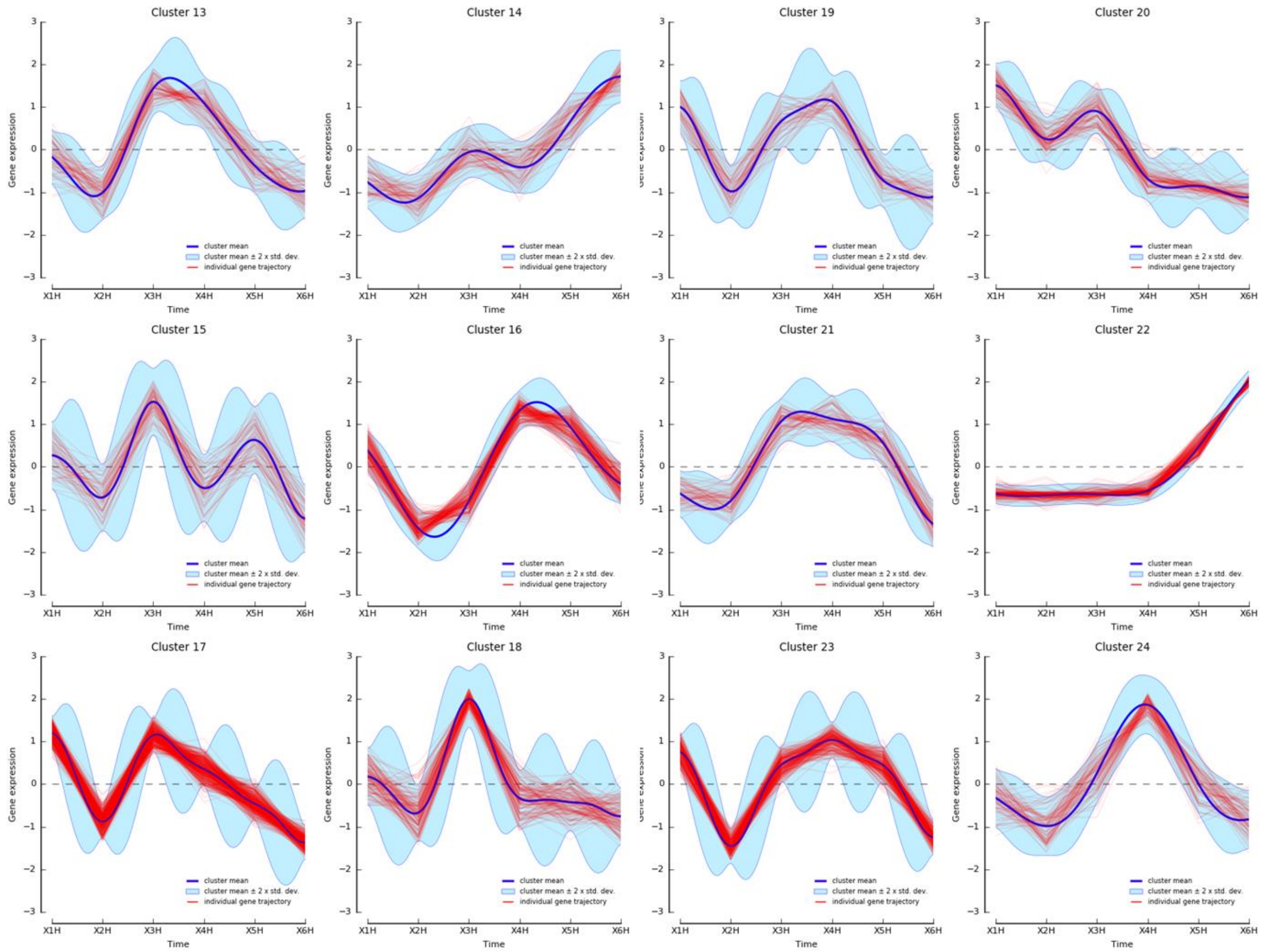

Extended Figure 16 C. Clustered trajectories of expressed genes across the early embryonic development of *B. oleae*.

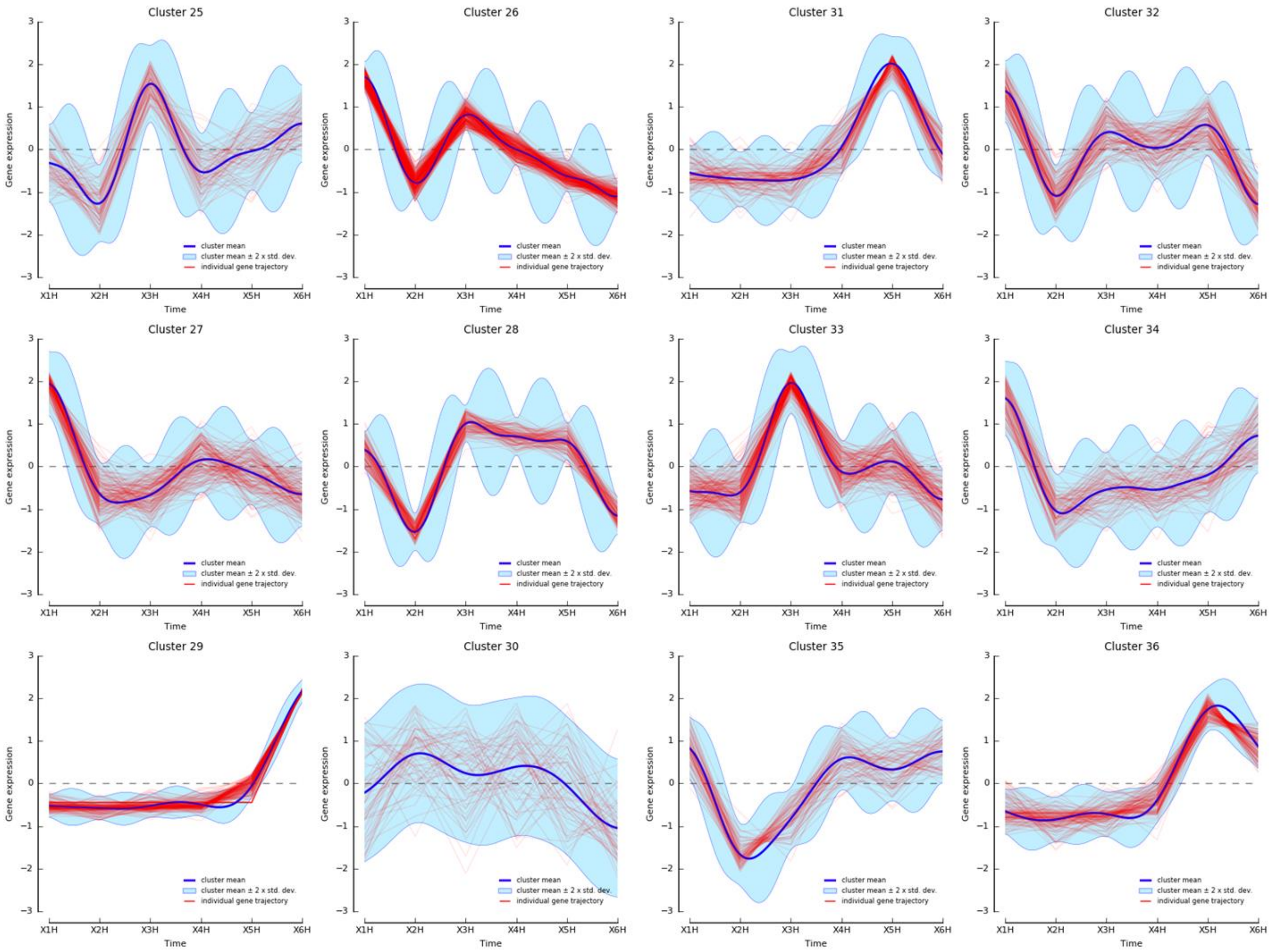

Extended Figure 16 D. Clustered trajectories of expressed genes across the early embryonic development of *B. oleae*.

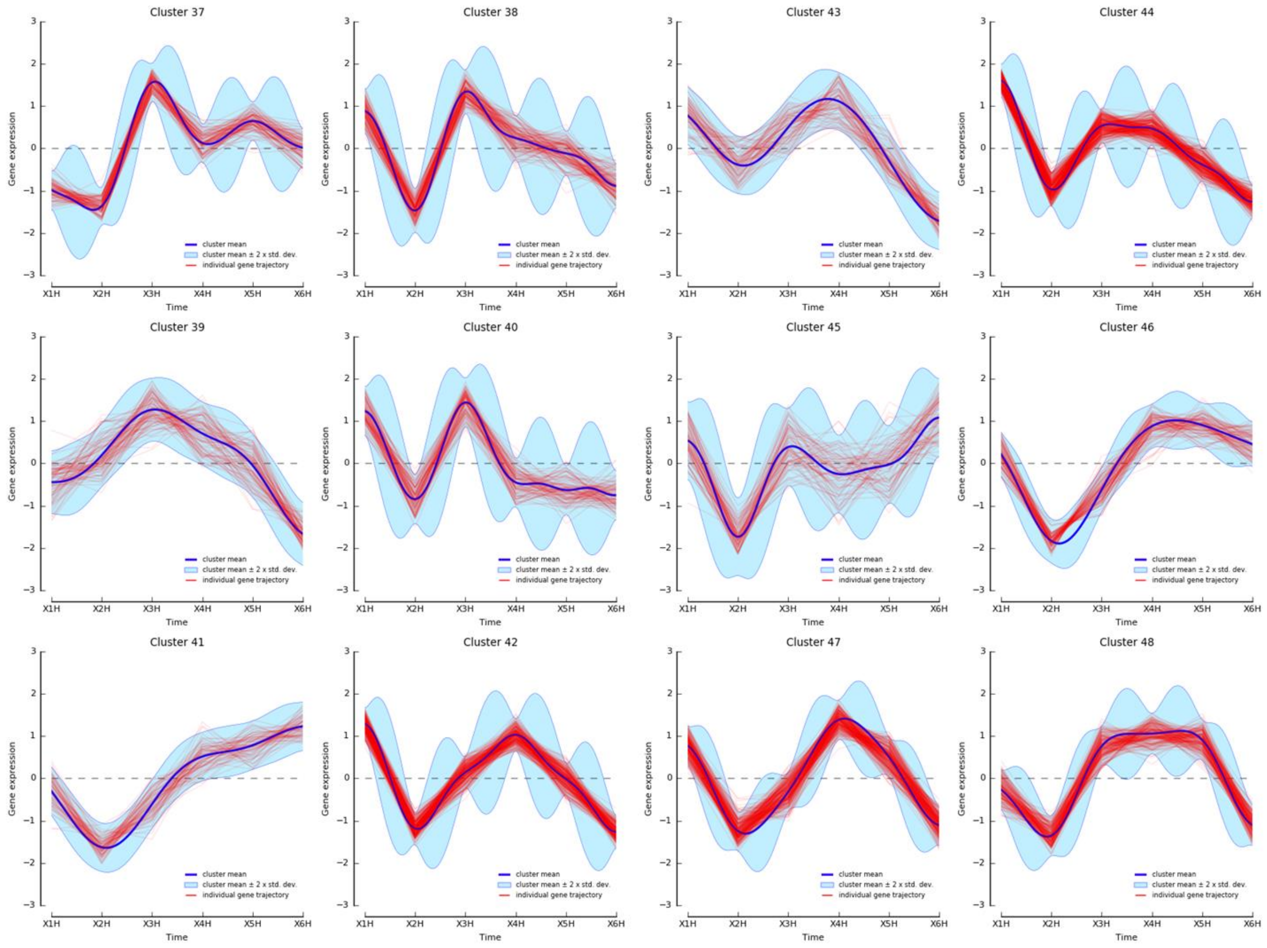

Extended Figure 16 E. Clustered trajectories of expressed genes across the early embryonic development of *B. oleae*.

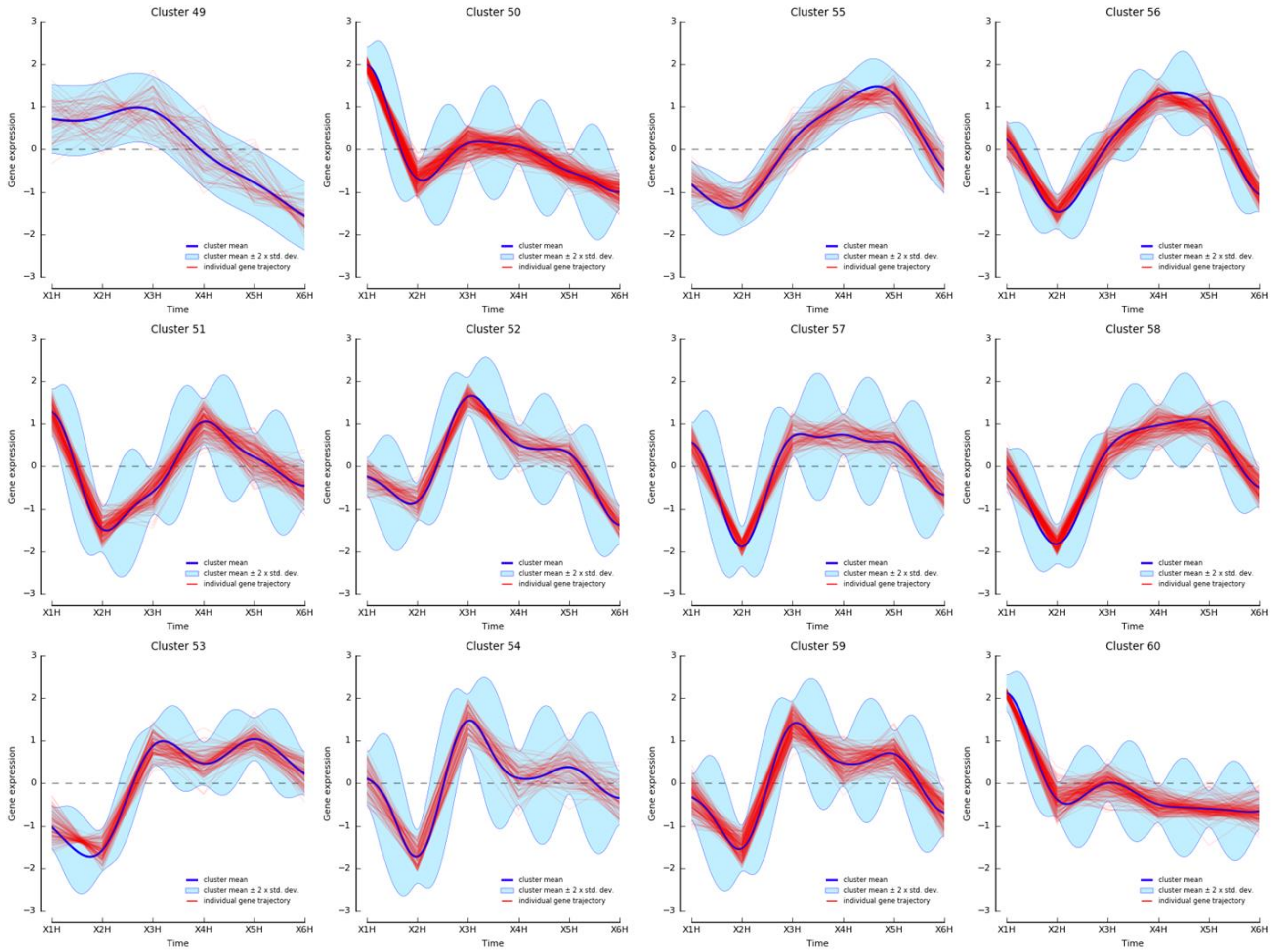

Extended Figure 16 F. Clustered trajectories of expressed genes across the early embryonic development of *B. oleae*.

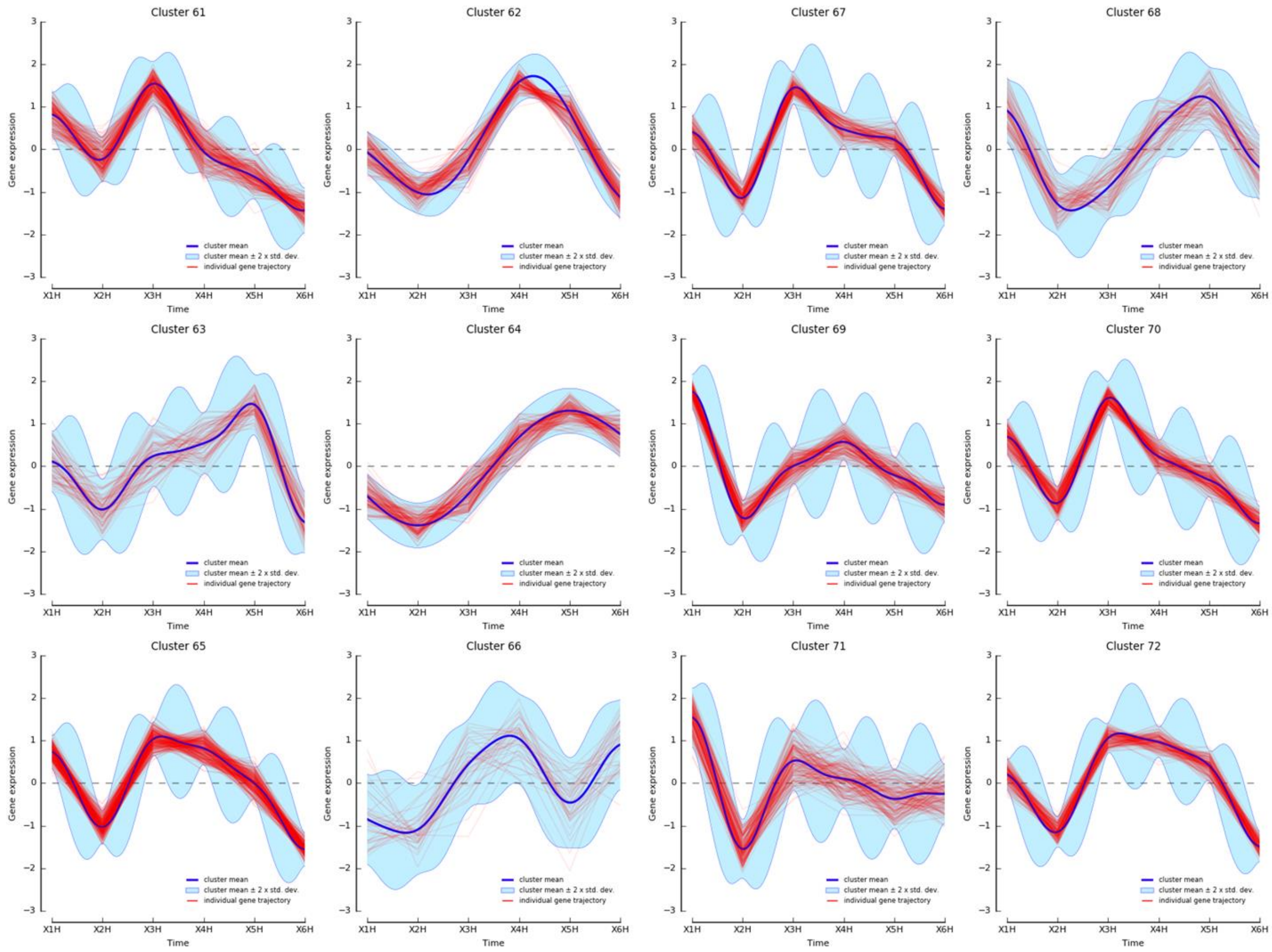

Extended Figure 16 G. Clustered trajectories of expressed genes across the early embryonic development of *B. oleae*.

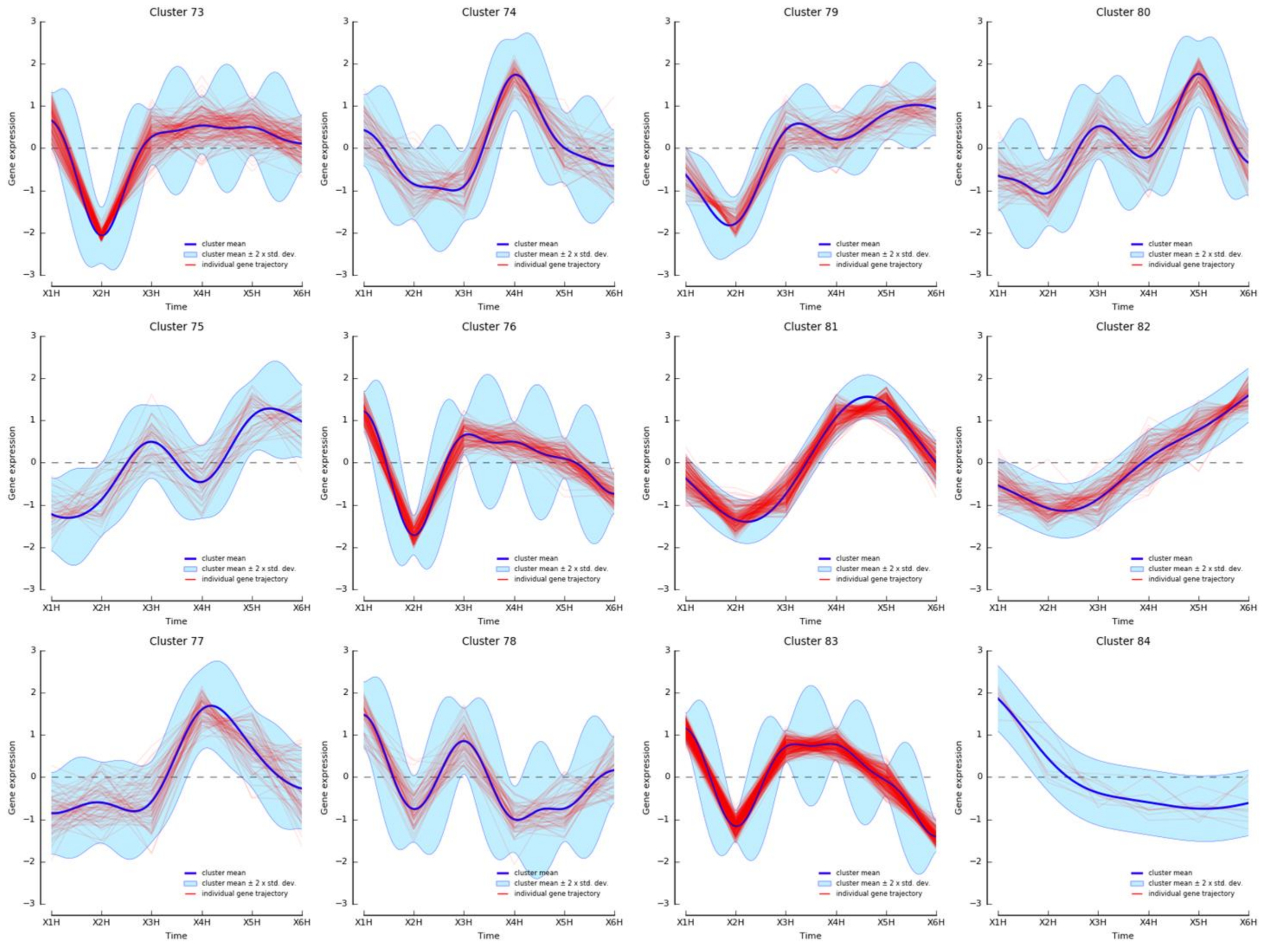

Extended Figure 16 H. Clustered trajectories of expressed genes across the early embryonic development of *B. oleae*.

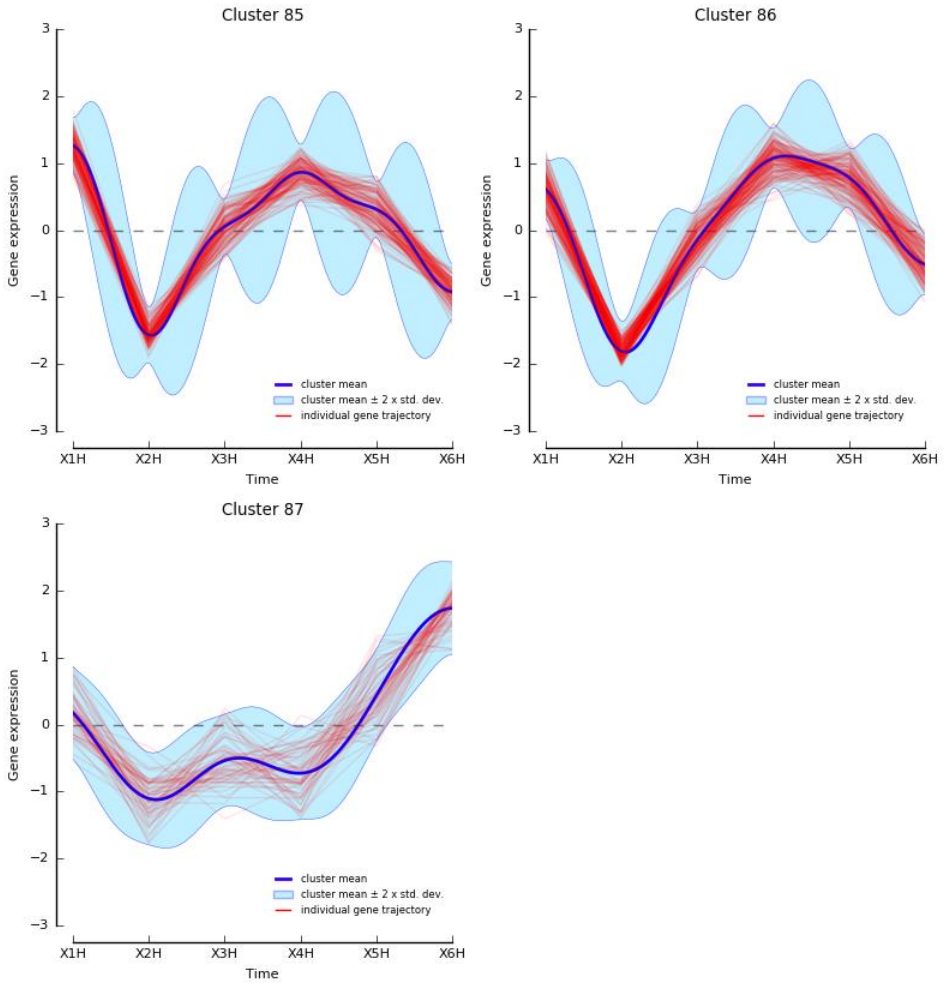

Extended Figure 16 I. Clustered trajectories of expressed genes across the early embryonic development of *B. oleae*.

Data analysis

Basecalling
Albacore (ONT, version 2.0.2)
read_fast5_basecaller.py -r --flowcell SQK-LSK108 --kit SQK-LSK108 --input %s --save_path %s --worker_threads 23 -o fastq" %(input_dir,save_path))

Minionqc [9] (version 1.0)
Rscript ~/MinionQC.R -p 23 -i $(‘pwd’)/files -o $(‘pwd’)/results

Pauvre (version 0.1.2, https://github.com/conchoecia/pauvre)
pauvre marginplot --no-transparent --fastq ../Bo_E_1H_C010_10_pass.fastq > pauvre.out 2> pauvre.out

Porechop (version 0.2.3, https://github.com/rrwick/Porechop)
~/porechop --format fasta -t 47 -i $read5.fasta -o $read5.choped.fasta > porechop.stdout 2> porechop.stdout

Cutadapt [55] poly(A) trimming from read ends (version 1.15)
~/.local/bin/cutadapt --info-file=trim_info -f fasta -a "A[100]" -o $read2.cutadapt.fasta $read2.fasta

GMAP [26] (version 2018-03-25)
GMAP for alignment QC
~/gmap -t 23 -D $dirc -f samse -d $ref $read1 > $outsam.sam

GMAP for transcriptome assembly
~/gmap -t 23 -D $dirc --cross-species --max-intronlength-ends=10000 -n 1 -z sense_force -f samse -d $ref $read1 > $outsam1.sam 2> gmap.stdout

Minimap2 [27] (version 2.9 (r720))
~/minimap2 -ax splice -t 23 $ref $reads1 > $outsam1.sam

Samtools [56] (version 1.3.2)

AlignQC [31] (version 1.2)
~/alignqc analyze $outsam1.sort.bam --specific_tempdir $dirc/tmp1 -r $ref -a $annotation -o alignqc.xhtml --output_folder $dirc/alignQC.ouput_b4_correction > alignqc.stdout

Canu [16] (Canu 1.7)
canu useGrid=false -correct gnuplotImageFormat=png corOutCoverage=10000 corMhapSensitivity=high corMinCoverage=0 correctedErrorRate=0.16 overlapper=minimap ovsMethod=sequential minReadLength=200 minOverlapLength=100 genomeSize=1500000000 -p Bo_E_all_pass_edited -d Bo_E_all_pass_edited -nanopore-raw Bo_E_all_pass_edited.fasta

LoRDEC [17] (v0.8, using GATB v1.4.1)

~/lordec-correct -2 $illumina_reads -T 47 -p -k 19 -s 3 -i $nanopore.fasta -o "$nanopore"_lordec_corrected.fasta

GFOLD [39] (v1.1.4)
gfold diff -norm NO -s1 Bo.E.2H -s2 Bo.E.1H -suf .abs_cnt3 -o Bo.E.2HvsBo.E.1H.abs.diff > Bo.E.2HvsBo.E.1H.abs.diff.stdout

cDNA_Cupcake (version 5.3, https://github.com/Magdoll/cDNA_Cupcake/wiki)
~/collapse_isoforms_by_sam.py --input $read1 -s $outsam.sorted.sam --dun-merge-5-shorter -o $pref
~/filter_by_count.py $pref.collapsed --min_count=2 >filter_by_count.stdout
~/filter_away_subset.py $pref.collapsed >filter_away_subset.stdout
~/filter_away_subset.py $pref.collapsed.min_fl_2
cDNA_Cupcake for assembly evaluation using 5-hour timepoint
~/collapse_isoforms_by_sam.py -c 0.95 -i 0.95 --input $read1 -s $sortedsam --dun-merge-5-shorter -o $pref

TAMA (version tc0.0, https://github.com/GenomeRIK/tama)
~/tama_collapse.py -d merge_dup -s $sortedsam -f $ref -p $pref -x no_cap -c 95 -i 95

TAPIS [20] (1.2.1)
alignPacBio.py -p 22 -v -K 10000 -o tapis_output $indexesDir $indexName $reference $reads
run_tapis.py -p -t 30 -o run_tapis_output $annotation tapis_output/$bamfile

SQANTI [22] (version 1.2)
sqanti_qc.py -z -t 47 -fl $fl_abundance -c $sj_covIllumina -e $isoExpression -x $gmapindex -o $output -d qc_output $isoforms.fa $gtf $ref
sqanti_filter.py -d filter_output -i "$isoforms"_corrected.fasta "$output"_classification.txt

Tomtom [42]
tomtom -no-ssc -oc . -verbosity 1 -min-overlap 5 -mi 1 -dist pearson -evalue -thresh 10.0 query_motifs db/FLY/fly_factor_survey.meme db/FLY/idmmpmm2009.meme db/FLY/flyreg.v2.meme db/FLY/OnTheFly_2014_Drosophila.meme db/FLY/dmmpmm2009.meme

DREME version 5.1.0 (Release date: Fri Oct 11 15:53:28 2019 -0700) [43]
dreme -verbosity 1 -oc . -dna -p zygoticEarly.promoter.fasta -n maternallydegradedCompletely.promoter.fasta -t 18000 -e 0.05

CentriMo version 5.1.0 (Release date: Fri Oct 11 15:53:28 2019 -0700) [41]
centrimo --oc . --verbosity 1 --local --score 5.0 --ethresh 10.0 --bfile zygoticEarly.promoter.fasta.bg zygoticEarly.promoter.fasta motifs.meme

PRAPI v1

sudo docker run -it --rm -v $[54]:/data prapi:v1 Pacbio_v16.py -c /data/basic/conf.txt >$path/prapi.stdout 2>$path/prapi.stdout

Max length for one internal intron

MaxIntron=500000

Width of peaks when searching for poly(A) sites

Width_of_peaks=5

##For test of difference between expression levels of zygotic and maternal genes we first checked for normality of data then did the test of difference:

shapiro.test(head(not_maternal_degraded_genes$TPE_1H, 1497))

wilcox.test(maternal_degraded_genes$TPE_1H,head(not_maternal_degraded_genes$TPE_1H, 1497),paired = FALSE,conf.level = 0.99)

References

1 Tzanakakis, M. E., Economopoulos, A. P. & Tsitsipis, J. A. The importance of conditions during the adult stage in evaluating an artificial food larvae of Dacus oleae (Gmelin) (Diptera: Tephritidae). *Zeitschrift für Angewandte Entomologie* **59**, 127-130, doi:10.1111/j.1439-0418.1967.tb03846.x (1967).

2 Winnebeck, E. C., Millar, C. D. & Warman, G. R. Why Does Insect RNA Look Degraded? *Journal of Insect Science* **10**, 159, doi:10.1673/031.010.14119 (2010).

3 Macharia, R. W., Ombura, F. L. & Aroko, E. O. Insects' RNA Profiling Reveals Absence of "Hidden Break" in 28S Ribosomal RNA Molecule of Onion Thrips, Thrips tabaci. *Journal of nucleic acids* **2015**, 965294, doi:10.1155/2015/965294 (2015).

4 Bayega, A. *et al.* Transcript Profiling Using Long-Read Sequencing Technologies. *Methods in molecular biology (Clifton, N.J.)* **1783**, 121-147, doi:10.1007/978-1-4939-7834-2_6 (2018).

5 Picelli, S. *et al.* Smart-seq2 for sensitive full-length transcriptome profiling in single cells. *Nature methods* **10**, 1096-1098, doi:10.1038/nmeth.2639 (2013).

6 Oikonomopoulos, S., Wang, Y. C., Djambazian, H., Badescu, D. & Ragoussis, J. Benchmarking of the Oxford Nanopore MinION sequencing for quantitative and qualitative assessment of cDNA populations. *Scientific reports* **6**, 31602, doi:10.1038/srep31602 (2016).

7 Sagri, E. *et al.* Olive fly transcriptomics analysis implicates energy metabolism genes in spinosad resistance. *BMC genomics* **15**, 714, doi:10.1186/1471-2164-15-714 (2014).

8 Sagri, E. *et al.* Housekeeping in Tephritid insects: the best gene choice for expression analyses in the medfly and the olive fly. *Scientific reports* **7**, 45634, doi:10.1038/srep45634 (2017).

9 Lanfear, R., Schalamun, M., Kainer, D., Wang, W. & Schwessinger, B. MinIONQC: fast and simple quality control for MinION sequencing data. *Bioinformatics (Oxford, England)*, doi:10.1093/bioinformatics/bty654 (2018).
10 Mavragani-Tsipidou, P., Karamanlidou, G., Zacharopoulou, A., Koliais, S. & Kastritisis, C. Mitotic and polytene chromosome analysis in Dacus oleae (Diptera: Tephritidae). *Genome* **35**, 373-378 (1992).
11 Bayega, A. *et al.* De novo assembly of the olive fruit fly (Bactrocera oleae) genome with linked-reads and long-read technologies minimizes gaps and provides exceptional Y chromosome assembly. *BMC genomics* **21**, 259, doi:10.1186/s12864-020-6672-3 (2020).
12 Martin, M. Cutadapt removes adapter sequences from high-throughput sequencing reads. *EMBnet.journal; Vol 17, No 1: Next Generation Sequencing Data Analysis* (2011).
13 Bolger, A. M., Lohse, M. & Usadel, B. Trimmomatic: a flexible trimmer for Illumina sequence data. *Bioinformatics (Oxford, England)* **30**, 2114-2120, doi:10.1093/bioinformatics/btu170 (2014).
14 Kim, D., Langmead, B. & Salzberg, S. L. HISAT: a fast spliced aligner with low memory requirements. *Nature methods* **12**, 357-360, doi:10.1038/nmeth.3317 (2015).
15 Li, B. & Dewey, C. N. RSEM: accurate transcript quantification from RNA-Seq data with or without a reference genome. *BMC bioinformatics* **12**, 323, doi:10.1186/1471-2105-12-323 (2011).
16 Koren, S. *et al.* Canu: scalable and accurate long-read assembly via adaptive k-mer weighting and repeat separation. *Genome research* **27**, 722-736, doi:10.1101/gr.215087.116 (2017).
17 Salmela, L. & Rivals, E. LoRDEC: accurate and efficient long read error correction. *Bioinformatics (Oxford, England)* **30**, 3506-3514, doi:10.1093/bioinformatics/btu538 (2014).
18 Richard, K. <https://github.com/GenomeRIK/tama Accessed 31/Aug/2019> (
19 Elizabeth, T.
20 Abdel-Ghany, S. E. *et al.* A survey of the sorghum transcriptome using single-molecule long reads. *Nature communications* **7**, 11706, doi:10.1038/ncomms11706 (2016).
21 Gao, Y. *et al.* PRAPI: post-transcriptional regulation analysis pipeline for Iso-Seq. *Bioinformatics (Oxford, England)* **34**, 1580-1582, doi:10.1093/bioinformatics/btx830 (2018).
22 Tardaguila, M. *et al.* SQANTI: extensive characterization of long-read transcript sequences for quality control in full-length transcriptome identification and quantification. *Genome research*, doi:10.1101/gr.222976.117 (2018).
23 Trapnell, C. *et al.* Differential gene and transcript expression analysis of RNA-seq experiments with TopHat and Cufflinks. *Nature Protocols* **7**, 562-578, doi:10.1038/nprot.2012.016 (2012).
24 Manousis, T. & Moore, N. F. Cricket Paralysis Virus, a Potential Control Agent for the Olive Fruit Fly, Dacus oleae Gmel. *Applied and environmental microbiology* **53**, 142-148 (1987).
25 Watanabe, M., Iwakawa, H. O., Tadakuma, H. & Tomari, Y. Biochemical and single-molecule analyses of the RNA silencing suppressing activity of CrPV-1A. *Nucleic acids research* **45**, 10837-10844, doi:10.1093/nar/gkx748 (2017).
26 Wu, T. D. & Watanabe, C. K. GMAP: a genomic mapping and alignment program for mRNA and EST sequences. *Bioinformatics (Oxford, England)* **21**, 1859-1875, doi:10.1093/bioinformatics/bti310 (2005).
27 Li, H. Minimap2: pairwise alignment for nucleotide sequences. *Bioinformatics (Oxford, England)*, doi:10.1093/bioinformatics/bty191 (2018).
28 Sovic, I. *et al.* Fast and sensitive mapping of nanopore sequencing reads with GraphMap. *Nature communications* **7**, 11307, doi:10.1038/ncomms11307 (2016).

29 Krizanovic, K., Echchiki, A., Roux, J. & Sikic, M. Evaluation of tools for long read RNA-seq splice-aware alignment. *Bioinformatics (Oxford, England)* **34**, 748-754, doi:10.1093/bioinformatics/btx668 (2018).
30 Chu, J., Mohamadi, H., Warren, R. L., Yang, C. & Birol, I. Innovations and challenges in detecting long read overlaps: an evaluation of the state-of-the-art. *Bioinformatics (Oxford, England)* **33**, 1261-1270, doi:10.1093/bioinformatics/btw811 (2017).
31 Weirather, J. L. *et al.* Comprehensive comparison of Pacific Biosciences and Oxford Nanopore Technologies and their applications to transcriptome analysis. *F1000Research* **6**, 100, doi:10.12688/f1000research.10571.2 (2017).
32 Byrne, A. *et al.* Nanopore long-read RNAseq reveals widespread transcriptional variation among the surface receptors of individual B cells. *Nature communications* **8**, 16027, doi:10.1038/ncomms16027

https://www.nature.com/articles/ncomms16027#supplementary-information (2017).
33 Byrne, A. *et al.* Nanopore long-read RNAseq reveals widespread transcriptional variation among the surface receptors of individual B cells. *Nature communications* **8**, 16027, doi:10.1038/ncomms16027 (2017).
34 Owens, N. D. L. *et al.* Measuring Absolute RNA Copy Numbers at High Temporal Resolution Reveals Transcriptome Kinetics in Development. *Cell reports* **14**, 632-647, doi:10.1016/j.celrep.2015.12.050 (2016).
35 Proposed methods for testing and selecting the ERCC external RNA controls. *BMC genomics* **6**, 150, doi:10.1186/1471-2164-6-150 (2005).
36 Jiang, L. *et al.* Synthetic spike-in standards for RNA-seq experiments. *Genome research* **21**, 1543-1551, doi:10.1101/gr.121095.111 (2011).
37 FlyBase--the Drosophila database. The FlyBase Consortium. *Nucleic acids research* **22**, 3456-3458 (1994).
38 Saccone, G., Pane, A. & Polito, L. C. Sex determination in flies, fruitflies and butterflies. *Genetica* **116**, 15-23 (2002).
39 Feng, J. *et al.* GFOLD: a generalized fold change for ranking differentially expressed genes from RNA-seq data. *Bioinformatics (Oxford, England)* **28**, 2782-2788, doi:10.1093/bioinformatics/bts515 (2012).
40 Fu, S., Shao, J., Zhou, C. & Hartung, J. S. Transcriptome analysis of sweet orange trees infected with 'Candidatus Liberibacter asiaticus' and two strains of Citrus Tristeza Virus. *BMC genomics* **17**, 349, doi:10.1186/s12864-016-2663-9 (2016).
41 Bailey, T. L. & Machanick, P. Inferring direct DNA binding from ChIP-seq. *Nucleic acids research* **40**, e128, doi:10.1093/nar/gks433 (2012).
42 Gupta, S., Stamatoyannopoulos, J. A., Bailey, T. L. & Noble, W. S. Quantifying similarity between motifs. *Genome Biol* **8**, R24, doi:10.1186/gb-2007-8-2-r24 (2007).
43 Bailey, T. L. DREME: motif discovery in transcription factor ChIP-seq data. *Bioinformatics (Oxford, England)* **27**, 1653-1659, doi:10.1093/bioinformatics/btr261 (2011).
44 Bailey, T. L. *et al.* MEME SUITE: tools for motif discovery and searching. *Nucleic acids research* **37**, W202-208, doi:10.1093/nar/gkp335 (2009).
45 Elkan, T. L. B. a. C. in *Proceedings of the Second International Conference on Intelligent Systems for Molecular Biology.* 28-36 (AAAI Press).
46 Ashburner, M. *et al.* Gene ontology: tool for the unification of biology. The Gene Ontology Consortium. *Nature genetics* **25**, 25-29, doi:10.1038/75556 (2000).

47 McDowell, I. C. *et al.* Clustering gene expression time series data using an infinite Gaussian process mixture model. *PLoS computational biology* **14**, e1005896, doi:10.1371/journal.pcbi.1005896 (2018).

48 Reimand, J., Kull, M., Peterson, H., Hansen, J. & Vilo, J. g:Profiler--a web-based toolset for functional profiling of gene lists from large-scale experiments. *Nucleic acids research* **35**, W193-200, doi:10.1093/nar/gkm226 (2007).

49 Penalva, L. O. & Sanchez, L. RNA binding protein sex-lethal (Sxl) and control of Drosophila sex determination and dosage compensation. *Microbiology and molecular biology reviews : MMBR* **67**, 343-359, table of contents (2003).

50 Lagos, D., Koukidou, M., Savakis, C. & Komitopoulou, K. The transformer gene in Bactrocera oleae: the genetic switch that determines its sex fate. *Insect molecular biology* **16**, 221-230, doi:10.1111/j.1365-2583.2006.00717.x (2007).

51 Lagos, D., Ruiz, M. F., Sanchez, L. & Komitopoulou, K. Isolation and characterization of the Bactrocera oleae genes orthologous to the sex determining Sex-lethal and doublesex genes of Drosophila melanogaster. *Gene* **348**, 111-121, doi:10.1016/j.gene.2004.12.053 (2005).

52 Gabrieli, P. *et al.* Sex and the single embryo: early deveiopment in the Mediterranean fruit fly, Ceratitis capitata. *BMC developmental biology* **10**, 12-12, doi:10.1186/1471-213X-10-12 (2010).

53 De Renzis, S., Elemento, O., Tavazoie, S. & Wieschaus, E. F. Unmasking activation of the zygotic genome using chromosomal deletions in the Drosophila embryo. *PLoS biology* **5**, e117, doi:10.1371/journal.pbio.0050117 (2007).

54 Meccariello, A. *et al.* Maleness-on-the-Y (MoY) orchestrates male sex determination in major agricultural fruit fly pests. *Science (New York, N.Y.)*, doi:10.1126/science.aax1318 (2019).

55 Martin, M. Cutadapt removes adapter sequences from high-throughput sequencing reads. *EMBnet.journal; Vol 17, No 1: Next Generation Sequencing Data AnalysisDO - 10.14806/ej.17.1.200* (2011).

56 Li, H. *et al.* The Sequence Alignment/Map format and SAMtools. *Bioinformatics (Oxford, England)* **25**, 2078-2079, doi:10.1093/bioinformatics/btp352 (2009).

Nanopore long-read RNA-seq and absolute quantification delineate transcription dynamics in early embryo development of an insect pest

Anthony Bayega, Spyros Oikonomopoulos, Maria-Eleni Gregoriou, Konstantina T Tsoumani, Antonis Giakountis, Yu Chang Wang, Kostas D Mathiopoulos, Jiannis Ragoussis

Protocol for cDNA synthesis and cDNA sequencing on the Oxford Nanopore Technologies (ONT) MinION platform

Materials and Reagents needed

General reagents

- RNase Zap (Thermo Fischer Scientific, AM9780)
- 1M Tris-HCl pH 8.0 (Thermo Fischer Scientific, AM9855G)
- Magnetic Stand for 1.5 mL tubes (e.g. Ambion P/N AM10026) and 0.2 mL tubes
- Filtered tips (10, 20, 100, 200, 1000 µL), and respective pipettes
- Eppendorf Centrifuge 5424R or 5424 (or equivalent)
- Micro-Centrifuge for 0.2 mL PCR tubes
- Qubit Assay Tubes (Thermo Fischer Scientific; Q32856)
- Qubit Fluorometer (Life Techonologies)
- VWR PCR 8-Tube Strip 0.2 mL (120 Strips) (VWR, 53509-304)
- Agilent TapeStation 2200 and the corresponding assay tubes
- Eppendorf DNA LoBind Tubes 1.5 mL (022431021)
- Mixer e.g HulaMixer (Thermo Fischer Scientific), Vortex Mixer (VWR)
- BioRad Thermo Cycler T100
- Agencourt AMPure XP beads (A63880, Beckman Coulter, 5 mL)
- Ethanol 100 % (reagent grade)

1 Reagents to assess the quality of the extracted RNA

Reagents for RNA Quantification

- Qubit RNA HS Assay Kit (Thermo Fischer Scientific, Q32852)

Reagents to examine the RNA profile

- Agilent RNA ScreenTape Ladder (Agilent; 5067-5578)
- Agilent RNA ScreenTape Sample Buffer (Agilent; 5067-5577)
- Agilent RNA ScreenTape (Agilent; 5067-5576)

2 Reagents for cDNA synthesis

Oligos and reconstitution buffer for cDNA synthesis primers:

- PolyT primer (V = A or C or G, N = A or C or G or T) (RNase-Free HPLC purification of the synthesized oligo is preferable. The oligo should be shipped lyophilized)

  5'-AAGCAGTGGTATCAACGCAGAGTATGCAACGCAACT$_{(30)}$VN -3'

- TSO oligo (TSO: Template-Switching oligonucleotide, the red marked bases are ribonucleotides. RNase-Free HPLC purification of the synthesized oligo is preferable. The oligo should be shipped lyophilized)

  5'- AAGCAGTGGTATCAACGCAGAGTGGATTCTATCACGCrGrGrG -3'

- THE RNA Storage Solution (Thermo Fischer Scientific, AM7000)

Reagents to remove DNA contamination in total RNA samples

- DNA-free DNA Removal Kit (Thermo Fischer Scientific, M1906)

Enzymes and reagents for the cDNA synthesis reaction:

- RNAse inhibitor 40 U/uL (Clontech, 2313A)
- Advantage UltraPure PCR Deoxynucleotide Mix (10 mM each dNTP) (Clontech, 639125)
- Water nuclease free PCR grade (eg. Affymetrix, 901578)
- SuperScript IV (Thermo Fischer Scientific, 18090010)
- Betaine (5M) (Sigma-Aldrich, B0300-1VL)
- $MgCl_2$ (1M) (Thermo Fisher Scientific, AM9530G)

Reagents to spike RNA molecules of known abundance inside the sample RNA:

- ERCC RNA Spike-In Mix (Thermo Fisher Scientific, 4456740)

3 Reagents for cDNA amplification:

Primer for cDNA amplification:

- cDNA amplification primer (Standard Desalting of the synthesized oligo can be ordered. The oligo can be shipped lyophilized or reconstituted at standard 100µM concentration.)

5'- /5Phos/ TCGTCGGCAGCGTCAAGCAGTGGTATCAACGCAGAGT-3'

Enzymes for cDNA amplification:

- Advantage 2 PCR Kit (Clontech, 639207)

4 Reagents for cDNA Quality Control:

Reagents for cDNA quantification

- Qubit HS DNA Assay Kit (Thermo, Q32851)

Reagents to examine the cDNA profile

- Agilent D5000 ScreenTape (Agilent, 5067-5588)
- Agilent D5000 Reagents (Agilent, 5067-5589)
- Agilent D5000 Ladder (Agilent, 5067-5590)

5 Reagents for cDNA library preparation for the nanopore platform

End repair of the cDNA molecules

- NEBNext End Repair Module (New England Biolabs, E6050S)

d(A) tailing of the cDNA molecules

- NEBNext dA-Tailing Module (New England Biolabs, E6053S)

Ligate ONT adapters on the cDNA molecules

- Ligation 1D Sequencing kit SQK-LSK108
- NEB Blunt/TA Master Mix (New England Biolabs, M0367S)
- Flow Cell Wash Kit (EXP-WSH002)

6 ONT MinION Sequencing

- MinION SpotON FLO-MIN106 flow cells
- MinION Mk1b

Preparation of reagents

1. The TSO oligo is reconstituted in “THE RNA Storage Solution” at a concentration of 1200 uM. *The information sheet from the manufacturer usually provides a dilution volume for a solution with a 100 uM oligo concentration. To create the solution with the 1200 uM oligo concentration, adjust the dilution volume accordingly by reducing 12X times the recommended volume presented on the information sheet*. Then 1 ul is diluted in 99ul of RNA storage solution (100X dilution; final concentration: 12 uM) and stored in aliquots at 5.6 ul per tube. The aliquots are stored at -80° C. The TSO ribonucleotides are prone to degradation. Loss of the ribonucleotides will lead in considerable reduction/absence of cDNA yield.
2. The PolyT primer is reconstituted in nuclease free H_20 at a concentration of 1200uM. *The information sheet from the manufacturer usually provides a dilution volume for a solution with a 100 uM oligo concentration. To create the solution with the 1200 uM oligo concentration, adjust the dilution volume accordingly by reducing 12X times the recommended volume presented on the information sheet*. Then 1 ul is diluted in 99ul of nuclease free H_20 and stored in aliquots at 7 ul per tube. The aliquots can be stored at -80° C.

RNA quantification

Total RNA was quantified using the "Qubit RNA HS Assay Kit" according to manufacturer instructions.

Assess DNA contamination in the RNA extraction

DNA contamination was measured using the Qubit dsDNA HS Reagent.

Removal of DNA contamination from total RNA

We used the DNA-free DNA Removal according to manufacturer instructions for the removal of DNA from RNA samples.

Assess the profile of the extracted RNA

Total RNA profile was determined using the Agilent RNA Screentape following manufacturer instructions except that the samples are not heated at 72 °C.

Spike-In RNA

ERCC (ERCC RNA Spike-In Mix 1) were added during the cDNA synthesis step. We aimed to obtain a final percentage of 5 % of our reads assigned to ERCCs assuming that ploy(A) fraction of total RNA is 5 %. We target the sequenced reads of the spiked-in RNA to be 5% of the total amount of sequenced reads). The amount of spiked RNA ($mass_{spiked\ RNA}$) that is going to be added in the reaction mix can be calculated as follows:

$$\mathbf{mass_{spiked\ RNA}} = \frac{\mathbf{fraction_{spiked\ reads}} \times \mathbf{fraction_{target\ RNA}} \times \mathbf{mass_{RNAinput}}}{\mathbf{Total\_RNA\_extracted}}$$

where:

mass $_{\text{spiked RNA}}$: mass (ngs) of spike-in RNA (SIRVs or ERCC) to be added in the sample.

fraction $_{\text{spiked reads}}$: desired fraction of sequenced spike-in RNA reads relative to the total amount of sequenced reads.

fraction $_{\text{target RNA}}$: fraction of the total RNA used in the sample, that is going to be synthesized into cDNA molecules.

mass $_{\text{RNA input}}$: mass (ngs) of RNA input per sample.

Then the volume (ul) of spike-in RNA to be used is calculated as follows:

$$\mathbf{volume_{spike-in\ RNA}} = \frac{\mathbf{mass_{spike-in\ RNA}}}{\mathbf{concentration_{spike-in\ RNA}}}$$

where

$\mathbf{concentration_{spike-in\ RNA}}$: concentration (ngs/ul) of the spike-in RNAs solution.

volume$_{\text{spike-in RNA}}$: volume (ul) from the spike-in RNAs solution to be added into the sample.

The value for the "mass $_{\text{RNA input}}$" is mass $_{\text{RNA input}}$ =300 ngs (We will use in the cDNA synthesis reactions 300 ngs of total RNA).

- For the ERCC RNA Spike-In Mix 1, the **mass $_{\text{spiked RNA}}$** = 0.45 ngs

1. The concentration of the stock solutions are:

- The "ERCC RNA Spike-In Mix 1" tube contains 10 µl of ERCC RNAs at a concentration of 103.515 fmoles/ul or 30.3 ng/ul .

- Prepare the appropriate dilution of each Spike-In Mix needed. In the new diluted solution the "mass $_{\text{spiked RNA}}$" for either the "ERCC RNA Spike-In Mix 1" or the "Spike-in RNA Variant (SIRVs) Control set 3 kit" should correspond, *if possible*, to 0.1 ul of the final diluted volume. So we need to have the following dilutions:
    - For the "ERCC RNA Spike-In Mix 1" we are going to dilute 6.72 times the stock solution. So in 5.72 ul of "THE RNA solution" add 1 ul from the "ERCC RNA Spike-In Mix 1" stock solution (new concentration= 4.5 ng/ul). Afterwards we will have to take Volume $_{\text{spike-in RNA}}$ = ((0.45 ngs)/(4.5 ng/ul))=0.1 ul of the diluted solution.

cDNA Library generation and sequencing on MinION

Generally, we followed the ONT "1D Strand switching cDNA by ligation (SQK-LSK108)" protocol but with custom cDNA synthesis protocol (as described below), and the end repair and d(A) tailing steps were performed separately. An overview of the protocol as follows:

1. cDNA synthesis and amplification
2. End-repair of cDNA molecules
3. dA-tail of cDNA molecules
4. Adapter ligation
5. Sequencing
6. Base-calling

cDNA synthesis

Our cDNA synthesis protocol involved a customized version of the Smart-seq protocol[1]. The protocol is based on the terminal deoxynucleotidyl transferase activity of the wild-type MMLV (Moloney murine leukemia virus) reverse transcriptase[2].

Preparation of Master Mixes

1. Thaw and vortex all reagents and keep master mixes on ice until use.
2. Label three 1.5 ml eppendorf tubes: "**pre-RT**", "**RT**", "**PCR**"
3. Always use fresh TSO primer as it is prone to degradation.
4. Prepare the "**pre-RT mix**" according to Table 1 below.

Table 1: **pre-RT mix**

	**pre-RT mix**	Total RNA (ul /sample)
1	ERCC RNA Spike-In Mix 1	x
2	RNase Inhibitor (40 U/uL * 125 uL = 5000U)	0.05
3	Poly-T primer (stock: 12 uM)	0.7
4	Superscript IV first-strand buffer (5×)	0.4
5	Nuclease free water	0.19
6	dNTP Mix (stock: 10 mM each)	0.56

	**Total =**	**2**

5. Pipette 2uL of **pre-RT mix** to a PCR tube and add 1uL of sample (**300ng of total RNA)**. Include a negative control (**1uL of water/RNA buffer**).
6. Incubate the samples in a thermocycler set according to Table 2 below.

Table 2: pre-RT incubation

**Temperature**	**Time**	**Purpose**
72°C	3 min	Unfolding of RNA secondary structures, Poly-T primer binding
4°C	10 min	Poly-T primer binds
25°C	1 min	Poly-T primer binds more specifically
4°C	Hold	

7 . Prepare the "**RT mix** according to Table 3

Table 3: **RT mix**

	**RT mix**	ul /sample
1	Nuclease free H_20	0.85
2	Superscript IV first-strand buffer (5×)	0.8
3	DTT (stock: 100 mM)	0.175
4	TSO (stock: 12 µM)	0.7
5	RNAse inhibitor (stock: 40 U/ µl)	0.175
6	SuperScript IV reverse transcriptase (stock: 200 U/ ul)	0.35
7	Betaine (stock: 5 M)	0.7
8	$MgCl_2$ (stock: 100 mM)	0.25
	**Total =**	**4**

8. Following pre_RT incubation, add 4 ul of RT mix to each sample, mix and briefly spin down.
9. Incubate the samples in a thermocycler set according to Table 4 below

Table 4: **SSIV RT protocol**

Temperature	Time	Cycle	Purpose
50°C	10 min	1	RT and template-switching
55°C	30 sec	10	Unfolding of RNA secondary structures
50°C	30 sec		Completion/continuation of RT
60°C	30 sec	5	Unfolding of RNA secondary structures
55°C	30 sec		Completion/continuation of RT
50°C	30 sec	1	Finish template switching
65°C	30 sec		Unfolding of RNA secondary structures
60°C	30 sec	5	Completion/continuation of RT
50°C	30 sec	1	Finish template switching
70°C	30 sec	5	Unfolding of RNA secondary structures
65°C	30 sec		Completion/continuation of RT
50°C	30 sec	1	Finish template switching
75°C	30 sec	5	Unfolding of RNA secondary structures
70°C	30 sec		Completion/continuation of RT
50°C	1 min	1	Final finish template switching
80°C	10 min	1	Enzyme inactivation
4°C	Hold	1	

10. Prepare the **PCR master mix** according to Table 5

Table 5: PCR master mix

	**PCR Mix**	(ul per 7 ul of RT reaction)
1	PCR-Grade Water	47.6
2	10X Advantage 2 PCR Buffer (not SA, short amplicon) (Advantage 2 PCR Kit)	7
3	50X dNTP Mix (Advantage 2 PCR Kit)	2.8
4	PCR primer (stock: 12 µM)	2.8
5	50X Advantage 2 Polymerase Mix (Advantage 2 PCR Kit)	2.8
	**Total =**	**63**

11. Following RT incubation, add 63 ul of PCR mix to each sample, mix and briefly spin down
12. Incubate the samples in a thermocycler set according to Table 6 below

Table 6: PCR protocol

Temperature	Time	Cycle
95°C	1 min	1
95°C	20 sec	5
58°C	4 min	
68°C	6 min	
95°C	20 sec	11 or 12 cycles , as many to produce around 1-2 ug of cDNA per 70 ul of PCR amplification reaction
64°C	30 sec	
68°C	6 min	
72°C	10 min	1
4°C	Hold	1

13. The amplified product is subsequently cleaned with Agencourt AMPure XP beads as is described below.

Agencourt AMPure XP cleanup of cDNA amplification products

1. Allow AMPure XP beads to equilibrate to room temperature for at least 30 minutes.
2. Vortex the beads until evenly mixed, then add 0.9X sample volume of Agencourt AMPure XP beads to the sample in the same tube as used for PCR.
3. Pipet the entire volume up and down to mix thoroughly. Place the sample tubes on a roler mix for 5 - 8 minutes to let the DNA bind to the beads. Briefly spin the samples to collect the liquid from the side of the tube.
4. Place the sample tubes on the magnetic separation device for ~2 minutes until the liquid appears completely clear, and there are no beads left in the supernatant.
5. While the samples are on the magnetic separation device, pipette out the supernatants. Keep the samples on the magnetic separation device. Add 200 µl of freshly made 80% ethanol to each sample without disturbing the beads. Wait for 30 seconds and carefully pipette out the supernatant containing contaminants.
6. DNA will remain bound to the beads during the washing process. Repeat step 4 once more. Briefly spin the samples to collect the liquid from the side of the wall.
7. Place the samples on the magnetic device for 30 seconds, then remove all the remaining ethanol with a pipette.
8. Place the samples at room temperature until the pellet appears dry (~ 5 minutes). You may see a tiny crack in the pellet when it is dry.
9. Once the beads are dry, add 51 µl of TE buffer to cover the bead pellet.

10. Remove the samples from the magnetic separation device and mix thoroughly to resuspend the beads. Incubate the sample with rotation at room temperature for 5 – 8 minutes.
11. Put the tubes on the magnet and after ~2 minutes recover the supernatant which should contain the cleaned amplified cDNA. Determine the quantity of the cDNA and profile using Qubit HS DNA Assay Kit and Agilent D5000 Tapestation, respectively, following manufacturer instructions.

End -repair of DNA

End repair of 1 µg of amplified cDNA was carried out using NEBNext End Repair Module (New England Biolabs, E6050S) following manufacturer instructions. This was followed by 0.9X Ampure XP beads cleanup (described above).

dA-tailing reaction

d(A) tailing of the recovered end-repaired cDNA was carried out using NEBNext dA-Tailing Module (New England Biolabs, E6053S) following manufacturer instructions. This was followed by 0.9X Ampure XP beads cleanup (described above).

Adapter ligation

Ligation of ONT sequencing adapters onto recovered d(A)-tailed cDNA (up to 1 µg) was carried out following ONT SQK-LSK-108 protocol. However, we increased the incubation time from 10 minutes to 1 - 4 hours at room temperature.

ONT MinION sequencing kit

ONT SQK-LSK-108 protocol was followed for the sequencing part.

Basecalling

We performed our basecalling off-line using Albacore version 2.0.2

Data analysis

Basecalling
Albacore (ONT, version 2.0.2)
read_fast5_basecaller.py -r --flowcell SQK-LSK108 --kit SQK-LSK108 --input %s --save_path %s --worker_threads 23 -o fastq" %(input_dir,save_path))

Minionqc[3] (version 1.0)
Rscript ~/MinionQC.R -p 23 -i $(‘pwd’)/files -o $(‘pwd’)/results

Pauvre (version 0.1.2, https://github.com/conchoecia/pauvre)
pauvre marginplot --no-transparent --fastq ../Bo_E_1H_C010_10_pass.fastq > pauvre.out 2> pauvre.out

Porechop (version 0.2.3, https://github.com/rrwick/Porechop)
~/porechop --format fasta -t 47 -i $read5.fasta -o $read5.choped.fasta > porechop.stdout 2> porechop.stdout

Cutadapt[4] poly(A) trimming from read ends (version 1.15)
~/.local/bin/cutadapt --info-file=trim_info -f fasta -a "A[100]" -o $read2.cutadapt.fasta $read2.fasta

GMAP[5] (version 2018-03-25)
GMAP for alignment QC
~/gmap -t 23 -D $dirc -f samse -d $ref $read1 > $outsam.sam

GMAP for transcriptome assembly
~/gmap -t 23 -D $dirc --cross-species --max-intronlength-ends=10000 -n 1 -z sense_force -f samse -d $ref $read1 > $outsam1.sam 2> gmap.stdout

Minimap2[6] (version 2.9 (r720))
~/minimap2 -ax splice -t 23 $ref $reads1 > $outsam1.sam

Samtools[7] (version 1.3.2)

AlignQC[8] (version 1.2)
~/alignqc analyze $outsam1.sort.bam --specific_tempdir $dirc/tmp1 -r $ref -a $annotation -o alignqc.xhtml --output_folder $dirc/alignQC.ouput_b4_correction > alignqc.stdout

Canu[9] (Canu 1.7)
canu useGrid=false -correct gnuplotImageFormat=png corOutCoverage=10000 corMhapSensitivity=high corMinCoverage=0 correctedErrorRate=0.16 overlapper=minimap ovsMethod=sequential minReadLength=200 minOverlapLength=100 genomeSize=1500000000 -p Bo_E_all_pass_edited -d Bo_E_all_pass_edited -nanopore-raw Bo_E_all_pass_edited.fasta

LoRDEC[10] (v0.8, using GATB v1.4.1)

~/lordec-correct -2 $illumina_reads -T 47 -p -k 19 -s 3 -i $nanopore.fasta -o "$nanopore"_lordec_corrected.fasta

GFOLD[11] (v1.1.4)
gfold diff -norm NO -s1 Bo.E.2H -s2 Bo.E.1H -suf .abs_cnt3 -o Bo.E.2HvsBo.E.1H.abs.diff > Bo.E.2HvsBo.E.1H.abs.diff.stdout

cDNA_Cupcake (version 5.3, https://github.com/Magdoll/cDNA_Cupcake/wiki)
~/collapse_isoforms_by_sam.py --input $read1 -s $outsam.sorted.sam --dun-merge-5-shorter -o $pref

```
~/filter_by_count.py $pref.collapsed --min_count=2 >filter_by_count.stdout
~/filter_away_subset.py $pref.collapsed >filter_away_subset.stdout
~/filter_away_subset.py $pref.collapsed.min_fl_2
```

cDNA_Cupcake for assembly evaluation using 5-hour timepoint

```
~/collapse_isoforms_by_sam.py -c 0.95 -i 0.95 --input $read1 -s $sortedsam --dun-merge-5-shorter -o $pref
```

TAMA (version tc0.0, https://github.com/GenomeRIK/tama)

```
~/tama_collapse.py -d merge_dup -s $sortedsam -f $ref -p $pref -x no_cap -c 95 -i 95
```

TAPIS[12] (1.2.1)

```
alignPacBio.py -p 22 -v -K 10000 -o tapis_output $indexesDir $indexName $reference $reads
run_tapis.py -p -t 30 -o run_tapis_output $annotation tapis_output/$bamfile
```

SQANTI[13] (version 1.2)

```
sqanti_qc.py -z -t 47 -fl $fl_abundance -c $sj_covIllumina -e $isoExpression -x $gmapindex -o $output -d qc_output $isoforms.fa $gtf $ref
sqanti_filter.py -d filter_output  -i "$isoforms"_corrected.fasta "$output"_classification.txt
```

References

1 Ramskold, D. *et al.* Full-length mRNA-Seq from single-cell levels of RNA and individual circulating tumor cells. *Nat Biotechnol* **30**, 777-782, doi:10.1038/nbt.2282 (2012).

2 Zajac, P., Islam, S., Hochgerner, H., Lonnerberg, P. & Linnarsson, S. Base preferences in non-templated nucleotide incorporation by MMLV-derived reverse transcriptases. *PLoS One* **8**, e85270, doi:10.1371/journal.pone.0085270 (2013).

3 Lanfear, R., Schalamun, M., Kainer, D., Wang, W. & Schwessinger, B. MinIONQC: fast and simple quality control for MinION sequencing data. *Bioinformatics (Oxford, England)*, doi:10.1093/bioinformatics/bty654 (2018).

4 Martin, M. Cutadapt removes adapter sequences from high-throughput sequencing reads. *EMBnet.journal; Vol 17, No 1: Next Generation Sequencing Data AnalysisDO - 10.14806/ej.17.1.200* (2011).

5 Wu, T. D. & Watanabe, C. K. GMAP: a genomic mapping and alignment program for mRNA and EST sequences. *Bioinformatics (Oxford, England)* **21**, 1859-1875, doi:10.1093/bioinformatics/bti310 (2005).

6 Li, H. Minimap2: pairwise alignment for nucleotide sequences. *Bioinformatics (Oxford, England)*, doi:10.1093/bioinformatics/bty191 (2018).

7 Li, H. *et al.* The Sequence Alignment/Map format and SAMtools. *Bioinformatics (Oxford, England)* **25**, 2078-2079, doi:10.1093/bioinformatics/btp352 (2009).

8 Weirather, J. L. *et al.* Comprehensive comparison of Pacific Biosciences and Oxford Nanopore Technologies and their applications to transcriptome analysis. *F1000Research* **6**, 100, doi:10.12688/f1000research.10571.2 (2017).

9 Koren, S. *et al.* Canu: scalable and accurate long-read assembly via adaptive k-mer weighting and repeat separation. *Genome research* **27**, 722-736, doi:10.1101/gr.215087.116 (2017).

10 Salmela, L. & Rivals, E. LoRDEC: accurate and efficient long read error correction. *Bioinformatics (Oxford, England)* **30**, 3506-3514, doi:10.1093/bioinformatics/btu538 (2014).

11 Feng, J. *et al.* GFOLD: a generalized fold change for ranking differentially expressed genes from RNA-seq data. *Bioinformatics (Oxford, England)* **28**, 2782-2788, doi:10.1093/bioinformatics/bts515 (2012).

12 Abdel-Ghany, S. E. *et al.* A survey of the sorghum transcriptome using single-molecule long reads. *Nature communications* **7**, 11706, doi:10.1038/ncomms11706 (2016).

13 Tardaguila, M. *et al.* SQANTI: extensive characterization of long-read transcript sequences for quality control in full-length transcriptome identification and quantification. *Genome research*, doi:10.1101/gr.222976.117 (2018).
